# Using broadband infrastructure as a social sensor to detect inequities in unemployment during the COVID-19 pandemic

**DOI:** 10.1038/s41598-023-48019-2

**Published:** 2023-12-12

**Authors:** Nicola Ritsch, Daniel Erian Armanios

**Affiliations:** 1 Department of Engineering and Public Policy, Carnegie Mellon University, Pittsburgh, 15213 USA; 2 Saïd Business School, University of Oxford, Oxford, OX1 1HP UK

**Keywords:** Civil engineering, Statistics, Information technology, Psychology and behaviour, Natural hazards

## Abstract

This study explores the potential of using physical infrastructure as a “social sensor” for identifying marginalized communities. Prior work tends to explore biases in infrastructure as a retrospective “social autopsy”. Instead, our study aims to create an introspective “social biopsy”, using existing infrastructure gaps to inform how future policy and investment can address existing inequities more sharply and proactively. Specifically, this work explores the possibility of using U.S. county-level broadband penetration rates as a social sensor to predict rates of unemployment amidst the COVID-19 pandemic. The result is a 2 × 2 typology of where broadband as a social sensor is sharper (or coarser), as well as prone to error (either false positives or false negatives). We further explore combining broadband with other forms of physical infrastructure (i.e., bridges, buildings, and WiFi-enabled libraries) to create a sensor “array” to further enhance detection. Overall, this work proposes an “infrastructure-as-sensor” approach to better detect social vulnerability during times of crises in hopes of enhancing resilience through providing services more quickly and precisely to those who most need it.

## Introduction

While the built environment has long been known to impact human behavior^1^, recent computational advancements and data availability allow us to explore such effects at unprecedented granularity and resolution. Particular configurations of infrastructure, ranging from clusters of buildings and bridges down to a single building and bridge site, are shown to impact mobility, decision-making, response time, well-being, and even entrepreneurship^2–5^. Such an approach is precise enough to detect behavior amidst crises as global as a pandemic^6^, and as local as an active shooter situation^7^. Moreover, a series of scaffolding technologies have now been shown to enhance data acquisition, communication, and coordination around such systems. These range from sensors measuring floor vibrations induced by an individual^8,9^ all the way to sensors used for social media tracking^10^ and digital twins^11,12^, as well as augmented and virtual reality operating across these individual and systems-level scales^13–17^.

Our study aims to take the conceptual terrain from this important body of work and expand it to address issues of equity. Seminal work undergirding this area^18,19^ find that not everyone equally benefits from infrastructure or the technologies that support it; infrastructure linkages to local communities are often skewed and biased^6,20–25^. However to borrow from a medical analogy used in this space, prior studies tend to evaluate infrastructure systems, and the calamities which impact such systems, as a retrospective post-mortem “social autopsy”^26^.

Our study asks can we transform such analyses into a “social biopsy” (rather than a social autopsy), allowing for a more immediate and introspective sampling of infrastructure gaps to identify and alleviate bias in real-time? We see a possibility for doing so by treating infrastructure as a *social sensor* of such bias and inequity. In other words instead of just analyzing how infrastructure was skewed by past bias, we treat current infrastructure asymmetries as characterizing the present impacts of said bias (i.e., infrastructure → present bias instead of past bias → infrastructure). In taking a social sensor approach, this study seeks to transcend the prevailing approach of documenting biases in existing infrastructure in order to start crafting more real-time solutions to alleviate such challenges.

Treating infrastructure as a social sensor complements the prevailing human-as-sensors approach^9,11^ which focuses on developing sensing mechanisms that are sensitive to an individual’s activity, thereby identifying human behavior and mobility at ever more precise and granular resolutions. However, scaling this latter human-as-sensors approach is computationally, capital, and environmentally intensive^27,28^. Currently, prevailing solutions are algorithmic in nature, whereby the approach is either creating more parsimonious algorithms that require less computational resources^27,28^, or optimizations that help increase savings by leveraging differences across a set of infrastructure assets^29^. Moreover, these approaches (1) rely primarily on propriety data which is challenging to make reproducible or available for government use, let alone do so in a way that is adequately sensitive to privacy issues; and (2) largely propose and evaluate possible sensors rather than provide guidelines for sensor choice and deployment.

Our “infrastructure-as-sensor” method proposes an alternative synergistic approach. In identifying those infrastructure configurations that lead to the most bias and inequity, we can better identify where to deploy more granular human-as-sensor approaches in ways that can realize greater benefits at less cost. In this manner, infrastructure-informed targeting could help further minimize costs by bringing into greater focus those areas that are most likely suffering from infrastructure-driven bias for which human-as-sensors approaches could more efficiently and effectively be deployed to better track and mitigate. Our approach uses thinking in urban studies^1^ and sociology^19,26^ to inform infrastructure sensor choice for these purposes. Furthermore, our approach relies only on public data, ensuring that the methods and outcomes of such research can be reproducible as well as be pragmatically integrated and deployed by government agencies in decision making processes. Overall while most prior sensing approaches rely on private data or indirect capture of inequity (i.e., social media)^30–32^, our approach sees what is feasible with more publicly available, and therefore more easily and cheaply accessible, data on infrastructure networks closer to the physical enablers and barriers of bias and inequity.

To explore the feasibility of our proposed infrastructure-as-sensor approach, we explore the asymmetries in broadband deployment and how that impacted unemployment amidst COVID-19. We chose this setting for several reasons. First, there is already an extensive literature that documents the impacts of broadband penetration and availability on unemployment (for a more detailed review, see SI Appendix A)^33–40^. Therefore, we keep the study’s focus on whether infrastructure is a useful social sensor of marginalized groups, especially those sensitive to the linkages between broadband and unemployment. Second, we find that economic vulnerability is best captured through unemployment rather than other commonly used metrics such as wages. As Fig. F11 in the SI shows, wages largely do not change while employment does during COVID-19, which suggests unemployment is a better more immediate benchmark of vulnerability. To our knowledge, there are only two other studies which explore the impact of broadband access during the COVID-19 pandemic. The first piece primarily focuses on the ability of an individual to work from home in light of their broadband connectivity and does not focus on what the lack of broadband connectivity may mean for employment^41^. The second finds that broadband adoption and availability may be associated with employment in rural America during March and April of 2020^42^. However, this work does not cover the entire United States, uses a limited set of broadband measures, and does not use empirical methods which allow for analyzing the change in unemployment before vs. during COVID-19.

Second, COVID-19 is a useful multidimensional shock. COVID-19 led to stay-at-home mandates implemented by all states, which required individuals to work from home if they were performing non-essential work (the dates for which are documented in SI Appendix B). This also required children to conduct much of their schooling from home and led to business shutdowns due to inability to patronize local establishments. While this shock has been used as an effective natural experiment in other studies^43^, the prevailing view with regards to broadband is that connectivity impacts unemployment through enhanced digital connectivity^33^. Given this is the case, broadband should largely influence those aspects of COVID-19 such as stay-at-home mandates for work or schooling that necessitate such digital connectivity. However, broadband deployment should not drive other aspects that do not depend on digital connectivity, namely business closures due to people unable to patron establishments such as hotels, restaurants, and other service-oriented firms. This multidimensional nature of the pandemic gives us a unique opportunity to gauge where infrastructure as a social sensor should increase sharpness and where it should increase coarseness (i.e., shed insight on those groups and facets for which the infrastructure provision should impact resilience vs. those that it should not).

As informed by prior theory^1,21,25^, our core proposition of this study is that infrastructure can serve as a social sensor that allows for sharper detection of groups whom are most vulnerable to disruption. From that core proposition, we develop two hypotheses that are specific to the context of broadband access and the COVID-19 stay-at-home mandates, both of which focus on the sensitivity of social sensors:

Hypothesis 1 (Social Sensor Sharpness): Populations that depend on broadband for work (i.e., people who work from home and people employed in the tech industry), or who historically lack broadband access (i.e., Latino/Hispanic and African American/Black households and Single Parent and Low-Income households) will exhibit greater rates of unemployment from COVID-19 due to broadband access.

Hypothesis 2 (Social Sensor Coarseness): Populations with less reliance on broadband for their employment (i.e. people who are employed in service work or essential industries and Rural communities) will exhibit lower rates of unemployment from COVID-19 due to broadband access.

As with any sensor, we expect there will be some slippage and error in the accuracy of this sensor. Thus, we also explore the following two boundary conditions:

Boundary Condition 1 (sensor slippage): Dimensions that are further away from what the sensor measures (i.e., broadband-dependent work) will exhibit weaker signaling effects.

Boundary Condition 2 (sensor array): Triangulating across multiple sensors will improve targeting along the most key dimensions and to the most critical locations.

We now proceed to present the main results and how different groups are (or are not) impacted by broadband access amidst the COVID-19 stay-at-home mandates. To empirically analyze boundary condition 1, we split cases into three subsets: (1) those that directly depend on broadband for work; (2) those that indirectly measure broadband access; and (3) those that do not directly depend on broadband. The intuition is that our social sensor will exhibit the strongest signals for cases in set (1) and the weakest for cases in set (3). To empirically analyze boundary condition 2, we then conduct a proof-of-concept supplementary analysis of how one could scale from broadband as a single senor measuring high-speed Internet access to an “array” of sensors that incorporate the built environment to see if we can more holistically and precisely capture such vulnerabilities. This includes buildings, bridges, and WiFi-enabled libraries. We conclude with a summary of the findings and discuss the implications and possible uses for this infrastructure-as-sensor approach.

## Results

### Mainline results for broadband: asymmetries in broadband access increase unemployment

Before discussing the results, we must first define empirically our key variable, namely what we mean by broadband access. We start with the definition used by the Federal Communications Commission (FCC) which states that broadband is “high-speed internet access that is always on and faster than the traditional dial-up access”. They define adequate broadband access for an individual as at least 25 Mbps upload speed and 3 Mbps download speed^44^. Broadband access can be measured in terms of several different metrics including a) actual speed (measured by Microsoft (MSFT), Ookla and MLab), b) advertised availability (measured by the FCC), and c) adoption (measured by the American Community Survey (ACS)). Therefore, our operating definition of broadband access is *the degree to which an individual can access high-speed internet as measured either in terms of self-reported access, advertised speed, and/or actual speed tests.* For the purposes of this study, “adequate” either means 50% or more of the population has 25 Mbps upload and 3 Mbps download in terms of advertised or actual speed, or 50% of the population or more report access to broadband. This definition aligns with those of other well-regarded sources^45^. We discuss the differences in these broadband metrics further in the Methods section and in SI Appendices C, D, and E. Table 1 also highlights how each metric is measured.

**Table 1:** A summary of each broadband metric used and an explanation of how it is measured and collected.

	FCC form 477^72^	M-Lab NDT^72^	Ookla speedtest^72^	ACS census	Microsoft
Type of measurement	Advertised availability	Actual speed	Actual speed	Adoption	Actual speed
Geographic precision	Census Block	Aggregated by any geography, though smaller than county or city not recommended	Aggregated in a grid of ~ 610.8 m^2^ tiles which can be used down to census tract level	Census tract	County
Metrics	Internet service provider (ISP reports) maximum advertised upload and download link capacity by access type (fiber, cable, direct subscriber lines, satellite and fixed wireless)	Single stream Transmission Control Protocol (TCP) measure of Bulk Transport Capacity. Measures the download & upload speed and latency the user’s device is getting from the router	Multi-stream TCP measure estimating link capacity. Measures the average of download/upload speeds, average latency, # tests, and # devices that the user’s router device is getting from their ISP	Individual reported access	Multi-stream TCP measure estimating link capacity. Percentage of population with 25/3 download and upload speed
Data collection	ISP-contributed, free and open access to aggregate data. Collection method left to the ISP	User-contributed, free and open access to individual data points. Open source server run by M-Lab	User-contributed, free and open access to aggregate data. Closed source server that is available for others to run	Census workers collect from individual households	User-contributed whenever user utilizes Microsoft product. Free and open to access

**Figure 1:**
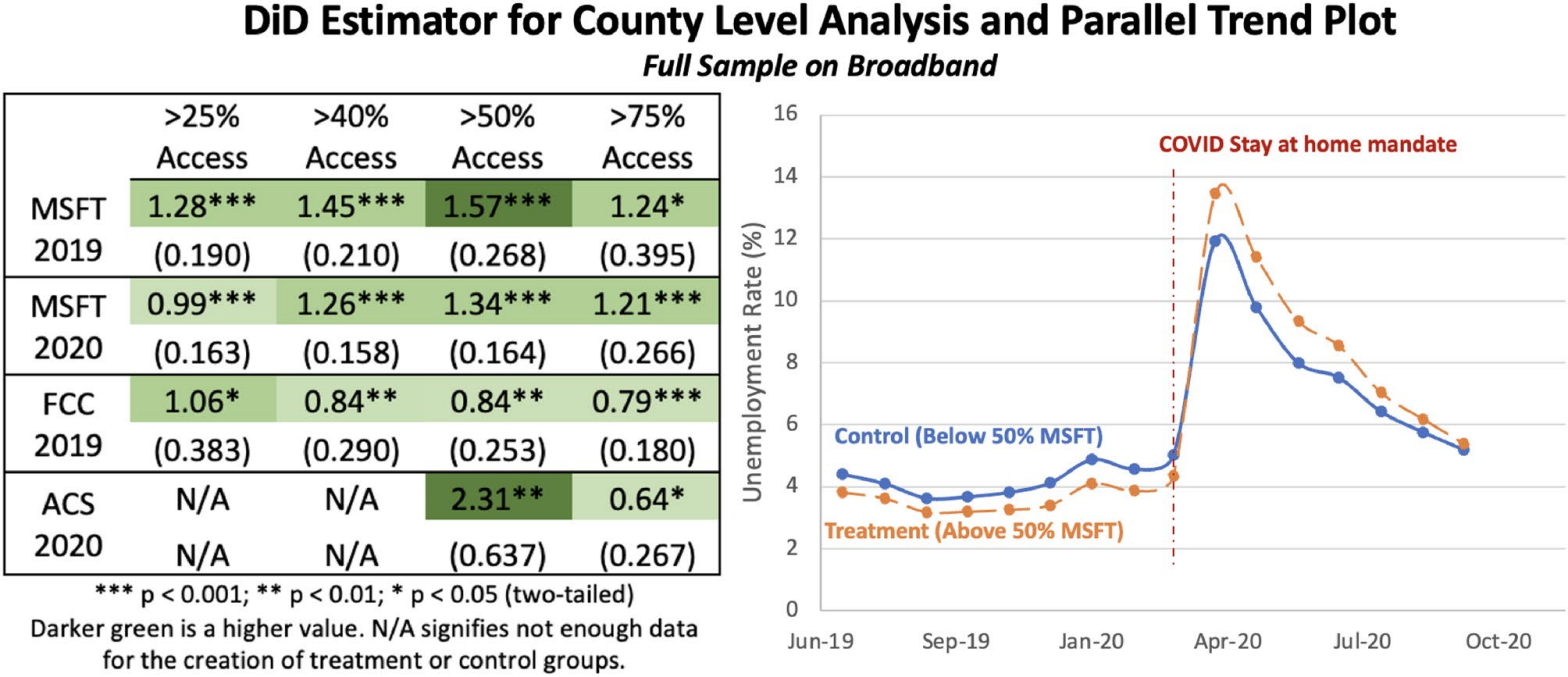
Left: difference-in-difference estimators for the full dataset. Robust standard errors, clustered at the state level, are included below each estimate with statistical significance indicated by the stars based off of a two-tailed test. In the base case (MSFT 2020 data, 50% penetration) when all else equal, after COVID, counties with more than 50% access to 25 Mbps download and 3 Mbps upload, experience an increase of 1.34% in their unemployment rate over similar counties that have less than 50% access. Right: parallel trends between the control (below adequate access to broadband at a county level for MSFT 2020) and treatment (above adequate access to broadband on average at a county level for MSFT 2020). Parallel trends hold prior to the shock of the COVID-19 pandemic.

Figure 1 presents our base case results exploring the efficacy of using broadband as a social sensor to detect unemployment during the COVID-19 pandemic. When we refer to the “base case” throughout the study, we are referring to the use of the Microsoft (MSFT) 2020 dataset to separate out treated counties (50% or more broadband penetration) and control counties (less than 50% broadband penetration).We use this as the base case for our work because this data represents actual user speeds and is the most publicly accessible dataset with the widest geographic coverage. In this base case, when all else is equal after the shock of the stay-at-home orders, we find that counties with more than 50% of the population having access to 25 Mbps download/3 Mbps upload broadband speeds experience an increase of 1.34% in their unemployment rate over similar counties that have less than 50% access. Presented on the right-hand size of Fig. 1 is the parallel trends plot for the base regression which shows that the unemployment rates for both treatment and control counties remain constant prior to the impact of COVID, which visually demonstrates that the parallel trends assumption is upheld (Fig. 7 provides a more formalized statistical test). We also conduct a synthetic controls analysis, which relaxes the parallel trends assumption and combines this approach with fixed effects as explained in prior work ^46^ (see Table 3 and Appendix H for more details), and results remain robust to this approach. Presented on the left-hand side of Fig. 1 are the difference-in-differences (DiD) estimators for each kind of broadband data considered in the study, using varying levels of penetration to define the control and treatment groups in the regression models (cases using M-Lab and Ookla data are available in Appendix G). When using MSFT, FCC or ACS as the treatment lens, this positive impact on unemployment remains robust across these different datasets and across different penetration levels (i.e., 25%, 40%, 50%, and 75%). While the results are robust across indicators, they do demonstrate variation based on what is being measured. At 50%, the results range from an increase of 0.84% in unemployment reported from FCC 2019 data to an increase of 2.31% in unemployment as reported by the ACS 2020 data. The regression models that serve as the basis for the heat maps in Fig. 1 are in SI Appendix I.

One may find this baseline result surprising as one would surmise greater broadband access means easier ability to stay-at-home to work, which should better ensure individuals can stay employed. However, what this immediate intuition does not consider is the tension between how broadband access is typically measured vs. what prior empirics, especially in the social sciences, tell us about how infrastructure is distributed. This has important empirical and conceptual implications.

Empirically, all sets of publicly available broadband data are currently *measured by spatial area*. While this is the convention in prior work^33,47,48^, a tacit assumption then is that access levels are *evenly distributed* across the spatial unit of analysis. For example, if we measure that 90% of the population within a spatial unit has adequate broadband access, the expectation is that 90% is evenly scattered across the unit. In our case, let’s assume a county has 100 neighborhoods, each with equivalent populations, this implies 90% of the people in each neighborhood has adequate access. However, several studies note that infrastructure access is actually not evenly distributed^18,21,49,50^. In other words, let’s revisit our fictitious county with 100 neighborhoods. The reality could be that if we measure on average 90% of a population within a county that has adequate access, perhaps 100% of the population in 90 of the county’s neighborhoods have adequate access but no one in the remaining 10 neighborhoods have adequate access. This would still arrive at an average of 90% coverage. For illustrative purposes in this example, we assume uneven distribution is based on space, but such unevenness can also be based on specific industries or populations that are known to have asymmetric need for broadband or access to it. We will discuss this in the next section.

Conceptually then, those without access being confined to ever more concentrated areas may be “out of sight, out mind”. If employers increasingly see that those around them have broadband access, then they are likely to assume everyone has access. In essence, what we theoretically argue is happening is a “halo effect”, whereby employers assume everyone can access broadband to continue work as they increasingly do not observe those ever concentrated few without such access.

**Figure 2:**
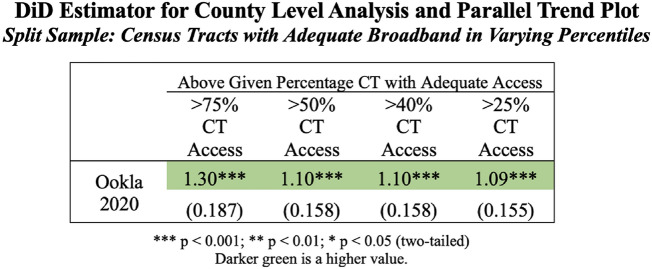
Left: difference-in-difference estimators for the dataset, subset by percentage of census-tracts which have access to adequate broadband within the treated group of counties. Robust standard errors, clustered at the state level, are included below each estimate with statistical significance indicated by the stars based off of a two-tailed test. In the base case (Ookla data, 50% of census-tracts with access in the county) when all else is equal, after COVID, counties with more than 50% of their census-tracts having adequate access to broadband experience an increase of 1.10% in their unemployment rate over similar counties that have less than 50% of the census-tracts in their county with adequate access. The plot of the parallel trends between the control (below adequate access to broadband at a county level) and treatment (above adequate access to broadband on average at a county level, parsed out by counties with varying levels of census tracts with adequate broadband access) is available in Appendix G. The parallel trends hold prior to the shock of the COVID-19 pandemic, and while the differences between treated and controls groups are small they can be observed in the plots. The ideas here is to assess whether concentrated in-access is what drives these potential effects. The full regressions that underpin these results are in SI Appendix I.

Considering these empirical and conceptual considerations, we would expect unemployment is sensitive to lack of access being confined to a specific set of neighborhoods, especially for counties where the majority have adequate access. We find evidence that this may indeed be the case. To gain insight into this, we sought census tract data that is the finest grained publicly available data that approximates the level of a neighborhood. While ACS, FCC and Ookla datasets all release broadband penetration estimates at the census-tract level annually, unemployment records at the census-tract level are only available from the ACS 5-year estimates, failing to provide the monthly temporal resolution required to run the prescribed DiD regression models. In order to make use of the data which is available, Fig. 2 shows the DiD estimator for sub-groups of counties which have above a given number of census-tracts with broadband access. In this subset, we use Ookla as our base case as this is the only dataset available at both a county and a census-tract level which measures quality of broadband speed thresholds. We find in Fig. 2 that as we subset the treated counties to ever greater percentages of census tracts with adequate (25%, 40%, 50%, and 75%) access, we see the unemployment rate increase between the treated vs. control counties. As we argue and anticipate, this suggests that lack of access is not evenly distributed but concentrated and when it is, employers are arguably more likely not able to adequately detect and accommodate those without adequate broadband access. This further implies that broadband may be a useful social sensor in detecting gaps at ever finer spatial scales to more sharply identify those groups which most need broadband support in times of crises such as during a pandemic.

### Fine tuning the sensor using socioeconomic and sociodemographic data

In light of the findings at this point, our aim now is to detect gaps in broadband spatial data and not assume the measure means even distribution across the spatial unit of analysis. In this spirit, we unpack who specifically within a spatial unit (in our case, a given county) does not have adequate broadband access, and in turn, is most negatively impacted by stay-at-home mandates implemented during the COVID pandemic. This is done by subsetting to specific populations known to have lower access levels or industries known to need broadband access more to work productively. If these gaps are consequential, we assert that unemployment will be higher for those counties where lack of access is indeed concentrated to particular groups or industries, hence the formulation of our aforementioned hypotheses.

### Sensor sharpening and coarsening

**Figure 3:**
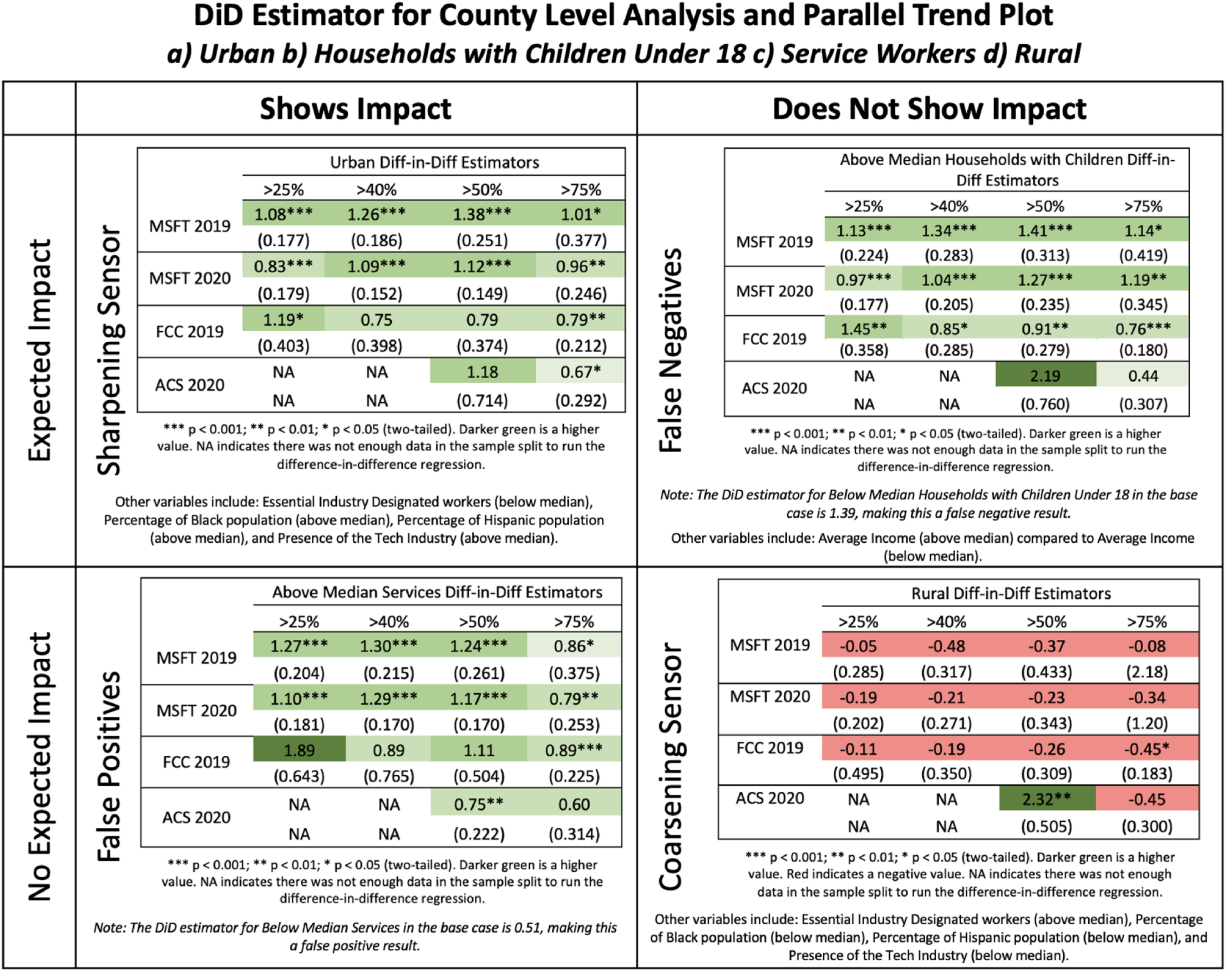
This 2 × 2 matrix typology shows where broadband as a social sensor is appropriately sharpened and coarsened, as well as prone to error (either false positives or false negatives). Robust standard errors, clustered at the state level, are included below each estimate with statistical significance indicated by the stars based off of a two-tailed test. The above figure includes regression results for an example of each scenario, but one should note here that this is just a representative sample of each case, hence why we document all other variables that fit into each cell in a line below these representative samples. The full regressions that underpin these results are in SI Appendix I.

To reiterate our hypotheses, broadband should sharpen as a social sensor for groups whose lack of broadband access makes them less resilient to the COVID-19 stay-at-home mandates, while broadband as a social sensor should coarsen for groups and locations where such access should be inconsequential in their ability to manage the pandemic. Several groupings lend support to our hypotheses. While we report the summary of our results in Fig. 3 and Table 2, the full regression models are all available in the SI Appendix I.

**Table 2:** The difference-in-difference regression estimators for each of the mechanism explorations are included in this table, along with the expected result, and a description of what this means for use as a social sensor.

Sensor target	Theorized behavior as a social sensor	DiD estimator for MSFT 2020 Data (Robust Standard Error)	Actual behavior as a social-sensor	DiD estimator for PL 2020 Data (Robust Standard Error)	Actual behavior as a social-sensor
Full dataset		1.34*** (0.16)		1.10*** (0.25)	Signal aligns with broadband
Urban	Higher unemployment rates as dependent on connectivity for local economy	1.12*** (0.15)	Sharpens sensor (supported)	0.91** (0.29)	Sharpening consistent but insignificant
Rural	Lower unemployment rates as local economy less dependent on connectivity	−0.23 (0.34)	Coarsens sensor (supported)	0.67* (0.27)	Coarsening consistent but insignificant
Above median essential industry workers	Lower unemployment rates as these jobs continued on-site during the pandemic	1.04*** (0.18)	Coarsens sensor (supported)	1.06* (0.40)	Coarsening consistent but insignificant
Below median essential industry workers	Higher unemployment rates as jobs had to continue from home so need connectivity	1.55*** (0.24)	Sharpens sensor (supported)	1.26** (0.31)	Sharpening consistent but insignificant
Above median income	Lower unemployment rates as more able to afford an improved internet plan	1.55*** (0.22)	False negative (coarsening unsupported)	1.29*** (0.29)	Coarsening unsupported but insignificant
Below median income	Higher unemployment rates as less able to afford an improved internet plan	1.03*** (0.16)	False negative (sharpening unsupported)	0.90** (0.30)	Sharpening unsupported but insignificant
Above median percentage Black	Higher unemployment rates as populous known to have less broadband access	1.42*** (0.20)	Sharpens sensor (supported)	1.13** (0.31)	Sharpening consistent but insignificant
Below median percentage Black	Lower unemployment rates as populous known to have more broadband access	0.95*** (0.24)	Coarsens sensor (supported)	0.99** (0.31)	Coarsening consistent but insignificant
Above median percentage Hispanic	Higher unemployment rates as populous known to have less broadband access	1.62*** (0.21)	Sharpens sensor (supported)	0.91** (0.29)	Sharpening inconsistent but insignificant
Below median percentage Hispanic	Lower unemployment rates as populous known to have more broadband access	0.69** (0.22)	Coarsens sensor (supported)	1.16*** (0.28)	Coarsening inconsistent but insignificant
Above median WFH	Lower unemployment rates as these jobs were prepared to WFH already	0.96*** (0.22)	Coarsens sensor (supported)	0.67** (0.27)	Coarsens sensor (Supported)
Below median WFH	Higher unemployment rates as more jobs had to pivot to WFH	1.29*** (0.20)	Sharpens sensor (supported)	1.32*** (0.31)	Sharpens sensor (Supported)
Above median percentage of tech industry	Higher unemployment rates as high tech-based industries depend on connectivity	1.01*** (0.16)	Sharpens sensor (supported)	0.80* (0.31)	Sharpening consistent but insignificant
Below median percentage of tech industry	Lower unemployment rates as low tech-based industries depend on connectivity	0.45 (0.22)	Coarsens sensor (supported)	0.76** (0.27)	Coarsening consistent but insignificant
Above median number of service workers	No difference as service work arguably less dependent on broadband	1.17*** (0.17)	False positive (coarsening unsupported)	0.94* (0.33)	Coarsening unsupported but insignificant
Below median number of service workers	No difference as service work arguably less dependent on broadband	0.51* (0.24)	False positive (sharpening unsupported)	0.76** (0.26)	Sharpening unsupported but insignificant
Above median number of single parents	Higher unemployment rates as single parents need to stay with children	1.39*** (0.16)	Sharpening consistent but insignificant	0.97** (0.33)	Sharpening inconsistent but insignificant
Below median number of single parents	Lower unemployment rates as multiple parents allow greater flexibility	1.26*** (0.24)	Coarsening consistent but insignificant	1.22*** (0.28)	Coarsening inconsistent but insignificant
Above median number of households with children	Higher unemployment rates as need to stay at home with kids	1.27*** (0.24)	False negative (sharpening unsupported)	1.04*** (0.26)	Sharpening unsupported but insignificant
Below median number of households with children	Lower unemployment rates as need to stay at home with kids	1.39*** (0.17)	False negative (coarsening unsupported)	1.15*** (0.28)	Coarsening unsupported but insignificant

Here MSFT 2020 indicates setting the treated and control counties based off of the indicated level of broadband penetration according to the Microsoft 2020 dataset. Similarly, PL indicates setting the treated and control counites based off of access to public libraries in the county. By sharpening, we mean an increase that is relatively greater for that sub-group, and by coarsening, we mean an insignificant effect or an effect that is relatively smaller for that sub-group. For instance, above median percentage black counties have a relatively larger increase than below median percentage black counties, so we argue the former sharpens the sensor, while the latter coarsens it. False negative is if the effect is insignificantly different between sub-groups (i.e., non-overlapping standard errors when comparing sub-groups above and below median threshold) or acts in the opposite direction than expected. False positive is if there is an identified effect that was unexpected. Robust standard errors, clustered at the state level, are included below each estimate with statistical significance indicated by the stars based off of a two-tailed test. Full regression models and actual p-values for all coefficients are reported in SI Appendix I.

The first subset focuses on regulatory-based mechanisms resulting from the stay-at-home orders which mandated that some groups work in-person based on their role in the local economy. In this scenario, we expect to see a greater impact on unemployment in those counties that have below median numbers of essential industry designated (EID) workers, because these workers were more reliant on having broadband access to both comply with stay-at-home mandates and to maintain their employment. For counties with above the median EID workers, when all else is equal, the base case finds that treated counties experience an increase in unemployment rate of 1.04% compared to control counties after the onset of COVID-19. For counties below the median number of EID workers, when all else is equal, the base case finds that the unemployment rate difference is 1.55% for treated counties when compared to control counties after the onset of COVID-19. We see in this gap that the sensor behaves as we expect; lower levels of essential workers sharpen the sensor and identify a larger gap in unemployment, while higher levels serve to coarsen the sensor.

To further solidify this mechanism, we also look at occupations that, by the nature of the tasks required for the work, are more amenable to the work-from-home mandates^51^. Here we expect that for counties that have on average higher numbers of people that are able to work from home prior to the pandemic, the impact of the forced work-from-home mandates during the COVID pandemic would have less of an impact as these occupations already allowed them to pivot to working from home (WFH) more easily. We find this to be the case. For counties with above the median numbers of people employed in industries that could easily work from home, when all else is equal, the base case finds that treated counties experience an increase in unemployment rate of 0.96% compared to control counties after the onset of COVID-19. For counties below the median numbers of people employed in industries that could easily work from home, the base case finds that the unemployment rate difference is 1.29% for treated counties when compared to control counties after the onset of COVID-19. Here we see the effect that people working in occupations and industries better suited to working online experienced lower rates of unemployment as a result of the work-from-home mandates.

The second subset focuses on marginalization. Certain groups, specifically Black and Hispanic populations, are known to have less access to broadband^52^ and we therefore expect them to be asymmetrically impacted when resilience depends upon broadband access during the stay-at-home mandates. Given this premise, we expect to see counties with higher median percentages of Black and Hispanic populations to also have higher rates of unemployment. For counties with above median percentage of Hispanic and Black populations, the base cases respectively find an increase of 1.62% and 1.42% in the unemployment rate of treated counties over control counties after the onset of COVID-19. For those counties below the median, when all else equal, the base cases respectively find that treated counties experience a lesser unemployment rate increase of 0.69% and 0.95% over the control counties after the onset of COVID-19. The difference for counties which have higher percentages of Hispanic and Black populations suggest that marginalized groups are indeed more detrimentally impacted by COVID-mandated stay-at-home orders, likely due to their systematically lower broadband access; higher levels of Hispanic and Black populations sharpen the sensor and lower levels coarsen it.

The third group focuses on industrial composition. We first look at industrial composition as demarcated by geography and how that should impact the importance of broadband access during COVID-19. Rural and urban areas are fundamentally different in their demand and supply for broadband services. The industries which drive the economic engines in rural areas (e.g., agriculture) are less dependent on broadband access. Therefore, we expect to see less impact on unemployment in rural areas when using broadband access to demarcate treatment and control groups. For urban counties and mixed urban/rural counties, when all else is equal, the base case finds an increase of 1.12% in the unemployment rate for treated counties over control counties after the onset of COVID-19. For solely rural counties, when all else is equal, the base case finds a statistically insignificant impact on unemployment after the onset of COVID-19. These findings suggest that the use of broadband as a social sensor is sharpened in urban areas, where the primary economic motors are more influenced by broadband access, and the sensor is coarsened in rural areas whose local economies are less dependent on broadband.

In addition to exploring the broad economic sectors associated with urban and rural areas, we also explore how work gets done in specific sectors can also impact social sensor sharpening or coarsening. For instance, the computational and analytical work in technology sectors likely necessitate greater reliance on broadband, so we would expect to see those counties with higher proportions of individuals employed in these sectors impacted more than individuals employed in other sectors. For counties with above median number of tech workers, when all else is held equal, the base case finds that treated counties experience an increase of 1.01% in unemployment over control counties after the onset of COVID-19. For counties below the median, when all else is equal, the base case increase in unemployment rate is insignificant for treated counties over control counties after the onset of COVID-19. This aligns with our expectations; given broadband is crucial for tech industry work, high-tech employment levels sharpen the sensor, while low-tech employment levels coarsen the sensor.

### False negatives and positives

So far, broadband operates effectively as a social sensor when what it measures (i.e., broadband access) is predominantly driving unemployment impacts for the social group of concern. However, as we know from engineering, sensors can start experiencing error when what it measures is conflated with other signals^53^. What that means is while we find alignment with our hypotheses for the subsets we discuss above, broadband will not always coarsen and sharpen as a social sensor in anticipated ways if subsets capture multiple conflating signals beyond broadband access. In particular, we seek to characterize both type I errors (false positives) and type II errors (false negatives).

False positives are those subgroupings that we expected not to affect our social sensor but demonstrate an effect. For example, we anticipate no effect on employment for service workers because their reasons for unemployment are due to COVID-induced business closures and arguably not access to broadband (i.e., you cannot necessarily deliver food, laundry, or run concierge services purely online). However, we find that for counties with above median levels of service workers, when all else is equal, the base case finds an increase in unemployment rate of 1.17% for treated counties over control counties after the onset of COVID-19. For those counties below the median, when all is equal, the base case finds an unemployment rate increase of 0.51% for treated over control counties after the onset of COVID-19. While this could mean such services are increasingly moving online^54^, this may also be due to confounding aspects associated with service metrics. For instance, the service sector is strongly associated and co-located with high-tech sectors (r = 0.47), which suggests those who work in high-tech also increasingly use such services. This suggests collinearity between industry variables that is spuriously picked up by broadband. Therefore in this case, the conflating signal that is leading to error is arguably the spatial and sectoral linkages between service sectors that are less broadband-dependent with high-tech sectors that are more broadband dependent.

False negatives are those subgroupings that we expected to sharpen (or coarsen) our social sensor but prove inconclusive. For example, we would expect that counties which have higher average income would experience a less severe impact from stay-at-home orders due to the capability of being able to purchase improved broadband speeds. However, we find that in counties with above median average incomes, when all else is equal, treated counties experience an increase in unemployment of 1.55% over control counties after the onset of COVID-19. Conversely for below median counties, when all else is equal, treated counties experience an increase in unemployment of 1.03% over control counties after the onset of COVID-19. This again is likely due to the fact that there are confounding aspects associated with income metrics such as education considerations (population with bachelor’s degree or higher—r = 0.49). In this case, the conflating factor is other proximate and interlinked sociodemographic characteristics that one must carefully tune and calibrate upon deployment. For instance, income may potentially be conflating sociodemographic factors that drive lack of broadband access (i.e., income) with those that reflect greater capabilities and skills to put it to good use (i.e., education).

We see similar false negatives with households with children. Here, we posit due to the need for children to engage in virtual school due to stay-at-home mandates, these households would likely require the parent to stay at home to tend to their children during these times, risking their employment. However, here too we do not find this expected impact as there is little difference in the unemployment rate increases in the base cases (households with children: 1.27% unemployment rate increase for above median vs. 1.39% for below median). We do see this is more consistently the case for counties with above vs. below median levels of single parent households, but the differences between the groups overlap, suggesting they are less significant (see Table 2). Again in this case, the conflating factor may be linkages to other sociodemographic characteristics. For example and as suggested above, perhaps these false negatives are conflating family composition with income, such that a larger family may be able to afford child support with children than smaller families. This suggests one interlinkage between income and family composition that is difficult to disentangle.

**Figure 4:**
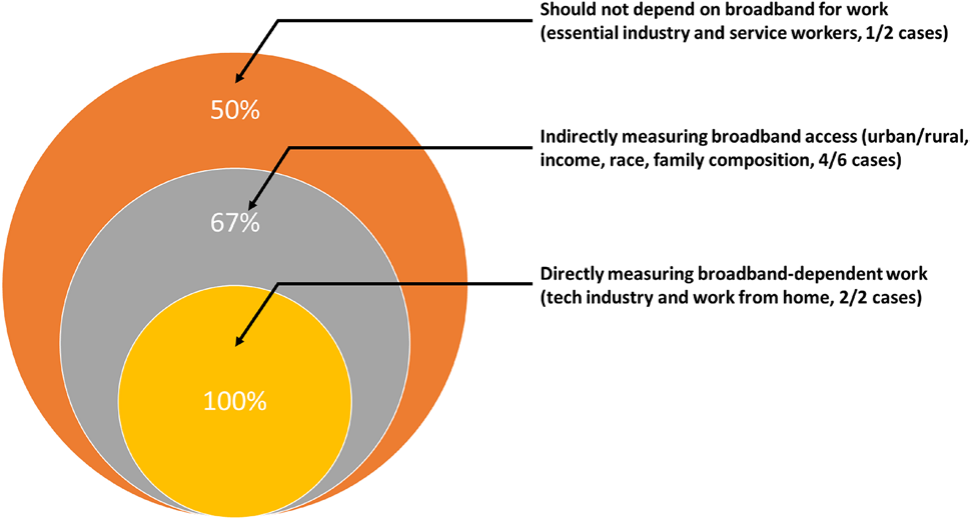
This figure presents a graphical summary of case consistency. As expected per Boundary Condition 1, the social sensor weakens as dimension is increasingly distant from broadband-dependent work.

Overall then, social sensors are designed to measure one signal (in our case, access to high-speed internet from broadband). Inevitably then, when that signal no longer dominates and/or has other conflating and competing signals, errors are likely to result. Figure 3 presents a 2 × 2 typology that summarizes the results from our analysis and presents an indicative heatmap that reflects each cell of the typology. Table 2 reports all DiD coefficient estimates from these regressions and provides a synopsis of what we expected versus what we actually find in our analysis. In line with Boundary Condition 1, Fig. 4 shows how the strongest signals are closest to those dimensions that most directly measure broadband-based dependent work.

### Creating an array of social sensors: additional built environment sensors—a proof-of-concept

How then can we help mitigate the errors from a social sensor (in this case broadband)? Perhaps as in engineering, sensors are more effective when they are *arrayed*, whereby multiple sensors that read different signals are linked together to mitigate weakeness in any one sensor. This reflects practices in the field of engineering to understand how sensors are developed and used to measure various parameters. One means for doing this is to have a set of redundant measures to ensure accurate readings. We see this in the field of atmospheric science. When measuring the temperature of clouds, which is a critical predictor of storm dynamics and cloud formation, both radiosondes and drone profiling are used to get the most accurate measure possible to include in the models^55^. We argue that this holds true for the use of infrastructure as a social sensor. We can perhaps strengthen the sensor by incorporating other, additional sensor measures highlighting the relationship between broadband internet and unemployment. Secondly, while there are some applications where a single sensor is enough to measure a given parameter, this is not always the case for more complex parameters. For example, one can get an accurate reading of temperature by only using a thermometer. However, in order to track an object’s movement, a complementary array of sensors may be required, including but not limited to an accelerometer and optical tracking capabilities^56^. In order to understand the complex dynamics of economic metrics during times of crises, a full array of infrastructure sensors may more accurately pinpoint counties which are detrimentally impacted.

We start by assessing how a redundant measure of broadband access, WiFi enabled public libraries, may work to strengthen the results of our study. Public libraries serve as a “first choice, first refuge, and last resort in a range of emergency and e–government circumstances”^57^, and many people who did not have access to broadband in their homes during COVID made use of their local public libraries to help fill the broadband gap^58^. For this redundant public (as opposed to private household) measure check, we created a parallel metric to our broadband metric which assessed what percent of a county’s population falls within the “legal service area” of a public library, defined as the population that lives within the boundaries of the geographic area the library was established to serve. For an additional sensor to be a useful component in an array, there should be some orthogonality, which suggests that the additional sensor is providing information that is not being captured in the existing sensors used. In this case, there is some correlation between public libraries and broadband access, but not perfect correlation, which suggests libraries are providing additional information not captured in our core broadband sensor. We find the correlation between the metrics, when in binary form of treated vs control, to be 0.2. Using this metric, we classify counties with below 50% of their population in the legal service area of a public library (akin to our below 50% penetration of broadband at a county level) as our control group and we classify counties with above 50% of their population in the legal service area of a public library as the treatment group (akin to our above 50% penetration for broadband). We separately run the same model presented above and find that our results directionally hold with the results using broadband access as the sensor, but that the signal of the results on average across most of the cases are smaller and less significant on average. We argue this suggests that public access to broadband helped reduce gaps seen in private access to broadband. The results from this analysis are included in Table 2. Triangulating across multiple sensor signals also not just reduces the instance of false positive and false negatives, but also isolates which subset provides the strongest signal for which to inform more targeted policy support. In this case, coupling libraries and broadband renders insignificant much of the false positives and negatives found in broadband alone, and helps identify occupations most equipped to work from home as the subset with the strongest signal for which to target policy support as both broadband and libraries detect this effect. Perhaps the reason for this is that public access to broadband is more suitable for less data-intensive needs (e.g., email or accessing websites) and less so for more data-intensive needs (e.g., Zoom calls and computational analyses) for which many occupations that were yet equipped for WFH may have necessitated.

To further explore the creation of sensor arrays to detect gaps, we also selected two forms of physical infrastructure which serve to complement the upstream and downstream rollout of broadband—bridges per county and new building permits per state (selected based on data availability). We selected these because they capture different dimensions as to how the built environment can influence broadband through rollout and point of access. Building networks are likely where broadband is deployed more downstream and therefore where it is accessed. Building networks have a correlation with the broadband access metric of 0.15. Bridge networks impact urban connectivity and therefore may influence where broadband is rolled out more upstream. Bridge networks have a correlation with broadband of 0.25^59^. As with libraries, building and bridge networks are adding novel information to our core broadband sensor. As a result of this, we would expect that by integrating both sets of physical infrastructure into our broadband models, this would help sharpen the (broadband) sensor. Perhaps also areas with physical connectivity enhance the expectation of digital connectivity more than areas without such connectivity.

**Figure 5:**
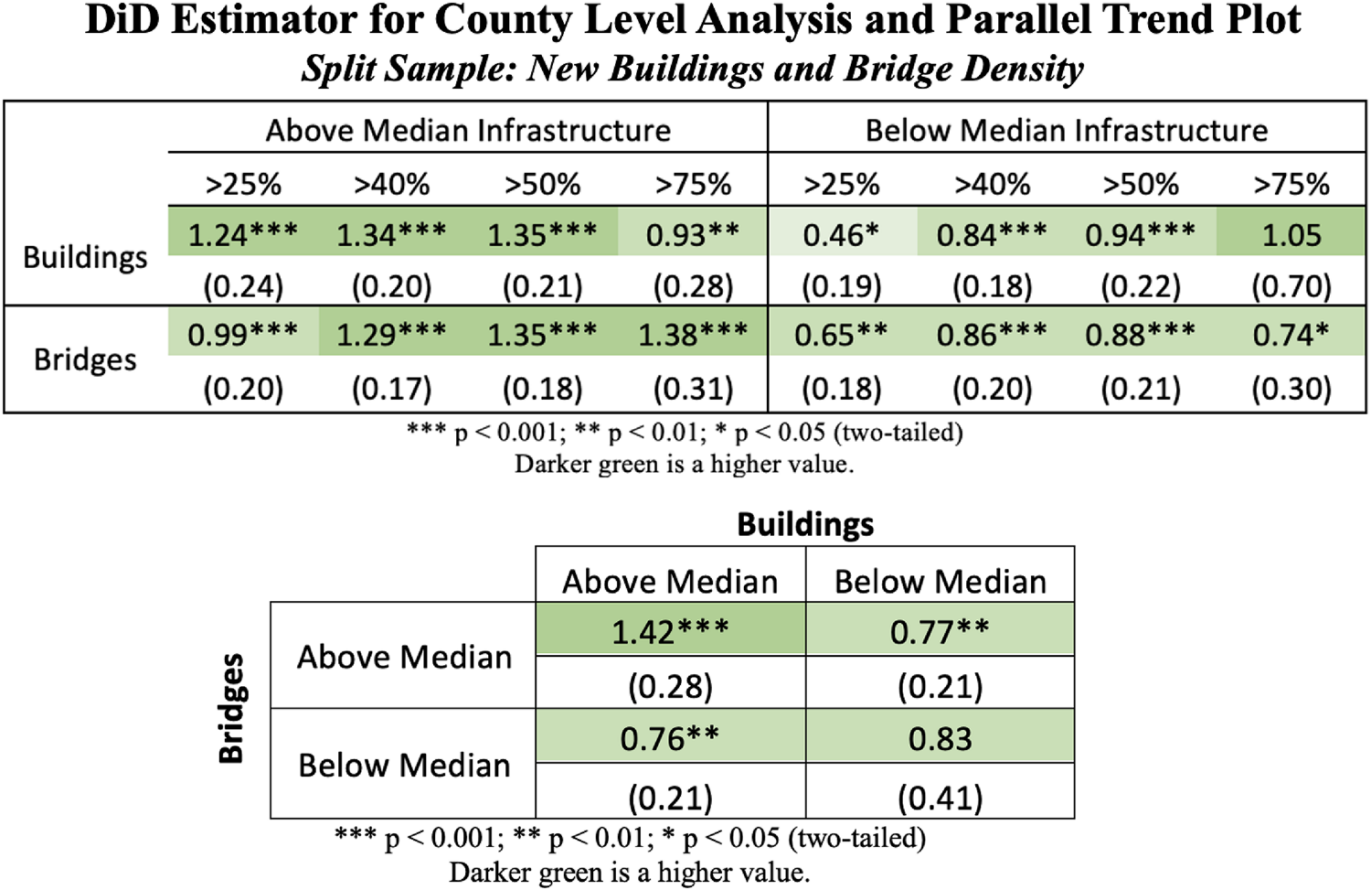
Top: difference-in-difference estimators for the full dataset. Robust standard errors, clustered at the state level, are included below each estimate with statistical significance indicated by the stars based off of a two-tailed test. Under the base case using MSFT 2020 data and a 50% penetration rate as the treatment and control groups, in the subset of counties which have above the median number of new building permits per county, all else equal and after the shock of the stay-at-home mandates, experience an increase in unemployment of 1.35%. This is compared to an increase in unemployment of 0.94% when the subset has a below median number of new buildings. We see that in the subset with above median number of bridges, the increase in unemployment for the base case is 1.35%, compared to the below median number of bridge subset with 0.88% increase. Bottom: based off the findings in the top figure, we investigate further the compounding effect of infrastructure and find that in counties with above median density of bridges and new houses, the impact on unemployment is further exacerbated, suggesting that infrastructure services may be integrated with the provision of broadband. The parallel trends between the control (below adequate access to broadband at a county level) and treatment (above adequate access to broadband on average at a county level) for both number of buildings (upper row) and number of bridges (bottom row) can be found in Appendix G. Parallel trends hold prior to the shock of the COVID-19 pandemic. The full regressions that underpin these results are in SI Appendix I.

As shown in Fig. 5, for counties with above median number of new building permits per state, when all else is equal, the base case finds treated counties experience an increase in unemployment rate of 1.35% over control counties after the onset of the COVID-19 stay-at-home mandates. This is compared to an increase in unemployment rate of 0.94% for counties below the median. For counties with above median number of bridges, the increase in unemployment for the base case is 1.35%, compared to the below median subset with a 0.88% increase, holding all else qual after the onset of COVID-19. Given the built environment has similar upstream and downstream impacts, we then further assessed whether these are complementary or substitutive. These effects seem to be complementary as the subset of areas where both bridges and buildings are above the median generate the largest delta (1.42%) between the treated and control counties in the base case. One may presume that perhaps these impacts are due to multicollinearity and that bridges and buildings are simply collocated with each other. This appears to not be the case as the correlation between our binary measures of bridges and buildings is near to zero (r = −0.02).

Overall, this suggests linking sensors into “arrays” strengthens the signal, reduces errors, mitigates detection gaps, and helps identify the most prominent subsets for targeting. In this case, public libraries reduce the false positives and negatives from broadband alone, help prioritize which subset yields most promising gaps for targeting (i.e., occupations yet equipped for WFH), and identifies more precisely where broadband signals weaken. Moreover in incorporating additional built environment features, we can detect influences on these broadband gaps based on gaps in rollout (bridges) or gaps in points of access (buildings). Clearly, we can conceive many other different sensors for such an array, so we see this as demonstrating a proof-of-concept for future work to explore more systematically other sensor arrays and outcomes, even beyond those centrally focused on broadband and unemployment. Overall in line with Boundary Condition 2, using multiple sensors in an array improves targeting to the most key variables (i.e., occupations not yet equipped for WFH) and to the most key locations (i.e., those with both upstream rollout and downstream access infrastructure).

## Discussion

Our study explores the possibility of using gaps in infrastructure as a “social sensor” to help us more introspectively target policy and investment towards areas whose resilience is especially fragile to disruptions and other exogenous events. We develop an “infrastructure-as-sensor” approach by analyzing the impacts that broadband access has on unemployment in the United States during the stay-at-home mandates implemented during the COVID-19 pandemic. The result is a 2 × 2 typology that explains what factors sharpen the sensor, coarsen it, and render it prone to error (both false positives and negatives). We further supplement this work by demonstrating how errors and gaps in the sensor can be addressed through creating a sensor “array” that couples broadband with other infrastructural measurements of access (i.e., libraries) as well as with other factors that may influence upstream rollout (i.e., bridges), or downstream points of access (i.e., buildings).

Besides the main objective of providing support more quickly to communities susceptible to disruption, we see several contributions resulting from this work. First, the infrastructure-as-sensor approach can potentially better address structural factors that hinder promising human-as-sensor interventions. Prior work from a human-as-sensors approach show how enhanced granularity can be achieved, but focuses less on how to best target the deployment of such approaches in lieu of their large computational costs^27,28^. An infrastructure-as-sensor approach can help identify the most promising subsets for targeting, and then focusing a human-as-sensors approach on these subsets can help maximize the approach’s benefits while mitigating its costs. Simply put, an infrastructure-as-sensor approach can help detect the most promising areas for which a human-as-sensor approach can bring greater granularity and value. More specifically, prior human-based social sensing approaches focus more on different individual interpretations of the surrounding social world^60^. Our infrastructure-based social sensing approach pioneered here focuses more on how infrastructure influences what comes to be available in one’s social world well before interpretations are made.

Second, we demonstrate the value of not just using one sensor, but a sensor array. Prior work on social sensors focuses primarily on the value of using one specific sensor but very little work looks at the interdependencies and interplay across sensors. Sensors for engineering applications are often designed to measure one signal^61^. However, sensors in social applications are often more complex whereby multiple social signals are interdependent and so measuring one may not always be sufficient to adequately characterize key behaviors and activities. Moreover, intertwined social signals may muddy the ability to measure any one signal that a sensor is intending to measure. As a practical example, consider the ambitions around smart city initiatives. The vision for smart cities is to integrate several data layers in real-time so cities can “self-diagnose” problems^62^. This requires combining different infrastructure data, each equipped to best measure different social activity. Moreover, each of these data have differing assumptions and biases as to what data most matters that could perpetuate when such approaches are scaled to a city level. One particular issue is that the algorithms used in such approaches are likely trained on data that are not necessarily representative^24^. In taking our infrastructure-as-sensor approach and, more importantly, using multiple sensors in an array, we see our work helping to address these issues in several ways. For instance, we find integrating social sensors in an array helps better sharpen and detect gaps and skews present in any one sensor alone. Perhaps then, these arrays could be used to penalize overfit in smart city planning models to observed priors when such gaps and skews are found increasingly present. This could be done through weighting residuals by the number of social sensors in the array present at a given location. We also find integrating sensors in an array helps further triangulate which subsets are more promising for more granular analysis (such as with a humans-as-sensor approach previously discussed). This can better ensure models are less reliant on proxies of access (such as spatial measures) that obscure and mask important gaps and skews that have significant equity implications.

Third, we highlight that our approach provides a novel method for using publicly available data to assess existing community vulnerabilities to infrastructure service gaps, which contrasts with existing approaches that rely on extensive computing power and large-scale proprietary data. Currently, much of the work in this space is focused either on how to use human online presence and commentary to gain insight into physical world events^30^ or how to use data collected about humans to understand how they interact with their physical environment, for example through the use of smart-wearables^31^ or by assessing point clouds which outline the coarse body shape of people in order to understand actions ^32^. All these approaches require the collection of large-scale sets of private data. Our approach uses infrastructure data that is publicly available and so further increases data access ease and speed. Given its basis is public data, such an approach is also arguably more scalable in ways that are more sensitive to privacy concerns.

**Figure 6:**
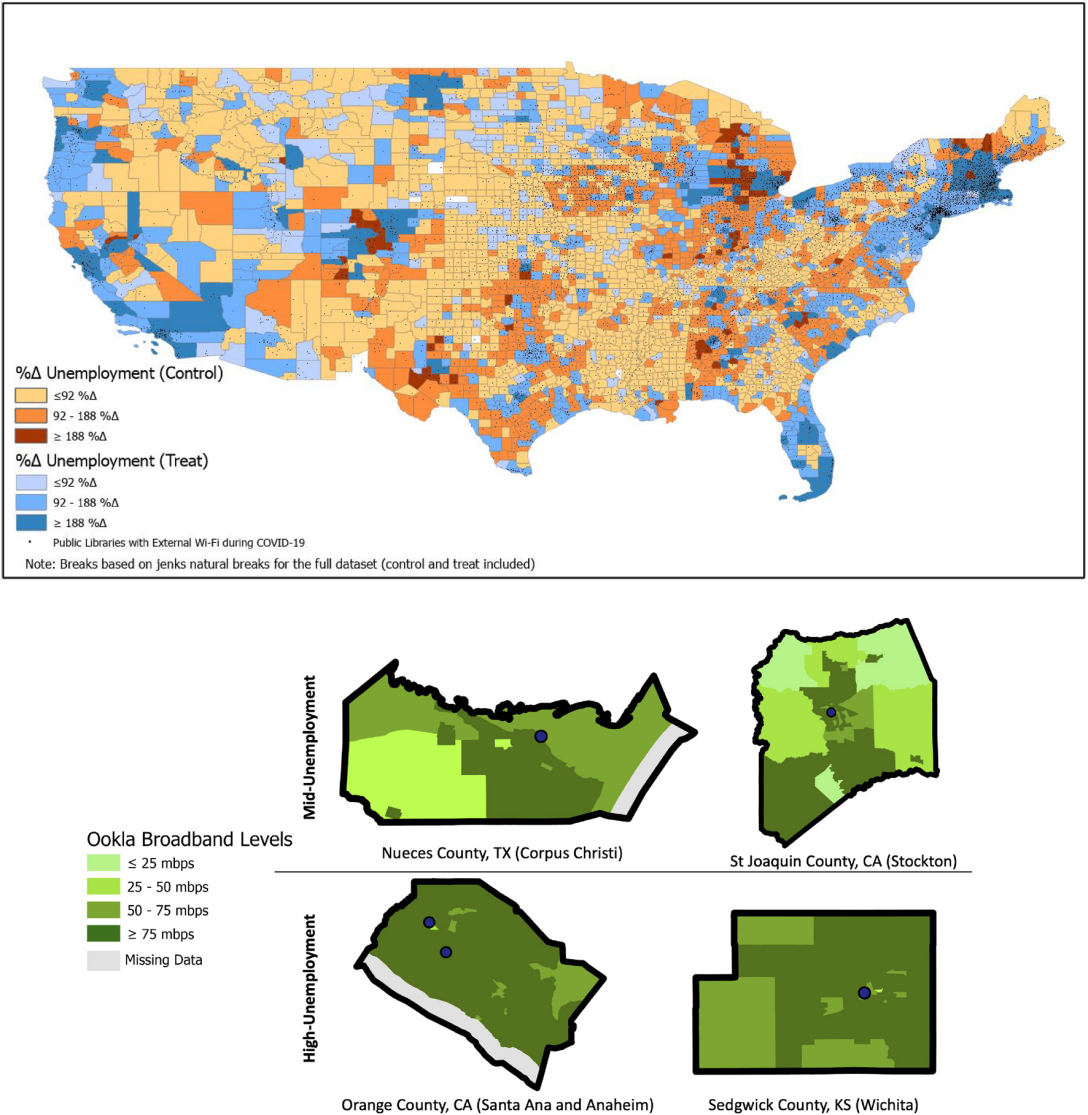
Top: Counties in the United States, presented in terciles of percent change in unemployment for the 6 months leading up to the COVID shock and for the 6 months following the COVID shock. Counties are colored according to being in the treatment (shades of orange = more than 50% of the population in the county has access to 25 mbps download speed, according to MSFT) or control (shades of blue = less than 50% of the population in the county has access to 25 mbps download speed, according to MSFT) groups. The darker the color, the higher the change in unemployment was from pre-COVID to post-COVID. Bottom: A highlight of treated counties which showcase how access is more clustered for treated counties with higher average unemployment. We see that in Orange and Sedgwick counties, the lack of broadband access is heavily clustered whereas in Nueces and St Joaquin County, the broadband access is more distributed. Maps created by Ritsch, N. Percent change in unemployment, by control and treated counties, featuring highlighted hot-spot counties [map]. Using: ArcGIS Pro [GIS software]. Version 2.6.

Moreover, we use urban studies and sociological thinking to guide sensor choices. Prior literature provides more guidance on evaluation of infrastructure deployment^5,21,25^, but less guidance on how to inform sensor choices (i.e., what a given infrastructure of interest can or cannot best detect). With such methodological advancements, government agencies can use the insight from such analyses to assess more quickly who in the future may most immediately need broadband support before the next pandemic occurs. To explain how that could occur using this work, we provide a demonstration case (Fig. 6) for which we highlight two sets of representative counties. We selected counties housed in similar moderately sized cities throughout the United States and focused only on treated counties in order to understand where gaps of in-access occur. We see that in both Orange County, CA and Sedgwick County, KS, the two treated counties which have a higher percent change in unemployment, there is more concentrated in-access of broadband at the census-tract level (i.e., fewer areas of lighter green shading). On the other hand, Nueces County, TX and St. Joaquin County, CA, which had increases in unemployment but not as much as Orange and Sedgwick Counties, have more distributed areas of in-access (i.e., larger areas of lighter green shading), making lack of broadband access in these areas arguably more observable to employers. This tracks with our key findings and mechanism around a halo effect (i.e., employees more likely to wrongly assume all have broadband to continue to work as lack of access becomes more concentrated). In response to this, our sensor would prescribe that federal support, such as vouchers or other forms of emergency support, be concentrated in these few low-access census tracts in high access counties to achieve the greatest unemployment impacts. Furthermore, once such isolated tracts have been identified, we would argue that the practice of using humans-as-sensors approaches can be more effectively implemented in order to understand the micro-level impacts of such access. Our argument is that the humans-as-sensor approach has shown the possibility and benefits of granularity but there is no systematic analysis of where such granular techniques can realize the greatest benefits. Our infrastructure-as-sensors approach helps provide a framework for where these granular, though more computationally and even labor-intensive approaches, are most suitable for enhancing equity. The findings from this work could be directly incorporated into decision response frameworks as a less computational-intense indicator for what areas might have underlying vulnerabilities, especially in instances where response speed is critical as is the case with a pandemic.

Finally, this approach entirely rethinks the fundamental incorporation of infrastructure in social and economic based studies. We can no longer consider infrastructure as an exogenous input that is equally accessible by all (as much of the existing analysis considers). Rather infrastructure is an endogenous input that is asymmetrically distributed and whose interdependencies matter. As such, we cannot take infrastructure for-granted as an equally accessible input that all can use to create value.

Overall, we hope this study motivates future work to advance the agenda that we propose around using infrastructure as a social sensor to better detect inequity and bias. If we take seriously the numerous retrospective studies that note infrastructure is more a measure of bias than equity, then we can more introspectively use such networks to detect harm in the moment, rather than ponder “what if” as structural inequalities continue to ossify.

## Methods

**Figure 7:**
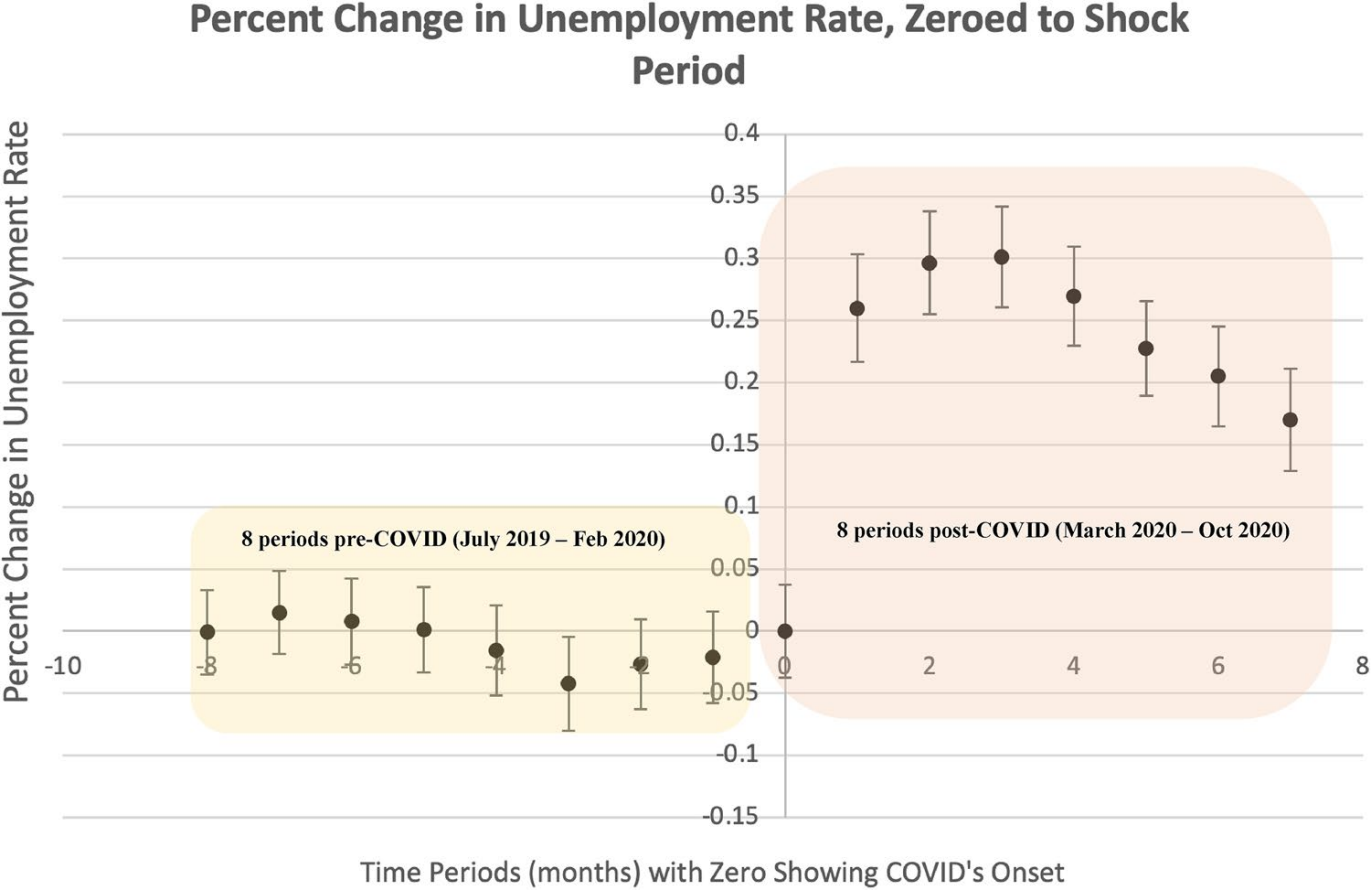
The graph presented in the top of this figure shows an event study plot that looks at the eight months prior to COVID-19 and the eight months post COVID-19. Prior to COVID, unemployment rate differences between the treated and control units are largely not different from zero. However, after the COVID pandemic we see differences between the treated and control units are increasingly and significantly different from zero.

The primary methodological approach used here is difference-in-differences (DiD) regression modeling. Several critical assumptions underpin DiD models. The first assumption states that the treatment and control groups behave similarly pre-shock and that there is not an unobserved factor sorting the groups which would violate strict exogeneity, an extension of Gauss-Markov’s zero-conditional mean of the error assumption^63^. A key way to assess this assumption is to analyze if the trends between the control and treatment groups track similarly prior to the onset of the treatment, commonly called the parallel trend assumption^64^. In this case, this means treatment and control group trends, as defined by broadband penetration levels, should track similarly prior to the onset of the COVID-19 stay-at-home mandate shock. To verify this, we present a parallel trends plot, which provides a visual check on the zero condition mean of the errors assumption required for the DiD analysis, for the main analysis in Fig. 1 and present the rest of the parallel trends plots throughout the SI to further demonstrate that these trend assumptions are qualitatively supported. In addition to these plots, we also create an event-study plot which shows how the difference in percent change between each group, when zeroed to the shock year, is close to zero in the pre-trend years and grows after the shock occurs. This can be found in Fig. 7. Furthermore, we find that the basic assumptions required to run a DiD regression model, such as the data is randomly sampled from the population and that the variables are normally distributed, are upheld and these checks can be found in SI Appendix F. We also conduct a generalized synthetic controls approach and separately a Bayesian causal Inference approach to further assess and verify whether the parallel trends assumption holds true (see Appendix H for more details). We also need to make assumptions about broadband treatment consistency over time. To ensure this penetration holds, we vary treatment based on different broadband penetration rate thresholds and even on different measures of broadband that appear throughout the paper and SI. We also run a robustness check on the counties that remain in either treated or control categories over the years of 2019 and 2020, the results of which are consistent and robust (found in SI Appendix G).

### Data

#### Resolution of data

This analysis is run at a county-level spatial and monthly time resolution. As of the 2020 U.S. census, there are 3143 counites^65^. We use the complete dataset where there is data available across all dependent and independent variables. We drop data for 8 counties due to lack of unemployment data (02063—Chugach Census Area(AK), 02066—Copper River Census Area (AK), 15005—Kalawao County (HI)), lack of demographic data (02195—Petersburg Census Area, (AK), 22059—La Salle Parish (LA), 35013—Dona Ana County (NM)), and lack of education data (02158—Kusilvak Census Area (AK), 46102—Oglala Lakota County (SD)). Where there is missing data across the splits, these counties are removed only for that analysis, as is represented in the observation counts presented in the regression tables in Appendix I.

#### Dependent variable: unemployment

This work focuses on unemployment rates as the dependent variable. As used in numerous prior other studies^33,35^, data from the Local Area Unemployment Statistics (LAUS) Dataset, published by Bureau of Labor Statistics (BLS), is used to construct the dependent variable. Specifically, we use the LAUS’ unemployment rate data, which is measured as the ratio of unemployed people to the total civilian labor force, in a percentage form^66^. We download the data directly from the BLS user interface, at a monthly and county-based resolution. We then combine this data with the finalized list of counties as defined in the 2020 census. For the main models, we use the unemployment rate as a percent. Due to potential concerns of non-normality (see SI Appendix F), we also re-run this analysis using log of unemployment rate, and the results were even stronger and more robust (see SI Appendix F).

#### Independent variable: broadband and COVID-19

The first measure of broadband we consider is the speed threshold measures. For the purposes of our work, there are three primary datasets which aim to measure speed thresholds. The Microsoft dataset counts the number of devices that have connected to the internet at broadband speed per each zip code^67^, while Ookla^68^ and M-Lab^69^ use individually conducted speed tests to assess median internet speed at a census-tract and county level respectively. MSFT data is used as the base case in this paper as it is the only dataset which measures speed thresholds for broadband quality systematically, without reliance on the user initiating the speed test (as is required by Ookla and M-Lab). However, the MSFT data only assesses devices using MSFT tools and Ookla and M-Lab are user defined. The MSFT data is pulled directly from Microsoft’s Github account for both 2019 and 2020^70^, while the Ookla and M-Lab data are sourced from the Indicators of Broadband Need Map in 2020^71^, a tool managed by the United States Department of Commerce and National Telecommunications and Information Administration. One should note that the MSFT dataset does have limitations in that the speed tests are only conducted anytime a user connects to a MSFT application, which in turn does not capture a full measure of servers being used^72^. Moreover, we choose to ground our interpretations at a 50% penetration rate because this aligns with prior work in this area which argues that in order to achieve adequate economic impact, digital infrastructure must reach at least 50% of the population^73^. When we refer to “base case” throughout the study, we are referring to this case (MSFT 2020 data with treatment being those counties as on or above 50% penetration rate and control being those counties with less than a 50% penetration rate). The results remain largely consistent across the various datasets and penetration rates and are presented for transparency.

The second measures broadband advertised availability. The most widely used dataset is published by FCC under the Form 477 which is available at census-block on a 6-month basis or county annually. Federal reporting mandates require that companies self-report their maximum advertised broadband level at every census tract. This is then assumed to be the level of access at that census tract. While this data is audited by a third party, the self-reported nature of this data is cause for concern^74^. Further, the dataset is predicated on the highest level of advertised broadband in a census tract, rather than being a measure of what speeds are *actually* measured. While this data is primarily available at the census-tract level, the FCC aggregates the data to county level on an annual basis. In order to keep our dataset comparable to other analyses run with both FCC and MSFT data, we use the measures of FCC data already included with the MSFT base data^70^. One should note that the FCC data is the only dataset which separates out mobile data. While this is not ideal for comparability across the datasets, we feel that the results are still indicative of general directionality.

The third and final measure of broadband internet assess adoption. The U.S. Census incorporates a question into the 5-year American Community Survey (ACS) which asks if a household has access to internet (which is divided out into broadband access, dial-up access and subscription access). This data does not assess the quality (primarily measured in speed) nor reliability (how often is the speed reported) of the internet connection. This data is available at census-tract level on an annual basis, although the data is not comparable over adjacent years due to ACS’s methodological data development^75^. This data was obtained from the Indicators of Broadband Need database^71^.

The average number of people with access to adequate levels of broadband, at a county level, are used to define the treatment and control groups for our DiD analysis. As we noted in the main body of the manuscript, adequate access to broadband is defined as 25 Mbps download and 3 Mbps upload. Though we use a 50% penetration rate for our base case, we vary the threshold for penetration rates (from above 25% to above 75%) to ensure greater robustness in our findings. Moreover, we further ran a robustness where we only keep those counties that are consistently in the treatment (or consistently in the control) between MSFT 2019 and MSFT 2020 data to ensure our treatment is consistent and robust in its findings (these results are in the SI Appendix G).

The COVID-19 shock was measured as the month in which a majority of the stay-at-home orders were put in place in response, which was March 2020 (1-on or after March 2020; 0-before). We run a sensitivity analysis, as is discussed in Appendix G, on shifting the shock month from March to April, and we find that the results are robust. We further ran a placebo regression where we shifted the shock month to either 6 months prior (September 2019) or 6 months after the start of COVID (August 2020), and found the results no longer hold. Thus, the results seems due to COVID and not some other secular trends.

#### Moderating variables

To assess sensor sharpening and coarsening as well as where broadband as a social sensor is prone to error, we use several moderating variables to subset the data. Regarding regulatory factors, we measured the number of essential industrial workers at the state level using the federal government’s definition of Essential Workers^76^.

Regarding marginalization factors, we used data from the 2019 ACS and 2020 Decadal Census which we pulled via the Census API interface in R, tidycensus^77^. We calculate the percent of Black/African American population by dividing the total population who is Black (B02001_003E in ACS 2019 and P1_004N in Decadal Census) by the total population (B01003_001E in ACS 2019 and P1_001N in Decadal Census). We calculate a similar metric for the percent of the population who is Latino/Hispanic by dividing the total population who is Latino/Hispanic (B03002_012E in ACS 2019 and P2_002Nin Decadal Census) by the total population (B01003_001E in ACS 2019 and P1_001N in Decadal Census). We pull the average household income directly from the ACS 2019 data (B19013_001E). We calculate the number of single parent households by summing the number of single fathers (B11005_006) with the number of single mothers (B11005_007), divided by total number of households (B11005_001) in ACS 2019. Similarly, we calculate the percentage of households with children under the age of 18 by dividing the number of households with children under 18 (B11003_001) by the total number of households (B11005_001). For industry factors, we consider both geographic and industry composition.

Regarding geography, the NCHS’s Urban-Rural Classification scheme for counties takes into consideration the population density at a county level and ranks the county as being solely urban, a mix of rural and urban, or only rural^78^. The analysis is run using subsets that separate out solely urban counties and mixed rural/urban counties from solely rural counties. This is done to capture the effect occurring in rural counties.

Regarding industry, we separate out the counties with above median numbers of employees working in “High Tech Fields” as defined by the National Science Foundation in their Science and Engineering Indicators^79^ and collect the data by county from the Bureau of Labor Statistics. We also measure the number of service sector workers in a county. Here, we define the service sector using NAICS Sector codes^80^ Retail Sales (44–45) and Accommodation and Food Services (72). We collect this data directly from the Bureau of Labor Statistics (BLS) Quarterly Workforce Indicators (QWI) database. We also separate out counties with above and below median numbers of employees that work in occupations which most likely can be done from home, and therefore already arguably have adequate broadband access. Here, we used the definition provided by Dingel and Neiman and focused on the industry they identified as having the highest share of jobs that can be done completely from home, Educational Services^51^. This analysis assesses the potential for conducting all work-related tasks from home, which implicitly assumes adequate broadband coverage. We collect this data again directly from BLS QWI database using NAICS Sector code 61.

Regarding additional built environment factors for creating the sensor arrays, we select three key supplemental infrastructure systems. To assess the interaction between bridges and broadband, we look at counties with above and below median number of bridges per county using data pulled directly from the National Bridge Inventory for 2020. For buildings, we look at the number of new building permits per state according to the US Census Bureau^81^. This is the only publicly available data we could find on building density. To assess wifi-enabled libraries, we use data from the Public Library Survey, available from the Institute of Museum and Library Services^82^. From the total 9245 public libraries included in the initial sample, we only kept those that had either "External Wifi Access Before COVID-19" or "External Wifi Access Added During COVID-19" and that did not have their legal service area data suppressed. This reduced the total sample to 7626. We summarized by county the unduplicated legal service area which provides an estimate of the number of people each library serves with any potential duplicate people removed. We then replicate the broadband metrics by taking the total number of people with "access" to a public library (i.e., are within the legal service area) and divide that by the total county population to get our percent library access for the county.

#### Controls

We controlled for key demographic factors obtainable through the U.S. Census and known to affect the role of broadband on employment. In particular, we control for education levels, ethnicity and population density in order to control for differences between counties, as is done in prior work^37,41^. We include education as a regressor because by increasing the broadband coverage in an area, it is likely that individuals who have previously not been able to access other forms of online education, may be able to access additional education, which would in turn result in greater likelihood of future employment. Education may also influence one’s digital literacy to use broadband connectivity for such productive purposes^83^. We capture the baseline of educational attainment as the number of people over the age of 25 with a Bachelors’ degree and the percentage change in the population over 25 which holds a Bachelors’ degree. We also include aspects of demographics as control metrics, such as the number of people who are Black and Hispanic in each county as demographics can reflect structural barriers or enablers to employment, irrespective of broadband access. These controls are included in all regressions which are run. These datasets are also used, in part, for the splits run on race. We also include population density as a way of controlling for larger cities and urban areas likely receiving “treatment” of broadband access earlier. We also included year and state fixed effects. For added robustness, we also ran models that included a wider range of controls that include more granular employment levels across several prominent sectors, COVID case loads, amongst other covariates found in prior work ^84^. These models with such additional controls led to similar and robust results (see Appendix G in the SI).

### Statistical methods

As we noted previously, we employ a DiD approach for this analysis as it allows the investigation to be structured into a quasi-experimental framework. In this approach, measurements of employment for both control and treatment groups before and after the “shock” of COVID are used as the dependent variables. While varied and assessed in the robustness check for correctness, the baseline analysis establish the control group as any county that has below the industry standard of 25 Mbps download and 3 Mbps upload speed^85^ for 50% of the population. The treatment group then include any county with above adequate levels broadband speed for 50% of the population. DiD allows us to compare the change in the treatment group after the impact of the shock (COVID in our scenario) to a comparable control group in order to understand how the treatment (in this case broadband access) impacts the dependent variables of interest. In essence, this design allows us to compare treatment group counties to highly comparable counterfactual controls to understand what impact broadband access has on unemployment.

Based off of this, the base regression model is presented in Eq. (1):

1 $${UnemploymentRate}_{i,t}={\beta }_{0}+{\beta }_{1}{\left(Broadband\right)}_{i}+{\beta }_{2}(COVI{D)}_{t}+{\beta }_{3}(Broadband\times COVI{D)}_{i,t}+{\beta }_{k}(Controls{)}_{i,t}+{\varepsilon }_{i,t}$$

*Unemployment Rate* is the average monthly unemployment rate for county *i* at month *t*. *Broadband* is a binary variable, reflecting if the county is above or below the threshold for adequate access to broadband at the given penetration rate. DiD approaches assume treatment and control groups remain constant throughout the analysis, so this should only vary by county *i* and not by month *t*. As stated earlier, this is checked and confirmed in Appendix G. *COVID* is a binary variable for when COVID began (0—July 2019 through February 2020; 1—March 2020 through December 2020). This impacts all counties, irrespective if they are in the treatment or not, so this impacts month *t* and not county *i.* The DiD indicator is the coefficient of the interaction between *Broadband *×* COVID.* The remaining terms are county-level controls, which vary over space. These variables do not vary by time due to the availability of data of the timeframe analyzed. Therefore, the pre-pandemic (2019) levels for the controls were included to ensure that the pre-pandemic levels are what drove the results and not any post-movements. *ε* is the error term. Given the policies were initiated at the state level (see SI Appendix B for complete list of state policies), the error term is clustered at the state level to reflect that policy reality. Moreover when running additional robustness checks, we ran a well-known variant of this DiD model that only includes the DiD estimator (*Broadband *×* COVID*) and fixed effects for time (month) and unit (state), known as a two-way fixed effect DiD model^86^, and find similar results. These results are available in Appendix G.

In addition to checking different scenarios for robustness, we also conduct a check on the parallel trends assumption. Figure 7 shows an event-study plot depicting the percent change between the treatment and control groups for the base case in the 8 time periods leading up to the introduction of the shock and for the 8 time periods after the introduction of the shock. We see that the trend leading up to the start of COVID show that the differences between the control and treatment group are not statistically different from zero and that following the start of COVID, we see that the percentage change in each group after COVID is statistically different from zero. This further demonstrates that the parallel trends assumption is upheld for this analysis.

### Robustness checks

In order to further perturb these results, we also conduct a range of robustness checks to ensure that the results are robust across a variety of different theoretical arguments. We summarize these 8 key checks below for added clarity and point to where the specific results can be found in the SI information:

1. Inclusion of fixed effects at the state and monthly level (SI Appendix G, Table G1)
2. Additional explanatory controls for industry employment (SI Appendix, , Table G2). This emulates work done by Isley and Low which includes controls for the percentage of the population employed in NAICS 2-digit industries^84^.
3. Controlling for COVID case load (SI Appendix, , Table G3)
4. Use of the various outlined datasets of broadband access at a county level. This check has been integrated throughout the analysis given how fundamental the critiques are across the different datasets and given the discussion around what each dataset is measuring include a range of other metrics which could create confounding effects.
5. Parametrizing what percentage of the population has access to 25 Mbps download and 3 Mbps of upload speed. This check is also incorporated throughout the paper.
6. Assessing consistency of control and treated groups over time (SI Appendix, Table G4)
7. Perturbing when COVID-19 occurred. This includes adjusting the implementation of the shock from March to April of 2020 to show the results are directionally and statistically consistent (SI Appendix, Table G5). This check also included running falsification tests to demonstrate how shifting the shock to a different month (either September 2019 or August 2020) greatly reduces the effects to confirm the findings are due to COVID-19 rather than some other secular trend (SI Appendix, Table G6 and G7)
8. Running the regressions using Ookla and Mlab data (SI Appendix G8).

We find that the results of all these robustness checks are directionally consistent and statistically robust to our base case findings.

### Supplementary analysis

We also ran several supplementary analyses to gauge robustness beyond our core DiD framework. We run a generalized synthetic controls approach^46^ as well as a Bayesian approach^87^ that aligns with our study’s panel data to ensure even tighter treatment counterfactuals. We use these techniques, as opposed to others such as Coarsened Exact Matching (CEM), as they are more appropriate to deploy both within a DiD framework and with panel data^88^. The results from the generalized synthetic control and Bayesian approaches are both robust and consistent with the results found in the main paper. A more detailed explanation of these methods can be found in the SI Appendix H, and we summarize the results of these robustness checks in Table 3.

**Table 3:** For all methods, the treated units were those whose populations have above 50% access to broadband.

	(1)	(2)	(3)	(4)
	DiD	DiD	Gsynth	bpCausal
Treated w/broadband × COVID shock	1.36*** (0.17)	1.34*** (0.16)	3.47*** (0.63)	2.26*** (0.16)
Growth rate of 25 + with BS degrees +		−0.34 (0.25)		0.09*** (0.02)
Percent of population 25 + with a BS degree +		1.00** (2.10)		-0.33*** (0.02)
Log of population density		0.50*** (0.09)		0.08*** (0.03)
Percent of population Black		2.24 (1.35)		0.35*** (0.02)
Percent of population Hispanic		0.54 (0.81)		0.19*** (0.02)
Percent of population Asian		1.70 (2.9)		0.02 (0.02)
Percent of population native American		4.71* (1.49)		0.32*** (0.02)
No. obs	49,984	49,984	49,984	49,984

Robust standard errors, clustered at the state level, are included below each estimate with statistical significance indicated by the stars based off of a two-tailed test. Method (1) uses a standard difference in difference regression approach, using the lm_robust regression package^91^, only including the variables of treatment and shock and we see the results are highly similar to our main results. Method (2) uses a standard difference in difference regression approach, again using the lm_robust regression package, including all proposed control variables used throughout the study. Method (3) uses the generalized synthetic control approach proposed by Xu^46^, as executed by the *gsynth* algorithm^92^. This approach relaxes the assumptions required for difference in difference analysis as they pertain to parallel trend assumptions, while combining the synthetic control approach with linear fixed effects. Method (4) uses a Bayesian approach proposed by Pang et al., as executed by their *bpCausal* algorithm^87^, in order to conduct a method that can integrate time-invariant covariates that the generalized synthetic approach cannot accommodate. This Bayesian approach is initialized through diffuse priors that come from a mixed normal-exponential distribution with a tuning parameter that seeks to balance between model variance and bias using values recommended from prior literature^93^**.**

### Boundary conditions: COVID case load

Another key assumption is that DiD also assumes no simultaneity (i.e., no other change that occurred at the same time that could plausibly change the results). While we cannot think of any other shock that would have occurred at the same time as COVID, we want to consider what impact COVID itself had. For instance, the Emergency Broadband Benefit program provided households with a supplemental income of $50/month to help pay for increased broadband access ^89^. Perhaps then, COVID-19 infection rates could drive how many take advantage of such a program. To probe into this, we separately did a placebo where we defined the treatment and control groups based on above and below median average COVID-19 cases for July 2020 (a representative month for the time frame we considered^90^). While we found some significance for these models, these effects greatly diminish once we incorporate the full set of controls (these results can be found in SI Appendix G9–G12). Moreover, they do not explain the same level of variation by industry as do the models where the treatment and control groups are based on broadband penetration (see Appendix G13 for these industry-specific results). Thus, we see this as evidence of the distinct role that broadband plays on unemployment beyond those impacts from increased COVID-19 infection rates. Nonetheless, COVID-19 case load naturally may still play a role in influencing how broadband impacts unemployment amidst the pandemic and hence we note it is an important boundary condition.

### Supplementary Information

Supplementary Information.

## Data Availability

All data used in this study is open-source, publicly available data. Some of the data is sourced through various APIs provided in the code above. When the data was pulled directly from an online repository, the downloaded file and relevant URL are also available at https://github.com/nikkiritsch/broadband_socialsensor.git.

## Code availability

The code used to run these difference-in-difference regression models is available online at https://github.com/nikkiritsch/broadband_socialsensor.git.
