# Supplementary material for: Using broadband infrastructure as a social sensor to detect inequities in unemployment during the COVID-19 pandemic
Source: Sci Rep. 2023 Dec 12;13:22031. doi: 10.1038/s41598-023-48019-2 (PMC10716178; doi:10.1038/s41598-023-48019-2)
Supplement: Supplementary file 1 — Supplementary Information. [file 41598_2023_48019_MOESM1_ESM.docx]

**SI Table of Contents**

This Supplementary Information (SI) section is provided to shed additional light on a variety of different aspects of the work conducted for this manuscript.

Table of Contents

[Appendix A: Supplemental Literature on the Relationship between Employment and Broadband 2](#_Toc122444224)

[Appendix B: COVID-19 State Work from Home Policies^15^ 4](#_Toc122444225)

[Appendix C: Different Modalities of Broadband Access 10](#_Toc122444226)

[Appendix D: Basic broadband speeds under generic use conditions^18^ 14](#_Toc122444227)

[Appendix E: Technical Details for Each Broadband Measurement Approach 15](#_Toc122444228)

[Appendix F: Exploratory Analysis for Regression Statistics 17](#_Toc122444229)

[Appendix G: Robustness Checks 23](#_Toc122444230)

[Appendix H: Synthetic Controls Analysis and Bayesian Causal Inference with Time-Series Cross-Sectional Data 27](#_Toc122444231)

[Appendix I: Regression Tables 30](#_Toc122444232)

[Appendix J: Appendix References 73](#_Toc122444233)

# Appendix A: Supplemental Literature on the Relationship between Employment and Broadband

There are two supplemental streams of literature we want to note here as being important background for our study. The first focuses on the existing economics of broadband work, specifically as it pertains to employment, with limited exploration allocated to understanding equity implications. The second stream of work pertains to the role broadband access has played during the COVID-19 pandemic.

The first stream focuses on the extensive analytical work which has explored the impacts of broadband access on employment. In general, prior studies find that broadband penetration is positively associated with employment rates. Gillett et al. find the availability of mass-market broadband for residential access, as defined by FCC’s Form 477, resulted in an increase of 1 – 1.4% in job growth over the years of 1998 – 2002^1^. Crandall and colleagues^2^ find that for every one percentage point increase in broadband penetration at a state level of assessment, “employment is projected to increase by 0.2 to 0.3 percent per year”. They argue this occurs because broadband infrastructure improves access to the full suite of Information Communications Technology (ICT) in a faster and cheaper manner. Similarly, Shideler^3^ finds that broadband’s deployment in Kentucky contributes 0.14 – 5.32% to total employment growth, as broadband infrastructure reduces costs of goods and services and/or increases market access to the same good and services, and thus leads to job creation and growth in total employment. Lehr and colleagues^4^ find results consistent to these prior studies at the zip code level, and they make similar arguments that this occurs because having access to broadband enhances economic activity, which in turn, helps to promote job creation. Based on data from the National Broadband Map and unemployment statistics, Jayakar and Eun-A Park^5^ find that counties with better broadband availability had lower unemployment rates in 2011, even when controlling for other macroeconomic factors. Kandilov and Renkow^6^ find that loans made under the U.S. Pilot Broadband Loan Program in 2002 and 2003 had a positive impact on employment. Some studies also find significant results that high levels of broadband penetration and adoption are positively associated with employment rates, especially in rural areas ^7,8^. However, the jury is still out on this topic somewhat as there is another body of literature which suggests that employment rates are unaffected by broadband expansion and adoption. While Kolko^9^ finds a relationship between broadband expansion and local economic growth, they state that average wage and the employment rate were unaffected. Others find that once the data is fully balanced, there is no observable economic payoff from an increase of internet speed^10^ and that increased broadband availability has no statistical impact on jobs or income over time^11^. In short, while there is predominant consensus that broadband positively impacts employment, there is debate as to whether this impact holds once analyses control for other macroeconomic factors.

A second stream of relevant literature focuses on assessing how the COVID-19 pandemic impacts employment, based off of access to broadband services. As of now, there are two relevant pieces of work in this space. The first is a working paper which finds that while income is correlated with differences in the ability to stay-at-home, the unequal distribution of broadband across regions drives most of the income effect ^12^. However, this work primarily focuses on the ability of an individual to work from home in light of their broadband connectivity and does not focus on what the lack of broadband connectivity may mean for employment. The other is a study by Isley and Low^13^ which finds that broadband adoption and availability may have been associated with economic benefits, namely employment, in rural America during March and April of 2020.

Our work serves to fill several gaps within both literature streams. The first is that overall, this work helps connect the aforementioned first stream of literature around the economic impacts of broadband with the second stream pertaining to these impacts amidst COVID-19, which are currently not linked throughout all of the United States. The second critical gap addresses a construct validity issue. Most prior work focuses on a single source of broadband data, namely FCC’s Form 477 which provides broadband measures of *advertised speed of broadband access*. However, other types of broadband measurement exist that more precisely measure both access via the American Community Survey’s (ACS) dataset and quality via Microsoft, Ookla and M-lab datasets. Yet, while there is a rich technical discussion on the implications of these alternative measures ^14^, FCC Form 477 is still the dataset predominantly used. This presents a construct validity challenge in prior studies regarding the measurement of *actual* (as opposed to advertised) broadband access and quality when assessing economic impact. This work addresses these construct validity issues through assessing consistency of findings across these varying datasets. Finally, Isley and Low’s^13^ study specifically notes the need for a future study to assess the change in economic indicators from the pre-pandemic to pandemic-era using a first differenced dependent variable, as we have done in the work presented here.

# Appendix B: COVID-19 State Work from Home Policies^15^

Table B1 shows the stay-at-home orders which were put in place by state, along with the date and time they were implemented. This data is from The National Academy for State Health Policy.

**Table B1**: COVID-10 State Work from Home Policies

| **State, District, and Territories** | **Effective Date** | **Duration or End Date** | **Resources** |
| --- | --- | --- | --- |
| Alabama | April 4, 2020 at 5:00 p.m. | - April 30, 2020 at 5:00 p.m. - Phased opening as of April 30 at 5:00 p.m. | - [Press Release](https://governor.alabama.gov/newsroom/2020/04/governor-ivey-issues-stay-at-home-order/) - [Health Officer's Order](https://governor.alabama.gov/assets/2020/04/Final-Statewide-Order-4.3.2020.pdf) - [Littler Article](https://www.littler.com/publication-press/publication/state-alabama-issues-stay-home-order) - [FAQs and More](https://governor.alabama.gov/newsroom/covid-19/) - [Amended Safer at Home Order](https://governor.alabama.gov/assets/2020/05/Safer-at-Home-Order-FINAL-5.8.2020.pdf) |
| Alaska | March 28, 2020 at 5:00 p.m. | - April 21, 2020 - Phased opening as of April 24 | - [Health Mandate 011](https://content.govdelivery.com/accounts/AKDHSS/bulletins/283a713) - [Health Mandate](https://gov.alaska.gov/home/covid19-healthmandates/) (extension) - All [COVID-19 Health Mandates](http://dhss.alaska.gov/dph/Epi/id/Pages/COVID-19/default.aspx) (including details for reopening in attachments) - [List of Essential Businesses](https://gov.alaska.gov/wp-content/uploads/sites/2/03232020-COVID-19-Health-Mandate-010-Attachment-A.pdf) |
| Arizona | March 31, 2020 at 5:00 p.m. | - May 15, 2020 at 11:59 p.m. - Phased opening as of May 16 | - [Press Release](https://azgovernor.gov/governor/news/2020/03/new-executive-order-stay-home-stay-healthy-stay-connected) - [Press Release](https://azgovernor.gov/governor/news/2020/04/governor-ducey-announces-updated-guidance-arizonans-businesses) (extension) - [Executive Order 2020-18](https://www.azdhs.gov/documents/preparedness/epidemiology-disease-control/infectious-disease-epidemiology/novel-coronavirus/eo-stay-home-stay-healthy-stay-connected.pdf) - [Executive Order 2020-33](https://azgovernor.gov/sites/default/files/eo_2020-33_0.pdf) - [Guidance on Essential Services](https://azgovernor.gov/governor/news/2020/04/additional-guidance-essential-services) - [Executive Order 2020-36](https://azgovernor.gov/sites/default/files/executive_order_2020-36_return_stronger.pdf) (reopening) |
| California | March 19, 2020 | - Phased opening as of May 8 | - [Executive Order N-33-20](https://covid19.ca.gov/img/Executive-Order-N-33-20.pdf) - [FAQs](https://covid19.ca.gov/stay-home-except-for-essential-needs/) - [Littler Article](https://www.littler.com/publication-press/publication/california-stay-home-order-breaking-it-down) |
| Colorado | March 26, 2020 at 6:00 a.m. | - April 26, 2020 - Phased opening as of April 27 | - [Press Release](https://www.colorado.gov/governor/news/gov-polis-announces-statewide-stay-home-order-provides-update-colorado-response-covid-19) - [Executive Order](https://drive.google.com/file/d/1O1EDCY6-A6QBKxzDImCSF8bBBdOOI3Km/view?usp=sharing) - [Executive Order 2020-24](https://drive.google.com/file/d/1UHbu2_DTdTvSlPcpsd_vDLtnaslmISuo/view) (extension) - [FAQs](https://drive.google.com/file/d/16nO05S6q0AGBew32r0NYelifwk11uDMO/view?usp=sharing) - [Stay at Home Page](https://covid19.colorado.gov/stay-home-except-essential-needs) |
| Connecticut | March 23, 2020 at 8:00 p.m. | - May 20, 2020 - Phased opening as of May 20 | - [Executive Order No.](https://portal.ct.gov/-/media/Office-of-the-Governor/Executive-Orders/Lamont-Executive-Orders/Executive-Order-No-7H.pdf) [7H](https://portal.ct.gov/-/media/Office-of-the-Governor/Executive-Orders/Lamont-Executive-Orders/Executive-Order-No-7H.pdf) - [Business Exemptions Guidance](https://portal.ct.gov/DECD/Content/Coronavirus-for-Businesses/Coronavirus-for-Businesses) - [FAQs](https://portal.ct.gov/Coronavirus) - [Littler Article](https://www.littler.com/publication-press/publication/new-york-new-jersey-and-connecticut-issue-restrictions-workplace) - See also [Executive Order 7N](https://portal.ct.gov/-/media/Office-of-the-Governor/Executive-Orders/Lamont-Executive-Orders/Executive-Order-No-7N.pdf?la=en) - [Executive Order 7PP](https://officeofthegovernor.cmail19.com/t/j-l-qtikuul-tkujztjyh-b/) (reopening) |
| Delaware | March 24, 2020 at 8:00 a.m. | - May 15, 2020 or until health threat eliminated - Phased opening targeted for June 1 | - [Press Release](https://news.delaware.gov/2020/03/22/governor-carney-issues-stay-at-home-order-for-delawareans/) - [FAQs](https://governor.delaware.gov/wp-content/uploads/sites/24/2020/03/FAQ-on-Non-essential-Business-Closure-State-of-Delaware-03222020.pdf) - [List of Essential Businesses](https://coronavirus.delaware.gov/wp-content/uploads/sites/177/2020/03/Delaware-list-of-essential-and-nonessential-businesses-March-22-2020-1.pdf) - [Press Release - Phase One Target Date](https://news.delaware.gov/2020/05/08/governor-carney-releases-statement-on-economic-reopening-announces-june-1-as-target-for-phase-i/) |
| District of Columbia | April 1, 2020 at 12:01 a.m. | June 8, 2020 | - [Press Release & Order](https://coronavirus.dc.gov/release/mayor-bowser-issues-stay-home-order) - [Press Release](https://coronavirus.dc.gov/release/mayor-bowser-extends-public-health-emergency-stay-home-order-and-closure-non-essential) (extension) - [Order](https://coronavirus.dc.gov/sites/default/files/dc/sites/coronavirus/publication/attachments/MayorsOrder2020.063.pdf) (extension) - [Littler Article](https://www.littler.com/publication-press/publication/covid-19-guide-employers-dmv-dc-maryland-virginia) - [Order](https://coronavirus.dc.gov/sites/default/files/dc/sites/coronavirus/page_content/attachments/Mayors-Order-2020-066-Extensions-of-Public-Emergency-and-Public-Health.pdf) (extension to June 8) |
| Florida | April 3, 2020 at 12:01 a.m. | - April 30, 2020 - Phased opening as of May 1 | - [Executive Order 20-91](https://s33330.pcdn.co/wp-content/uploads/2020/04/EO-20-91.pdf) - [Executive Order 20-92](https://www.flgov.com/wp-content/uploads/orders/2020/EO_20-92.pdf) |
| Georgia – for vulnerable individuals only as of May 1 | April 3, 2020 at 6:00 p.m. | - April 30, 2020 at 11:59 p.m. (for general population) - Phased opening as of April 24 - June 12, 2020 (for elderly and medically fragile individuals) | - [Press Release](https://gov.georgia.gov/press-releases/2020-04-01/kemp-toomey-carden-bryson-give-covid-19-update) - [Executive Order 04.02.20.01](https://gov.georgia.gov/document/2020-executive-order/04022001/download) - [Executive Order 04.08.20.02](https://gov.georgia.gov/document/2020-executive-order/04082002/download) - [Executive Order 04.30.20.01](https://gov.georgia.gov/document/2020-executive-order/04302001/download) - [Additional Guidance](https://t.co/p7qclV3QZA?amp=1) - [Littler Article](https://www.littler.com/publication-press/publication/georgia-issues-state-wide-shelter-place-order) |
| Hawaii | March 25, 2020 at 12:01 a.m. | - May 31, 2020 at 11:59 p.m. - Phased opening as of May 7 | - [Proclamation](https://hawaiicovid19.com/wp-content/uploads/2020/03/2003162-ATG_Third-Supplementary-Proclamation-for-COVID-19-signed-12.pdf) - [Proclamation](https://governor.hawaii.gov/wp-content/uploads/2020/04/2004144-ATG_Sixth-Supplementary-Proclamation-for-COVID-19-distribution-signed.pdf) (extension) - [Press Release](https://governor.hawaii.gov/newsroom/latest-news/office-of-the-governor-news-release-governor-ige-issues-statewide-order-to-stay-at-home-work-from-home-to-fight-covid-19/) - [Press Release](https://governor.hawaii.gov/newsroom/latest-news/governors-office-news-release-gov-ige-extends-stay-at-home-order-across-the-state-through-may-31/) (extension) |
| Idaho | March 25, 2020 | - April 30, 2020 - Phased opening as of May 1 | - [Press Release](https://gov.idaho.gov/pressrelease/governor-little-issues-statewide-stay-home-order-signs-extreme-emergency-declaration/) - [Order to Self-Isolate](https://coronavirus.idaho.gov/wp-content/uploads/sites/127/2020/03/statewide-stay-home-order_032520.pdf) - [FAQs](https://coronavirus.idaho.gov/wp-content/uploads/sites/127/2020/03/032520_Order-FAQ_updated.pdf) - [Stay at Home Page](https://coronavirus.idaho.gov/) - [Amended Order](https://coronavirus.idaho.gov/statewide-stay-home-order/) |
| Illinois | March 21, 2020 at 5:00 p.m. | May 30, 2020 | - [Executive Order 2020-18](https://www2.illinois.gov/Pages/Executive-Orders/ExecutiveOrder2020-18.aspx) - [Executive Order 2020-32](https://www2.illinois.gov/Pages/Executive-Orders/ExecutiveOrder2020-32.aspx) (extension) - [FAQs](https://coronavirus.illinois.gov/s/stay-at-home-faqs) - [Littler Article](https://www.littler.com/publication-press/publication/illinois-stay-home-what-does-mean-employers) |
| Indiana | March 24, 2020 at 11:59 p.m. | - May 4, 2020 at 11:59 p.m. - Phased opening as of May 5 | - [Executive Order 20-08](https://www.in.gov/gov/files/Executive_Order_20-08_Stay_at_Home.pdf) - [Press Release](https://www.in.gov/gov/3232.htm) - [Executive Order 20-18](https://www.in.gov/gov/files/Executive%20Order%2020-18%20Cont%20Stay%20at%20Home%20Restaurants%20Govt%20Ops.pdf) (extension) - [Executive Order 20-22](https://www.in.gov/gov/files/Executive%20Order%2020-22%20Extension%20of%20Stay%20at%20Home.pdf) (further extension) - [Executive Order 20-26](https://www.in.gov/gov/files/Executive%20Order%2020-26%20Roadmap%20to%20Reopen.pdf) (Stages 1 and 2) - Updated [FAQs](https://www.in.gov/gov/3232.htm) - All [Executive Orders](https://www.in.gov/gov/2384.htm) |
| Kansas | March 30, 2020 at 12:01 a.m. | - May 3, 2020 - Phased opening as of May 4 | - [Press Release](https://governor.kansas.gov/governor-kelly-issues-temporary-statewide-stay-home-order-in-ongoing-effort-to-combat-covid-19/) - [Executive Order](https://governor.kansas.gov/wp-content/uploads/2020/03/EO20-16.pdf) - [Executive Order](https://governor.kansas.gov/wp-content/uploads/2020/04/EO-20-24-Executed.pdf) (extension) |
| Kentucky^*^ | March 26, 2020 at 8:00 p.m. | - Until the emergency concludes - Phased opening as of May 11 | - [Press Release](https://kentucky.gov/Pages/Activity-stream.aspx?n=GovernorBeshear&prId=104) - [Executive Order 2020-257](https://governor.ky.gov/attachments/20200325_Executive-Order_2020-257_Healthy-at-Home.pdf) - [Littler Article](https://www.littler.com/publication-press/publication/kentucky-closes-all-nonessential-retail-businesses) |
| Louisiana | March 23, 2020 at 5:00 p.m. | - May 14, 2020 - Phased opening as of May 15 | - [Proclamation 33 JBE 2020](https://gov.louisiana.gov/assets/Proclamations/2020/JBE-33-2020.pdf) - [Proclamation 41 JBE 2020](https://gov.louisiana.gov/assets/Proclamations/2020/41-JBE-2020-Stay-At-Home-Extended.pdf) (extension) - [Press Release](https://gov.louisiana.gov/home-order-extended-may15/) (further extension) - [Littler Article](https://www.littler.com/publication-press/publication/louisiana-stay-home-proclamation-effective-march-23-what-does-it-mean) - All [Proclamations](https://gov.louisiana.gov/index.cfm/newsroom/category/10) - [Help Desk for Businesses](https://gov.louisiana.gov/index.cfm/newsroom/detail/2437) |
| Maine | April 2, 2020 at 12:01 a.m. | - May 31, 2020 - Phased opening as of May 11, particularly in rural counties | - [Press Release](https://www.maine.gov/governor/mills/news/governor-mills-issues-stay-healthy-home-mandate-2020-03-31) - [Press Release](https://www.maine.gov/governor/mills/news/governor-mills-presents-safe-gradual-plan-restart-maines-economy-2020-04-28) (extension) - [Executive Order](https://www.maine.gov/governor/mills/sites/maine.gov.governor.mills/files/inline-files/CORRECTED_An%20Order%20Regarding%20Further%20Restrictions%20on%20Public%20Contact%20and%20Movement%2C%20Schools%2C%20Vehicle%20Travel%20and%20Retail%20Business%20Operations.pdf) |
| Maryland | March 30, 2020 at 8:00 p.m. | - May 15 at 5:00 p.m. - Phased opening as of May 15 at 5:00 p.m., in most counties | - [Press Release](https://governor.maryland.gov/2020/03/30/as-covid-19-crisis-escalates-in-capital-region-governor-hogan-issues-stay-at-home-order-effective-tonight/) - [Executive Order 20-03-30-01](http://governor.maryland.gov/wp-content/uploads/2020/03/Gatherings-FOURTH-AMENDED-3.30.20.pdf) - [Executive Order 20-05-06-01](https://governor.maryland.gov/wp-content/uploads/2020/05/Gatherings-FIFTH-AMENDED-5.6.20.pdf) (5/6/20) - [Littler Article](https://www.littler.com/publication-press/publication/covid-19-guide-employers-dmv-dc-maryland-virginia) |
| Massachusetts^*^ | March 24, 2020 at 12:00 noon; updated order effective April 1, 2020 at noon | - May 18, 2020 - Phased opening as of May 18 | - [Press Release](https://www.mass.gov/news/baker-polito-administration-extends-non-essential-business-closures-and-executive-branch) (3/31/20) - [Press Release](https://www.mass.gov/news/baker-polito-administration-extends-non-essential-business-closures-to-may-18th-announces) (4/28/20) - [Emergency Order](https://www.mass.gov/doc/march-23-2020-essential-services-and-revised-gatherings-order) - [Extension Order](https://www.mass.gov/doc/march-31-2020-essential-services-extension-order) - Updated [List of Essential Services](https://www.mass.gov/doc/march-31-essential-services-list) - [Essential Services FAQs](https://www.mass.gov/info-details/covid-19-essential-services-faqs) - [Littler Article](https://www.littler.com/publication-press/publication/massachusetts-stay-home-advisory-effective-march-24) - [Safer at Home Advisory](https://www.mass.gov/news/safer-at-home-advisory) (5/18/20) - [COVID-19 Order No. 33](https://www.mass.gov/doc/may-18-2020-re-opening-massachusetts-order) (5/18/20) |
| Michigan | March 24, 2020 at 12:01 a.m. | - May 28, 2020 at 11:59 p.m. - Phased opening as of May 7 | - [Press Release](https://www.michigan.gov/whitmer/0,9309,7-387-90487-522625--,00.html) - [Press Release](https://www.michigan.gov/whitmer/0,9309,7-387-90499-525173--,00.html) (extension) - [Press Release](https://www.michigan.gov/whitmer/0,9309,7-387-90499_90640-527845--,00.html) (further extension) - [Executive Order](https://content.govdelivery.com/attachments/MIEOG/2020/03/23/file_attachments/1408152/EO%202020-21%20Stay%20Home,%20Stay%20Safe.pdf) - [Executive Order](https://content.govdelivery.com/attachments/MIEOG/2020/04/09/file_attachments/1423850/EO%202020-42.pdf) (extension) - [Executive Order](https://www.michigan.gov/whitmer/0,9309,7-387-90499_90705-527847--,00.html) (further extension) - [Executive Order](https://gcc01.safelinks.protection.outlook.com/?url=https%3A%2F%2Flnks.gd%2Fl%2FeyJhbGciOiJIUzI1NiJ9.eyJidWxsZXRpbl9saW5rX2lkIjoxMDIsInVyaSI6ImJwMjpjbGljayIsImJ1bGxldGluX2lkIjoiMjAyMDA1MDcuMjExOTk3NDEiLCJ1cmwiOiJodHRwczovL2NvbnRlbnQuZ292ZGVsaXZlcnkuY29tL2F0dGFjaG1lbnRzL01JRU9HLzIwMjAvMDUvMDcvZmlsZV9hdHRhY2htZW50cy8xNDQ2MTI0L0VPJTIwMjAyMC03Ny5wZGYifQ.yqtIu2g9Pj0t78UQllLKd0obAbQqONul24LfrCIIM_g%2Fbr%2F78380114846-l&data=02%7C01%7Csaylort%40michigan.gov%7C0749655b291f41b6a57d08d7f2b9608e%7Cd5fb7087377742ad966a892ef47225d1%7C0%7C0%7C637244750309814062&sdata=mXLfP3oAV4olq36HSUCqaWwTnAXQc9elFrMfL%2F%2FP3B4%3D&reserved=0) (extension to May 28) - [Littler Article](https://www.littler.com/publication-press/publication/michigan-issues-stay-home-stay-safe-executive-order) - [Littler Article](https://www.littler.com/publication-press/publication/michigan-issues-executive-order-extending-retaliation-protections-amid) - Retaliation Protections |
| Minnesota | March 27, 2020 at 11:59 p.m. | - May 17, 2020 at 11:59 p.m. - Phased opening as of April 27 | - [Executive Order](https://mn.gov/governor/assets/EO%2020-20%20FINAL_tcm1055-424864.pdf) - [Executive Order](https://www.leg.state.mn.us/archive/execorders/20-33.pdf) (extension) - [Press Release](https://mn.gov/governor/covid-19/news/#/detail/appId/1/id/430501) (further extension) - [FAQs](https://mn.gov/governor/covid-19/faq.jsp) - [Stay at Home Page](https://mn.gov/governor/covid-19/) - [Executive Order 20-56](https://mn.gov/governor/assets/EO%2020-56%20Final_tcm1055-431921.pdf) (5/13/20) |
| Mississippi | April 3, 2020 at 5:00 p.m. | - April 27, 2020 at 8:00 a.m. - Phased opening as of April 27 | - [Executive Order](https://www.sos.ms.gov/Education-Publications/ExecutiveOrders/1466.pdf) - [Executive Order](https://www.sos.ms.gov/content/executiveorders/ExecutiveOrders/1473.pdf) (extension) - [FAQs](https://governorreeves.ms.gov/wp-content/uploads/Shelter-In-Place-FAQ-Provided-4.13.2020.pdf) |
| Missouri^1^ | April 6, 2020 at 12:01 a.m. | - May 3, 2020 - Phased opening as of May 4 | - [Press Release](https://governor.mo.gov/press-releases/archive/governor-parson-issues-statewide-stay-home-missouri-order-control-contain) - [Press Release](https://governor.mo.gov/press-releases/archive/governor-parson-extends-statewide-stay-home-missouri-order-through-may-3) (extension) - [Health Director's Order](https://content.govdelivery.com/attachments/MOGOV/2020/04/03/file_attachments/1419322/Stay%20at%20Home%20Missouri%20Order.pdf) - [Health Director's Order](https://governor.mo.gov/priorities/extension-stay-home-order-covd-19) (extension) - [FAQs](https://governor.mo.gov/stay-home-missouri-order-guidance-and-frequently-asked-questions) - [Littler Article](https://www.littler.com/publication-press/publication/what-employers-need-know-about-newly-issued-stay-home-missouri-order) |
| Montana | March 28, 2020 at 12:01 a.m. | - April 26, 2020 (for individuals) - Phased opening as of April 27 | - [Press Release](http://governor.mt.gov/Pressroom/governor-bullock-extends-directives-issued-to-respond-to-covid-19-pandemic) (Extension) - [Directive Implementing Executive Orders](https://covid19.mt.gov/Portals/223/Documents/Stay%20at%20Home%20Directive.pdf?ver=2020-03-26-173332-177) - [Extension of Directives](http://governor.mt.gov/Portals/16/Extension%20of%20Directives.pdf?ver=2020-04-07-172755-170) - [FAQs](https://montana.maps.arcgis.com/apps/MapSeries/index.html?appid=7c34f3412536439491adcc2103421d4b) |
| Nevada | April 1, 2020 at midnight | - May 15, 2020 - Phased opening as of May 1 - Phase 1 as of May 9 | - [Press Release](https://nvhealthresponse.nv.gov/wp-content/uploads/2020/04/Governor-Sisolak-announces-Stay-at-Home-directive-extends-closure-date-to-the-end-of-April.pdf) - [Directive 010](https://nvhealthresponse.nv.gov/wp-content/uploads/2020/04/Declaration-of-Emergency-Directive-010-Stay-at-Home-3-31-20.pdf) (Stay at Home) - [Directive 003](https://t.co/5VXFJIEGxW) (Business Closures) - [Directive 016](https://nvhealthresponse.nv.gov/news-resources/governor-directives-and-declarations/) (Extension) - [Guidance on Directive 016](https://nvhealthresponse.nv.gov/wp-content/uploads/2020/04/Governor-Sisolak-Directive-016-Guidance-4-29-20.pdf) - [Implementing Regulations](https://t.co/bSxalZWX7j) |
| New Hampshire | March 27, 2020 at 11:59 p.m. | - May 31, 2020 (Stay at Home 2.0) - Phased opening as of May 4 | - [Emergency Order No. 17](https://www.nheconomy.com/NHEconomy/media/NH-Economy/2020-0-Emergency-Order-(1)-17_1.pdf) - [Emergency Order No. 40](https://www.governor.nh.gov/news-media/emergency-orders/documents/emergency-order-40.pdf) - [List of Essential Businesses](https://t.co/RmCrgWG3Hm?amp=1) |
| New Jersey | March 21, 2020 at 9:00 p.m. | Until revoked or modified | - [Executive Order 107](https://nj.gov/infobank/eo/056murphy/pdf/EO-107.pdf) - [All Executive Orders](https://nj.gov/infobank/eo/056murphy/approved/eo_archive.html) - [Littler Article](https://www.littler.com/publication-press/publication/new-york-new-jersey-and-connecticut-issue-restrictions-workplace) - [FAQs for Businesses](https://faq.business.nj.gov/en/collections/2198378-information-for-nj-businesses-on-the-coronavirus-outbreak) |
| New Mexico | March 23, 2020 | - Until at least May 31, 2020 - Phased opening as of May 16 | - [Press Release](https://cv.nmhealth.org/2020/03/23/state-enacts-further-restrictions-to-stop-spread-including-stay-at-home-instruction/) - [Press Release](https://www.governor.state.nm.us/2020/04/06/governor-officially-extends-emergency-order-to-april-30/) (extension) - [Press Release](https://www.governor.state.nm.us/2020/04/30/state-extends-modified-stay-home-order/) (further extension) - [Public Health Order](https://www.governor.state.nm.us/wp-content/uploads/2020/03/COVID-19-DOH-Order-fv.pdf) - [Public Health Order](https://www.governor.state.nm.us/wp-content/uploads/2020/04/040620-DOH-PHO.pdf) (extension) - [Public Health Order](https://www.governor.state.nm.us/wp-content/uploads/2020/05/DOH-PHO-4-29-essential-businesses-mass-gatherings-fv.pdf) (further extension) - [FAQs](https://cv.nmhealth.org/stay-at-home-faqs/) - [List of Essential Businesses](https://cv.nmhealth.org/stay-at-home-essential-businesses/) - [Press Release re: Phased Opening](https://www.governor.state.nm.us/2020/05/13/state-to-further-modify-public-health-emergency-order/) (5/13/20) - [Executive Order 2020-30](https://cv.nmhealth.org/wp-content/uploads/2020/05/2020-30-phase-1A.pdf) (5/15/20) |
| New York | March 22, 2020 | - May 28, 2020, for regions that have not started to reopen - Phased opening as of May 15; on a regional basis | - [Press Release](https://www.governor.ny.gov/news/governor-cuomo-signs-new-york-state-pause-executive-order) - [Press Release](https://www.governor.ny.gov/news/amid-ongoing-covid-19-pandemic-governor-cuomo-announces-nys-pause-functions-extended-additional) (extension) - [Press Release](https://www.governor.ny.gov/news/amid-ongoing-covid-19-pandemic-governor-cuomo-announces-nys-pause-extended-until-may-15) (further extension) - [Executive Order 202.8](https://www.governor.ny.gov/news/no-2028-continuing-temporary-suspension-and-modification-laws-relating-disaster-emergency) - [Executive Order 202.18](https://www.governor.ny.gov/news/no-20218-continuing-temporary-suspension-and-modification-laws-relating-disaster-emergency) - [All Executive Orders](https://www.governor.ny.gov/executiveorders) - [Littler Article](https://www.littler.com/publication-press/publication/new-york-new-jersey-and-connecticut-issue-restrictions-workplace) - [Littler Article](https://www.littler.com/publication-press/publication/covid-19-roadmap-new-york-employers) - NY Roadmap - [Executive Order 202.31](https://www.governor.ny.gov/news/no-20231-continuing-temporary-suspension-and-modification-laws-relating-disaster-emergency) (extension/regional reopening) - [Press Release](https://www.governor.ny.gov/news/amid-ongoing-covid-19-pandemic-governor-cuomo-announces-five-regions-will-begin-reopening-today) (extension to May 28) |
| North Carolina | March 30, 2020 at 5:00 p.m. | - May 8, 2020 at 5:00 p.m. - Phased opening as of May 8 at 5:00 p.m. | - [Executive Order](https://files.nc.gov/governor/documents/files/EO121-Stay-at-Home-Order-3.pdf) - [Press Release](https://governor.nc.gov/news/governor-extends-stay-home-order-through-may-8-plans-three-phase-lifting-restrictions-based) (extension) - [Executive Order](https://files.nc.gov/governor/documents/files/EO135-Extensions.pdf) (extension) - [Executive Order](https://files.nc.gov/governor/documents/files/EO138-Phase-1.pdf) (reopening) - [FAQs](https://t.co/wV1j6SNrGT) |
| Ohio | March 23, 2020 at 11:59 p.m. | - May 29, 2020 at 11:59 p.m. - Phased opening as of May 4 | - [Press Release](https://ohio.gov/wps/portal/gov/site/media-center/news-and-events/ohio-issues-stay-at-home-order-and-new-restrictions-placed-on-day-cares-for-children) - [Stay at Home Order](https://coronavirus.ohio.gov/static/DirectorsOrderStayAtHome.pdf) - [Amended Stay at Home Order](https://coronavirus.ohio.gov/static/publicorders/Directors-Stay-At-Home-Order-Amended-04-02-20.pdf) - [Stay Safe Ohio Order](https://coronavirus.ohio.gov/static/publicorders/Directors-Stay-Safe-Ohio-Order.pdf) (4/30/20) - [FAQs](https://gcc01.safelinks.protection.outlook.com/?url=https%3A%2F%2Fcontent.govdelivery.com%2Fattachments%2FOHOOD%2F2020%2F03%2F22%2Ffile_attachments%2F1407841%2FStay%2520At%2520Home%2520FAQ%252003.22.20.pdf&data=02%7C01%7Ccatherine.sulecki%40governor.ohio.gov%7Cf09fb0ea1e904f11109f08d7ce9eec29%7C50f8fcc494d84f0784eb36ed57c7c8a2%7C0%7C1%7C637205054274020549&sdata=s3G0Z9osnnWaYqAbXSK4%2B995PyT8wpPZTv%2BVHDa7Grs%3D&reserved=0) - [Littler Article](https://www.littler.com/publication-press/publication/ohio-stay-home-order-effective-march-23-what-it-means-employers) |
| Oklahoma – for vulnerable individuals only | March 24, 2020 | May 24, 2020 | - [Executive Order 2020-07](https://www.sos.ok.gov/documents/executive/1926.pdf) (7th am.) - [Press Release](https://www.governor.ok.gov/articles/press_releases/gov-stitt-extends-safer-at-home-order) (extension) - [Executive Order 2020-13](https://www.sos.ok.gov/documents/executive/1931.pdf) (extension) |
| Oregon | March 23, 2020 | - Until terminated - Phased reopening as of May 15; by county | - [Press Release](https://www.oregon.gov/newsroom/Pages/NewsDetail.aspx?newsid=36240) - [Executive Order 20-12](https://govsite-assets.s3.amazonaws.com/jkAULYKcSh6DoDF8wBM0_EO%2020-12.pdf) - [Littler Article](https://www.littler.com/publication-press/publication/oregon-issues-stay-home-save-lives-executive-order) - [Stay at Home Page](https://govstatus.egov.com/or-covid-19) - [Executive Order 20-25](https://www.oregon.gov/gov/Documents/executive_orders/eo_20-25.pdf) |
| Pennsylvania | April 1, 2020 at 8:00 p.m. | - May 8, 2020 - Phased opening as of May 8; based on county | - [Press Release](https://www.governor.pa.gov/newsroom/gov-wolf-sec-of-health-pennsylvania-on-statewide-stay-at-home-order-beginning-at-8-pm-tonight-most-prudent-option-to-stop-the-spread/) - [Press Release](https://www.governor.pa.gov/newsroom/gov-wolf-sec-of-health-extend-statewide-stay-at-home-order-until-may-8/) (extension) - [Executive Order](https://www.governor.pa.gov/wp-content/uploads/2020/04/20200401-GOV-Statewide-Stay-at-Home-Order.pdf) - [Executive Order](https://www.governor.pa.gov/wp-content/uploads/2020/04/20200420-GOV-Stay-at-Home-Order-Amendment.pdf) (extension) - [Health Mandate](https://www.scribd.com/document/454418390/04-01-20-SOH-Statewide-Stay-at-Home-Order) - [Health Mandate](https://www.governor.pa.gov/wp-content/uploads/2020/04/20200420-SOH-Stay-at-Home-Order-Amendment.pdf) (extension) - [Business Guidance](https://www.scribd.com/document/452553026/UPDATED-Industry-Operation-Guidance-March-20-2020) - [FAQs](https://www.scribd.com/document/452553495/Life-Sustaining-Business-FAQs) - [Littler Article](https://www.littler.com/publication-press/publication/update-pennsylvania-covid-19-business-closures-impending-waiver) |
| Puerto Rico | March 15, 2020 | - May 25, 2020 - Phased opening as of May 4 | - [Lockdown and Closures Order](https://www.littler.com/files/oe-2020-029.pdf) (3/30/20) - [Lockdown and Closures Extension](http://file:/C:/Users/Bcammarata/Downloads/Orden%20Ejecutiva%202020-033%20English.pdf) (4/12/20) - [Littler Article](https://www.littler.com/publication-press/publication/puerto-rico-extends-lockdown-and-curfew-imposes-additional) (4/1/20) - [Littler Article](https://www.littler.com/publication-press/publication/key-takeaways-puerto-ricos-latest-covid-19-related-executive-order) (4/13/20) - [Littler Article](https://www.littler.com/publication-press/publication/governor-puerto-rico-extends-curfew-and-relaxes-lockdown) (5/2/20) - [Lockdown Extended/Reopen Steps](https://basecero.ogp.pr.gov/apex/apex_util.get_blob?s=12940596661289&a=161&c=112063554695324788&p=15&k1=4776&k2=&ck=Fy7TYGugLOipubJ4D-fJ0koIdfmr2q3kVzANk0CZTdzTSM9n5W90v25vf1G5i8C-N0tvvmT9hJscZH8QJm_l0Q&rt=IR) (5/1/20) |
| Rhode Island | March 28, 2020 | - May 8, 2020 - Phased opening as of May 9 | - [Press Release](https://www.ri.gov/press/view/38033) - [Press Release](https://www.ri.gov/press/view/38091) (extension) - [Executive Order](http://www.governor.ri.gov/documents/orders/Executive-Order-20-13.pdf) - [Executive Order](http://www.governor.ri.gov/documents/orders/Executive-Order-20-18.pdf) (extension) - [List of Critical Businesses](https://dbr.ri.gov/documents/DBR_Critical_retail_businesses_list_032820.pdf) |
| South Carolina | April 7, 2020 at 5:00 p.m. | - May 3, 2020 - Phased opening as of May 4 | - [Executive Order 2020-21](https://governor.sc.gov/sites/default/files/Documents/Executive-Orders/2020-04-06%20eFILED%20Executive%20Order%20No.%202020-21%20-%20Stay%20at%20Home%20or%20Work%20Order.pdf) - [Press Release](https://governor.sc.gov/news/2020-05/gov-henry-mcmaster-lift-work-or-home-order-may-4th) - Lifting Work or Home Order - [Executive Order 2020-31](https://governor.sc.gov/sites/default/files/Documents/Executive-Orders/2020-05-03%20eFILED%20Executive%20Order%20No.%202020-31%20-%20Modification%20of%20Home%20or%20Work%20Order%20%26%20Authorization%20of%20Outdoor%20Dining%20Services.pdf) (lifting) |
| Tennessee | March 31, 2020 at 11:59 p.m. | - April 30, 2020 at 11:59 p.m. - Phased opening as of April 27 | - [Press Release](https://www.tn.gov/governor/news/2020/4/2/gov--lee-requires-tennesseans-to-remain-at-home-as-data-shows-increased-activity-among-citizens-.html) (Order No. 23) - [Press Bulletin](https://www.tn.gov/governor/covid-19/covid-19-daily-bulletin/2020/3/30/covid-19-bulletin--8.html) (Order No. 22) - [Executive Order No. 22](https://publications.tnsosfiles.com/pub/execorders/exec-orders-lee22.pdf) (advisory) - [Executive Order No. 23](http://publications.tnsosfiles.com/pub/execorders/exec-orders-lee23.pdf) (mandatory) - [Executive Order No. 27](https://publications.tnsosfiles.com/pub/execorders/exec-orders-lee27.pdf) (extension) |
| Texas | April 2, 2020 at 12:01 a.m. | - April 30, 2020 - Phased opening as of May 1 | - [Press Release](https://gov.texas.gov/news/post/governor-abbott-issues-executive-order-implements-statewide-essential-services-and-activities-protocols) - [Executive Order](https://gov.texas.gov/uploads/files/press/EO-GA-14_Statewide_Essential_Service_and_Activity_COVID-19_IMAGE_03-31-2020.pdf) - [List of Essential Services](http://www.tdem.texas.gov/essentialservices/) |
| Vermont | March 25, 2020 at 5:00 p.m. | - May 15, 2020 - Phased opening as of April 20 | - [Press Release](https://governor.vermont.gov/press-release/governor-phil-scott-issues-%E2%80%9Cstay-home-stay-safe%E2%80%9D-order-directs-additional-closures) - [Press Release](https://governor.vermont.gov/press-release/governor-phil-scott-extends-state-emergency-vermont) (extension) - [Executive Order](https://governor.vermont.gov/sites/scott/files/documents/ADDENDUM%206%20TO%20EXECUTIVE%20ORDER%2001-20.pdf) - [Extension Order](https://governor.vermont.gov/sites/scott/files/documents/ADDENDUM%209%20TO%20EXECUTIVE%20ORDER%2001-20.pdf) (extension) - [FAQs](https://accd.vermont.gov/covid-19-guidance/stay-home-stay-safe-business-faqs) - [Updated Guidance](https://accd.vermont.gov/news/update-new-work-safe-additions-stay-home-stay-safe-order) (4/17/20) |
| Virginia | March 30, 2020 | - June 10, 2020 - Phased opening as of May 15, outside Northern Virginia Region - Phased opening for Northern Virginia Region as of May 29 | - [Press Release](https://www.governor.virginia.gov/newsroom/all-releases/2020/march/headline-855702-en.html) - [Executive Order No. 55](https://www.governor.virginia.gov/media/governorvirginiagov/executive-actions/EO-55-Temporary-Stay-at-Home-Order-Due-to-Novel-Coronavirus-(COVID-19).pdf) - [FAQs](https://www.virginia.gov/coronavirus/faq/) - [Littler Article](https://www.littler.com/publication-press/publication/virginia-issues-stay-home-executive-order) - Virginia - [Littler Article](https://www.littler.com/publication-press/publication/covid-19-guide-employers-dmv-dc-maryland-virginia) - DMV - [Executive Order No. 62](https://www.governor.virginia.gov/media/governorvirginiagov/executive-actions/EO-62-and-Order-of-Public-Health-Emergency-Four---Jurisdictions-Temporarily-Delayed-From-Entering-Phase-One-in-Executive-Order-61-and-Permitted-to-Remain-in-Phase-Zero-Northern-Virginia-Region.pdf) (Northern Virginia Region) |
| Washington State | March 23, 2020 | - May 31, 2020 - Phased opening as of May 5 | - [Announcement](https://medium.com/wagovernor/inslee-announces-stay-home-stay-healthy-order-4891a7511f5e) - [Proclamation 20-25](http://www.governor.wa.gov/sites/default/files/proclamations/20-25%20Coronovirus%20Stay%20Safe-Stay%20Healthy%20%28tmp%29%20%28002%29.pdf) - [Proclamation 20-25.1](https://www.governor.wa.gov/sites/default/files/20-25.1%20-%20COVID-19%20-%20Stay%20Home%2C%20Stay%20Healthy%20Extension%20%28tmp%29.pdf?utm_medium=email&utm_source=govdelivery) (extension) - [List of Essential Critical Infrastructure Workers](https://www.documentcloud.org/documents/6817901-FINAL-WA-Essential-Critical-Infrastructure.html) - [Littler Article](https://www.littler.com/publication-press/publication/washington-issues-stay-home-stay-healthy-order-strictly-limiting) - [Proclamation 20-25.3](https://www.governor.wa.gov/sites/default/files/20-25.3%20-%20COVID-19%20Stay%20Home%20Stay%20Healthy%20-%20Reopening%20%28tmp%29.pdf?utm_medium=email&utm_source=govdelivery) (Adjusting and Extending Stay Home-Stay Healthy Order) |
| West Virginia | March 24, 2020 at 8:00 p.m. | - Until terminated - Phased opening as of April 30 | - [Executive Order No.](https://apps.sos.wv.gov/adlaw/executivejournal/readpdf.aspx?DocID=89504) [9-20](https://apps.sos.wv.gov/adlaw/executivejournal/readpdf.aspx?DocID=89504) - [Stay at Home Page](https://dhhr.wv.gov/COVID-19/Pages/Governor-Issues-Stay-at-Home-Order.aspx) |
| Wisconsin | March 25, 2020 at 8:00 a.m. | - May 26, 2020 at 8:00 a.m. - Phased opening as of April 29 - ***On May 13, the*** [***Wisconsin Supreme Court struck down most of the safer at home order***](https://www.littler.com/publication-press/publication/wisconsin-supreme-court-strikes-down-covid-19-safer-home-order)***.*** *Numerous localities have adopted safer at home orders of their own, including, for example, the* [*City of Milwaukee*](https://city.milwaukee.gov/Order1MovingMilwaukeeForward)*.* | - [Executive Order #12](https://content.govdelivery.com/attachments/WIGOV/2020/03/24/file_attachments/1409408/Health%20Order%20%2312%20Safer%20At%20Home.pdf) - [Emergency Order #28](https://content.govdelivery.com/attachments/WIGOV/2020/04/16/file_attachments/1428995/EMO28-SaferAtHome.pdf) (extension) - [Press Release](https://content.govdelivery.com/accounts/WIGOV/bulletins/286d626) (extension) - [Guidance Page](https://wedc.org/essentialbusiness/) - Updated [FAQs](https://content.govdelivery.com/attachments/WIGOV/2020/04/16/file_attachments/1428997/2020-04-16%20Safer%20at%20Home%20extension%20FAQ.pdf) |

# Appendix C: Different Modalities of Broadband Access

A more comprehensive description of the various modalities considered comes paraphrased from FCC documentation:^16^

- Wireless technologies using longer-range directional equipment provide broadband service in remote or sparsely populated areas where digital subscriber line (DSL) or cable modem service would be costly to provide. Speeds are generally comparable to DSL and cable modem. An external antenna is usually required to use such technologies.
  - Fixed Wireless networks allow consumers to access the Internet from a fixed point while stationary and often require a direct line-of-sight between the wireless transmitter and receiver. This form of connectivity is offered using both licensed spectrum and unlicensed devices. For example, thousands of small Wireless Internet Services Providers (WISPs) provide such wireless broadband at speeds of around one Mbps using unlicensed devices, often in rural areas not served by cable or wireline broadband networks.
  - Wireless Local Area Networks (WLANs) provide wireless broadband access over shorter distances and are often used to extend the reach of a "last-mile" wireline or fixed wireless broadband connection within a home, building, or campus environment. Wi-Fi networks use unlicensed devices and can be designed for private access within a home or business or can be used for public Internet access at "hot spots" such as restaurants, coffee shops, hotels, airports, convention centers, and city parks.
  - Mobile wireless broadband services are becoming available from mobile telephone service providers and others. These services are generally appropriate for highly mobile customers and require a special PC card with a built-in antenna that plugs into a user’s laptop computer. Generally, they provide lower speeds (several hundred Kbps).
- Satellite is another form of wireless broadband that is especially useful for serving remote or sparsely populated areas. Downstream and upstream speeds for satellite broadband depend on several factors, including the provider and service package purchased, the consumer’s line of sight to the orbiting satellite, and the weather. Usually, one can expect to download at a speed of about 500 Kbps and upload at a speed of about 80 Kbps. These speeds may be slower than DSL and cable modem, but they are about 10 times faster than the download speed with dial-up Internet access. Service can be disrupted in extreme weather conditions.
- Cable uses the same coaxial cables that deliver pictures and sound to your TV set so that cable operators can provide broadband through existing services. Modems are usually external devices that have two connections: one to the cable wall outlet, the other to a computer. Transmission speeds of 1.5 Mbps or more. Subscribers can access cable modem service by turning on their computers (without dialing-up an ISP (Internet Service Provider)). Users can still use other services while accessing broadband, such as watching cable TV. Transmission speeds vary depending on the type of cable modem, cable network, and traffic load. Despite such variation, speeds are comparable to DSL.
- DSL uses a wireline technology to transmit data faster over traditional copper telephone lines usually installed to homes and businesses. Transmission speeds range from several hundred Kbps to millions of bits per second (Mbps). Availability and speed may depend on distance from home/business to closest telephone company facility.
  - This also includes Asymmetrical Digital Subscriber Line (ADSL) – Primarily residential customers who receive ample data but do not send much (such as Internet surfers). This is usually faster in the downstream direction than the upstream direction.
  - Symmetrical Digital Subscriber Line (SDSL) – Primarily by businesses for services such as video conferencing that need significant bandwidth both upstream and downstream.
- Fiber converts electrical signals carrying data to light and sends the light through tiny transparent glass fibers (diameter of a human hair). This form transmits data at speeds far exceeding current DSL or cable modem speeds, typically by tens or even hundreds of Mbps. Actual speed will vary depending on how close to your computer the service provider brings the fiber and how the service provider configures the service, including the amount of bandwidth used. The same fiber providing your broadband can also simultaneously deliver voice (VoIP) and video services, including video-on-demand. Telecommunications providers sometimes offer fiber broadband in limited areas, though many have announced plans to expand their fiber networks and offer bundled voice, Internet access, and video services. Variations of the technology run the fiber all the way to the customer’s home or business, to the curb outside, or to a location somewhere between the provider’s facilities and the customer, and the access modes can either be wireless or wired. The Table below summarizes these different forms and which forms are captured by which broadband datasets used in this analysis.
- Broadband over Powerline (BPL): BPL is the delivery of broadband over the existing low- and medium-voltage electric power distribution network. BPL speeds are comparable to DSL and cable modem speeds. BPL can be provided to homes using existing electrical connections and outlets. BPL is an emerging technology that is available in very limited areas. It has significant potential because power lines are installed virtually everywhere, alleviating the need to build new broadband facilities for every customer


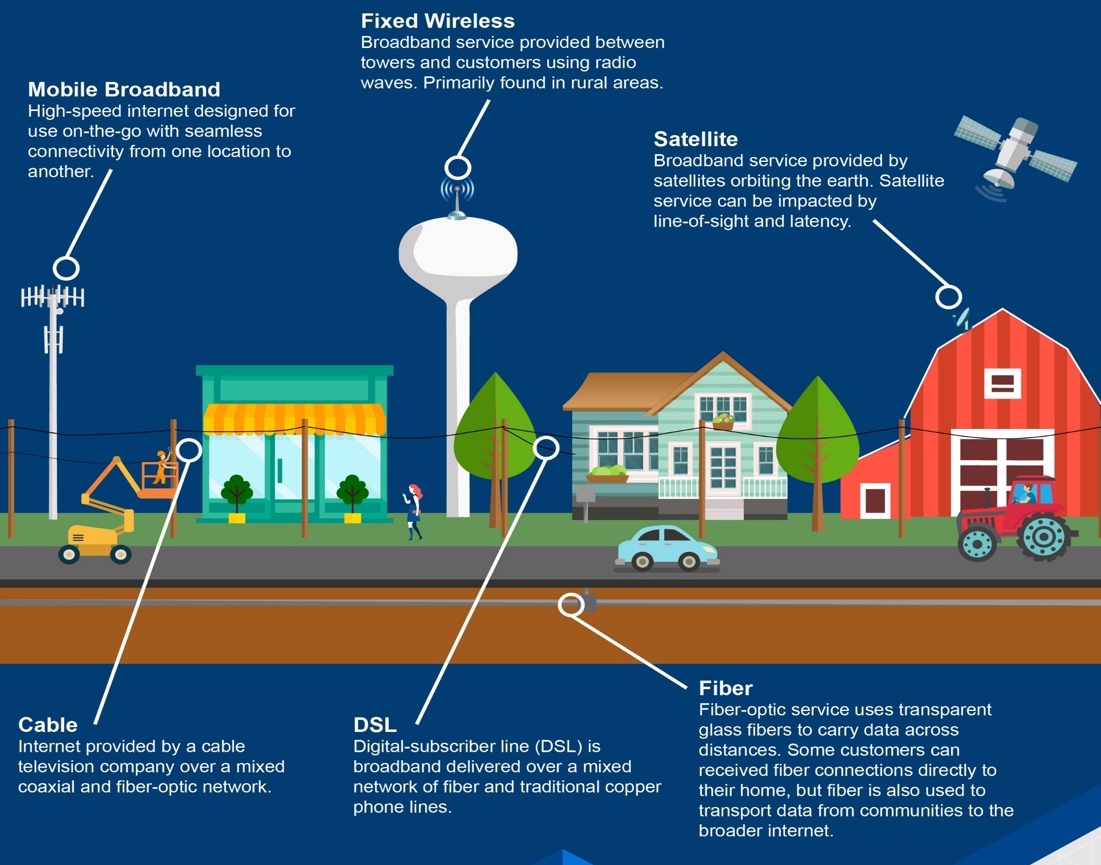
The following figure also helps visualize where these different forms of broadband operate:^17^

**Figure C1**: Summarizing the different kinds of broadband internet

Table C1 provides an overview of how each different broadband dataset considers or incorporates the various types of broadband provision.

**Table C1:** Broadband Data Type by Dataset

|  | **What it measures** | **Direct Subscriber Lines (DSL)** | **Satellite** | **Cable modems** | **Fixed Wireless** | **Fiber optics** |
| --- | --- | --- | --- | --- | --- | --- |
| Definition |  | Transmits data to homes and businesses over traditional copper telephone lines | Transmits data via geospatially orbiting satellites | Transmit data through coaxial cables that generate pictures and sounds on TV sets | Transmission of data from a local antenna to a permanent location such as a home or business. | Convert electrical signals carrying data into light and send the light through transparent glass fibers. |
| MSFT | Every time a device receives an update or connects to a MSFT service, a measurement is taken of throughput speed, package size sent, and total download time. County aggregate measure is based upon the number of devices that have connected at adequate broadband speed. | Yes – as long as above 25/3 | Yes – as long as above 25/3 | Yes – as long as above 25/3 | Yes – as long as above 25/3 | Yes – as long as above 25/3 |
| ACS | Self-assessment access to internet or not, aggregated up to county level. | Yes | Yes – but separate item from broadband | Yes | Not explicitly mentioned | Yes |
| FCC – County level data provided by the annual report | Maximum advertised speeds as self-reported by service providers, aggregated up to county level. | Yes | Yes | Yes | Yes | Yes |
| Ookla | Multi-stream Transmission Control Protocol ((TCP) which facilitate the delivery of data; multi-stream typically allows for higher through put than single streams do) measurement estimating link capacity, aggregated at county and census tract level | Yes | Yes | Yes | Yes | Yes |
| MLab | Single stream TCP measurement of Bulk Transport Capacity, aggregated at county level. | Yes | Yes | Yes | Yes | Yes |

# Appendix D: Basic broadband speeds under generic use conditions^18^


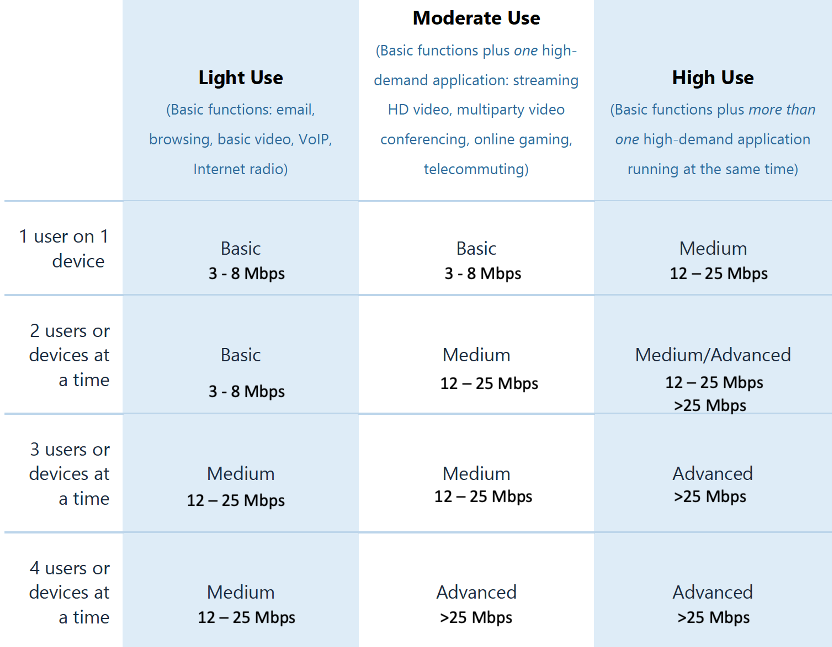


**Figure D1:** Basic broadband speeds under generic use conditions

# Appendix E: Technical Details for Each Broadband Measurement Approach

Table E1 shows a more technical discussion on how each dataset is measured and collected. The responses for FCC, M-Lab and Ookla data are all provided directly by the authors of The National Telecommunications and Information Administration’s (NTIA) Indicators of Broadband Need^19^.

**Table E1**: Technical Details for Each Broadband Measurement Approach

|  | **FCC Form 477**^19^ | **M-Lab NDT**^19^ | **Ookla Speedtest**^19^ | **ACS Census** | **Microsoft** |
| --- | --- | --- | --- | --- | --- |
| **Server Location** | N/A (self-reported from corporation) | Off-net, tests conducted to the closest server geographically | On-net, tests conducted to servers with least latency (time delay between sender and receiver). | N/A (self-reported by individual) |  |
| **Servers Used per Test** | N/A | 1 | 4+ | N/A |  |
| **# TCP Streams** | N/A | 1 | 4+ | N/A |  |
| **Measurement Specification/Description** | N/A | Single stream TCP measure of Bulk Transport Capacity. Measures the speed the user’s device is getting from the router. | Multi-stream TCP measure estimating link capacity. Measures the speed the user’s router device is getting from their ISP. | N/A |  |
| **Geographic Precision** | Census Block | Aggregate by any geography, though smaller than county or city not recommended. | Provide Shapefile and Apache Parquet map tiles, aggregated in a grid of ~610.8m^2^ tiles which can be used down to census tract level | Census tract | County |
| **Metrics** | ISP reports maximum upload and download link capacity by access type (fiber, cable, dsl satellite, etc.). | Download & upload speed, latency. | Per tile weighted average of download/upload speeds, average latency, # tests, # devices. | Individual reported access. | Percentage of population with 25/3 Download and upload speed |
| **Mobile vs. Fixed** | Measured separately | Combined | Combined | Combined | Combined |
| **Provider Information** | Provider Name, “Doing business as” Name, Holding Company Name | ASN - Autonomous System Number/Name | Not provided | N/A | N/A |
| **Data Collection/Access** | ISP-contributed, free and open access to aggregate data. Collection method left to the ISP. | User-contributed, free and open access to individual data points. Open source server run by M-Lab. | User-contributed, free and open access to aggregate data. Closed source server that is available for others to run. | Census workers collect from individual households. | User-contributed whenever user utilizes Microsoft product. Free and open to access. |

# Appendix F: Exploratory Analysis for Regression Statistics


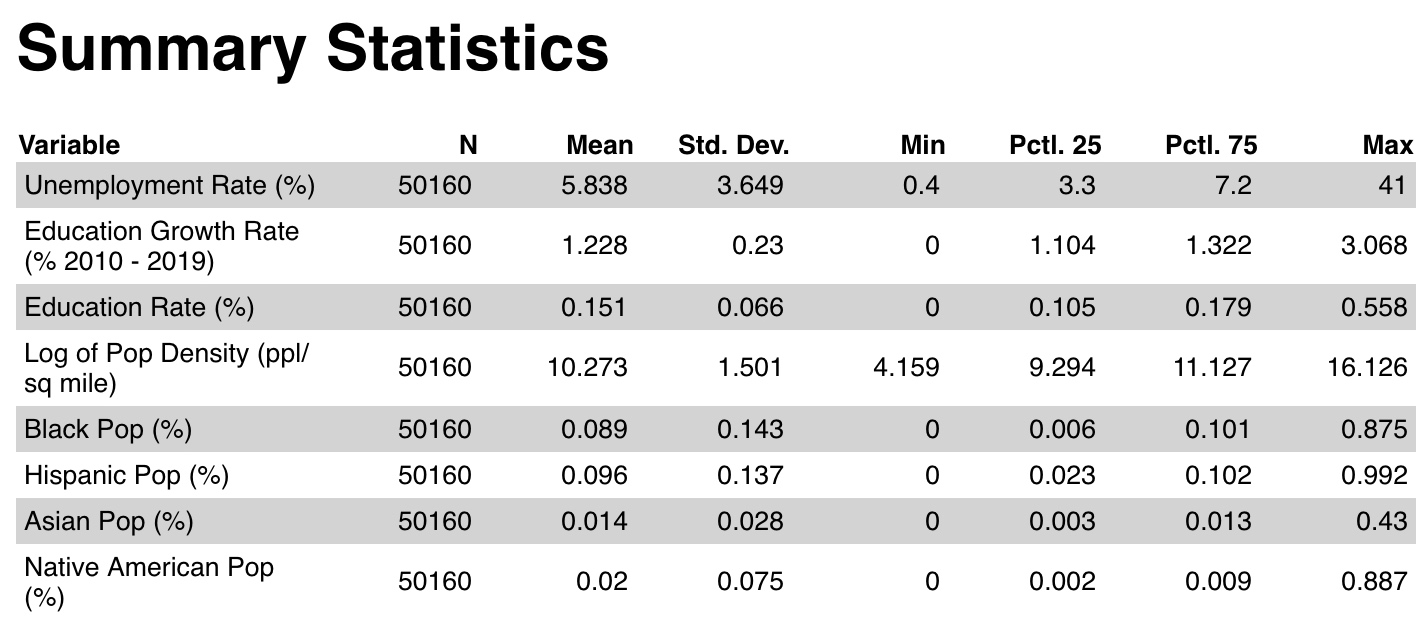


**Figure F1**: Summary statistics table


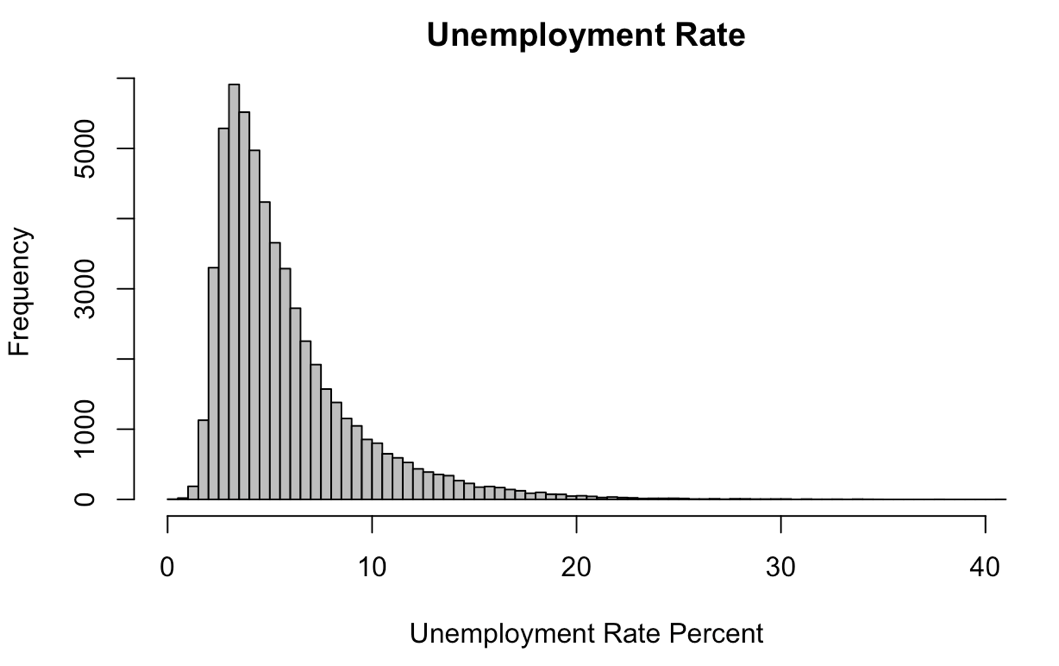
We conduct a check on the distribution of our dependent variable, percent unemployment rate and find that the variable is skewed left. To assess if this impacts our results, we take the log of the unemployment rate percent and find that this creates a more normal distribution in the variable, as reflected in histograms (Figures F2-F3), Q-Q plots (Figures F4-F5), residual plots and (Figures F6-F7) Q-Q plots of the residuals (F8 – F9).

**Figure F2**: Histogram of Unemployment Rate


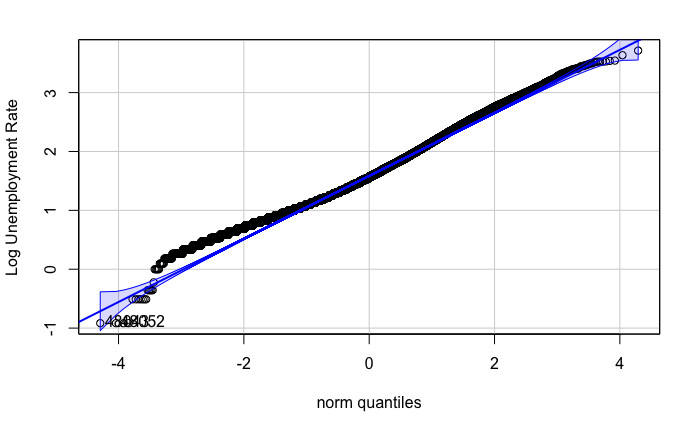

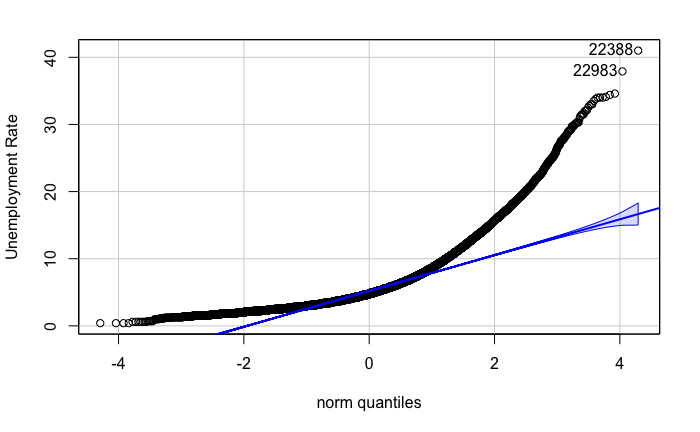

**Figure F3**: Histogram of Log of Unemployment Rate
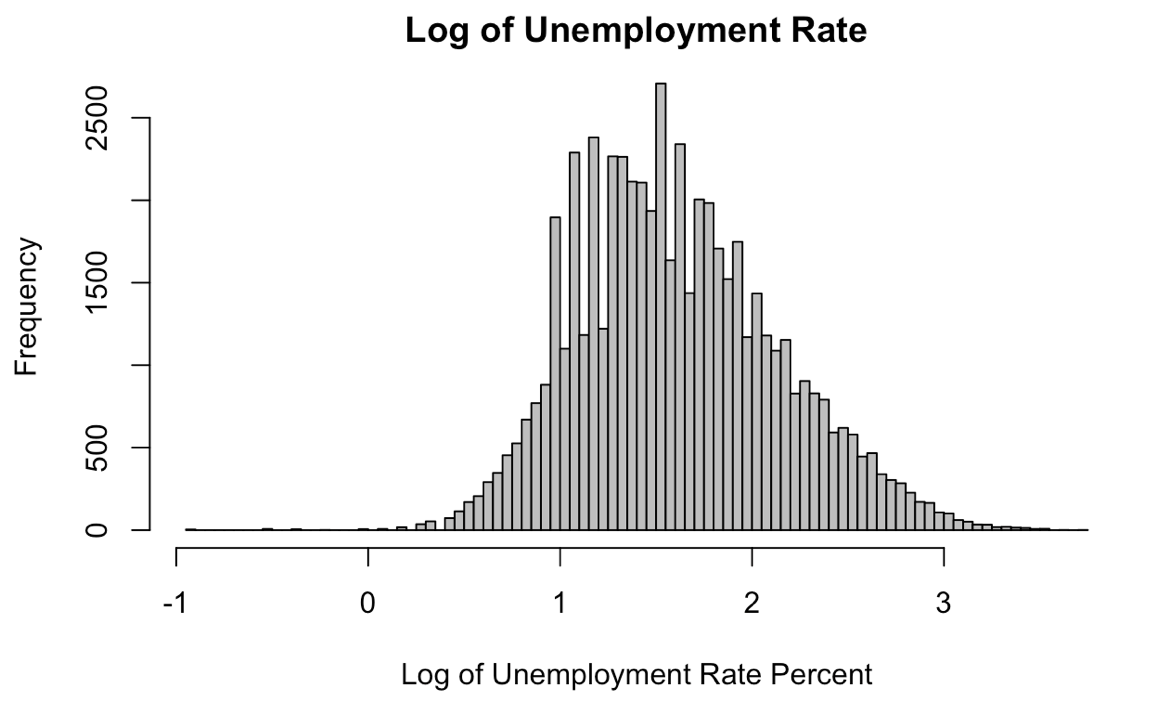


**Figure F4:** Q-Q Plot of Unemployment Rate. We see here that the variable is not distributed normally.
**Figure F5:** Q-Q Plot of the log of Unemployment Rate. We see here that the logged value becomes normally distributed.


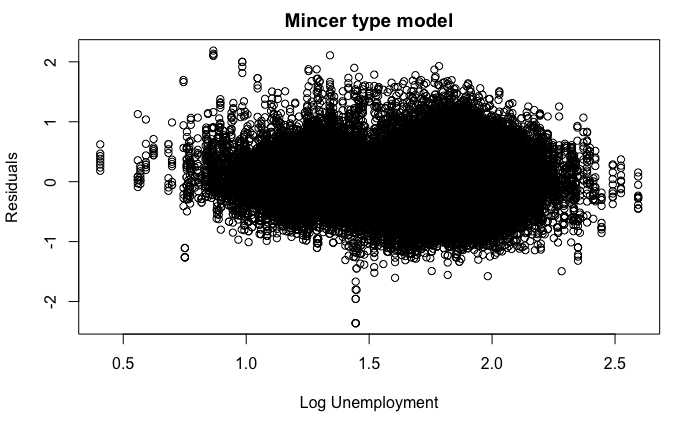

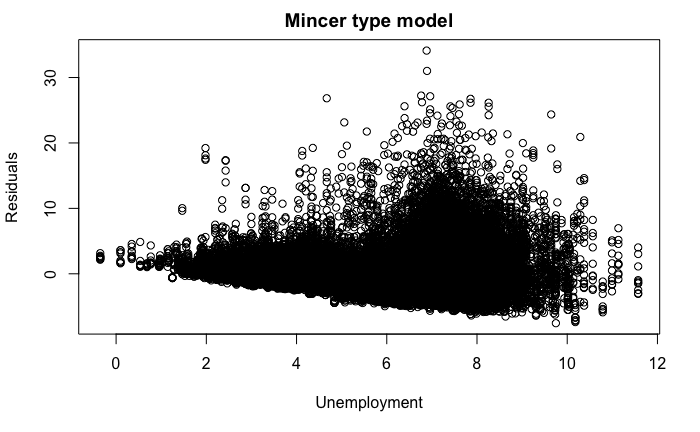


**Figure F6:** Mincer model plot of the residual spread for the base case analysis. We can see here that the residuals tend to cluster and pattern linearly with the non-logged version of unemployment.
**Figure F7:** Mincer model plot of the residual spread for the base case analysis using the logged version of unemployment. We see here a more random distribution of the residuals.


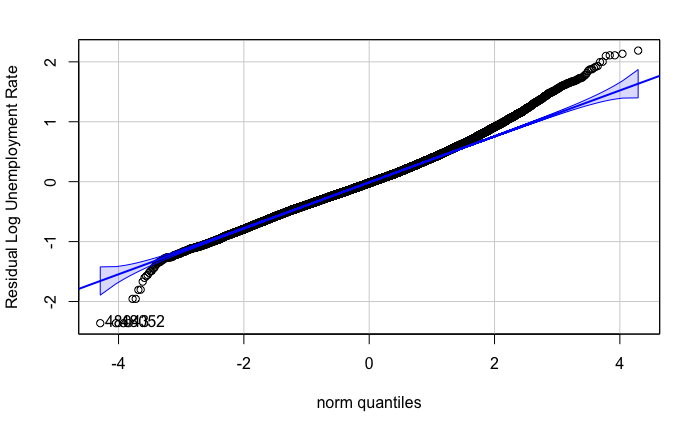

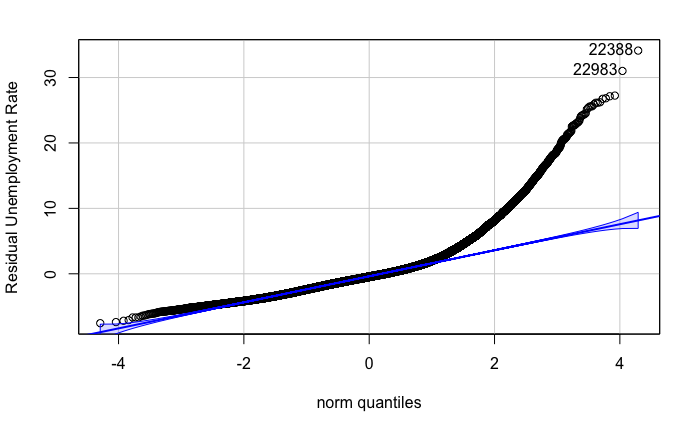


**Figure F8:** Q-Q Plot of base case regression residuals. We see here that the residuals are not distributed normally.
**Figure F9:** Q-Q Plot of base case regression residuals. We see here that the residuals are now distributed more normally.

We run our base case analysis to ensure that our results hold consistently across scenarios. We display here the base regression run for the log of the dependent variable and show that the results are robust and consistent. We see from the table below that in the base case, the change in the log of unemployment rate for 50% access is 0.23. In order to convert this to a percent change of unemployment rate, we use exp(0.23) - 1)*100% to find a total of 25% increase in unemployment. Given that the avg unemployment rate for full sample (including treat and control) is 5.78, and in our base case when using the standard unemployment rate we see an increase of 1.34, this gives us a percent change in the unemployment rate of (5.78+1.34)/5.78 ≈ 23%. So if anything, in using the standard unemployment rate in our calculations above, this is actually a slight underestimate.

**Table F1:** MSFT 2020 – Log of Unemployment Rate


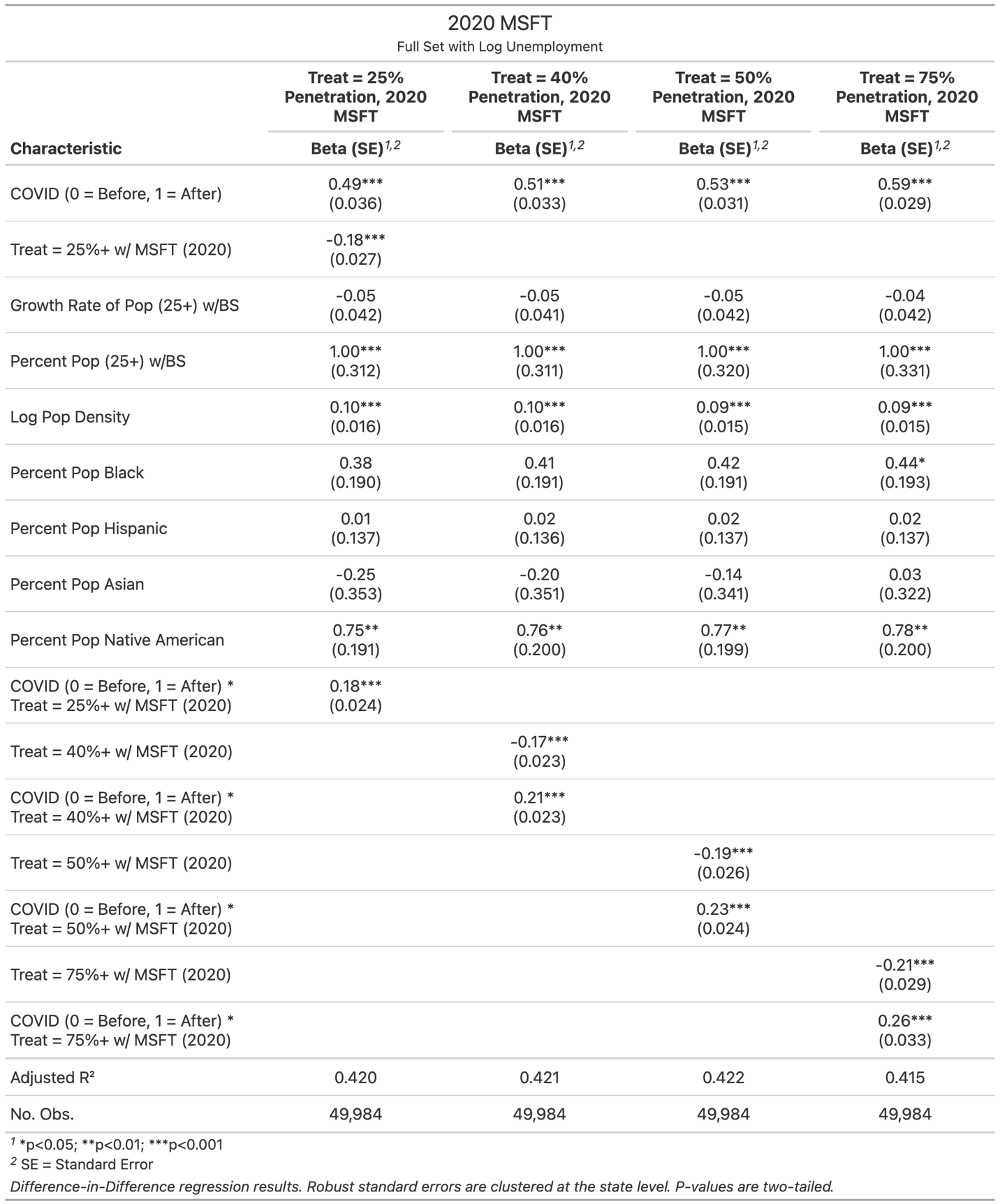


In addition to exploring the statistics for the entire data set, we also inspected correlation between all relevant variables, as is presented in Figure F10.


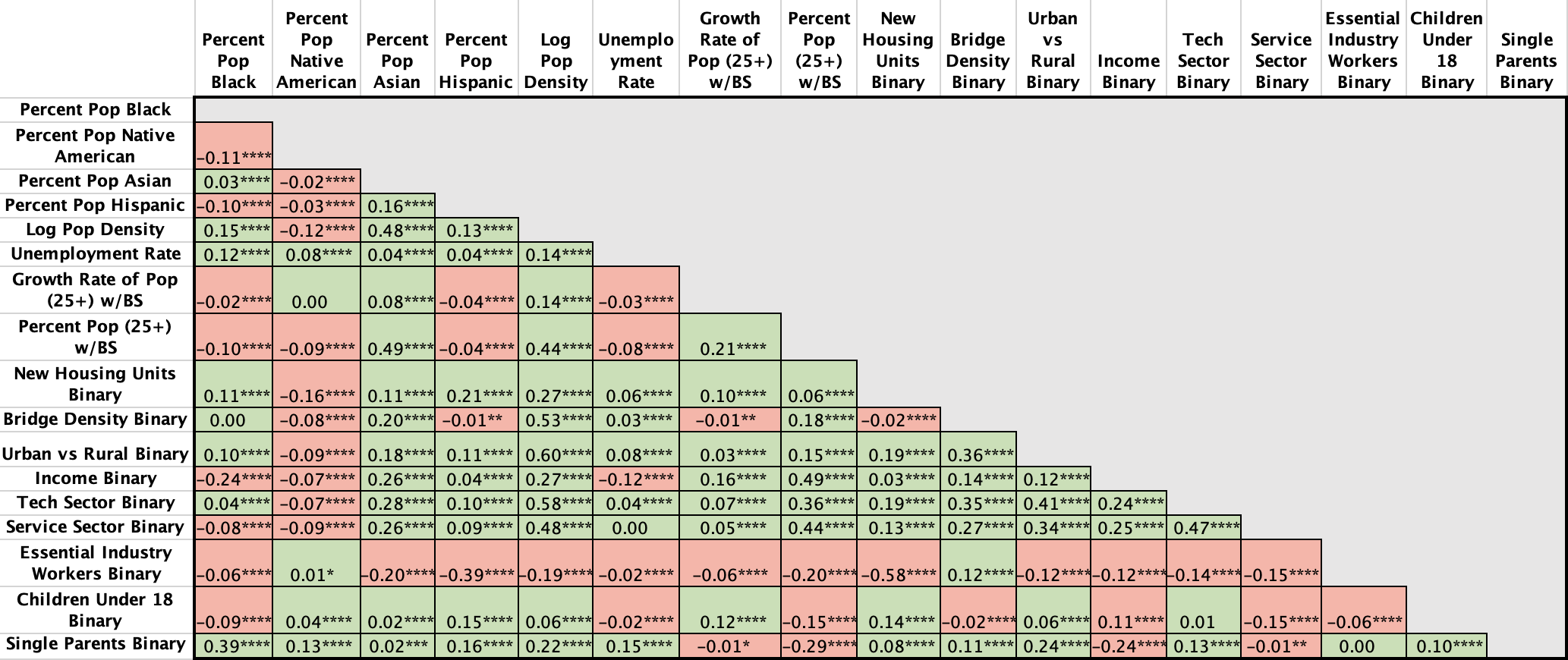
**** p < 0.0001; *** p < 0.001; ** p < 0.01; * p < 0.05 (two-tailed)
Green is a positive value; Red is a negative value.

**Figure F10:** Correlation matrix between all key variables.

Figure F11 shows an overview of how different metrics of economic health responded after the COVID-19 work from home mandates.


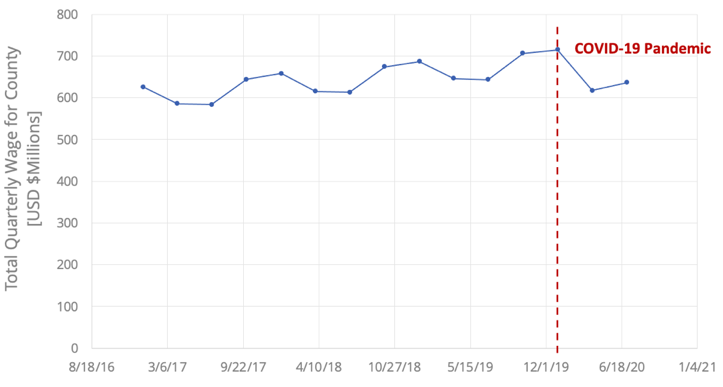


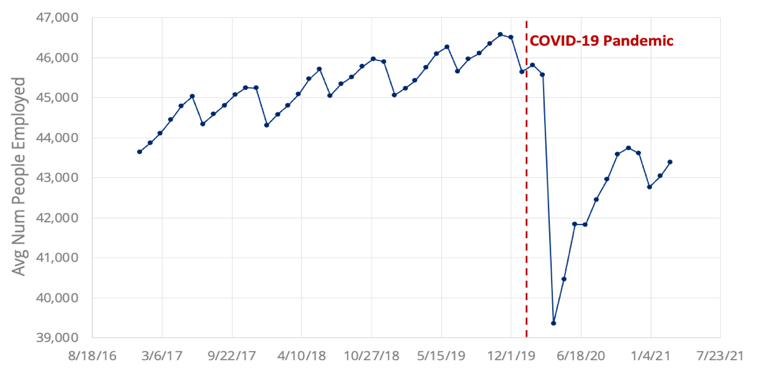


**Figure F11:** Top: This graph shows how the quarterly wages, at a county level, were impacted during the COVID-19 pandemic stay-at-home mandates. We see the impacts of seasonality, but not observable impacts from the COVID-19 pandemic. Bottom: This graph shows how the employment rate responded to the COVID-19 stay-at-home mandates. Here we can see that employment was significantly impacted right after the start of the stay-at-home mandates.

# Appendix G: Robustness Checks

To confirm the directionality of the results across different assumptions and datasets, we also run a large series of various robustness checks which lead to either robust or consistent results. The first, and arguably most important of these checks is to include a variety of fixed effects to ensure that the observed effect is not due to a time-unvarying effect. Here we present the results of our model incorporating fixed effects at the state geographic level and the monthly temporal level. State level fixed-effects are used rather than county level fixed-effects because work from home policies are made at the state level (rather than the county level) so this is the resolution of relevance for applying fixed effects. We do however include the county fixed effect analysis in case this is of particular interest and find the results also hold robust and consistent.

**Table G1:** Results of the main model including Fixed Effects at the State and Month. Robust standard errors, clustered at the state level, are included below each estimate with statistical significance indicated by the stars based off of a two-tailed p-test. Here we see that when all else equal, after COVID, counties with more than 50% access to 25 Mbps download and 3 Mbps upload as measured by MSFT 2020 with fixed effects included at the state and month level, experience an increase of 1.35% in their unemployment rates, respectively, over similar counties that have less than 50% access. This is statistically consistent and robust with the findings of our main results in the paper.

*
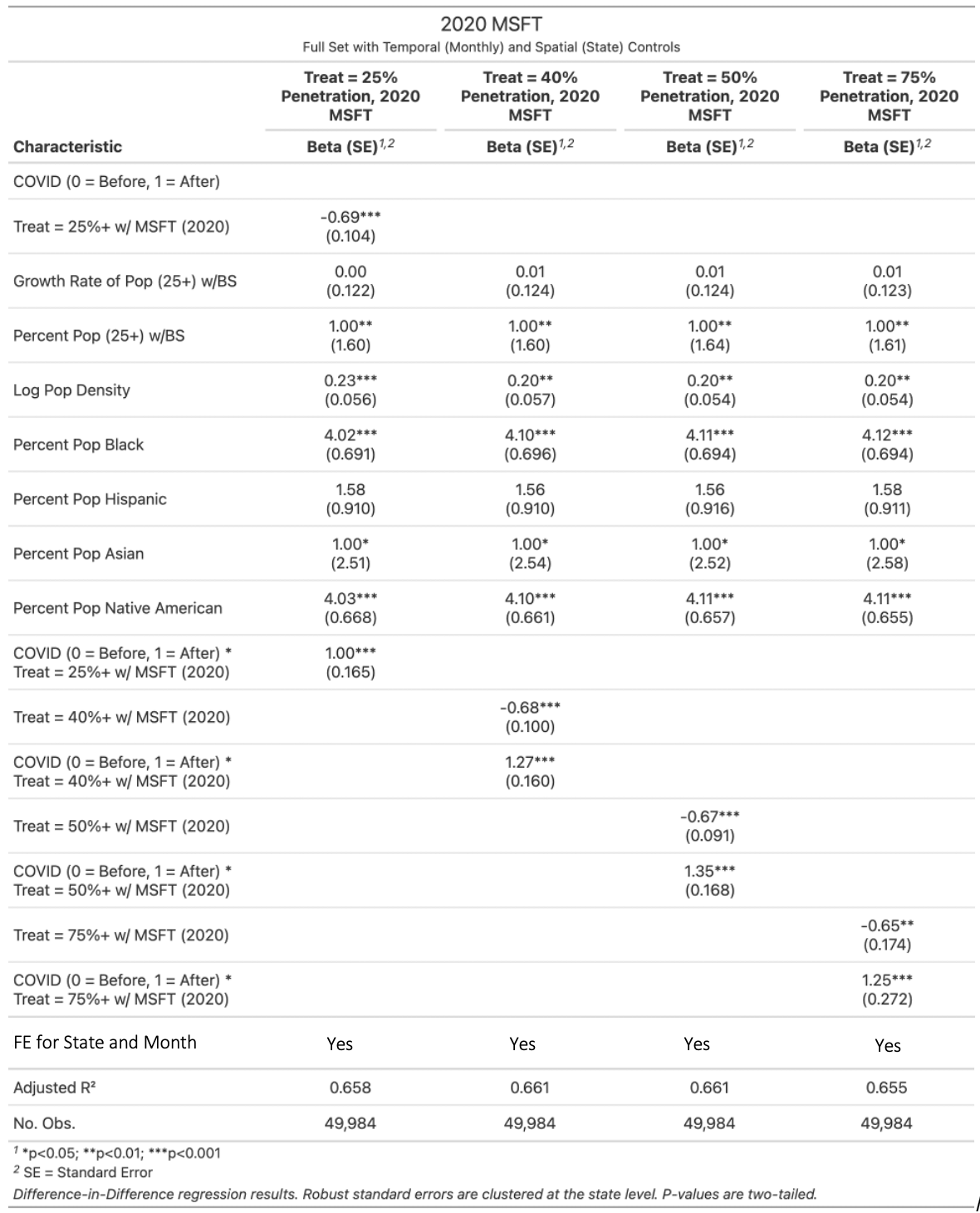
*

The second set of checks we assess is the incorporation of other potential controls. In addition to the controls included in our base model, we also include controls used by others in prior work, namely considering the number of people employed in other industries, as classified by NAICS 2-code specifications ^20^. The results from this analysis are presented below.

**Table G2:** Results of the main model with additional controls included for other forms of industry. Robust standard errors, clustered at the state level, are included below each estimate with statistical significance indicated by the stars based off of a two-tailed p-test. Here we see that when all else equal, after COVID, counties with more than 50% access to 25 Mbps download and 3 Mbps upload as measured by Microsoft, experience an increase of 0.97% in their unemployment rates, respectively, over similar counties that have less than 50% access. This is statistically consistent and robust with the findings of our main results in the paper.


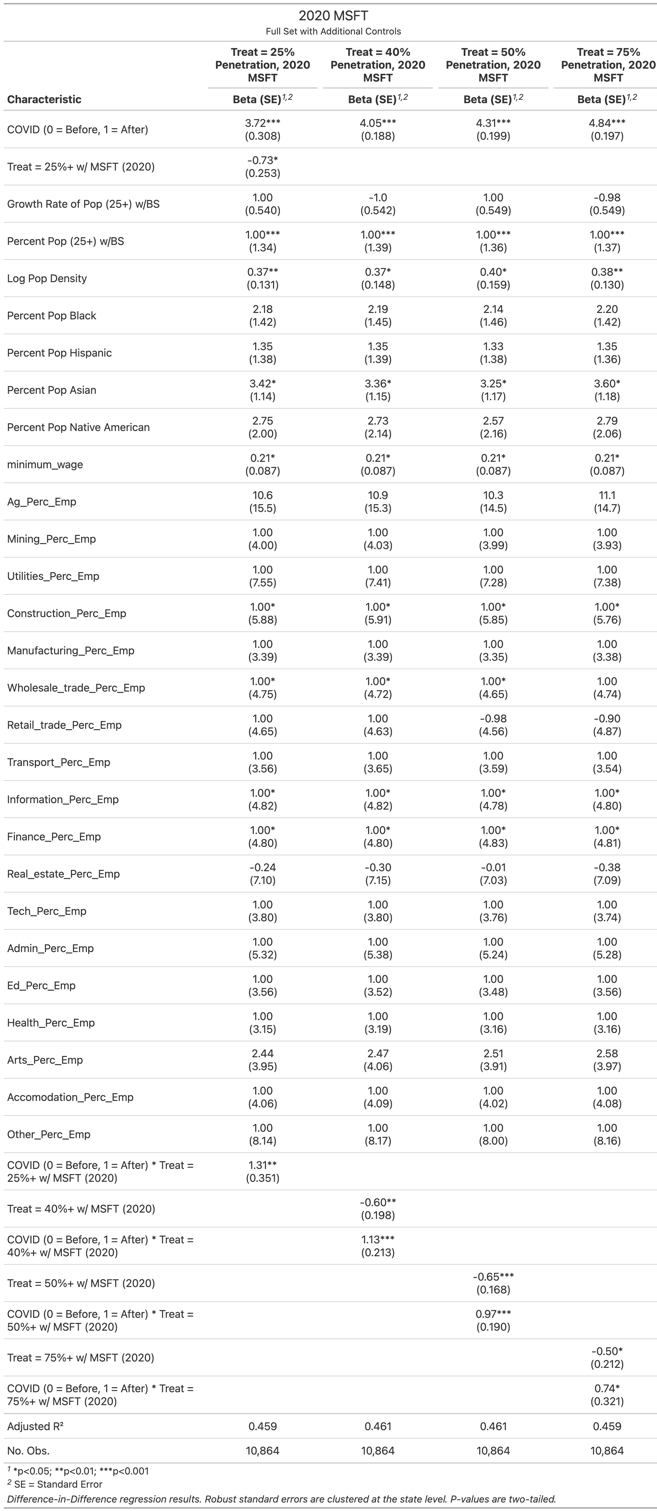


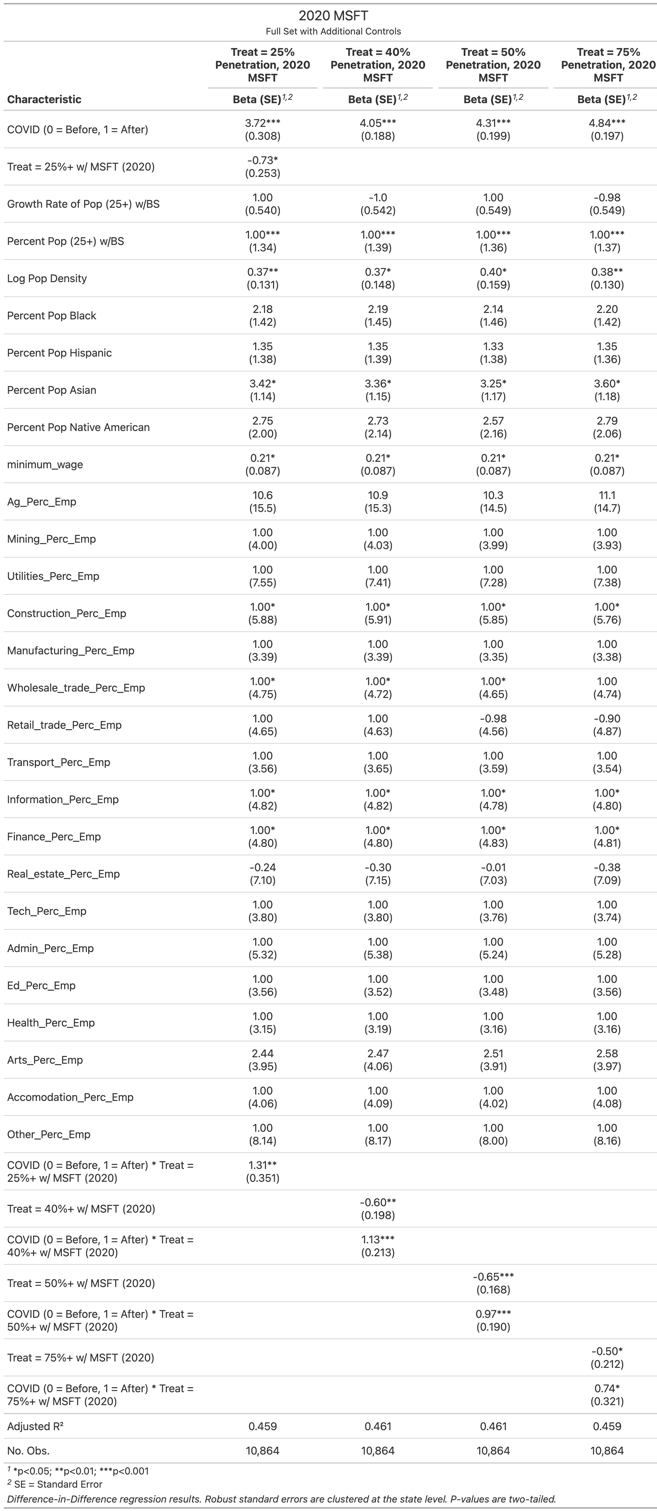


The third set of checks we assess is controlling for the spread of the COVID pandemic at the county level. We believe that infection rates should not influence the creation of the treatment and control groups as broadband penetration rates are unrelated to the spread of COVID. We see this, in part, to be one of the main strengths of this research design as the creation of the treatment and control groups are separate from the pandemic itself. However, there could be worry of how it could intervene in via another reform that manifested itself also during the pandemic, namely the Emergency Broadband Benefit program which provided households with a supplemental income of $50/month to help pay for increased broadband access. As such perhaps, infection rate could drive how many take advantage this program. We register the concern around controlling for infection rates in the rest of the model and have incorporated a control for that in our robustness checks.

**Table G3:** Results of the main model with additional controls for COVID case load. Robust standard errors, clustered at the state level, are included below each estimate with statistical significance indicated by the stars based off of a two-tailed p-test. Here we see that when all else equal, after COVID, counties with more than 50% access to 25 Mbps download and 3 Mbps upload as measured by Microsoft, experience an increase of 1.28% in their unemployment rates, respectively, over similar counties that have less than 50% access. This is statistically consistent and robust with the findings of our main results in the paper.


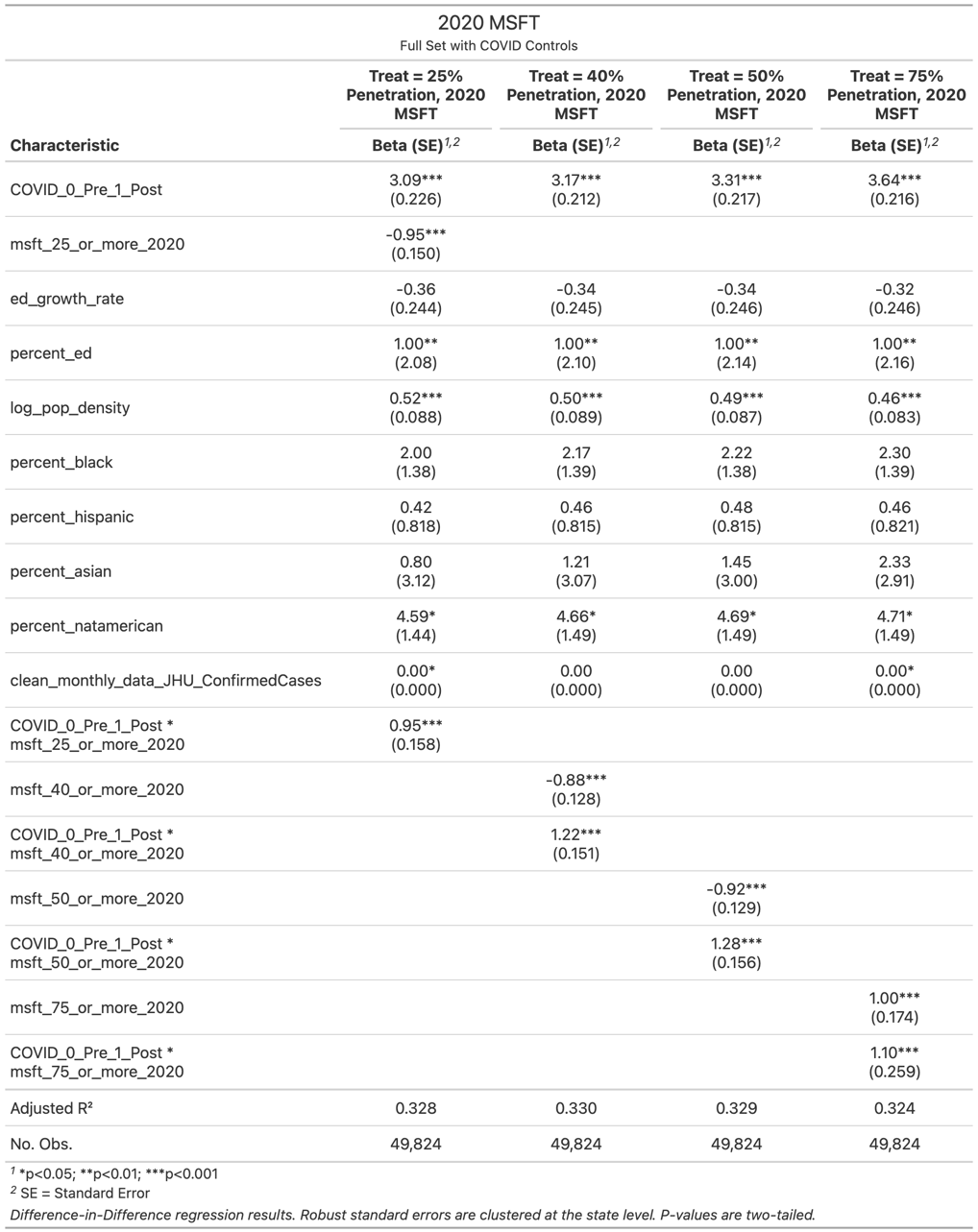


The fourth set of checks we assess is the use of the various datasets of broadband access at a county level. This check has been integrated throughout the analysis given how fundamental the critiques are across the different datasets and given the discussion around what each dataset is measuring (e.g., actual, advertised, or self-reported broadband speed and access). As is presented above, while there are differences in the magnitude, the findings from the FCC and MSFT dataset largely hold across the major findings. In the case that the directionality differs, in all cases, it is slight. In most case, there is also statistical significance for the FCC and MSFT data, with MSFT usually higher in magnitude than FCC. The ACS data seems to exhibit less statistical significance across most key results. In totality, this suggests actual (as opposed to advertised) broadband quality (rather than mere access) for enhancing the precision of broadband as a social sensor (in this case for unemployment).

The fifth critical robustness check, also included throughout, is parametrizing what percentage of the population has access to 25 Mbps download and 3 Mbps of upload speed. As is presented in all of the results tables, the scenarios of 25%, 40%, 50% and 75% of the population, at a county level, with access to adequate levels of broadband have been analyzed. It is interesting to note that the impact on wages seems to increase monotonically across most scenarios, up until the 50% penetration point, at which the impact on unemployment rates usually tends to remain constant or decrease. This aligns with prior work in this area which finds argues that in order to achieve adequate economic impact, digital infrastructure must reach at least 50% of the population.^21^

In addition to the aforementioned robustness checks that have been integrated throughout, several other critical checks have also been run. The sixth check was focused on assessing a critical assumption of difference-in-difference analysis which states that treatment and control groups must remain constant over time. Unfortunately, due to the data availability of both the FCC and ACS data, only the 2019 sets of access are currently available. However, there is data available for both the 2019 and 2020 for the MSFT datasets. In this robustness check, only the counties that remain in the same treatment or control groups are included in the analysis and as seen in Table G1 the magnitude of the findings, under the base case, are higher in this case. This suggests that we are actually likely underestimating the potential effect of broadband access on unemployment rates.

**Table G4:** Difference in difference estimators for counties that remain in the same treatment or control groups over the transition from 2019 to 2020 MSFT data. Robust standard errors, clustered at the state level, are included below each estimate with statistical significance indicated by the stars. Here we see that with the current state of the research, in our base case (MSFT data, 50% penetration) when all else equal, after COVID, counties with more than 50% access to 25 Mbps download and 3 Mbps upload, experience an increase of 1.7% in their unemployment rate over similar counties that have less than 50% access.

|  | In Treatment Diff-in-Diff Estimator | | | |
| --- | --- | --- | --- | --- |
|  | >25% | >40% | >50% | >75% |
| In treatment MSFT 2019 to MSFT 2020 | 1.31*** | 1.67*** | 1.79*** | 1.64** |
|  | (0.192) | (0.064) | (0.279) | (0.450) |

*** p < 0.001; ** p < 0.01; * p < 0.05 (two-tailed)
Darker green is a higher value, darker red is a lower value

The seventh check is assessing if shifting the periodicity of the COVID-19 shock impacts the findings of the results. As is seen in Appendix A: COVID-19 State Work from Home Policies, 34 of the 51 states (including Washington DC) implement their stay-at-home policies at the end of March. The remaining 16 states all implement stay at home orders within the first 7 days of April. In order to maintain the highest level of data resolution that the unemployment numbers are published at, the initial difference-in-differences regressions are run with a temporal timeframe of months. In order to ensure that the shock is being represented accurately, we shift the shock of COVID to happening in April in order to account for any slight variation which might not be captured by keeping the shock in the month of March. As can be seen in Table G5, the results across these two scenarios also hold consistent.

**Table G5**: Difference in difference estimators, allowing the shock of COVID to occur in April rather than in March (i.e., *COVID* variable in main models goes from April onwards instead of March onwards). Robust standard errors, clustered at the state level, are included below each estimate with statistical significance indicated by the stars based off of a two-tailed p-test. Here we see that with the current state of the research, in our base case (MSFT data, 50% penetration) when all else equal, after COVID, counties with more than 50% access to 25 Mbps download and 3 Mbps upload, experience an increase of 1.55% in their unemployment rate over similar counties that have less than 50% access. When we compare this to our full set of data, we see that this is similar and slightly higher, indicating that our main estimates may be underestimating the effect.

|  | Sensitivity to COVID in April Diff-in-Diff Estimator | | | |
| --- | --- | --- | --- | --- |
|  | >25% | >40% | >50% | >75% |
| MSFT 2019 | 1.49*** | 1.69*** | 1.82*** | 1.44* |
|  | (0.215) | (0.236) | (0.302) | (0.450) |
| MSFT 2020 | 1.18*** | 1.47*** | 1.55*** | 1.39*** |
|  | (0.189) | (0.181) | (0.188) | (0.303) |

*** p < 0.001; ** p < 0.01; * p < 0.05 (two-tailed)
Darker green is a higher value, darker red is a lower value

In addition to shifting COVID to occur during April 2020 to show that there is little impact on the results between assuming COVID occurs in March or April, we also run falsification tests which shift when the “shock” of COVID occurs. To do this, we ran placebo regressions where we assumed COVID happened in September 2019 (-6 months from the actual shock) and in August 2020 (+6 months from the actual shock) and we show that the signal grows close to zero across the base case for MSFT in both of these cases. This suggests that the shock observed over the case of COVID is due to COVID and not present prior or post the introduction of the shock. The results from this regression are in Table G3 and Table G4 below.

**Table G6:** Placebo regressions where the shock of COVID is simulated to occur in September 2019 (-6 months from the actual shock). Robust standard errors, clustered at the state level, are included below each estimate with statistical significance indicated by the stars based off of a two-tailed p-test. Here we see that when all else equal, after adjusting the shock to September 2019, there are no observed effects between counties with more than 50% access to 25 Mbps download and 3 Mbps upload speed. This suggests that it is the shock of COVID which is creating the impact on unemployment, rather than a shock spuriously correlated with COVID.


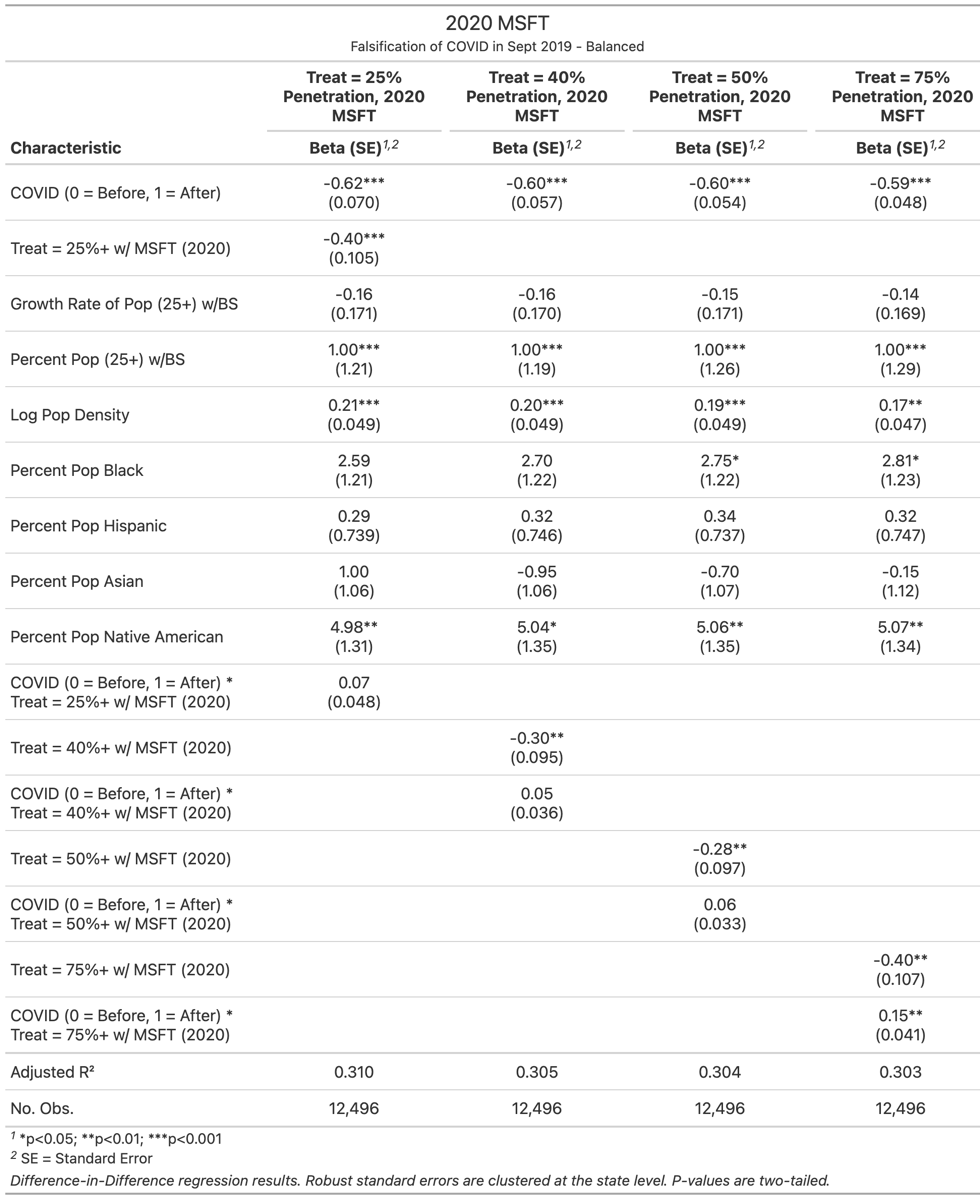


**Table G7:** Placebo regressions where the shock of COVID is simulated to occur in August 2020 (+6 months from the actual shock). Robust standard errors, clustered at the state level, are included below each estimate with statistical significance indicated by the stars based off of a two-tailed p-test. Here we see that when all else equal, after adjusting the shock to August 2020, the observed effects are smaller and negative. This suggests that it is the shock of COVID which is creating the impact on unemployment, rather than a shock spuriously correlated with COVID.


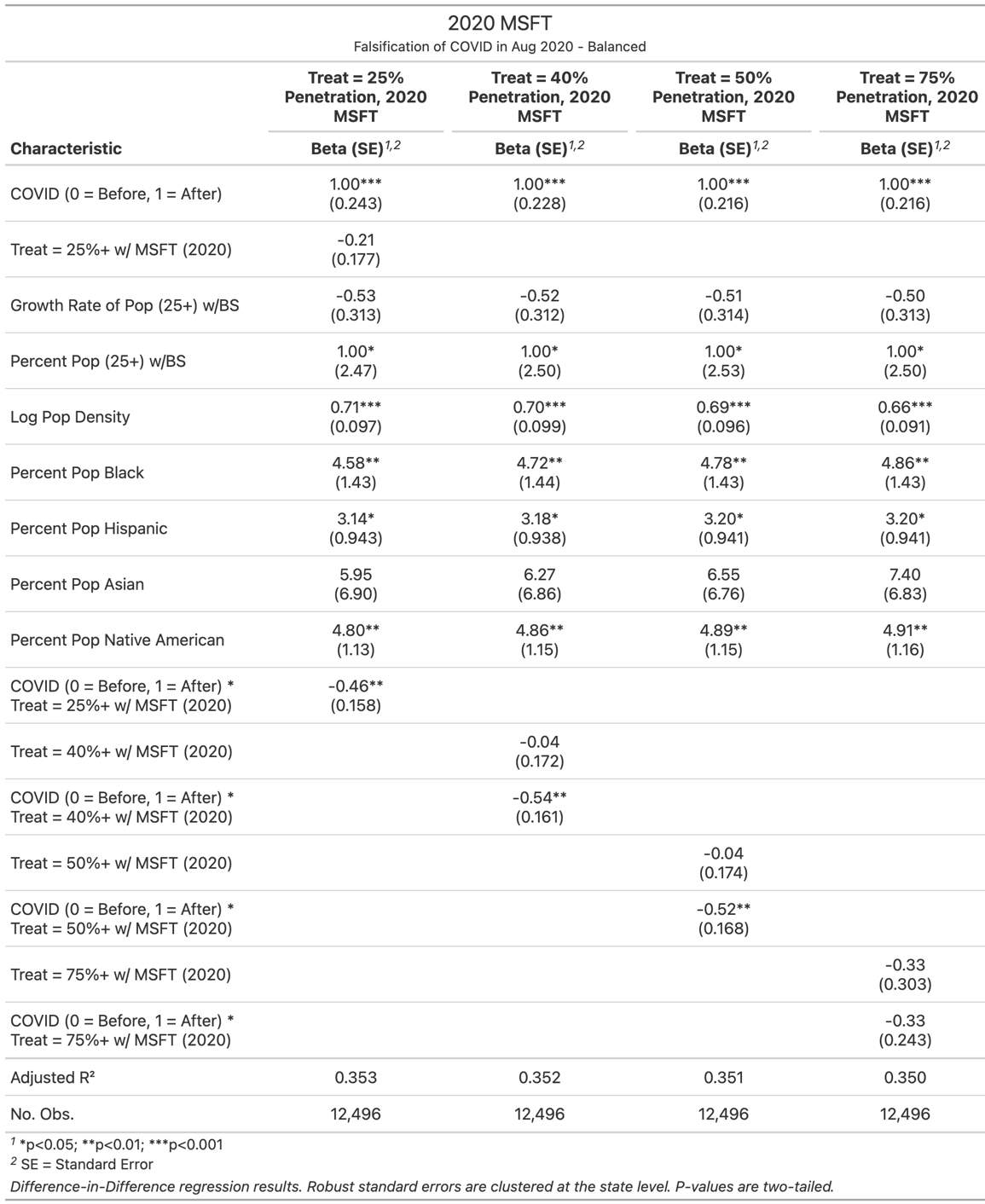


The eight and final robustness check runs the standard regression models shared in the main body of the paper using both Ookla and MLab data as the source of broadband internet data. The Ookla and MLab data both use a 50% penetration rate of 25 Mbps download/3 Mbps upload speed. The results for the full regression models are consistently robust and in alignment with the results found in the main body of the paper.

**Table G8:** 2020 Ookla and MLab Results for the base case full sample. Robust standard errors, clustered at the state level, are included below each estimate with statistical significance indicated by the stars based off of a two-tailed p-test. Here we see that when all else equal, after COVID, counties with more than 50% access to 25 Mbps download and 3 Mbps upload as measured by Ookla and MLab, experience an increase of 1.16% or 0.94% in their unemployment rates, respectively, over similar counties that have less than 50% access. This is statistically consistent and robust with the findings of our main results in the paper.


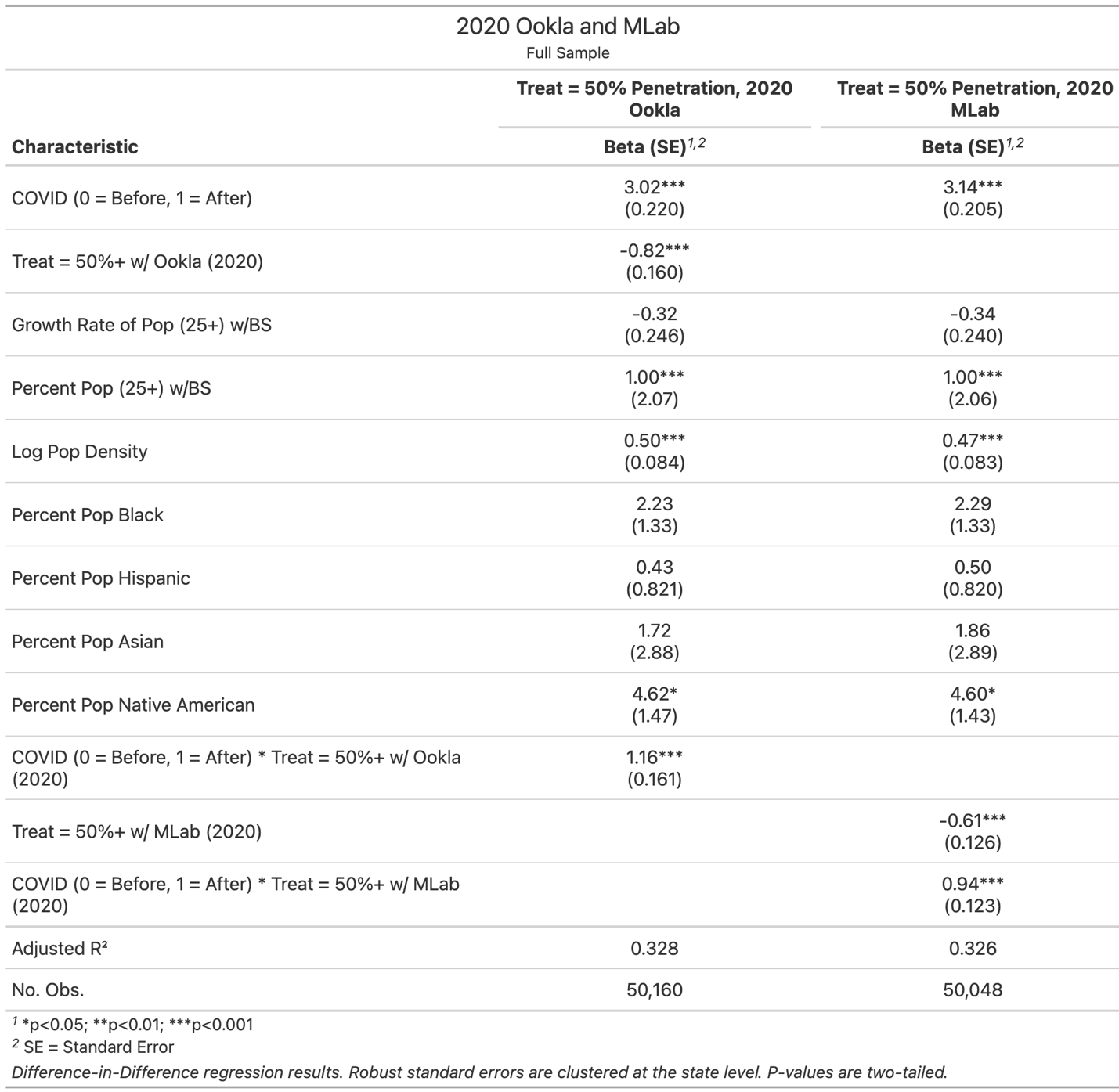


Note, the difference in sample size is due to the difference in data availability from Ookla and MLab.

In addition to this wide supplement of robustness checks that we run, we also provide a set of boundary condition explorations which address concerns on simultaneity due to COVID occurring. In order to assess this, we run a placebo where we defined the treatment and control groups based on above and below median average COVID-19 cases for July 2020 (a representative month for the time frame we considered^86^). While we found some significance for these models, these effects greatly diminish once we incorporate the full set of controls as can be seen in Table G9-G12 below.

**Table G9:** Main base model results, with added splits on high and low COVID cases in July 2020. Robust standard errors, clustered at the state level, are included below each estimate with statistical significance indicated by the stars based off of a two-tailed p-test. Here we see that when all else equal, after COVID, counties with more than 50% access to 25 Mbps download and 3 Mbps upload and in areas that have higher levels of COVID, experience an increase of 1.14% in their unemployment rates over similar counties that have less than 50% access. This is in comparison to counties with more than 50% access to 25 Mbps download and 3 Mbps upload and in areas that have lower levels of COVID, which experience an increase of 0.83% in their unemployment rates. We find these effects greatly diminish once we incorporate the full set of controls as can be seen in Table G10.


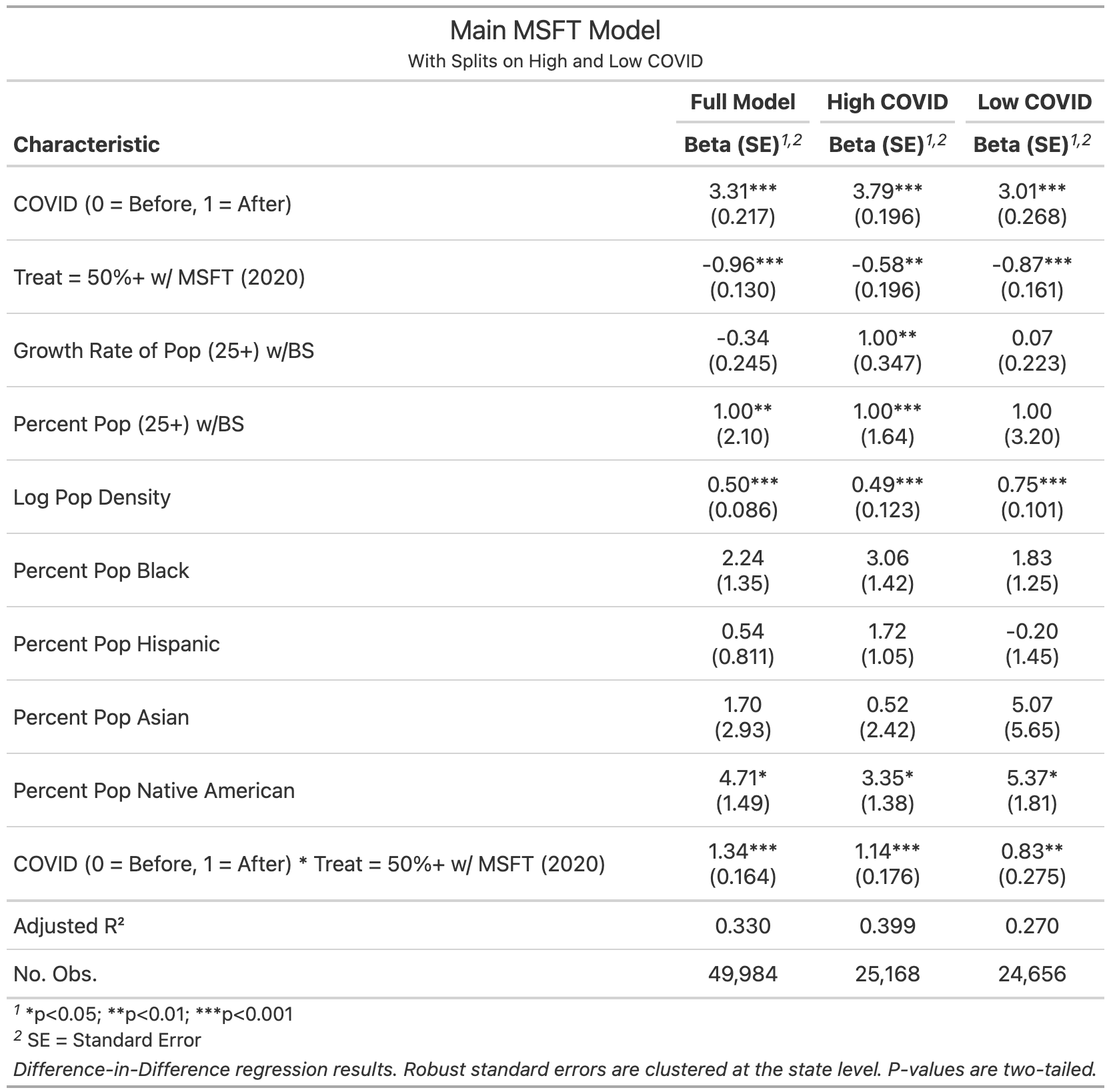


**Table G10:** Full base model results, with added splits on high and low COVID cases in July 2020. Robust standard errors, clustered at the state level, are included below each estimate with statistical significance indicated by the stars based off of a two-tailed p-test. Fixed effects at State and Month are included in all models. Here we see that when all else equal, after COVID, counties with more than 50% access to 25 Mbps download and 3 Mbps upload and in areas that have higher levels of COVID, experience an increase of 0.86% in their unemployment rates over similar counties that have less than 50% access. This is in keeping with counties with more than 50% access to 25 Mbps download and 3 Mbps upload and in areas that have lower levels of COVID, which experience an increase of 0.89% in their unemployment rates.


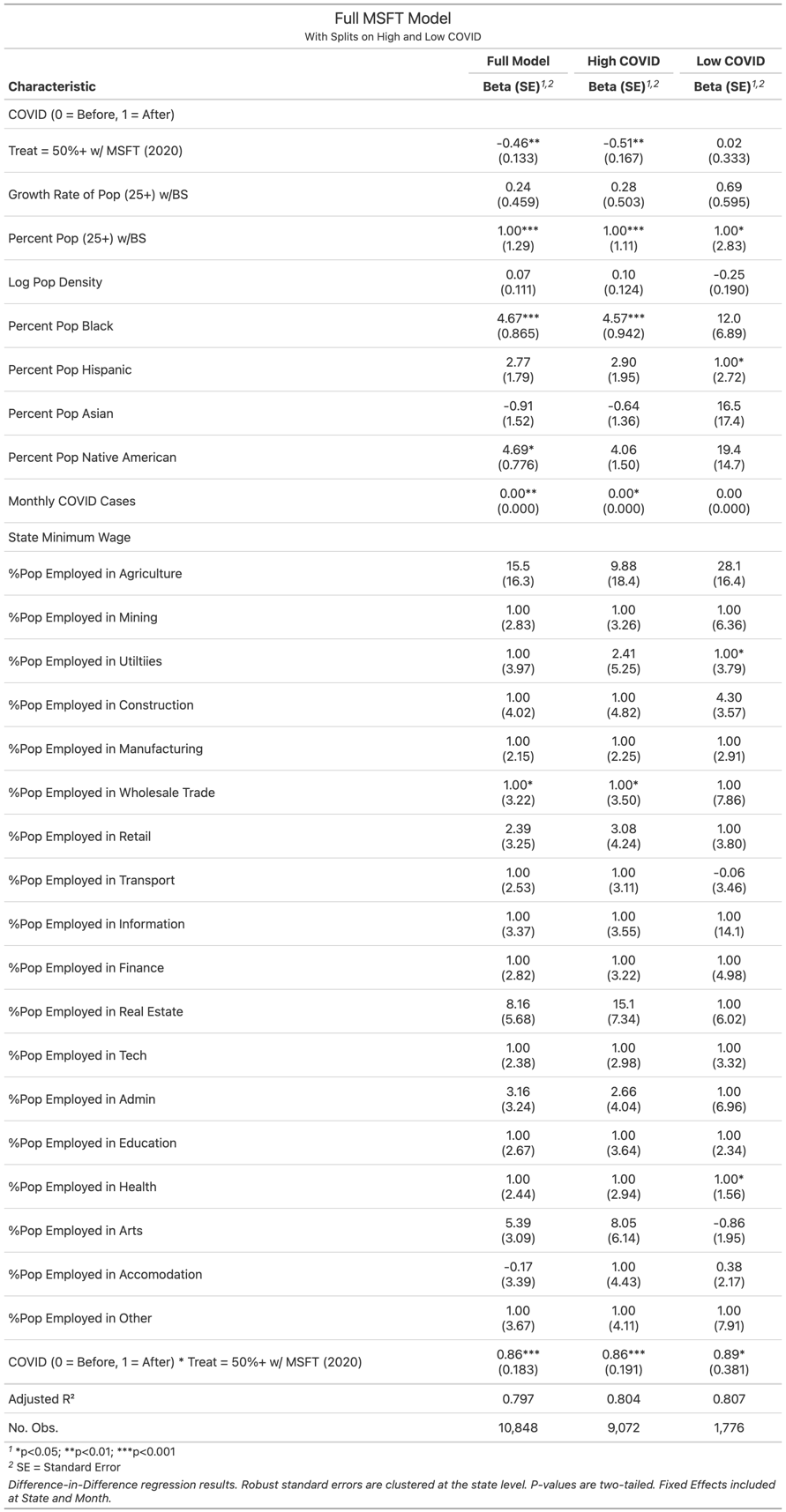


**Table G11:** This model uses levels of COVID to establish the treated and control groups in order to assess for simultaneity of effect. We also include splits on high and low broadband access, using the MSFT 2020 dataset. Robust standard errors, clustered at the state level, are included below each estimate with statistical significance indicated by the stars based off of a two-tailed p-test. Here we see that when all else equal, after COVID, counties with higher cases of COVID and in areas that have higher broadband access, experience an increase of 1.07% in their unemployment rates over similar counties that have less COVID. This is in comparison to counties with higher cases of COVID and in areas that have lower levels broadband access experience an increase of 0.78% in their unemployment rates. We find these effects greatly diminish once we incorporate the full set of controls as can be seen in Table G12.


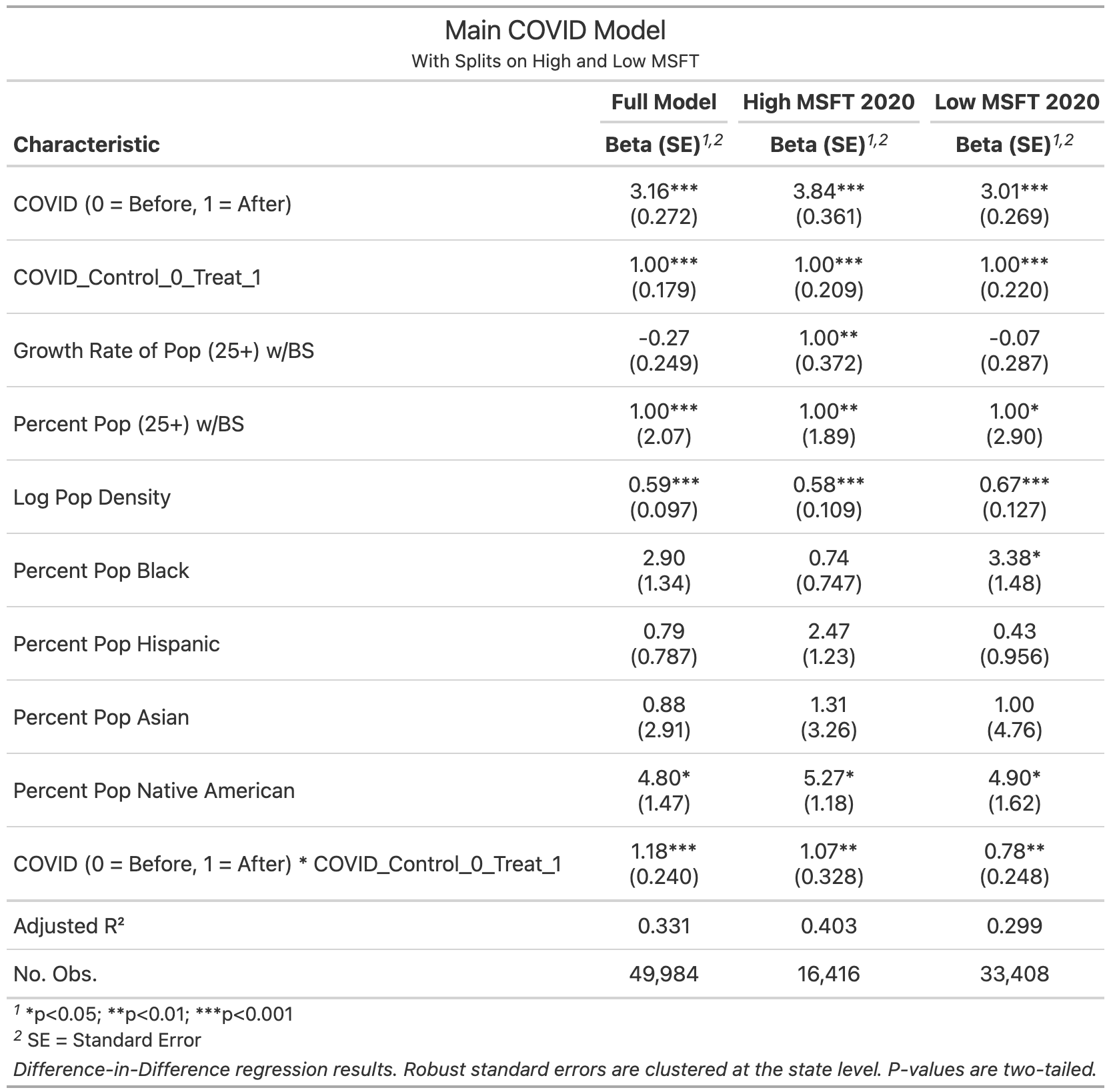


**Table G12:** This model uses levels of COVID to establish the treated and control groups in order to assess for simultaneity of effect and includes the full set of robust controls we have tested above. We also include splits on high and low broadband access, using the MSFT 2020 dataset. Robust standard errors, clustered at the state level, are included below each estimate with statistical significance indicated by the stars based off of a two-tailed p-test. Here we see that when we include all controls, the regression results for splitting control and treated counties by COVID become statistically non significant, suggesting that there is evidence of the distinct role that broadband plays on unemployment beyond those impacts from increased COVID-19 infection rates.


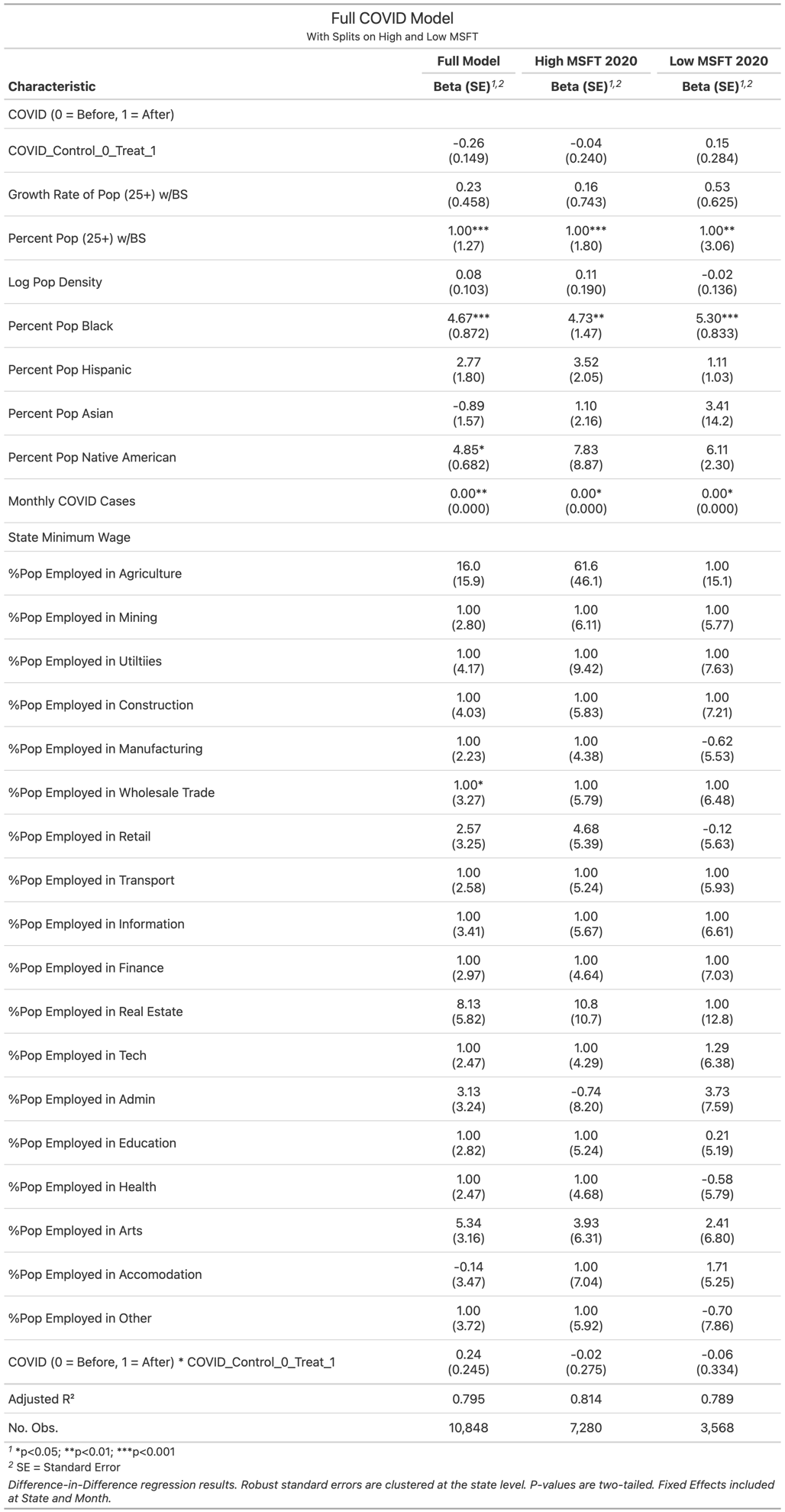


To further explore these results, we conduct an assessment of the industries included as controls. We find that the results in Tables G9 – G12 do not explain the same level of variation by industry as do the models where the treatment and control groups are based on broadband penetration as can be seen in Table G13. Thus, we see this as evidence of the distinct role that broadband plays on unemployment beyond those impacts from increased COVID-19 infection rates. Nonetheless, COVID-19 case load naturally may still play a role in moderating how broadband impacts unemployment amidst the pandemic and hence we note it is an important boundary condition.

**Table G13:** Main base model results with fixed effects included, split by industry type, with variation presented for when MSFT is used to separate treated and controlled units compared to when COVID is used to separated treated and control units. Robust standard errors, clustered at the state level, are included below each estimate with statistical significance indicated by the stars based off of a two-tailed p-test. The regression tables for all of these results can be found in Appendix I.

|  | **DiD Estimators for  Top 10% in Industry** | | **DiD Estimators for  Bottom 10% in Industry** | |
| --- | --- | --- | --- | --- |
| **Industry (as defined by NAICS)** | **MSFT as Treat/Control** | **COVID as Treat/Control** | **MSFT as Treat/Control** | **COVID as Treat/Control** |
| Accommodation | 1.38** [0.47] | -0.15 [0.49] | -0.19 [0.44] | 0.81 [0.39] |
| Admin | 1.62*** [0.30] | 0.58 [0.69] | -0.83* [0.33] | 0.59 [0.34] |
| Agriculture | 1.32 [1.46] | 0.35 [0.39] | 0.52 [0.38] | 0.82 [1.22] |
| Arts | 1.29** [0.43] | 0.30 [0.43] | 0.38 [0.38] | 0.07 [0.50] |
| Construction | 1.02*** [0.24] | 0.95* [0.41] | 1.53** [0.44] | 0.71* [0.30] |
| Ed | 0.92* [0.39] | 0.32 [0.32] | 0.64* [0.23] | -0.22 [0.33] |
| Finance | 1.81*** [0.33] | 2.50*** [0.31] | 1.21* [0.50] | -0.11 [0.40] |
| Health | 1.12* [0.42] | 1.16* [0.52] | 1.16* [0.50] | 0.95 [0.50] |
| Information | 1.74*** [0.30] | 2.09*** [0.31] | 0.57 [0.30] | -0.07 [0.35] |
| Manufacturing | 0.21 [0.30] | 0.06 [0.32] | 2.02*** [0.30] | 1.25* [0.50] |
| Mining | 1.02 [0.65] | 1.49* [0.41] | 2.28** [0.61] | -0.28 [0.97] |
| Other | 0.72* [0.29] | 1.14* [0.39] | 0.26 [0.41] | 0.78* [0.32] |
| Real Estate | 2.22*** [0.37] | 0.23 [0.50] | -0.02 [0.39] | 0.41 [0.36] |
| Retail Trade | 1.55*** [0.33] | 0.91 [0.50] | 1.35** [0.41] | 1.04** [0.33] |
| Tech | 1.60*** [0.30] | 1.88*** [0.29] | 0.03 [0.37] | 0.55 [0.38] |
| Transport | 1.30** [0.38] | 1.07*** [0.27] | 0.31 [0.31] | 0.63 [0.39] |
| Utilities | 0.51 [0.43] | 1.20** [0.37] | 0.36 [0.35] | -0.03 [0.62] |
| Wholesale Trade | 0.63 [0.54] | 1.85** [0.46] | 1.26** [0.42] | -0.11 [0.46] |

**Table G14:** Results of the main model including Fixed Effects at the State and Month level in the left hand column and at the County and Month level in the right hand column. Robust standard errors, clustered at the state level, are included below each estimate with statistical significance indicated by the stars based off of a two-tailed p-test. Here we see that when all else equal, after COVID, counties with more than 50% access to 25 Mbps download and 3 Mbps upload as measured by MSFT 2020 with fixed effects included at the state and month level, experience an increase of 1.24% in their unemployment rates, respectively, over similar counties that have less than 50% access. This is statistically consistent and robust with the findings of our main results in the paper. We also find that when all else equal, after COVID, counties with more than 50% access to 25 Mbps download and 3 Mbps upload as measured by MSFT 2020 with fixed effects included at the county and month level, experience an increase of 1.15% in their unemployment rates, respectively, over similar counties that have less than 50% access. This is also statistically consistent and robust with the findings of our main results in the paper.

*
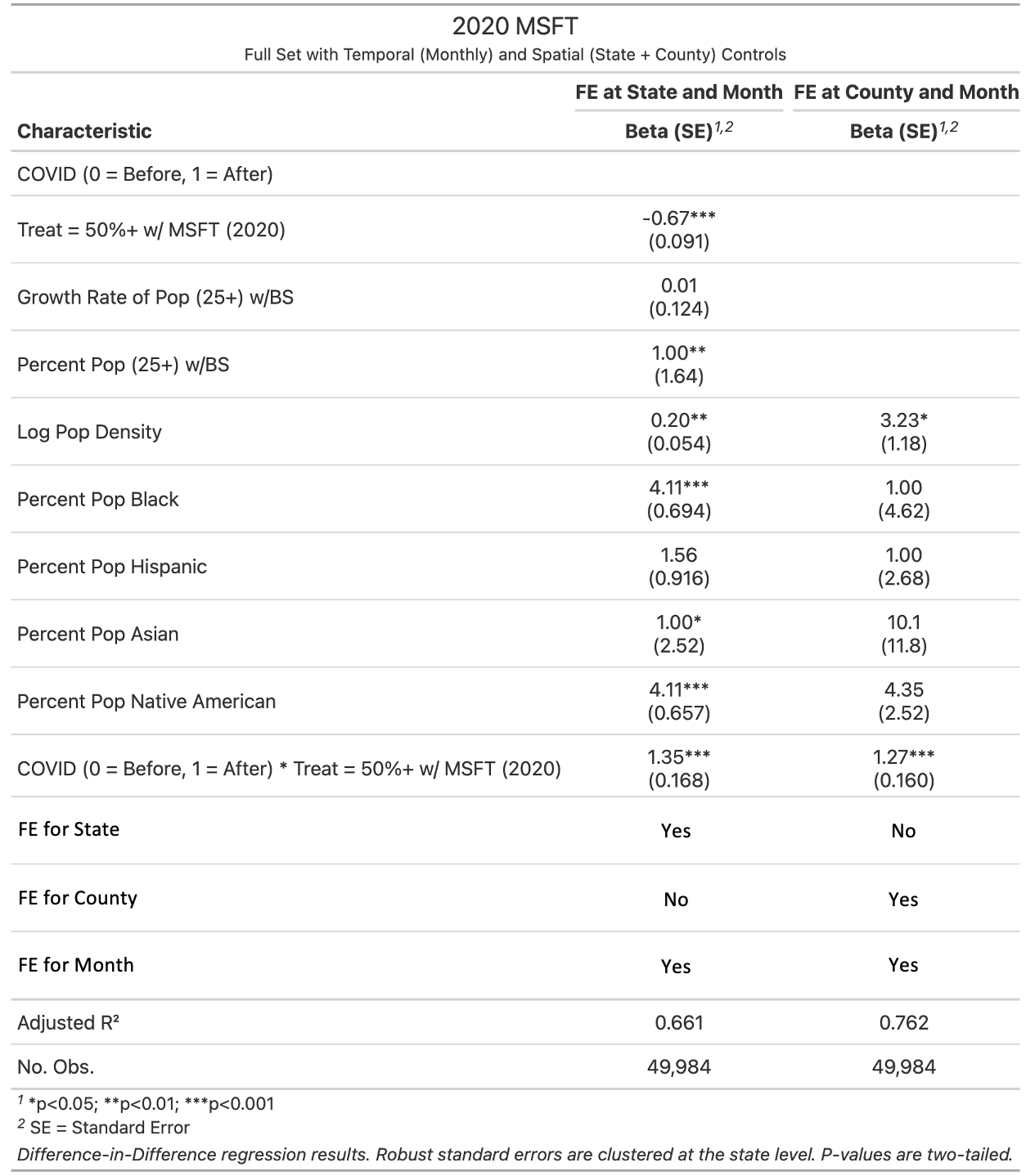
*

In addition to running strict robustness tests, we also present the supplementary parallel trends from analysis conducted in the main body of the paper. Figure G1 shows the parallel trends for Figure 3 in the main body of the paper while Figure G2 shows the parallel trends for Figure 4.


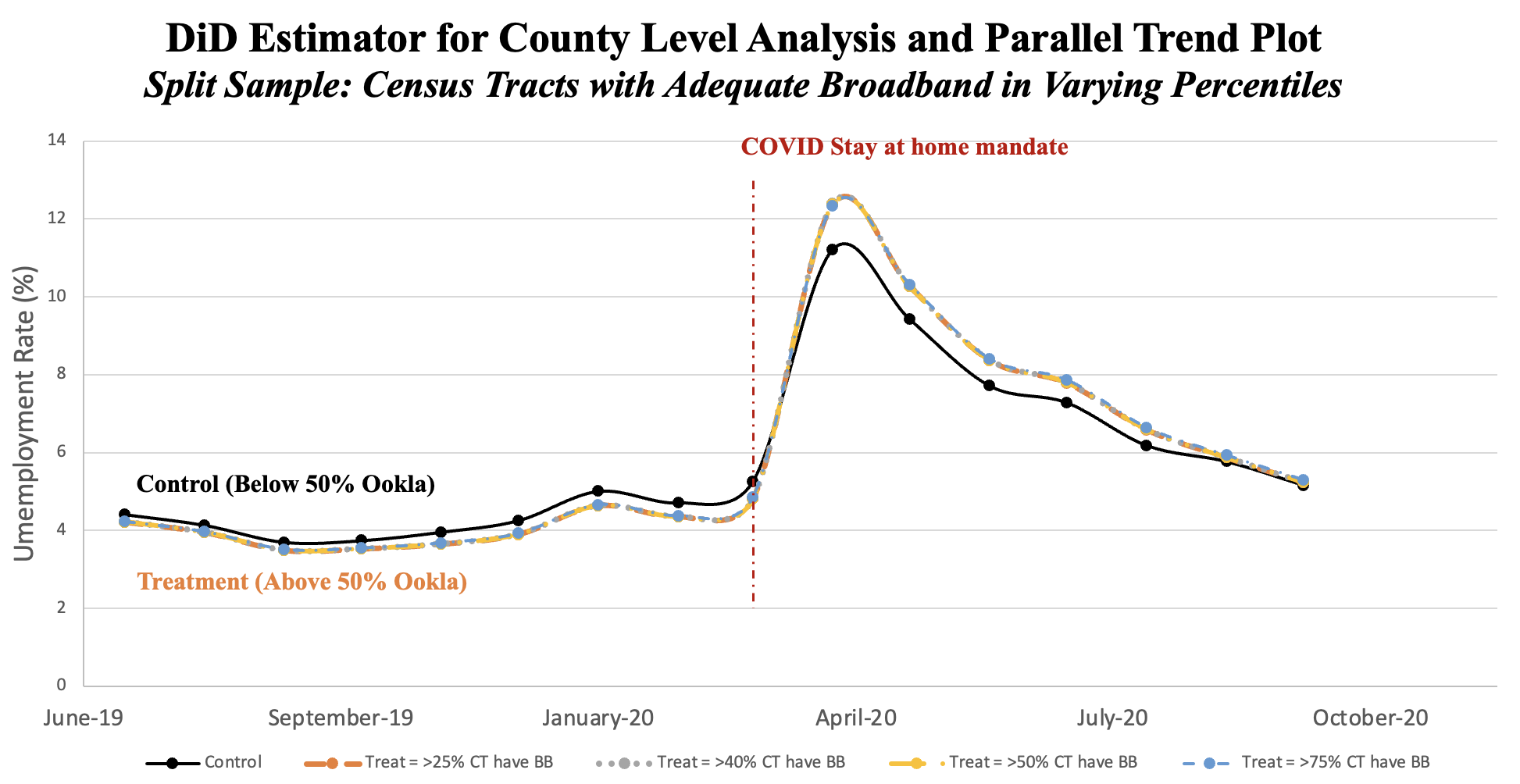


**Figure G1**: The plot of the parallel trends between the control (below adequate access to broadband at a county level) and treatment (above adequate access to broadband on average at a county level, parsed out by counties with varying levels of census tracts with adequate broadband access). The parallel trends hold prior to the shock of the COVID-19 pandemic, and while the differences between treated and controls groups are small they can be observed in the plots. The ideas here is to assess whether concentrated in-access is what drives these potential effects. The full regressions that underpin these results.


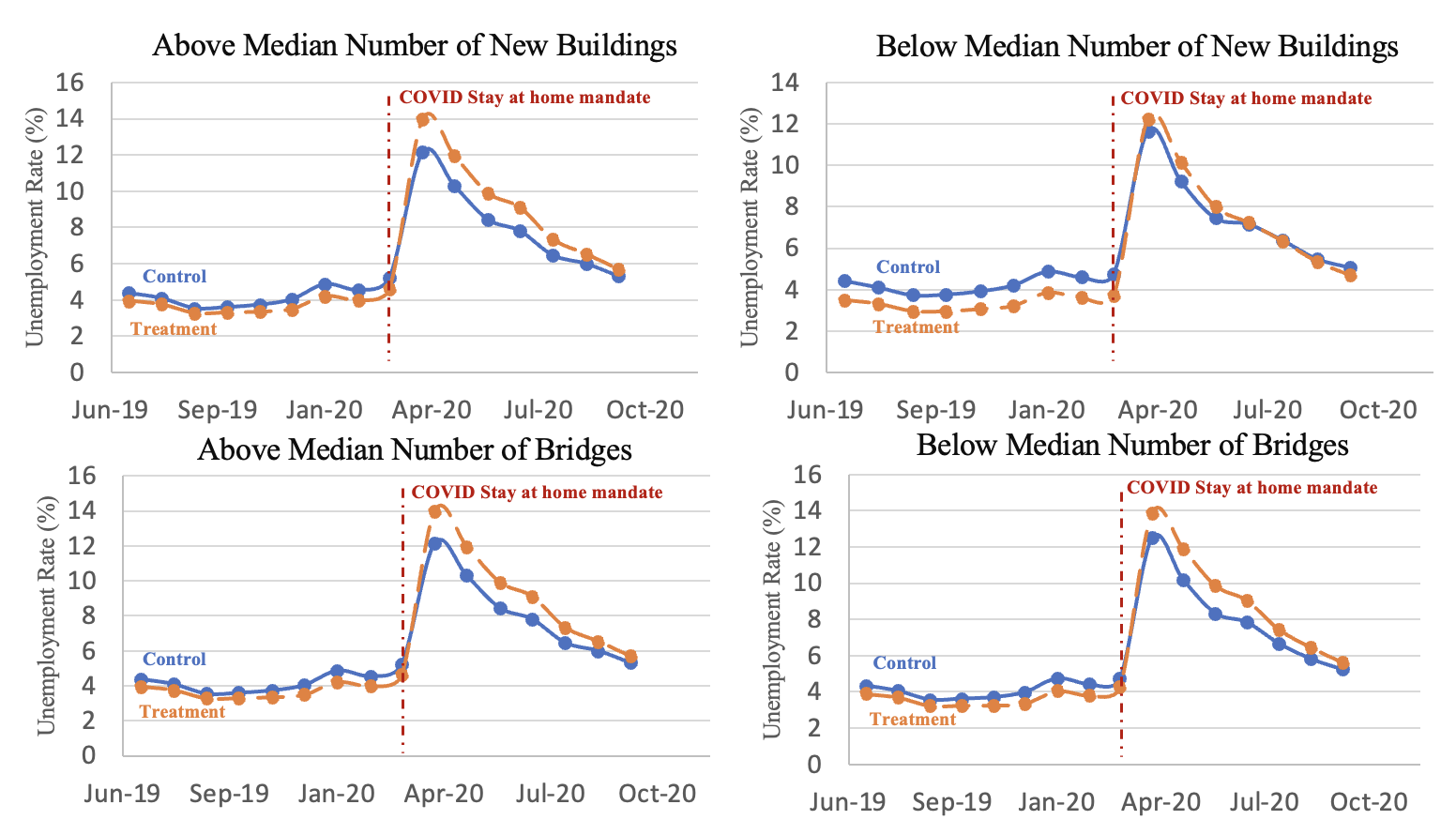


**Figure G2:** The parallel trends between the control (below adequate access to broadband at a county level) and treatment (above adequate access to broadband on average at a county level) for both number of buildings (upper row) and number of bridges (bottom row). Parallel trends hold prior to the shock of the COVID-19 pandemic.

# Appendix H: Synthetic Controls Analysis and Bayesian Causal Inference with Time-Series Cross-Sectional Data

In addition to our robust, clustered OLS regression, we also ran a synthetic control approach. In this approach, counterfactuals to the treated unit (above percent threshold access to broadband) are created as composite of the control units (below percent threshold access to broadband) that have the tightest fit prior to the onset of the treatment, hence the name “synthetic controls”^22^. Historically, this method only allowed for a single treated unit^22^, or a single aggregation of treated units^23^. However, advancements now allow for multiple and disaggregated treated units^24^. Figure H1 shows a full overview of the raw data, both pre and post the start of the treatment. Figures H2-H5 shows the average treatment effect on the treated (ATT) separately (Figure H2), in relation to the average control units (Figure H3 and H4), as well as individually across each treated unit and its counterpart synthetic control (Figure H5)

**Figure H1:** Displaying the raw treated and control data as a function of time

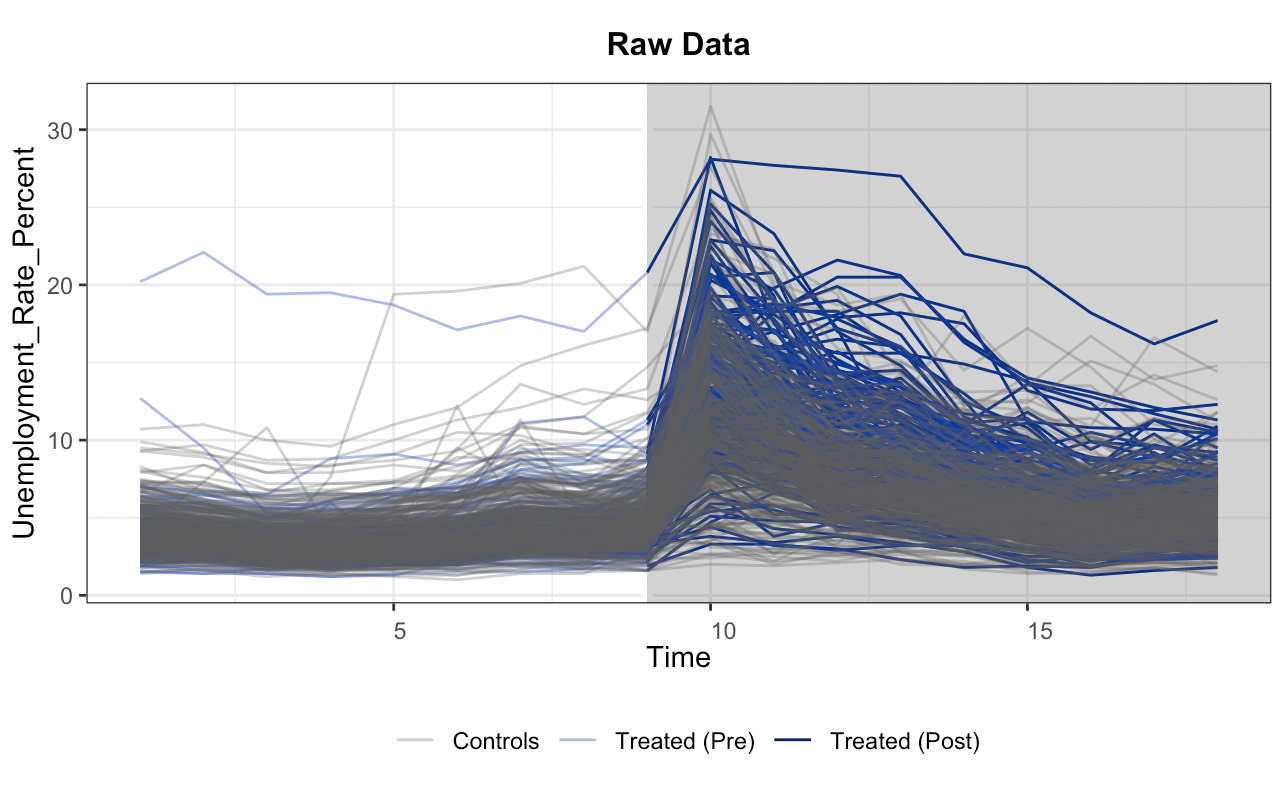


**Figure H2: Estimated Average Treatment Effect of the Treated (ATT).** Here the bold line is the ATT and the gray band is the confidence interval.
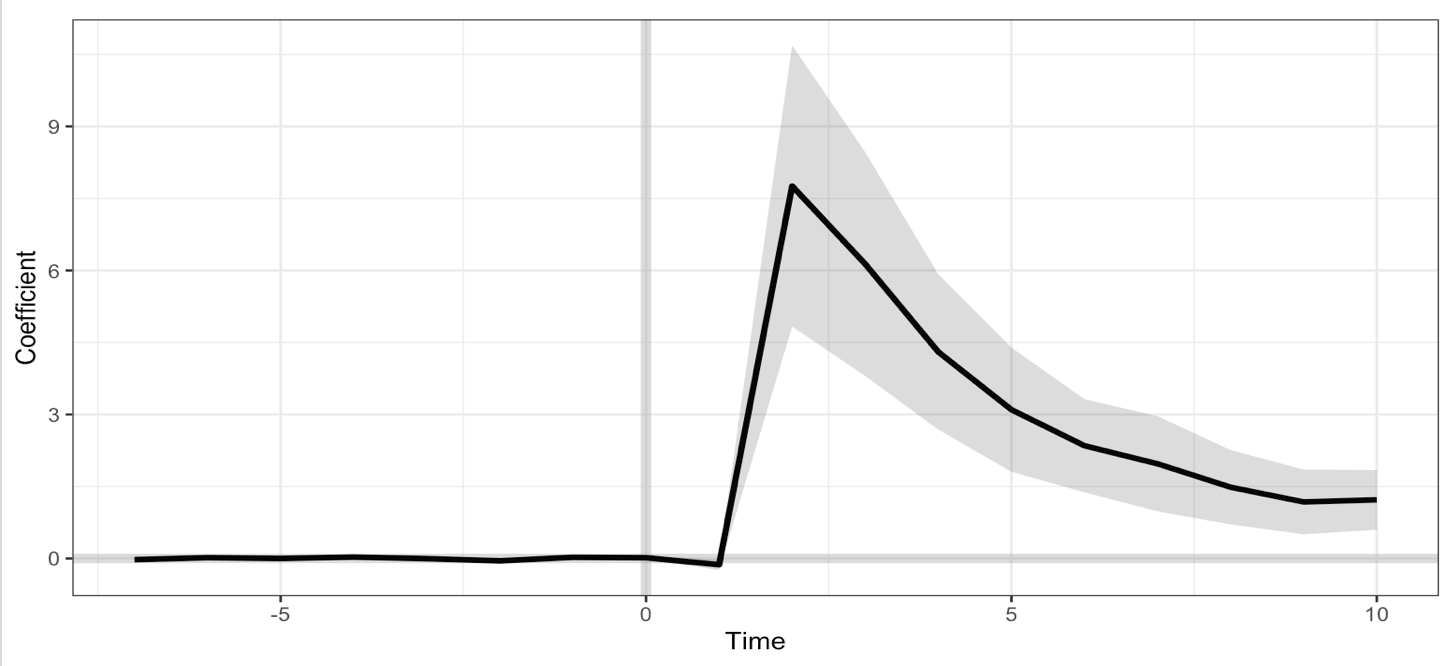


**Figure H3:** Estimated Average Treatment Effect of the Treated (ATT) with the Counterfactual


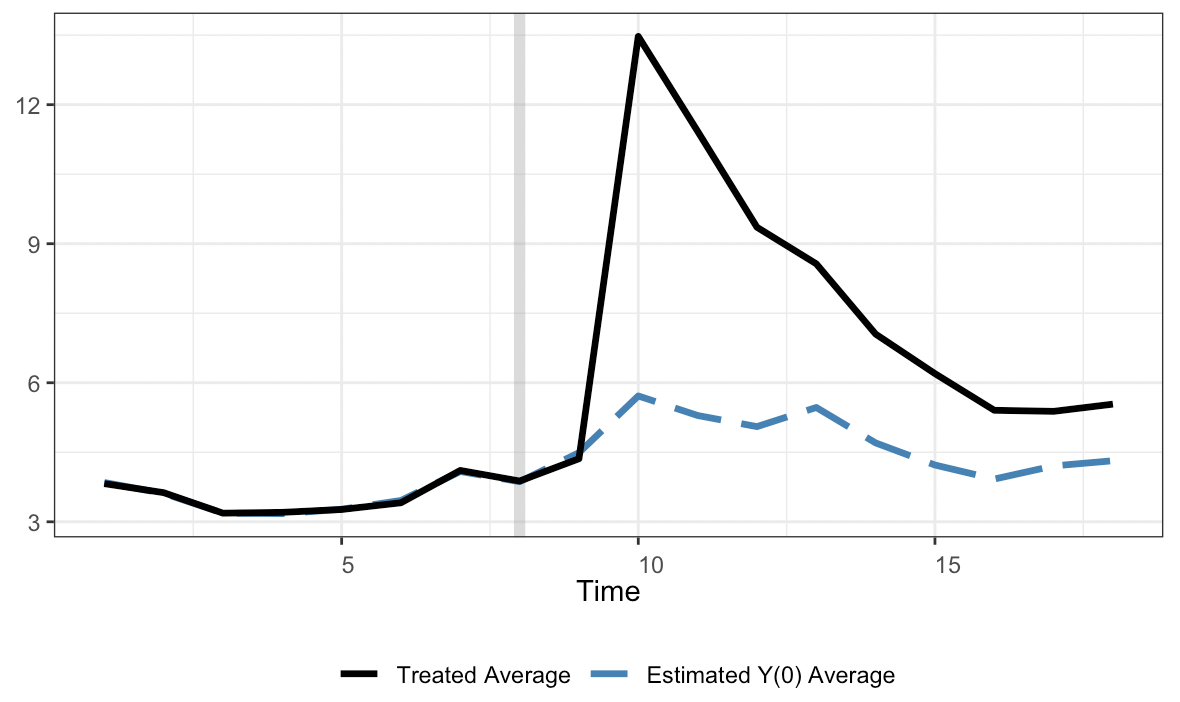


**Figure H4:** Estimated Average Treatment Effect of the Treated (ATT) with the Counterfactual averages


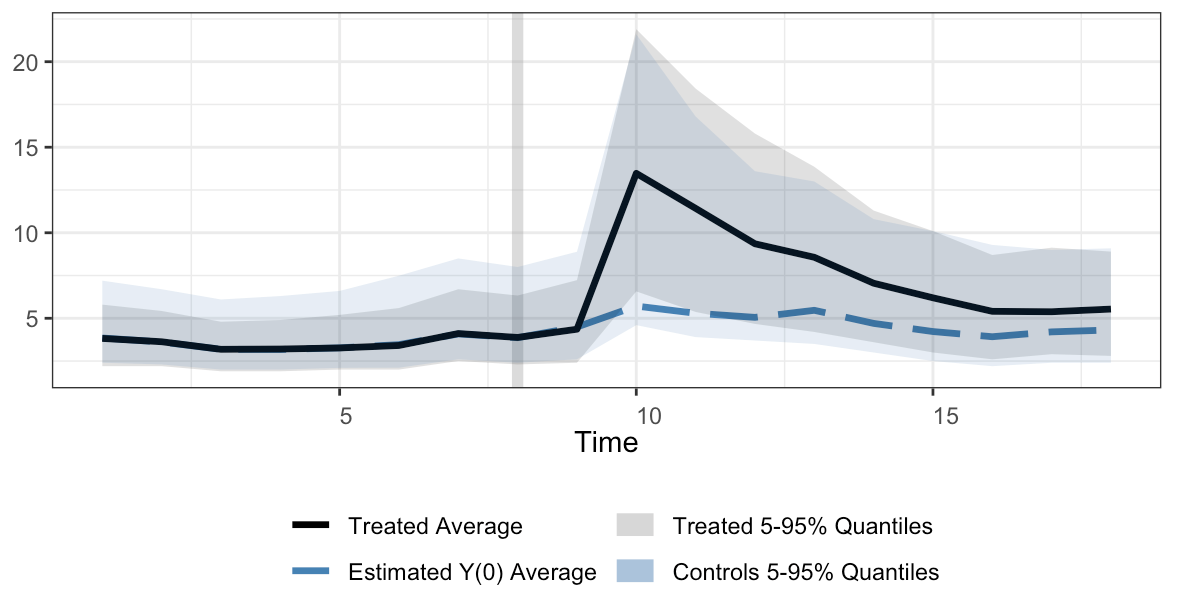


**Figure H5: Estimated Average Treatment Effect of the Treated (ATT) with the Counterfactual averages as individual measures**
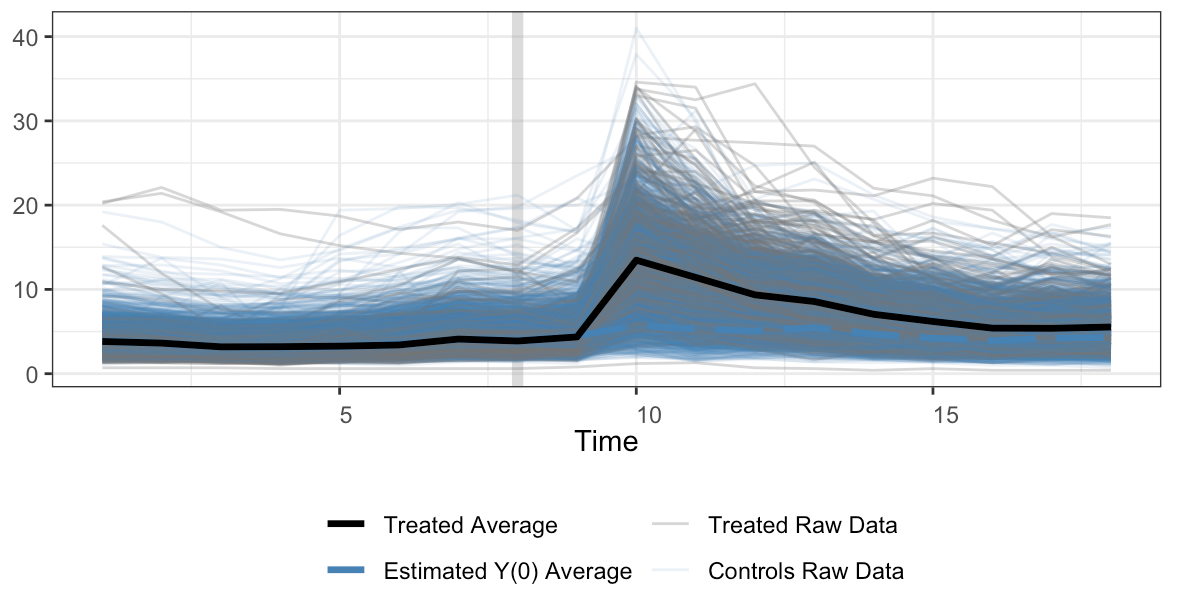


Table 2 (in the main body) presents the results for the generalized synthetic controls approaches that were visualized above, as well as a Bayesian analog that allows for inclusion of controls that are not time-varying. We can see from this approach that the main model results are highly consistent and robust to this approach.

# Appendix I: Regression Tables

**Table I1: MSFT 2020**

**
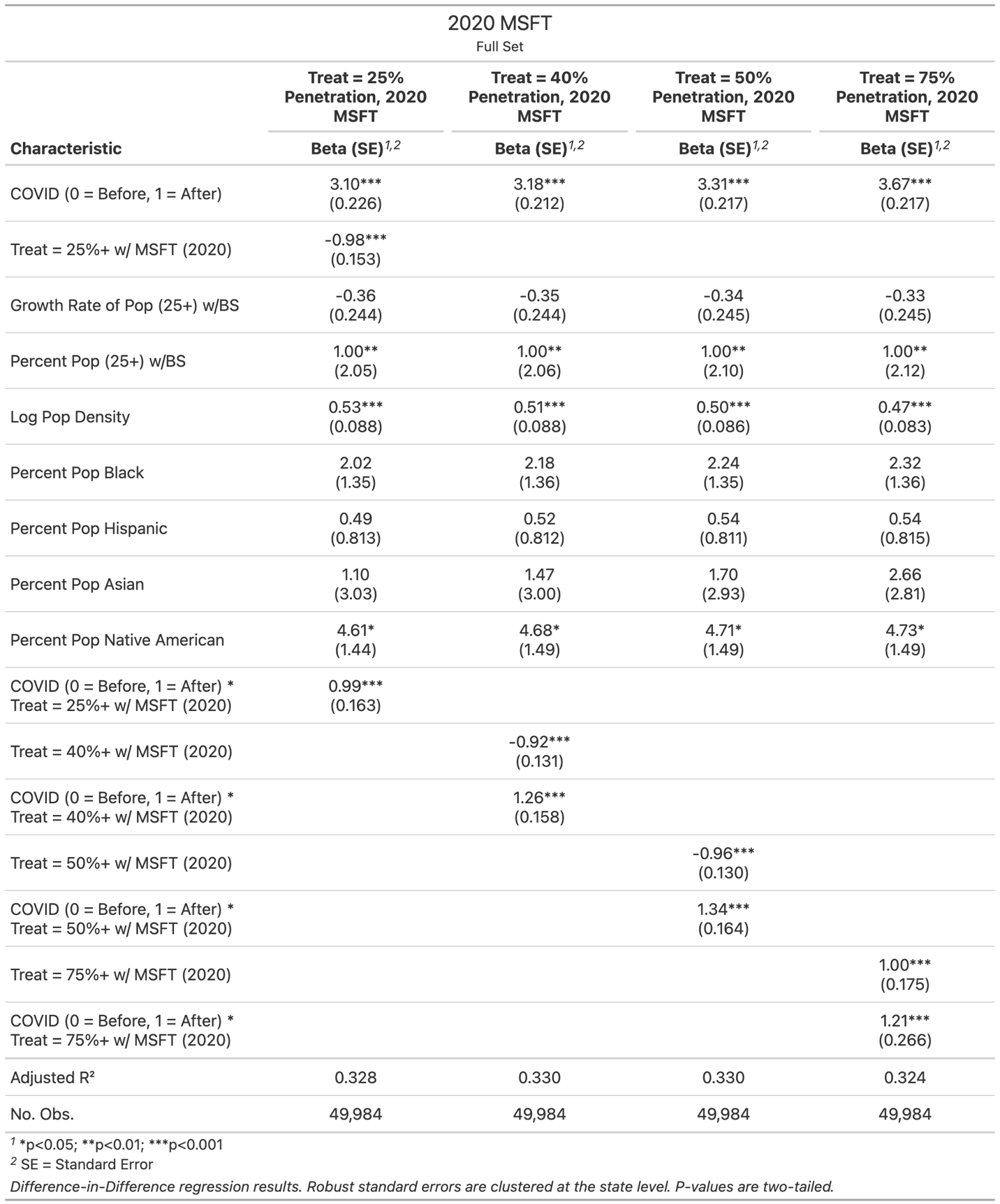
**

**Table I2: MSFT 2019**

**
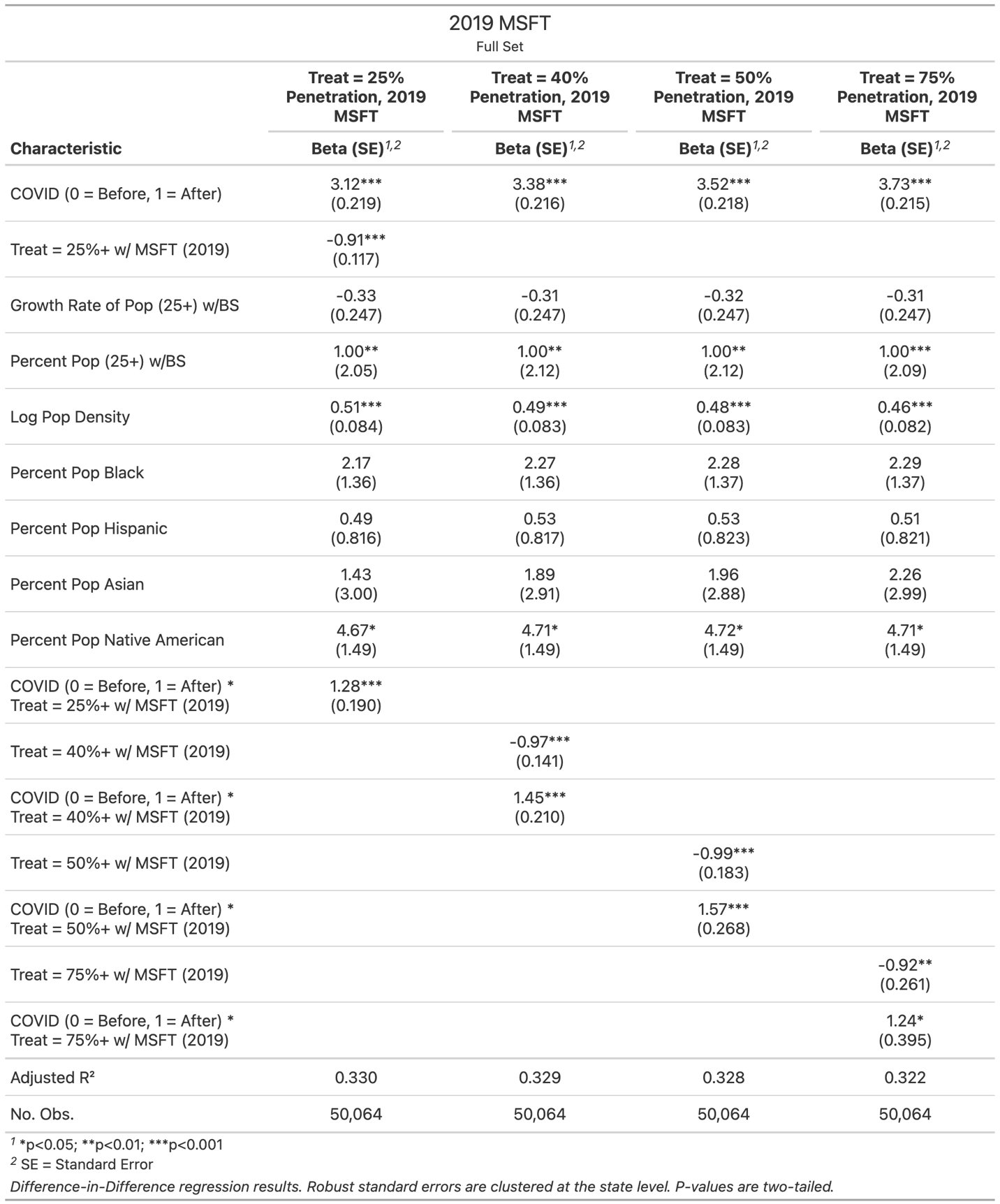
**

**Table I3: FCC 2019**

**
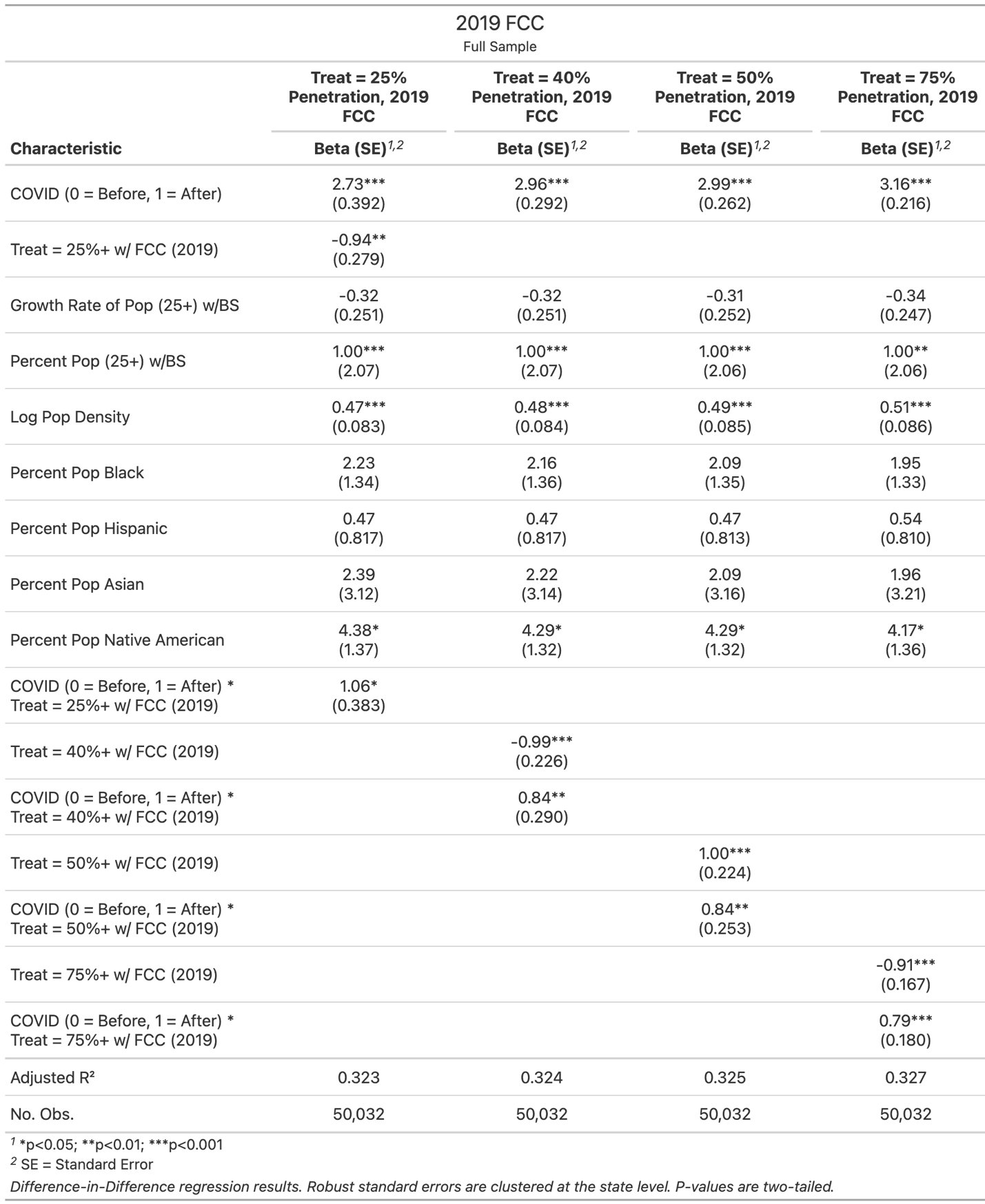
**

**Table I4: ACS 2020**

**
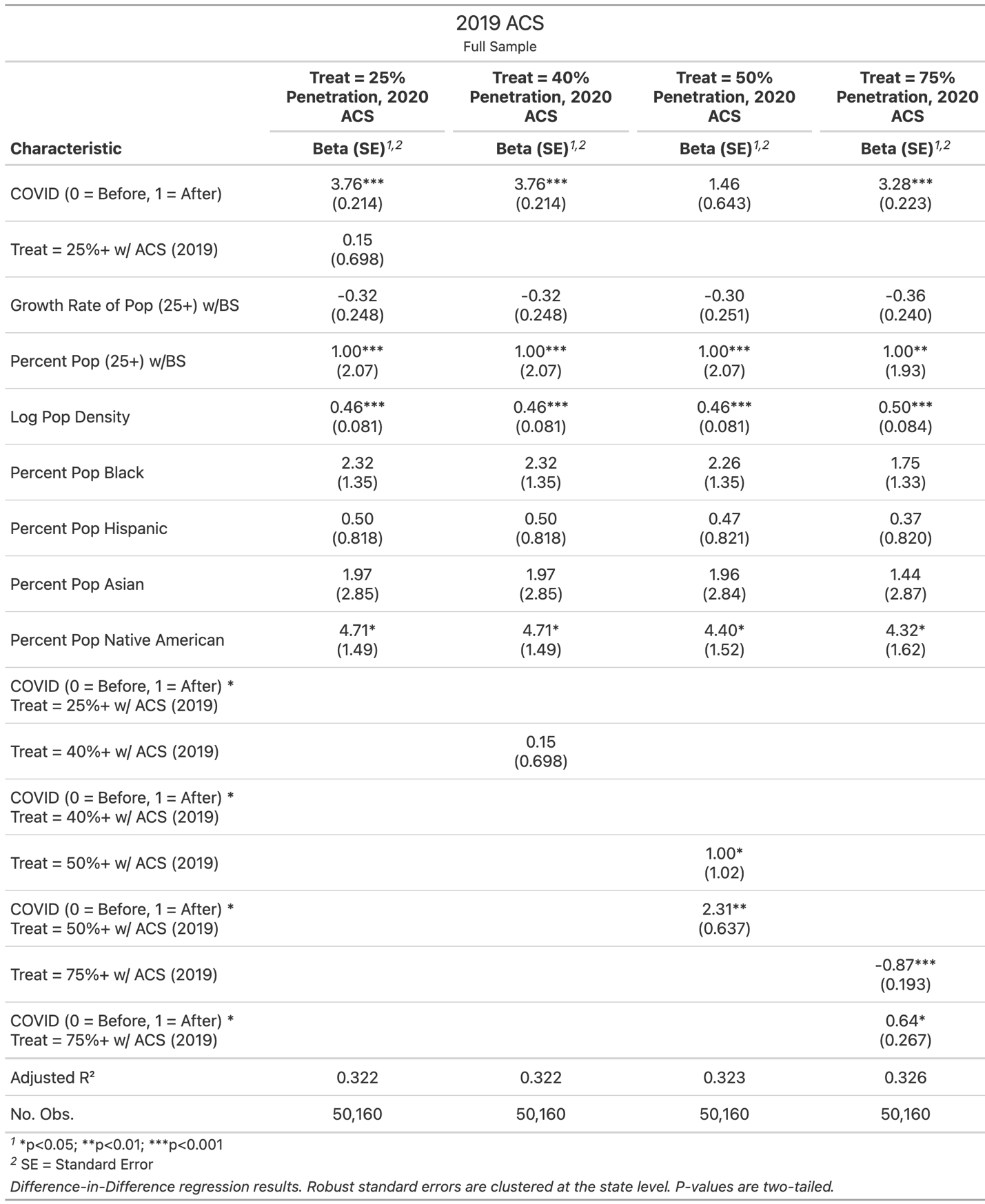
**

**Table I4: MSFT 2020 Urban**


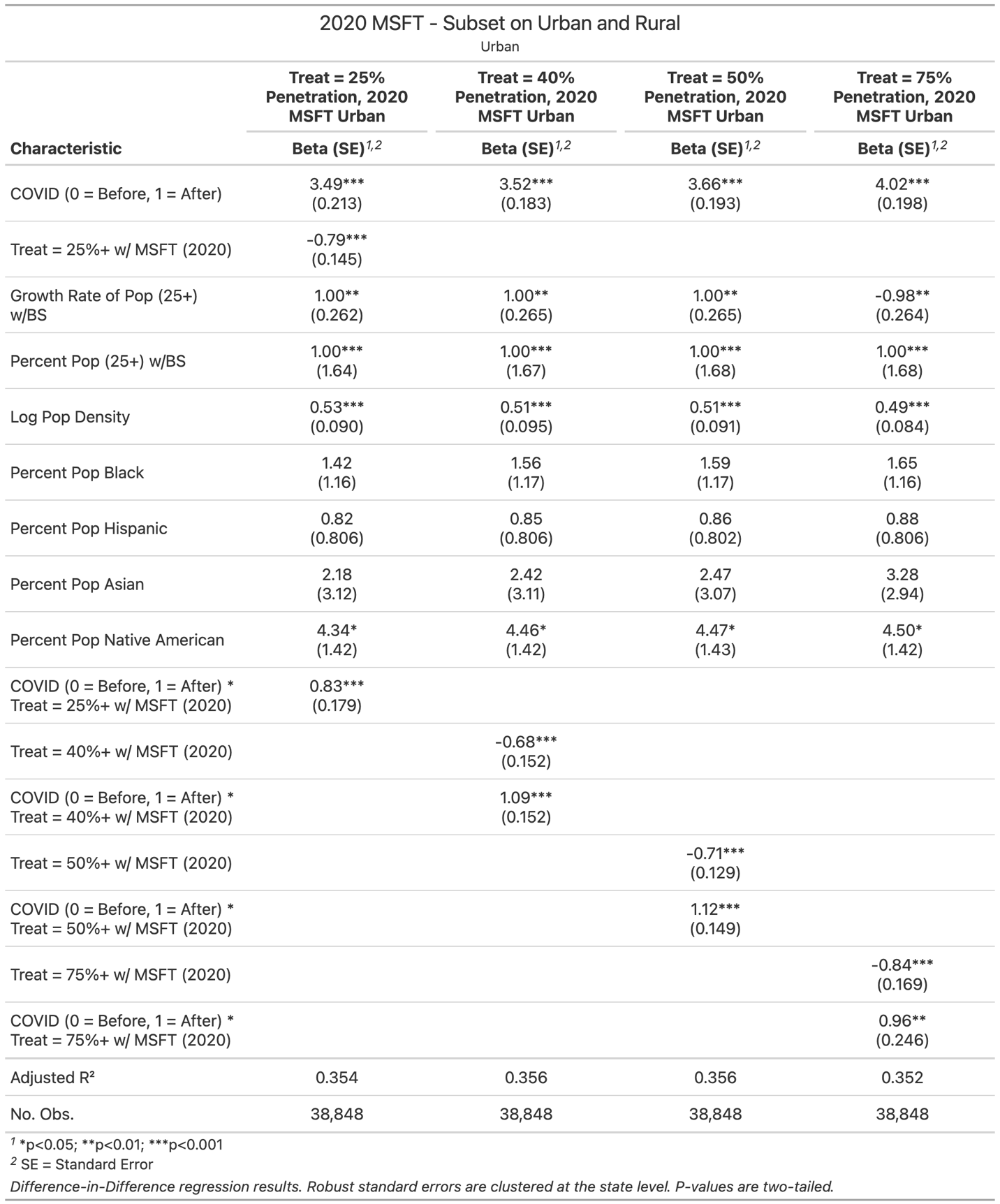


**Table I5: MSFT 2020 Rural**

**
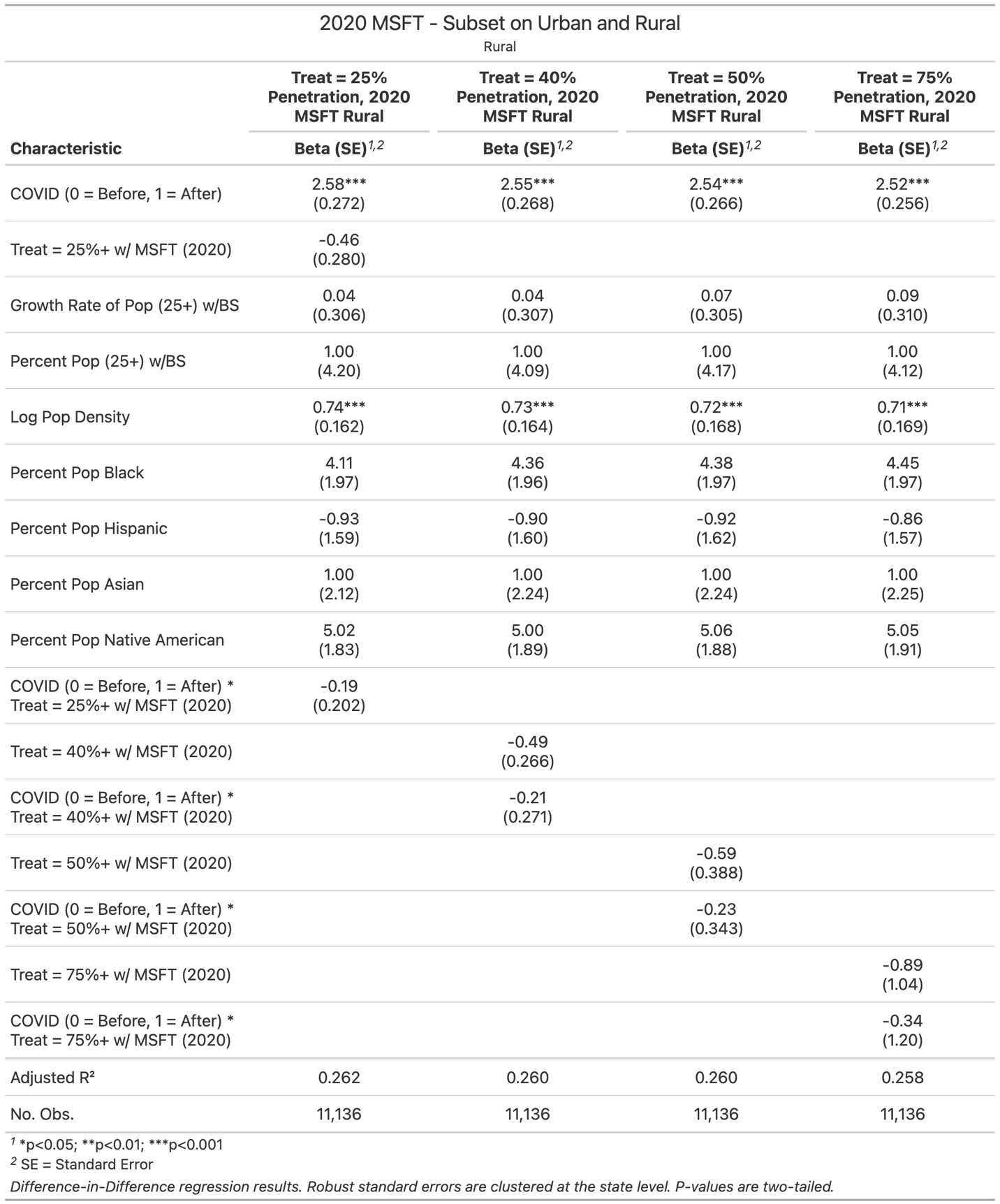
**

**Table I6: MSFT 2020 Above Median Essential Industry Workers**

**
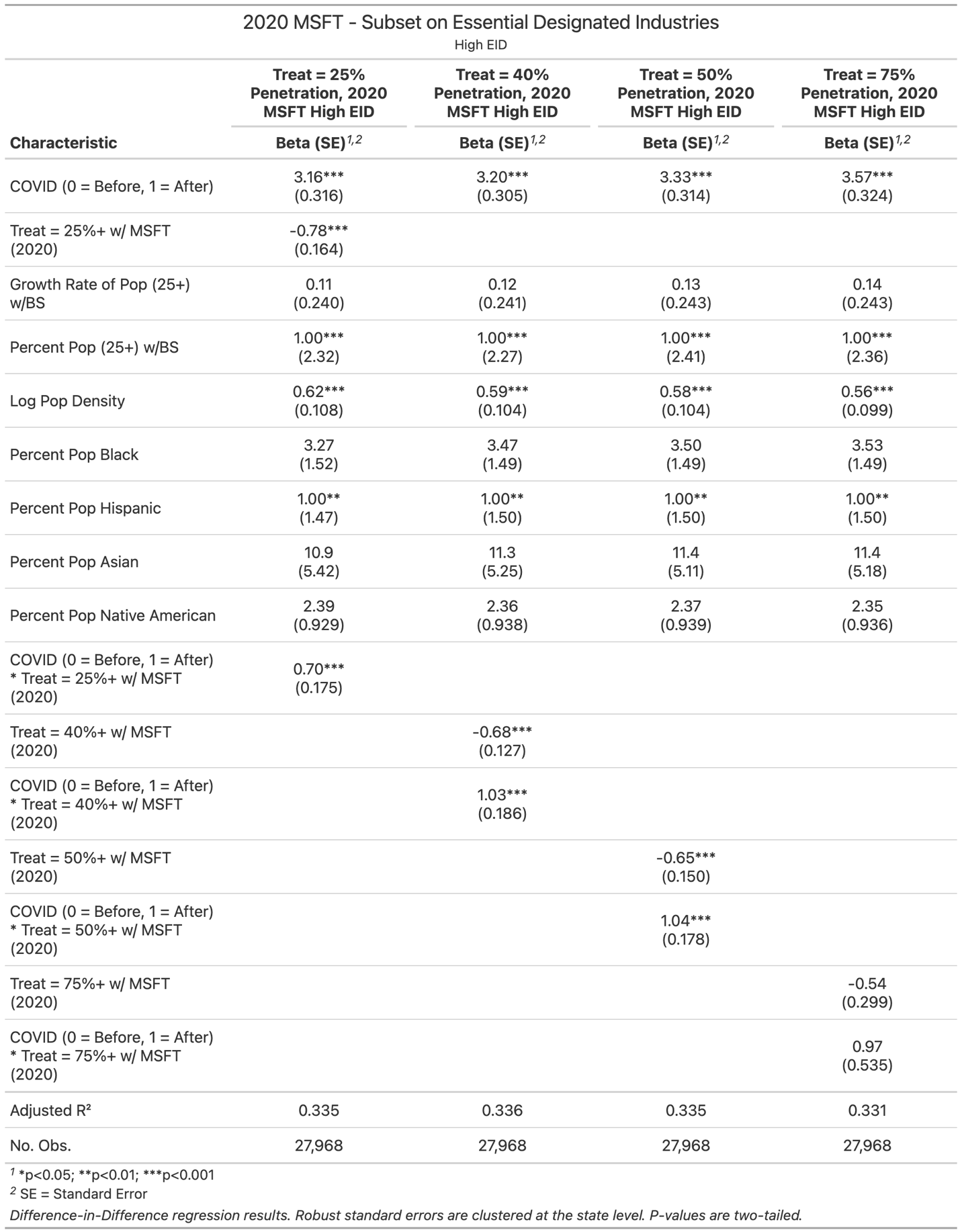
**

**Table I7: MSFT 2020** **Below Median Essential Industry Workers**

**
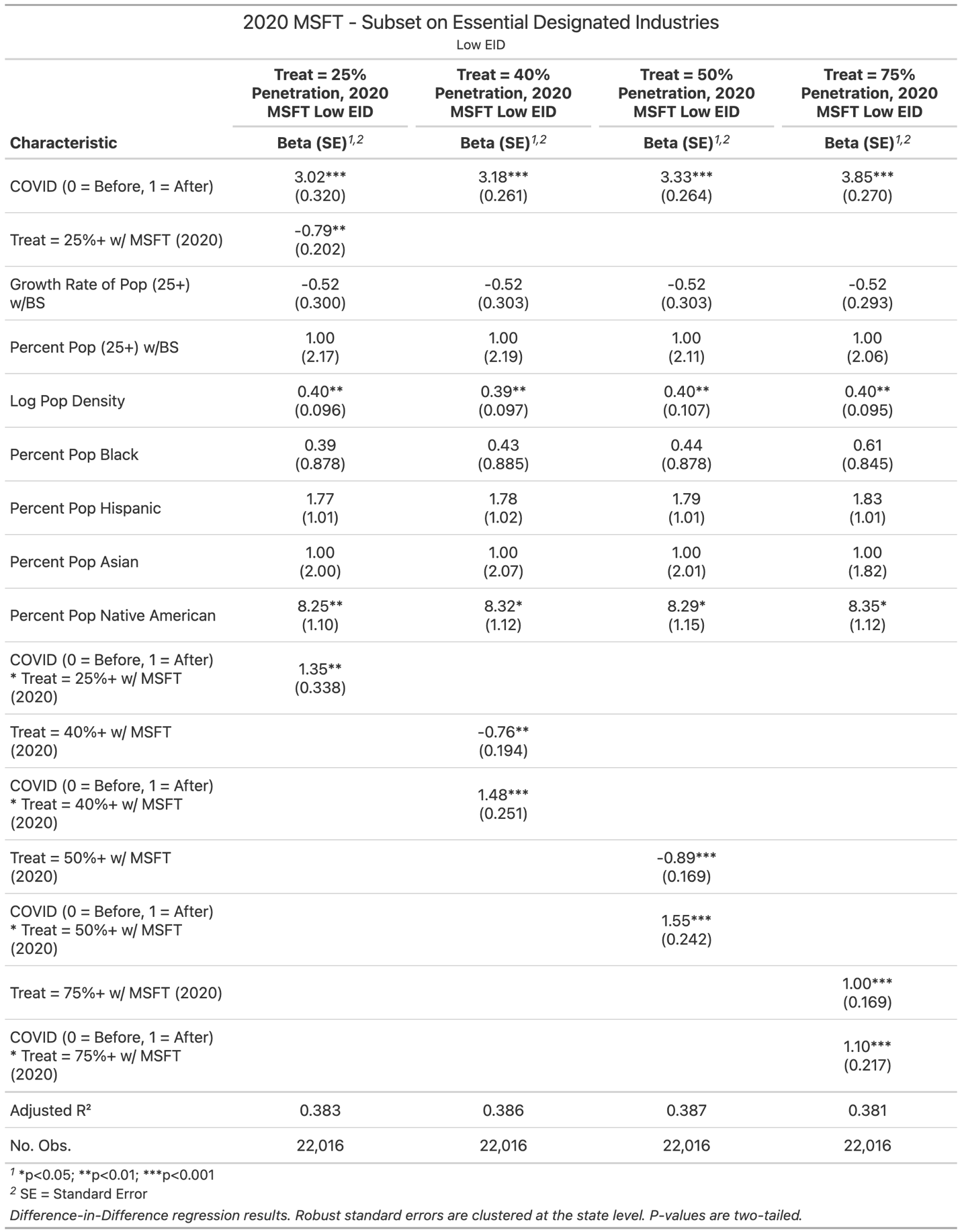
**

**Table I8: MSFT 2020 Above Median Income**

**
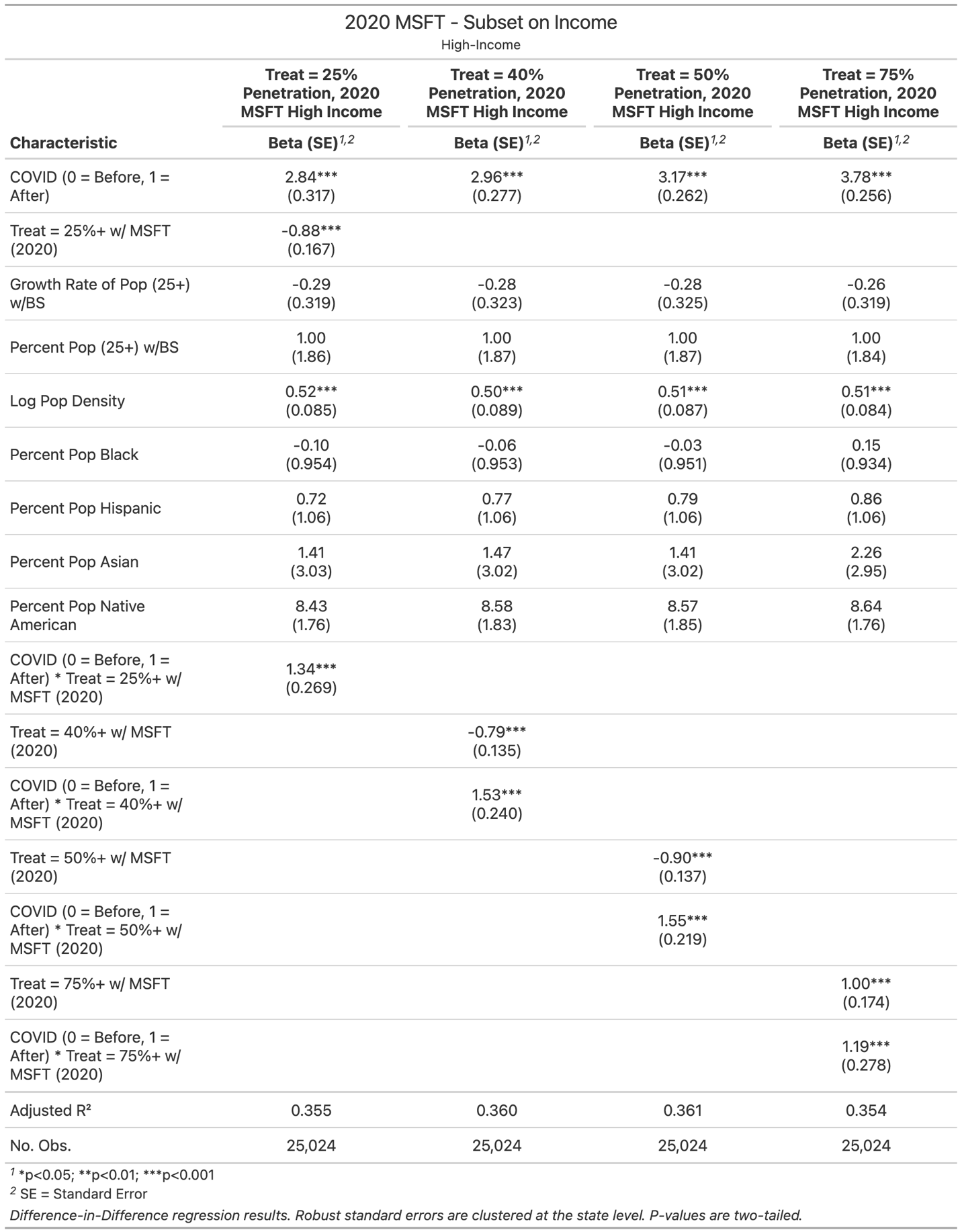
**

**Table I9: MSFT 2020** **Below Median Income**


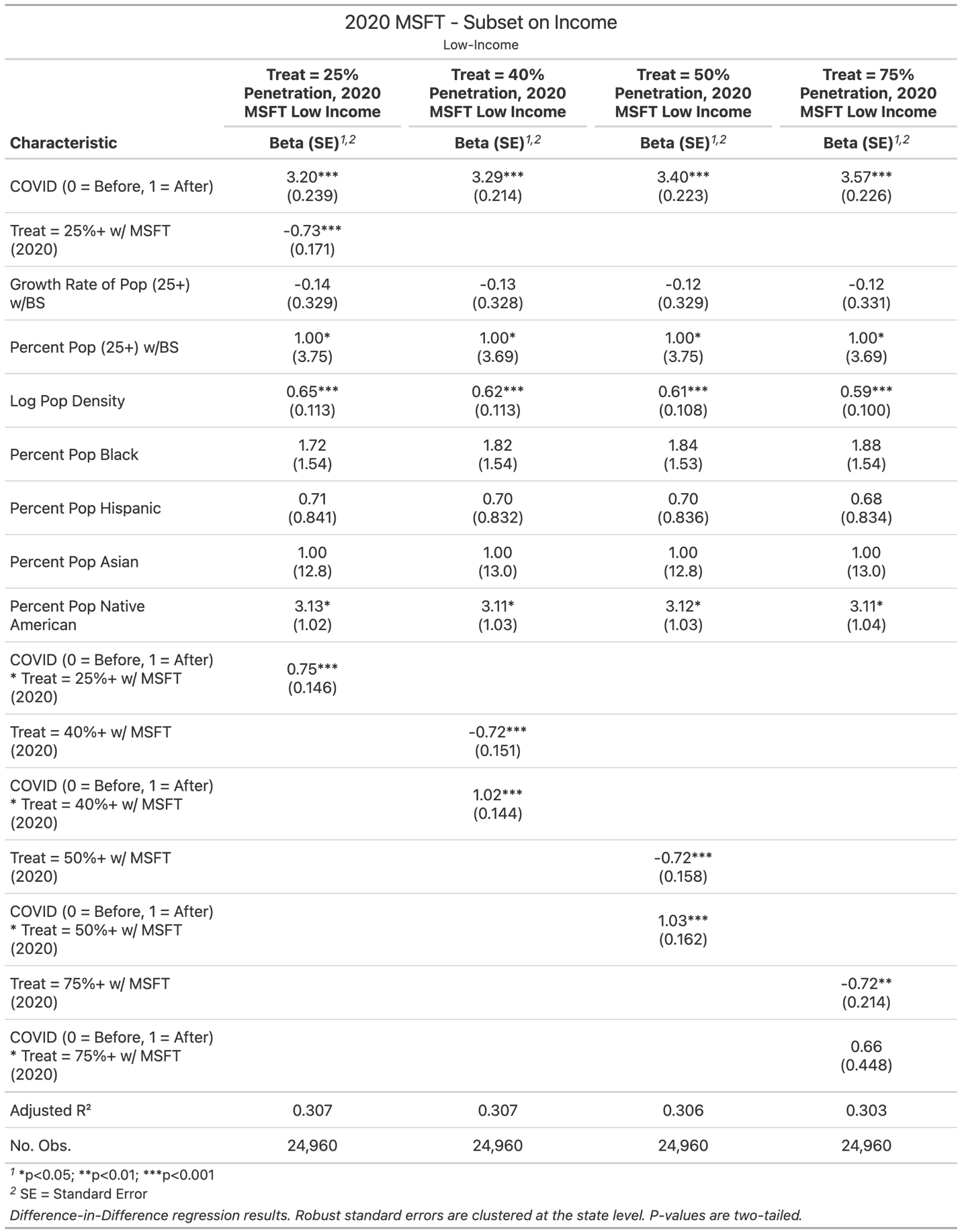


**Table I10: MSFT 2020 Above Median Percentage Black**


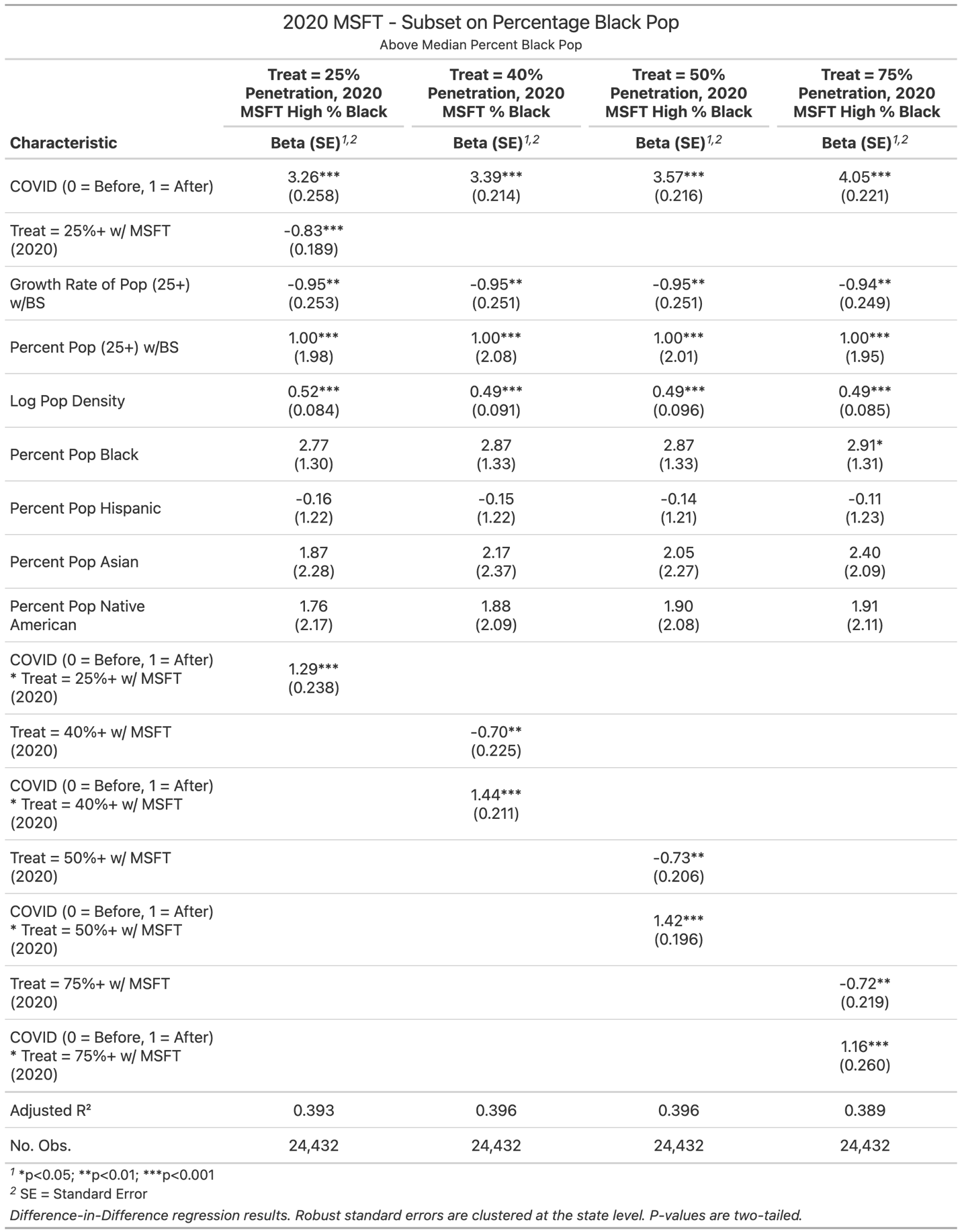


**Table I11: MSFT 2020 Below Median Percentage Black**

**
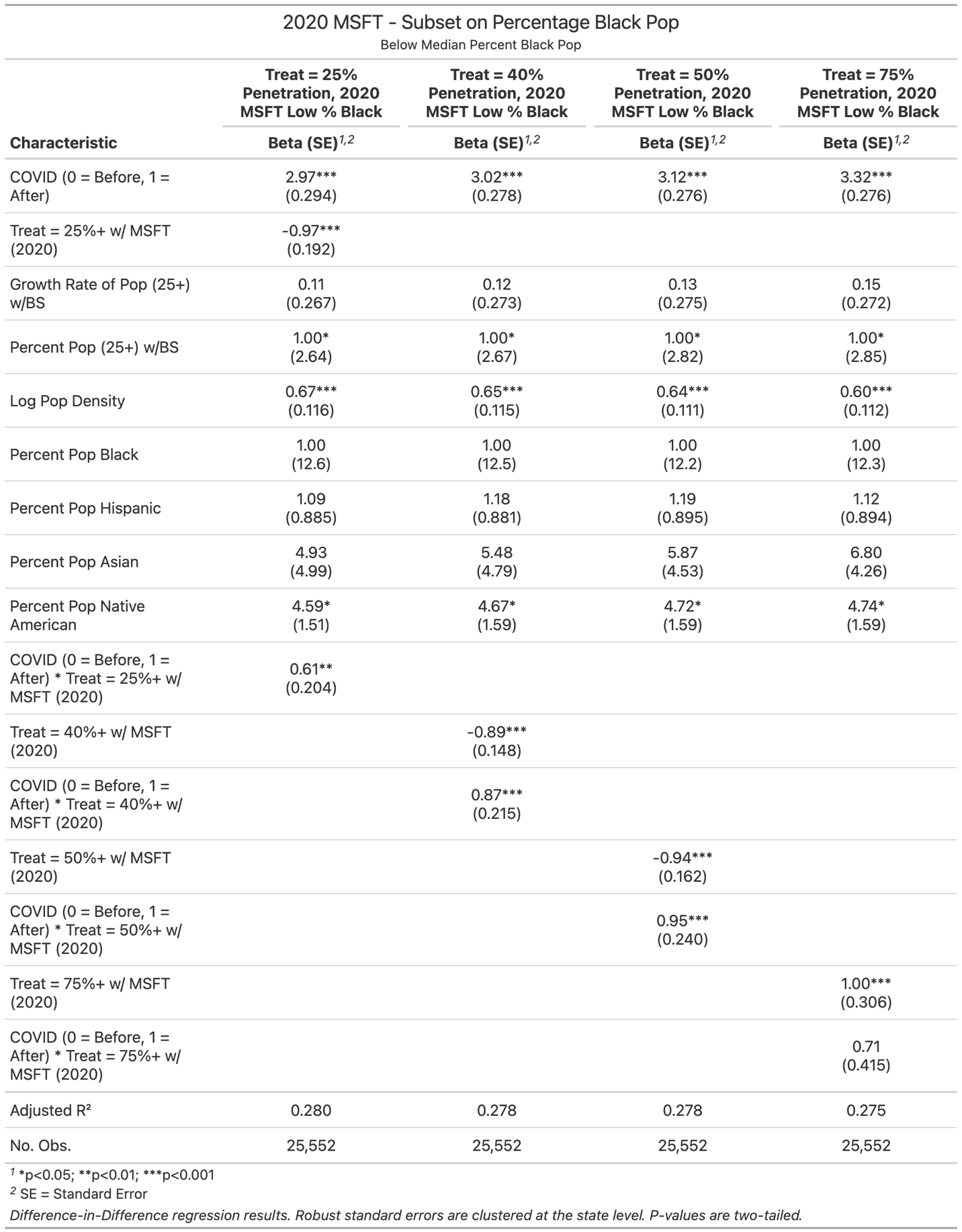
**

**Table I12: MSFT 2020 Above Median Percentage Hispanic**

**
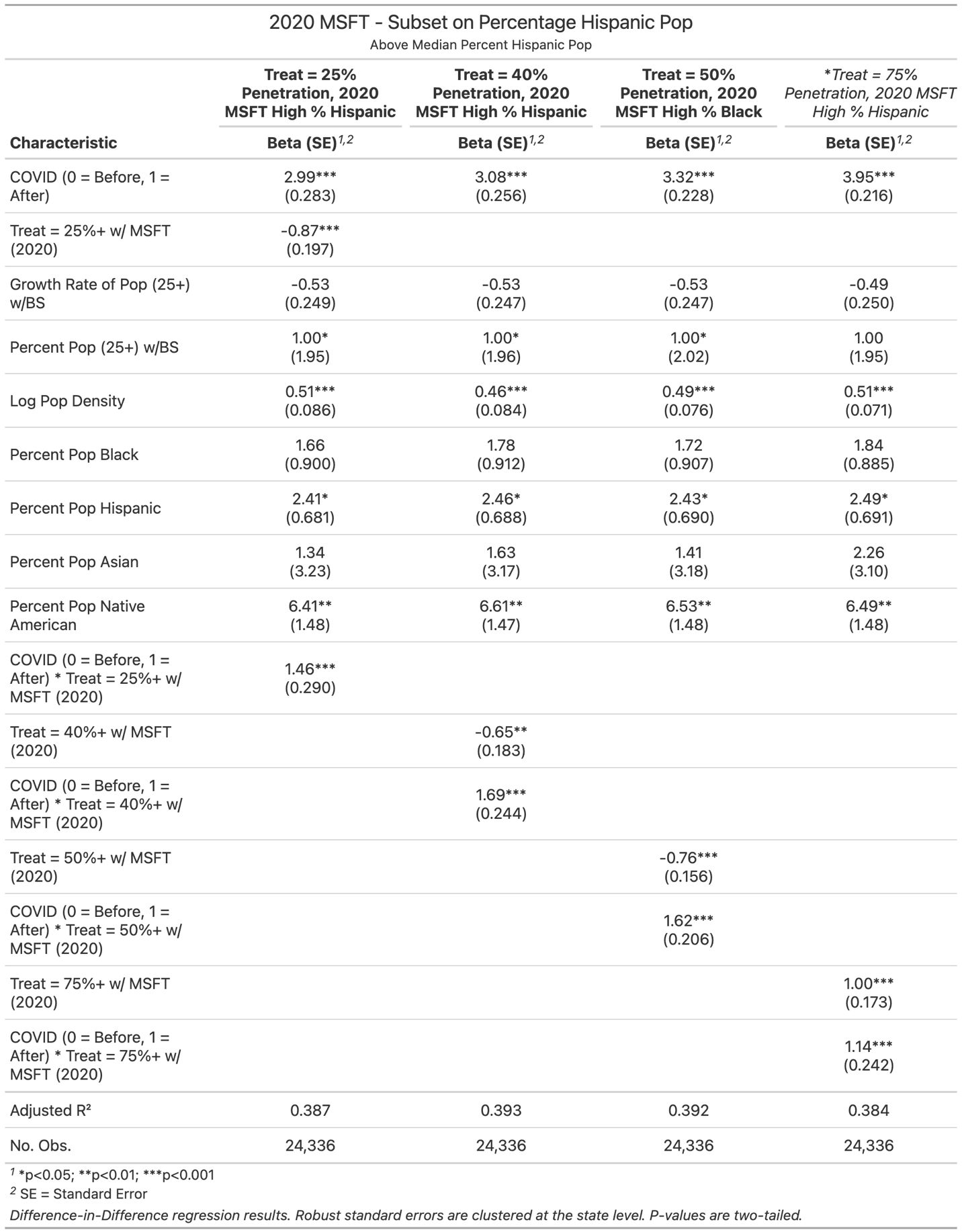
**

**Table I13: MSFT 2020 Below Median Percentage Hispanic**

**
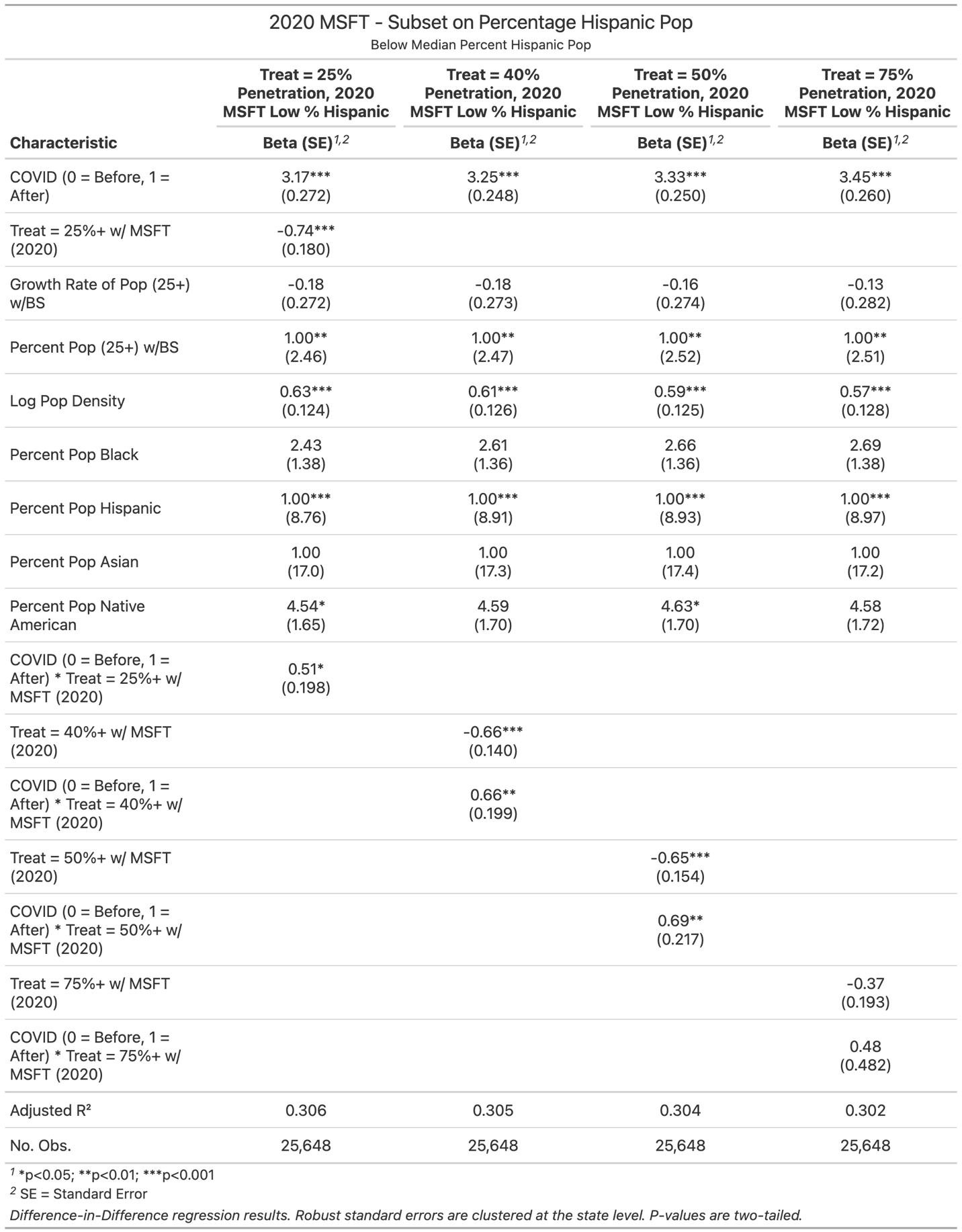
**

**Table I14: MSFT 2020 Above Median Percentage of Tech Industry**

**
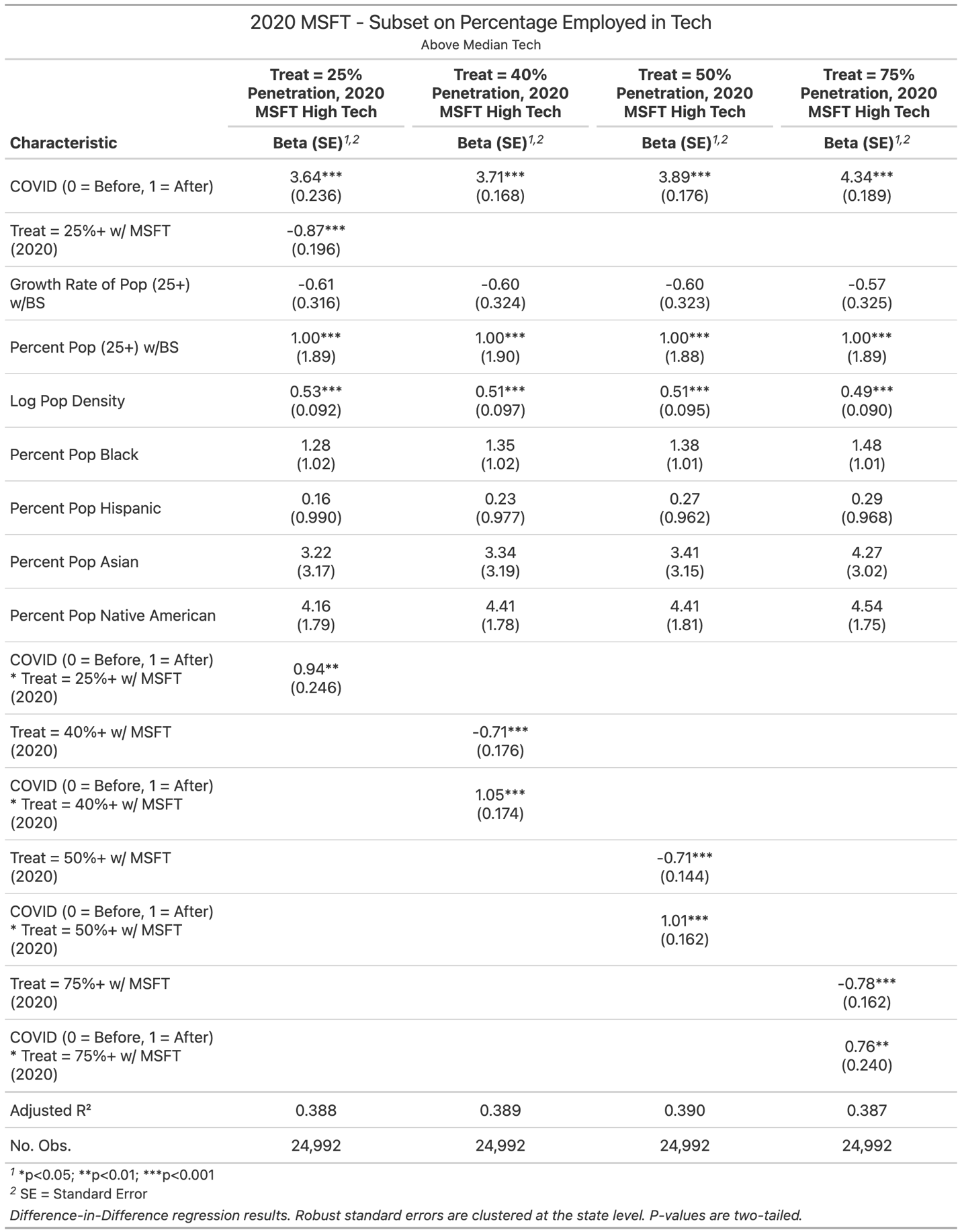
**

**Table I15: MSFT 2020 Below Median Percentage of Tech Industry**

**
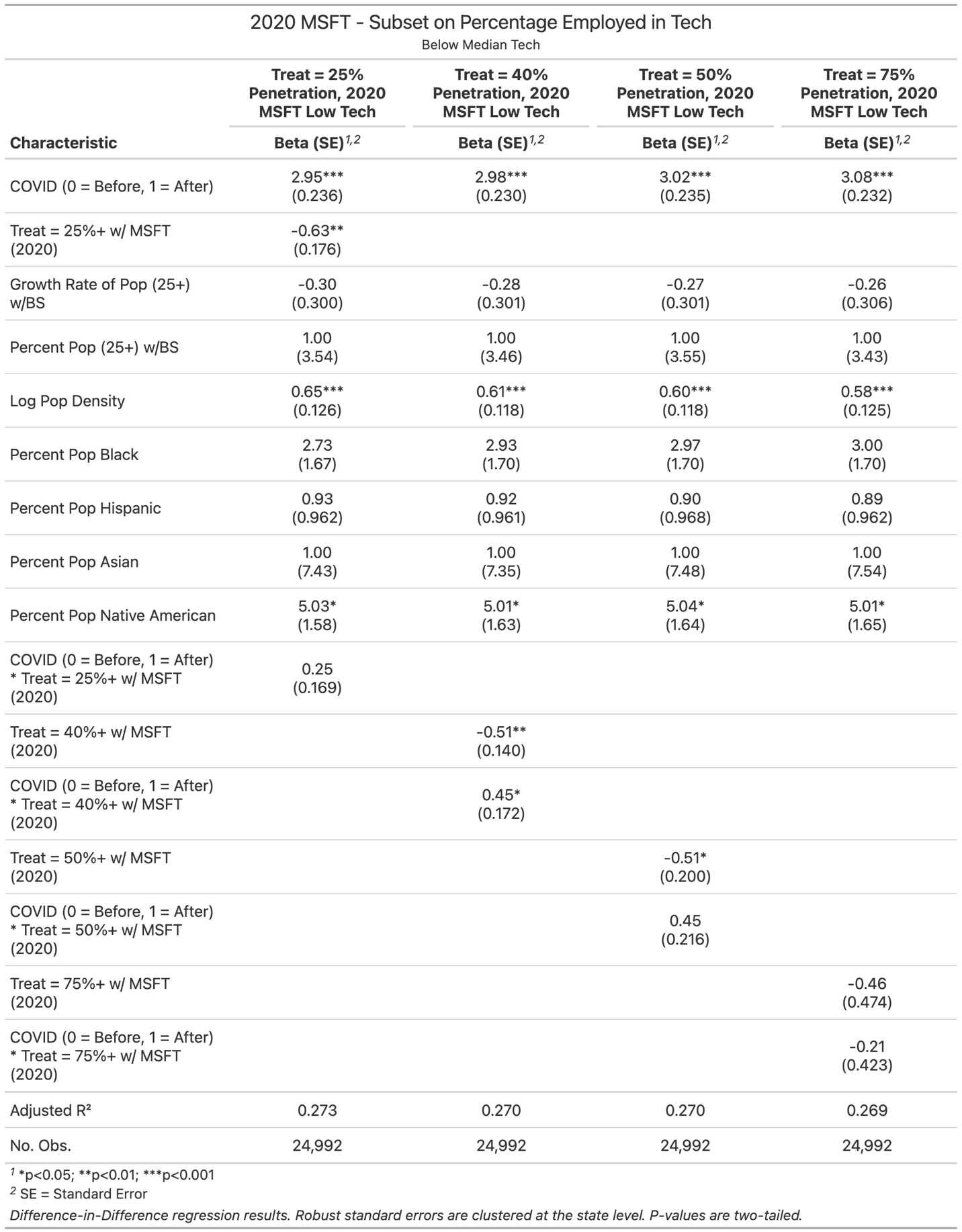
**

**Table I16: MSFT 2020 Above Median Number of Service Workers**

**
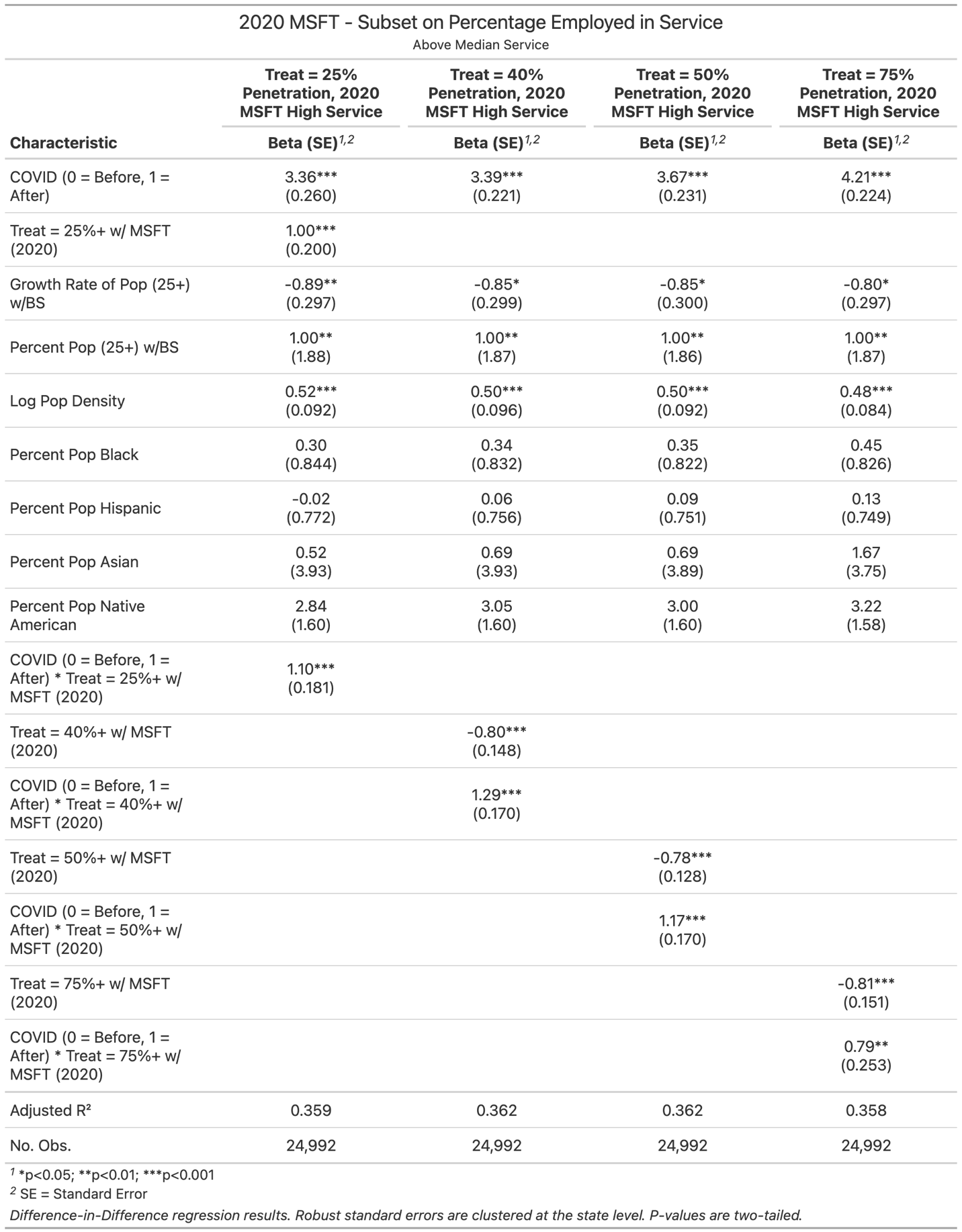
**

**Table I17: MSFT 2020 Below Median Number of Service Workers**

**
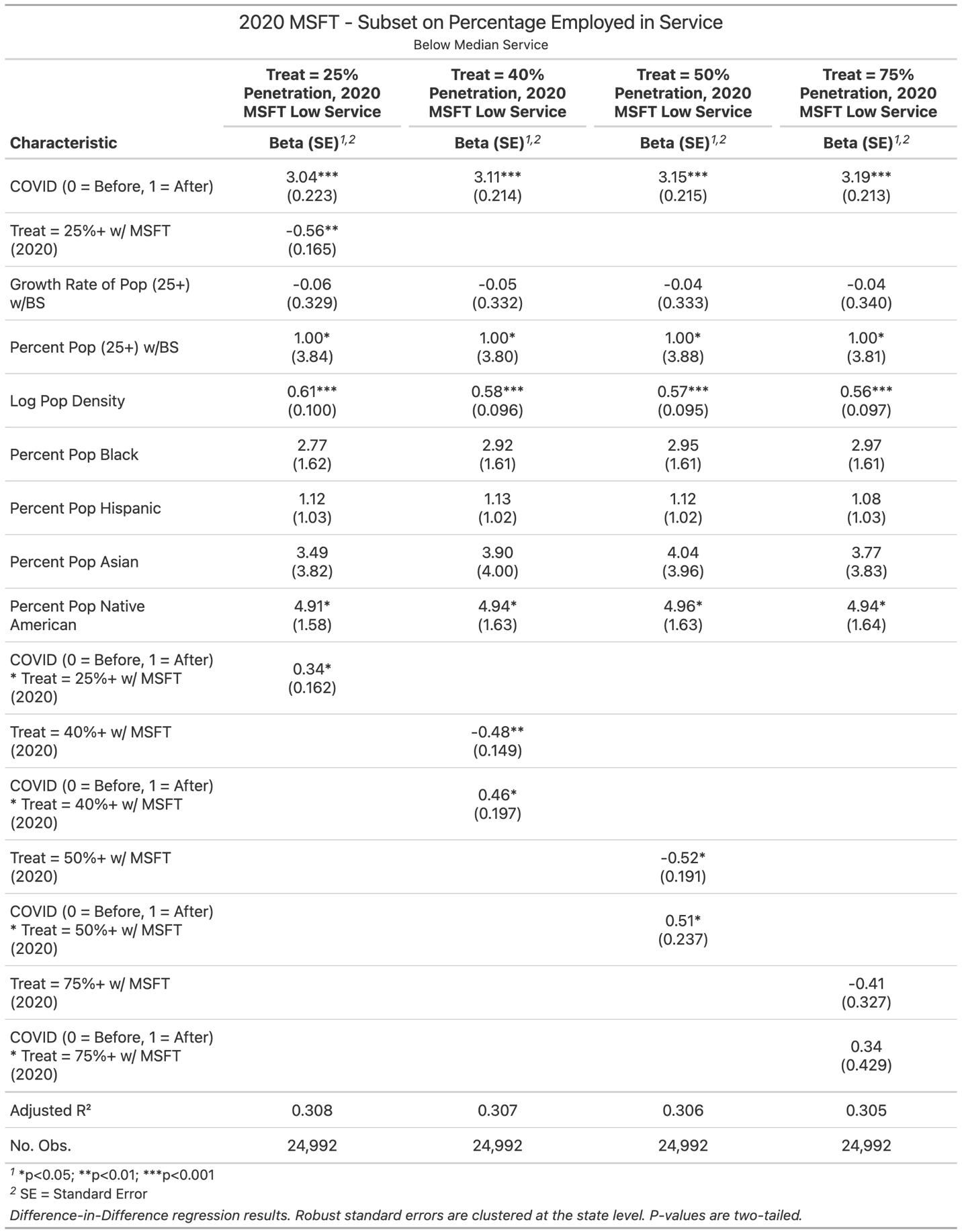
**

**Table I18: MSFT 2020 Above Median Number of Single Parents**

**
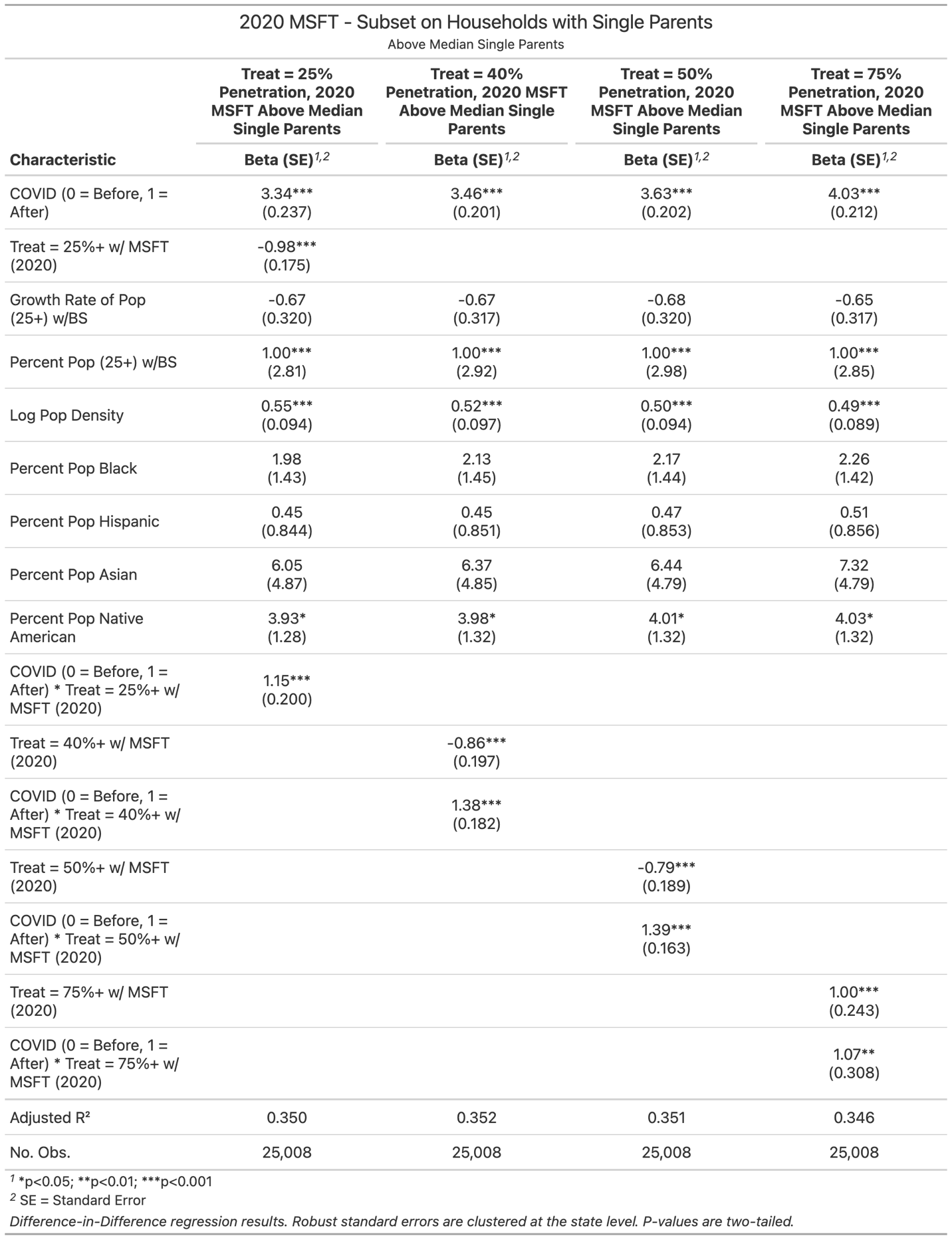
**

**Table I19: MSFT 2020 Below Median Number of Single Parents**

**
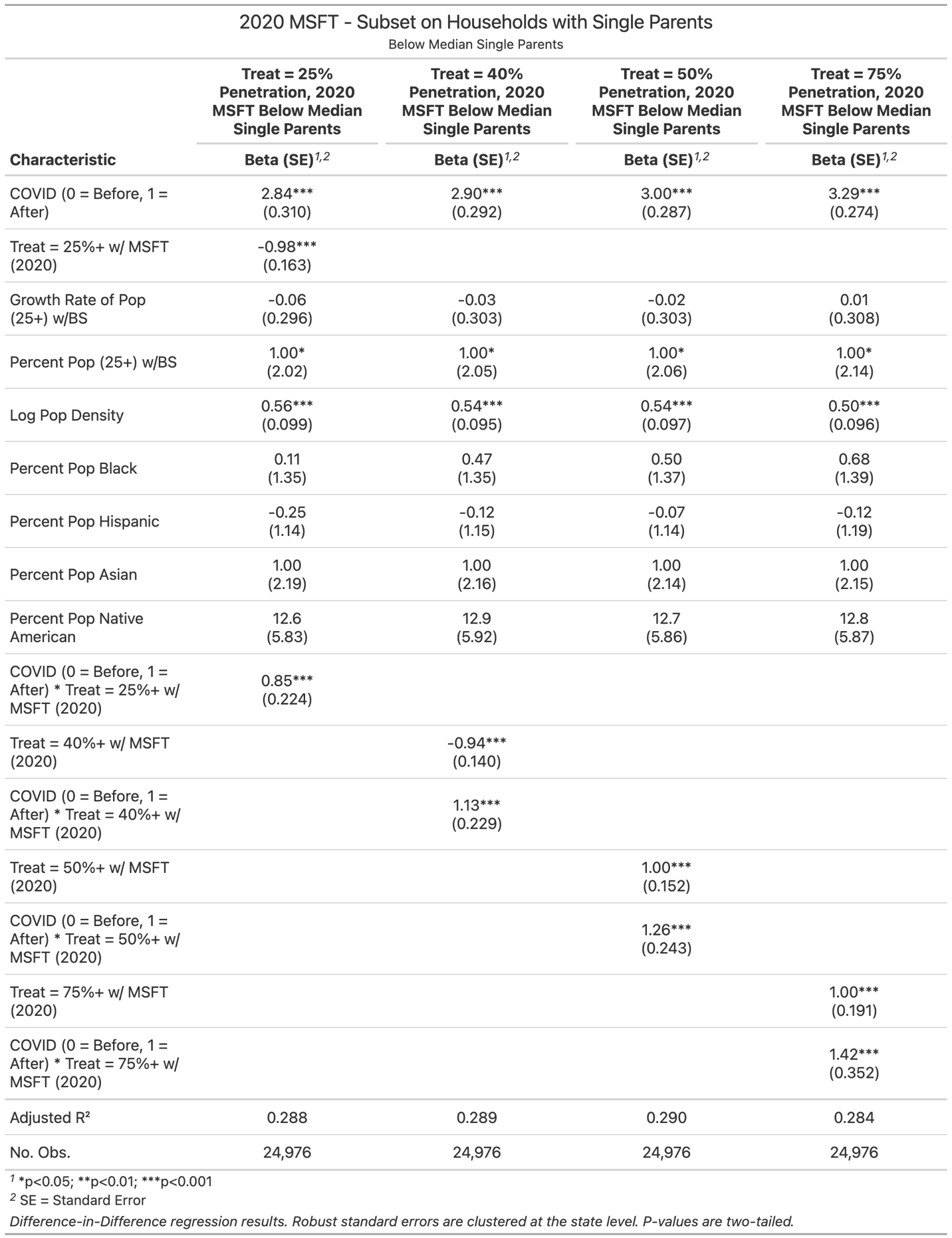
**

**Table I20: MSFT 2020 Above Median Number of Households with Children**


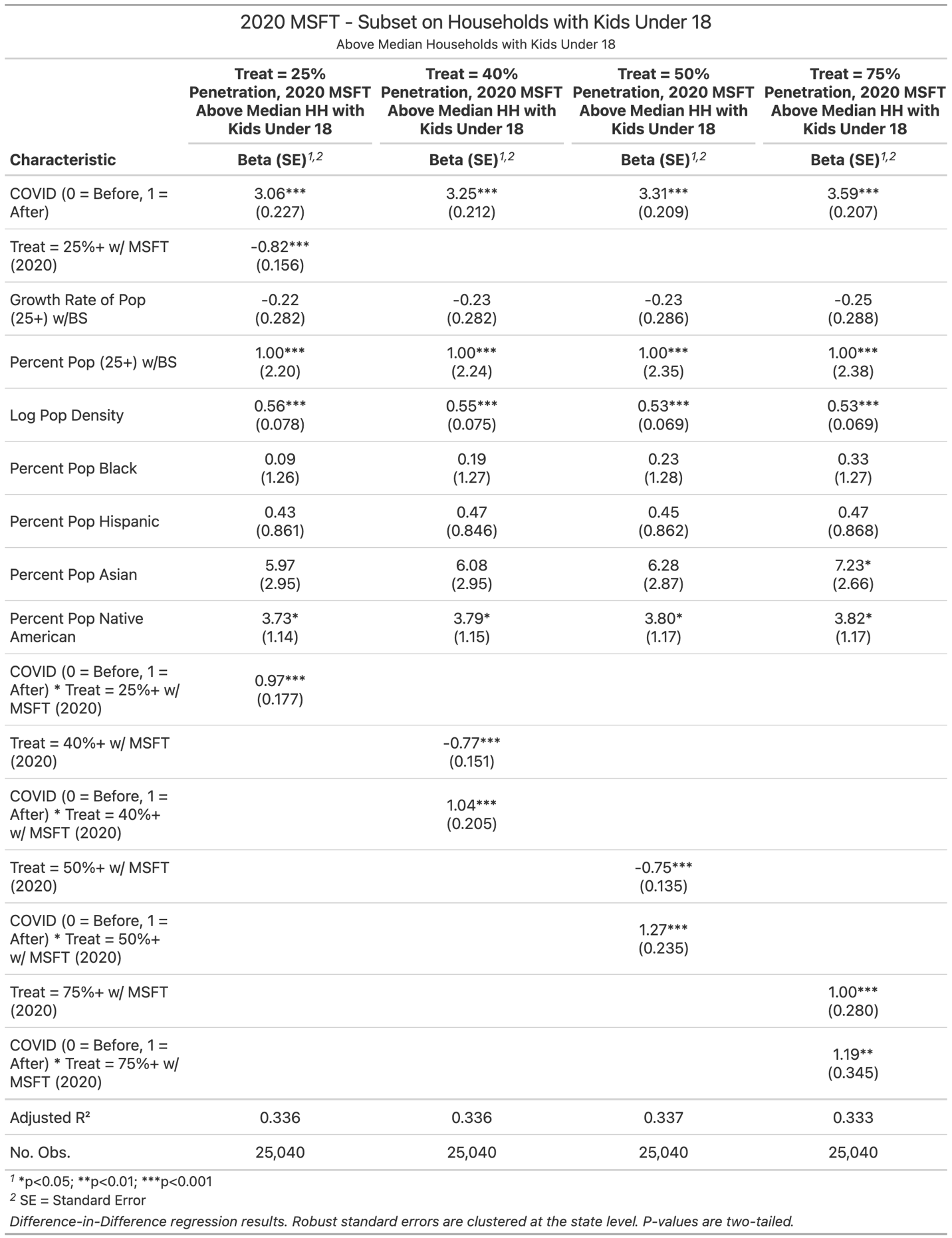


**Table I21: MSFT 2020 Below Median Number of Households with Children**

**
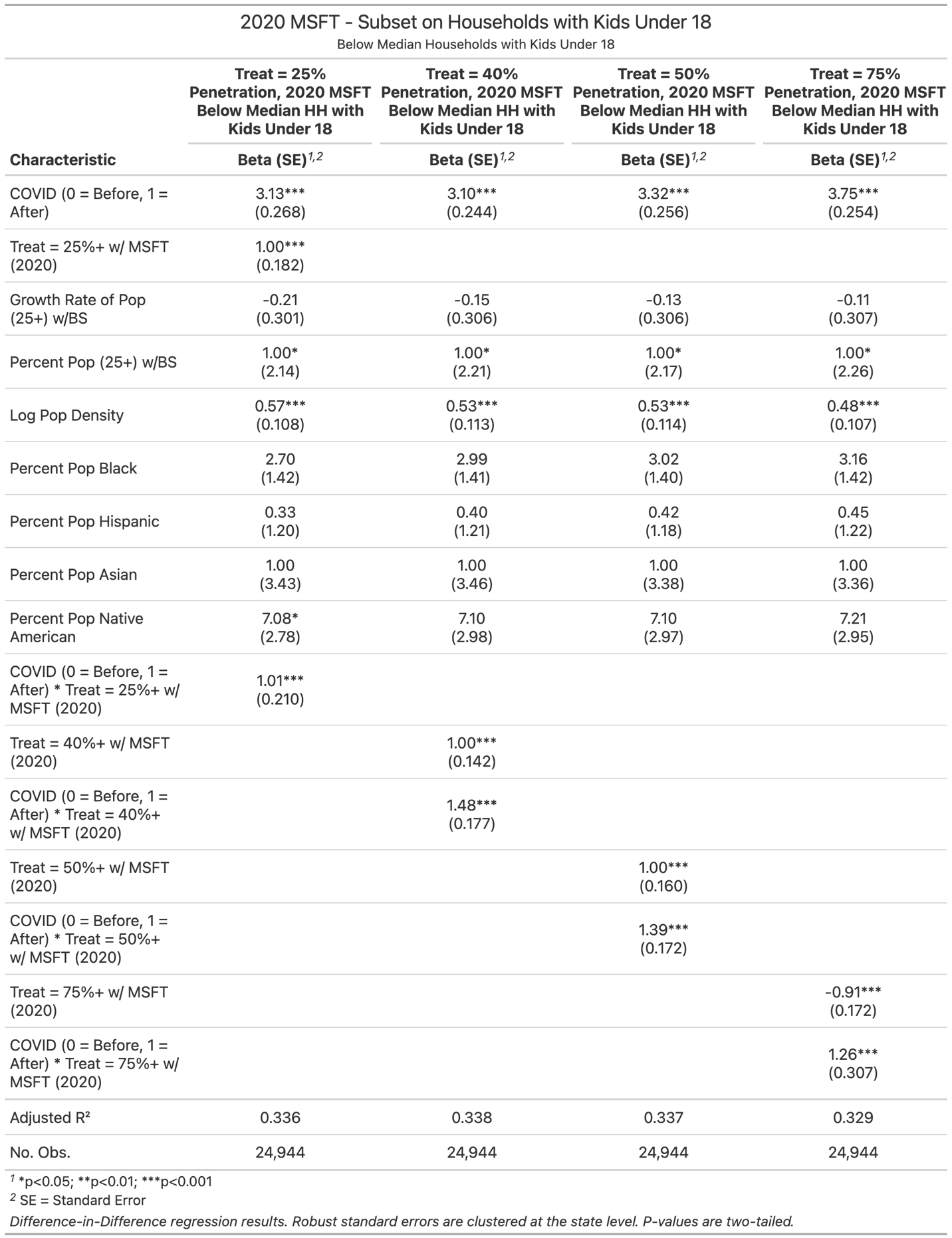
**

**Table I22: MSFT 2019 Urban**

**
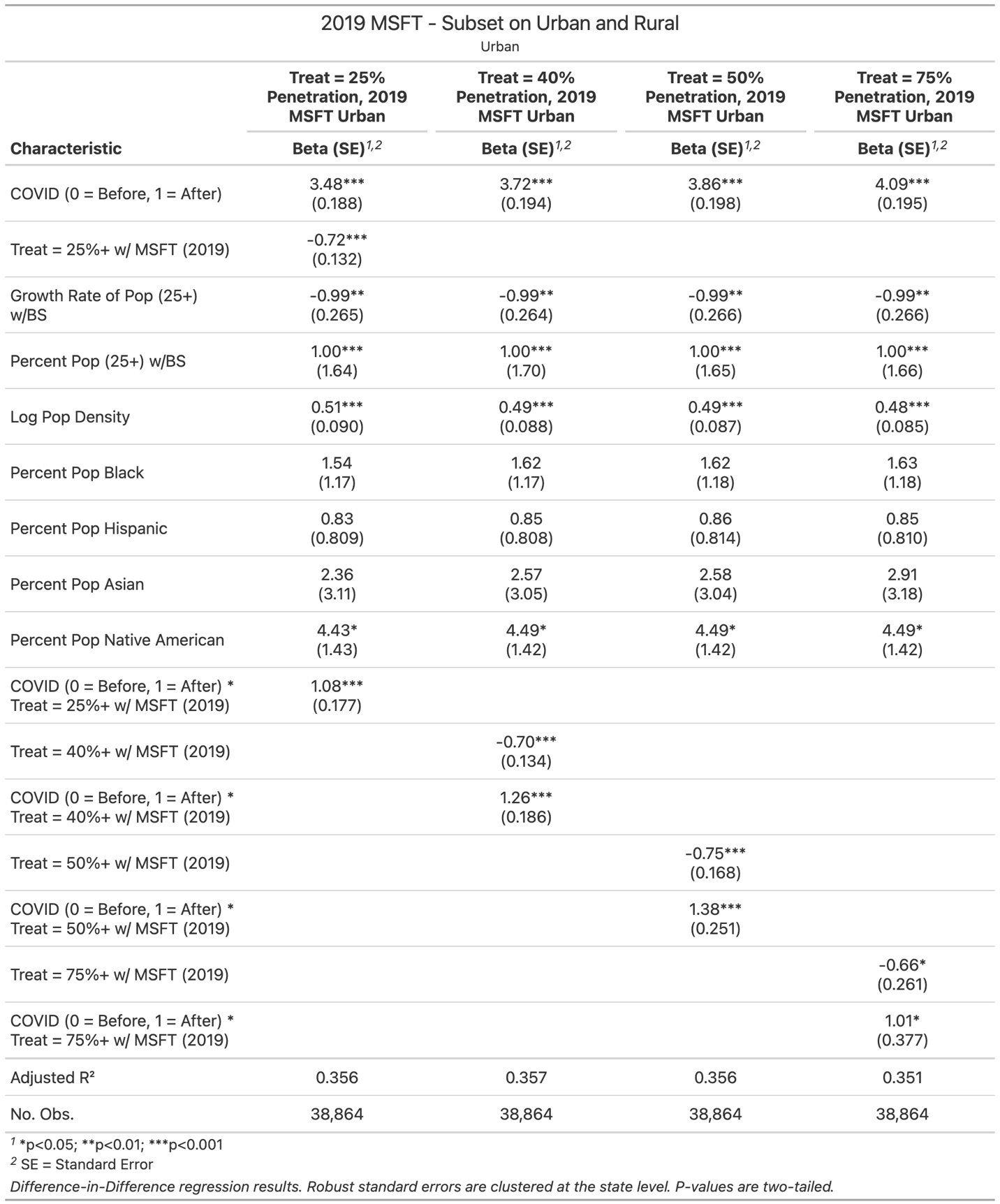
**

**Table I23: FCC 2019 Urban**

**
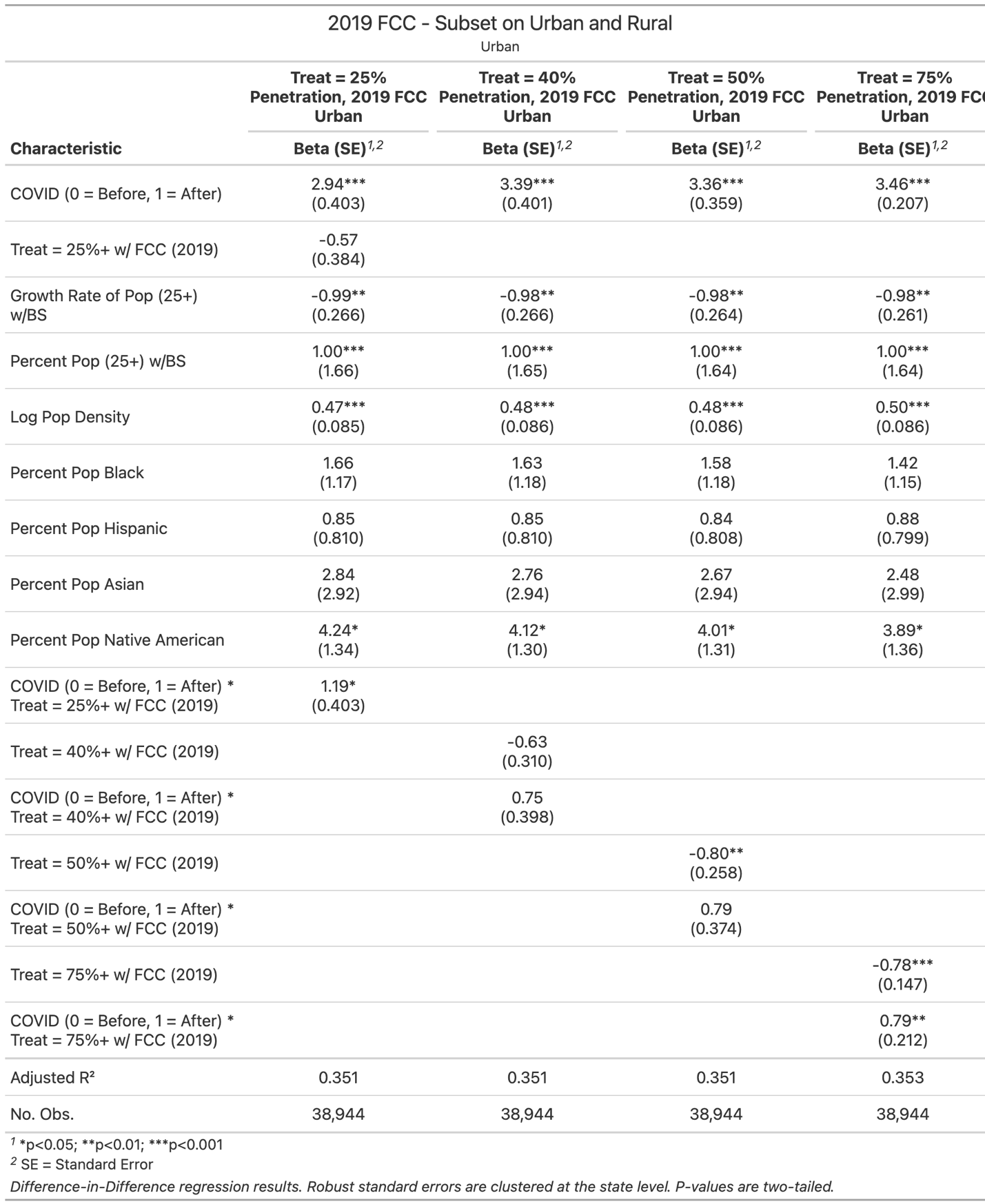
**

**Table I24: ACS 2020 Urban**

**
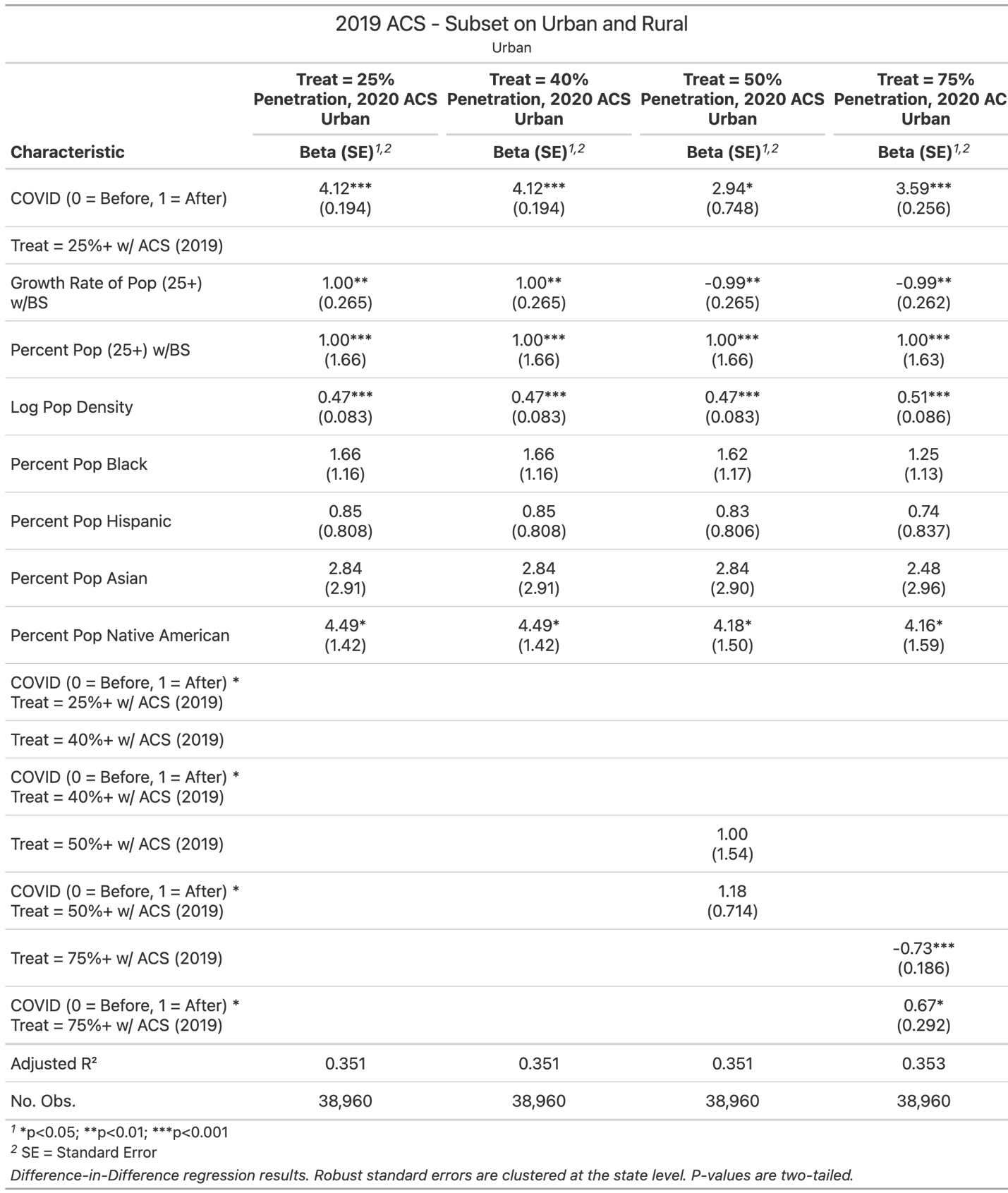
**

**Table I25: MSFT 2019 Rural**

**
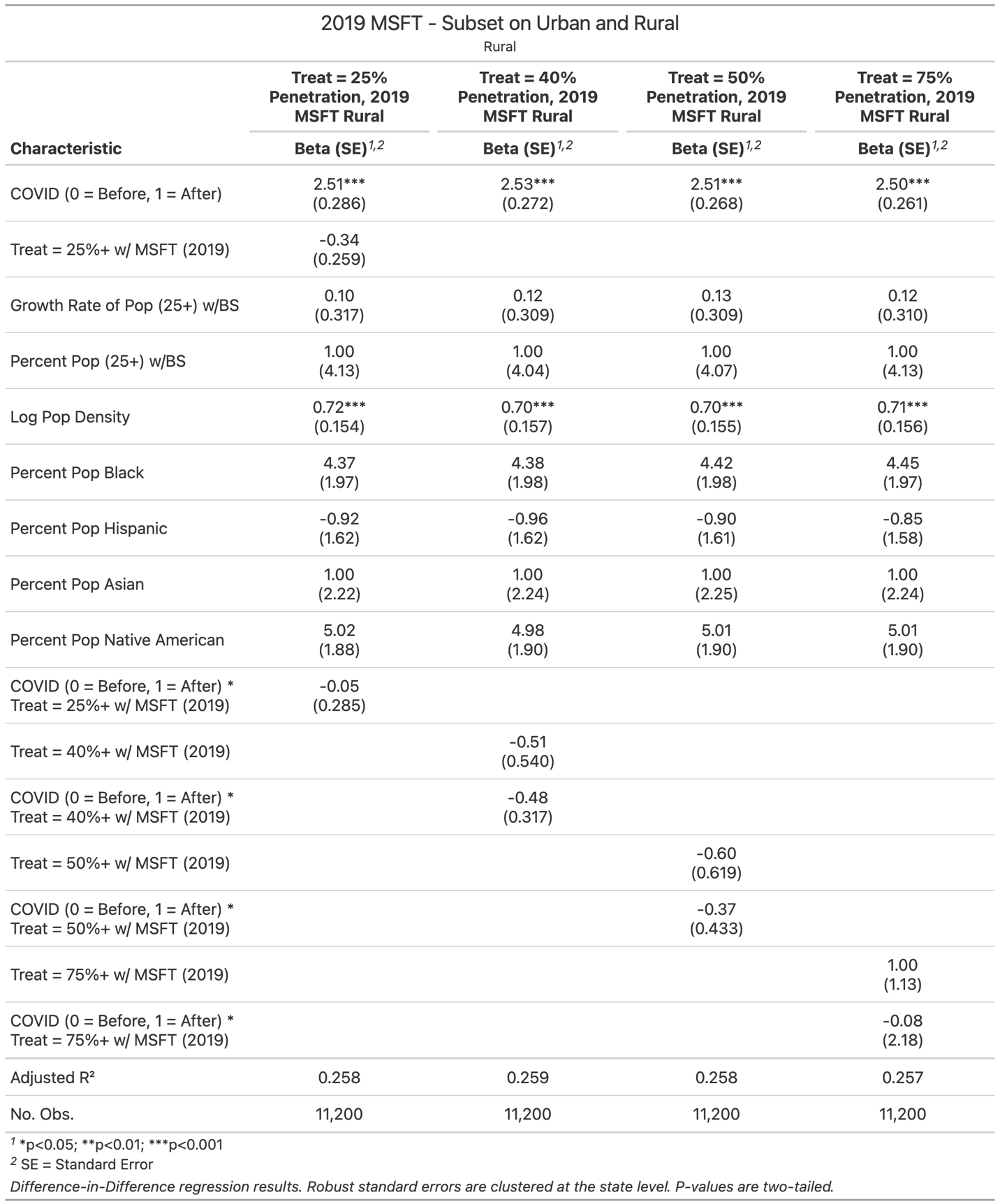
**

**Table I26: FCC 2019 Rural**

**
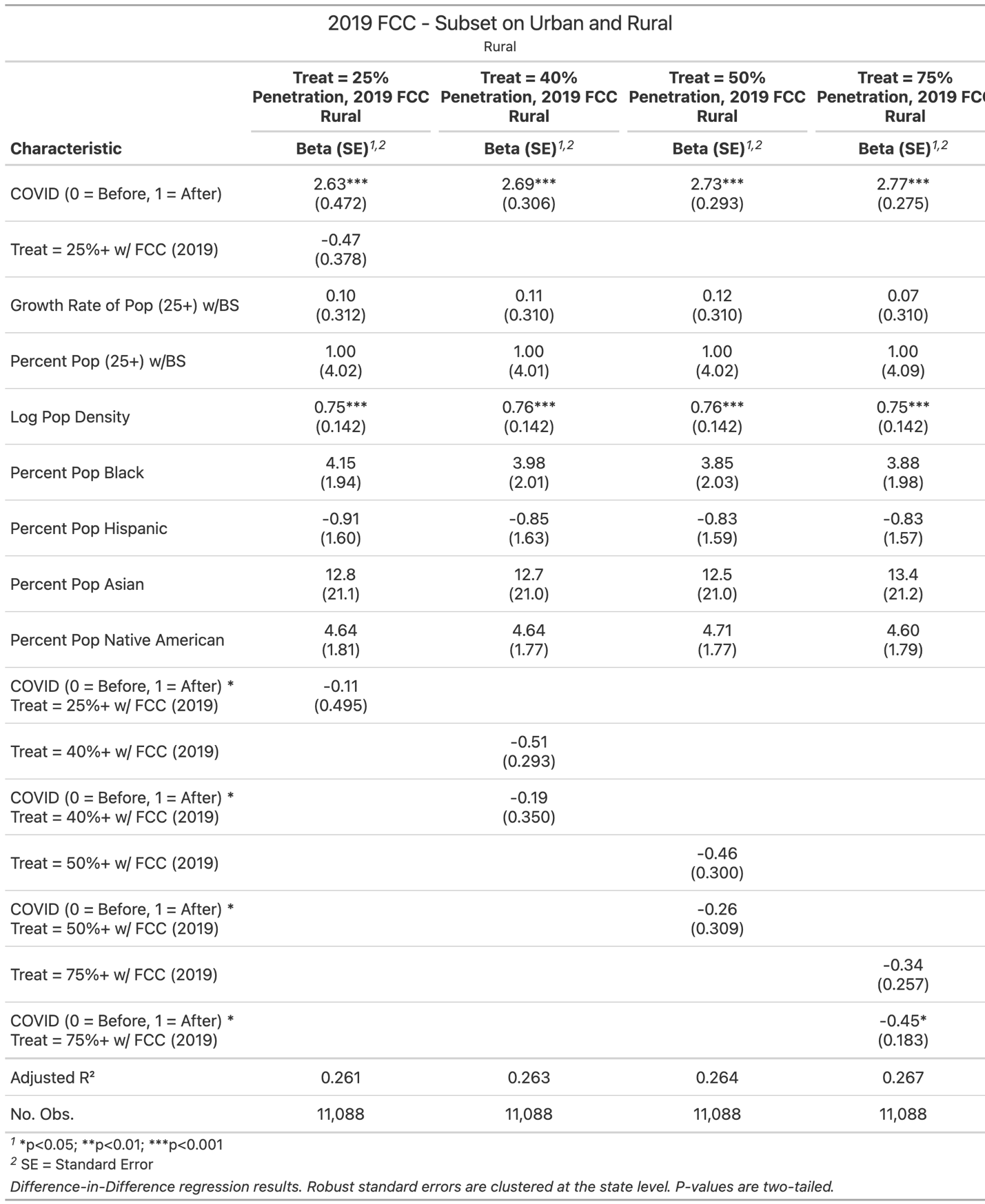
**

**Table I27: ACS 2020 Rural**

**
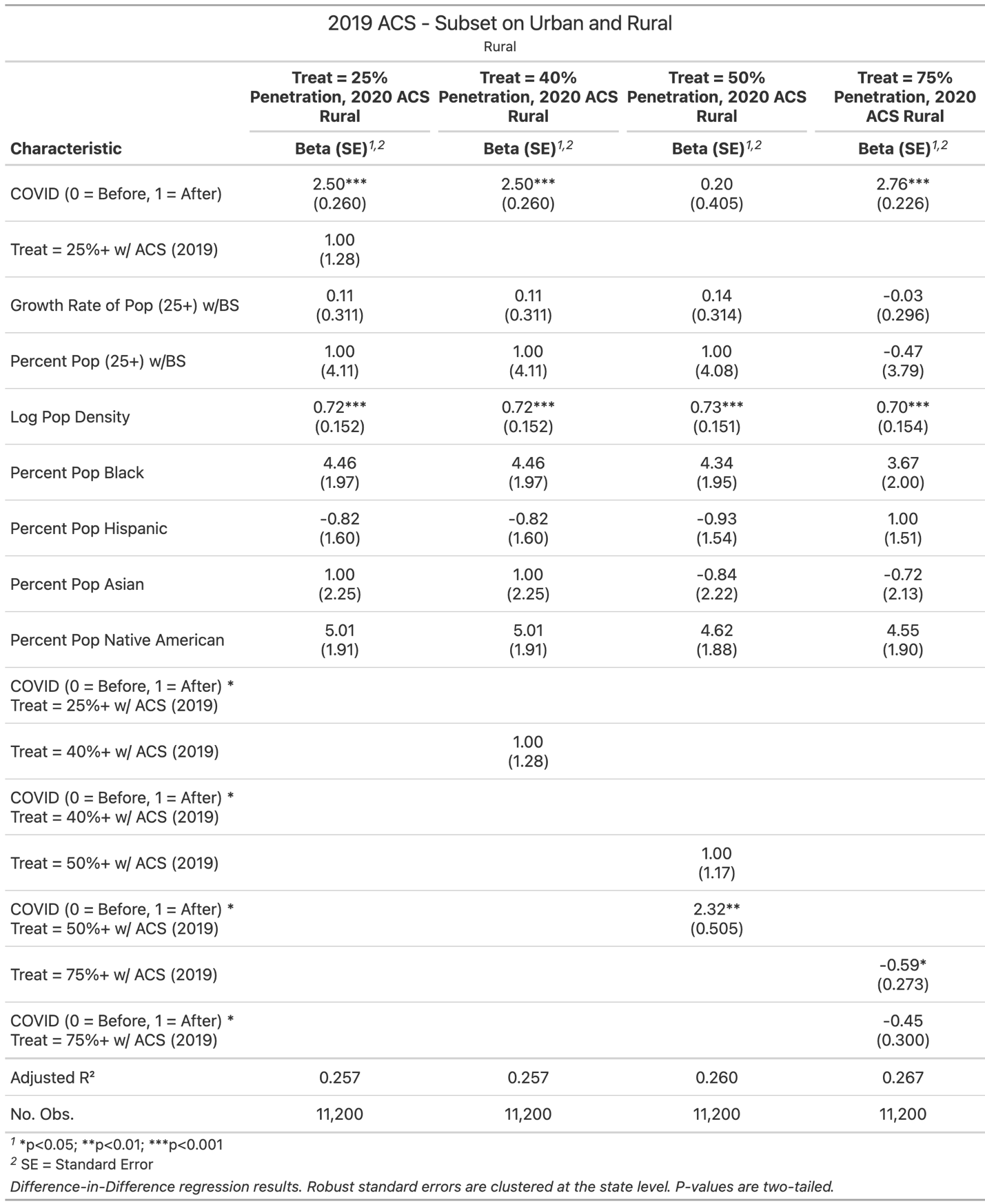
**

**Table I28: MSFT 2019 Above Median Number of Households with Children**

**
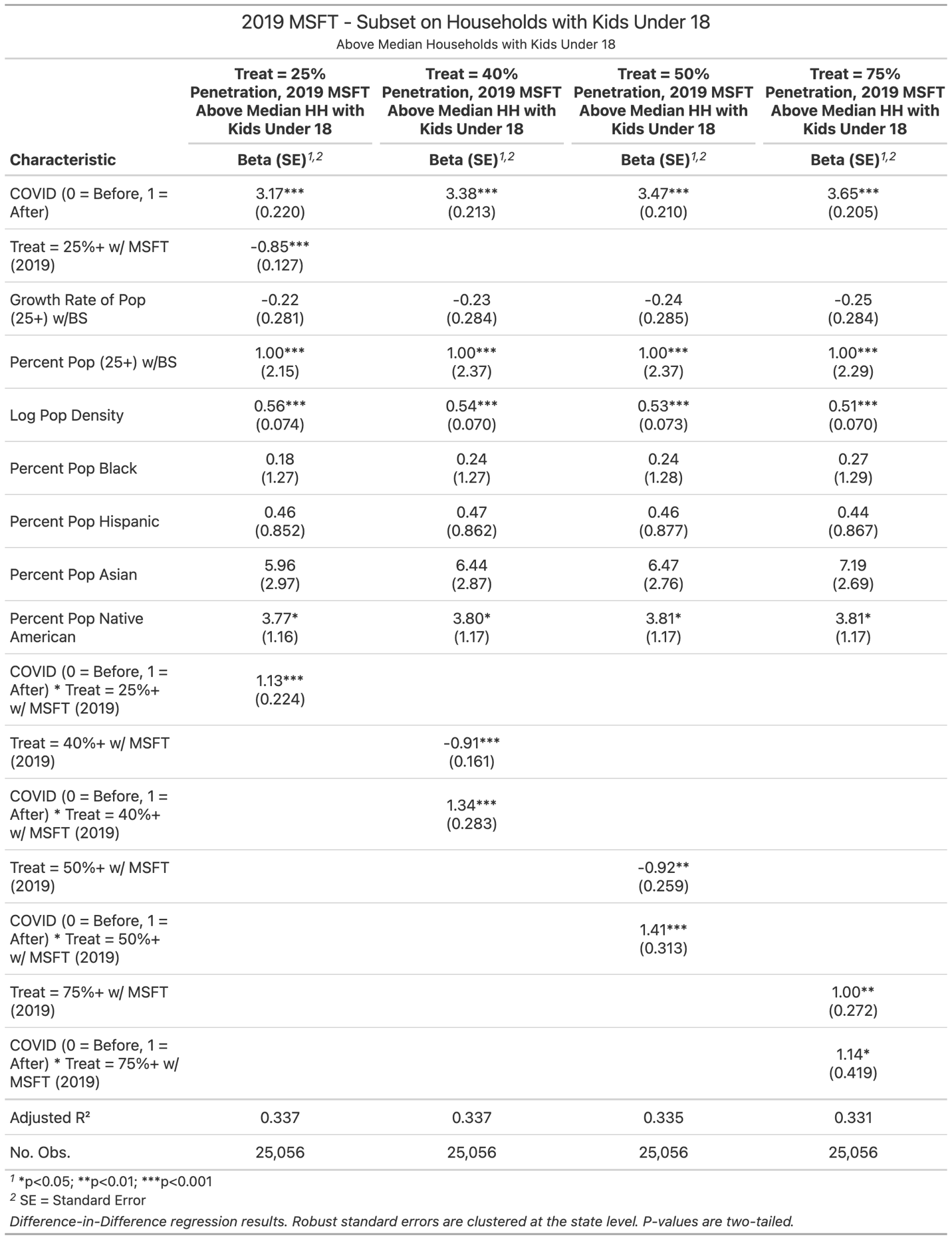
**

**Table I29: FCC 2019 Above Median Number of Households with Children**

**
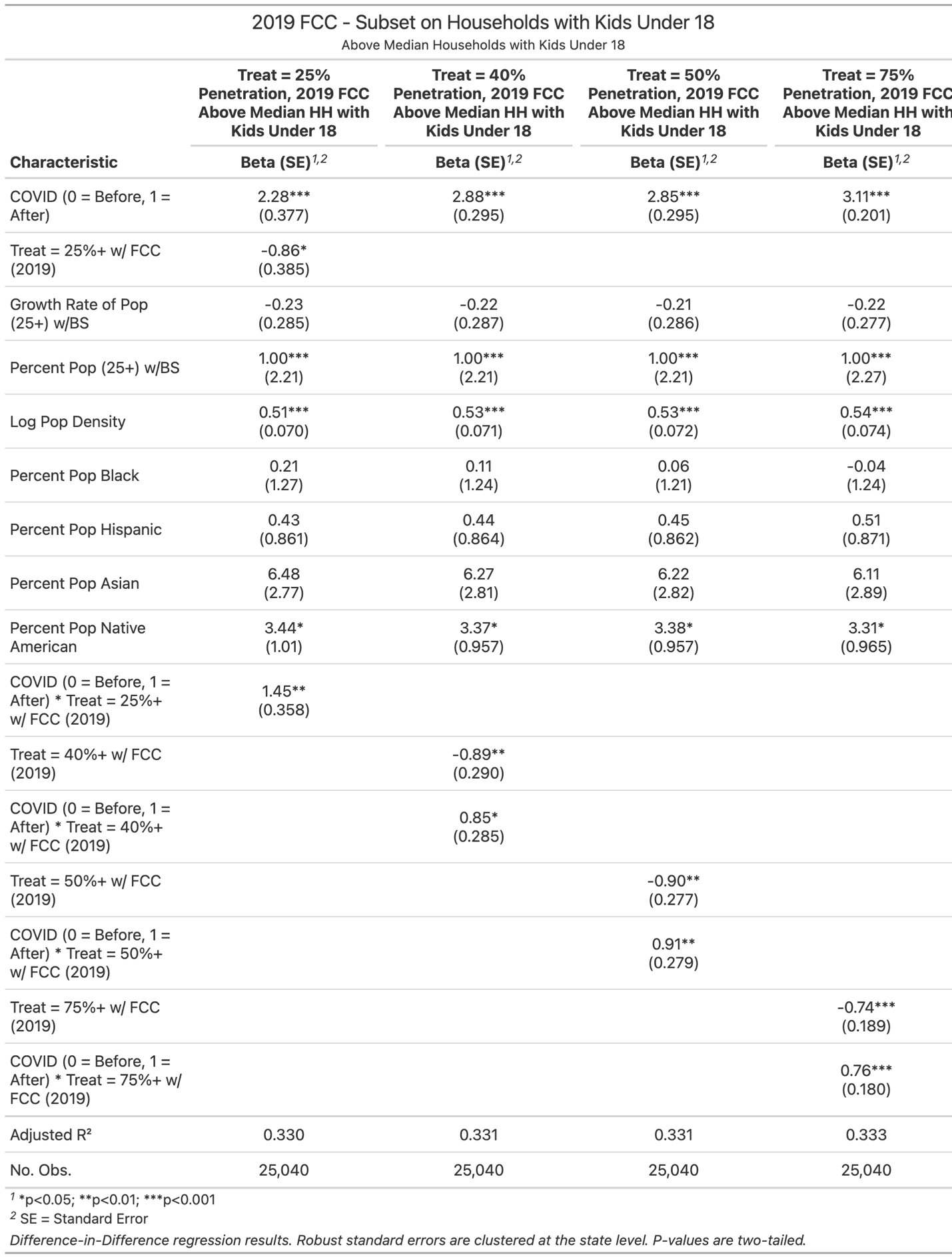
**

**Table I30: ACS 2020 Above Median Number of Households with Children**

**
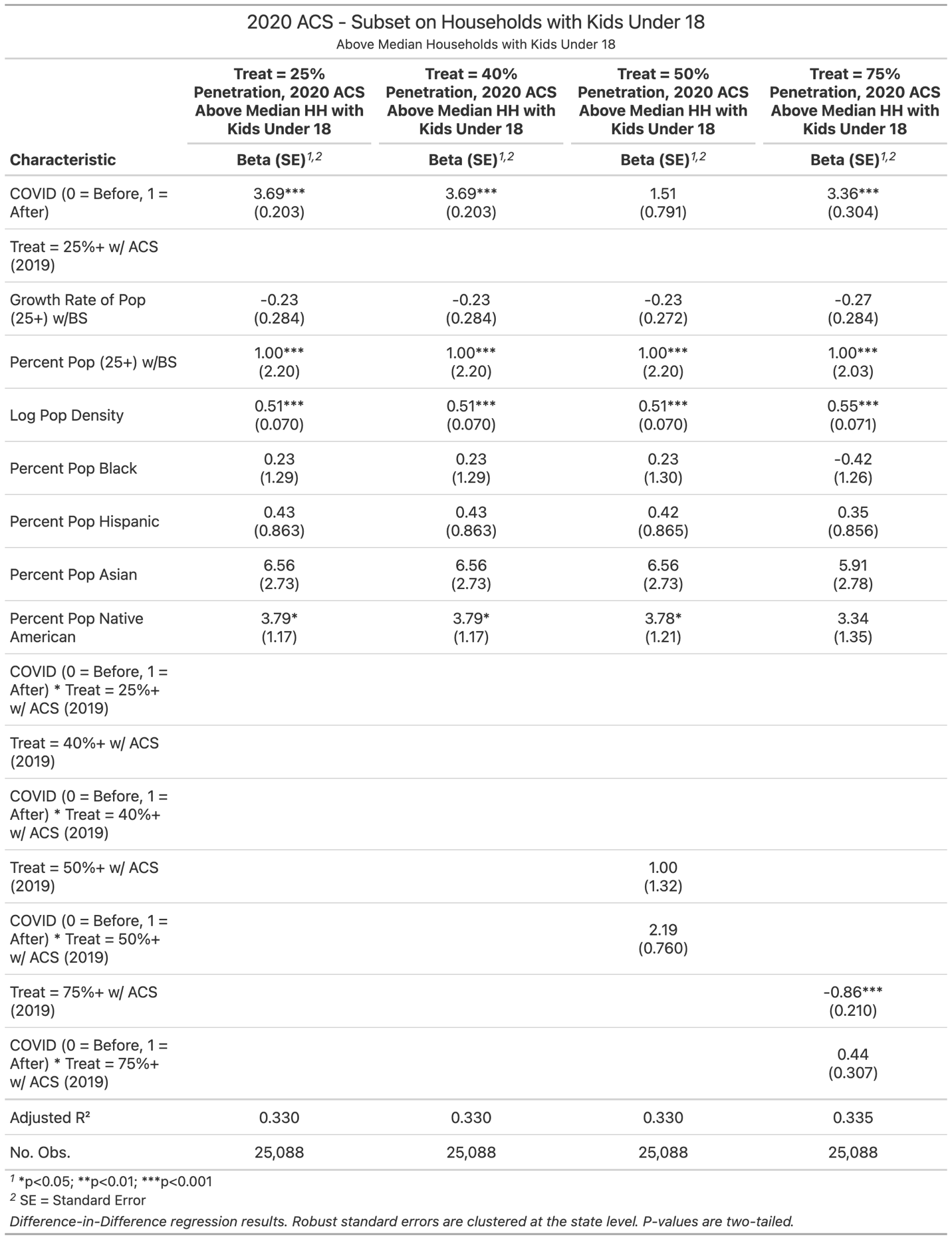
**

**Table I31: MSFT 2019 Above Median Number of Service Workers**

**
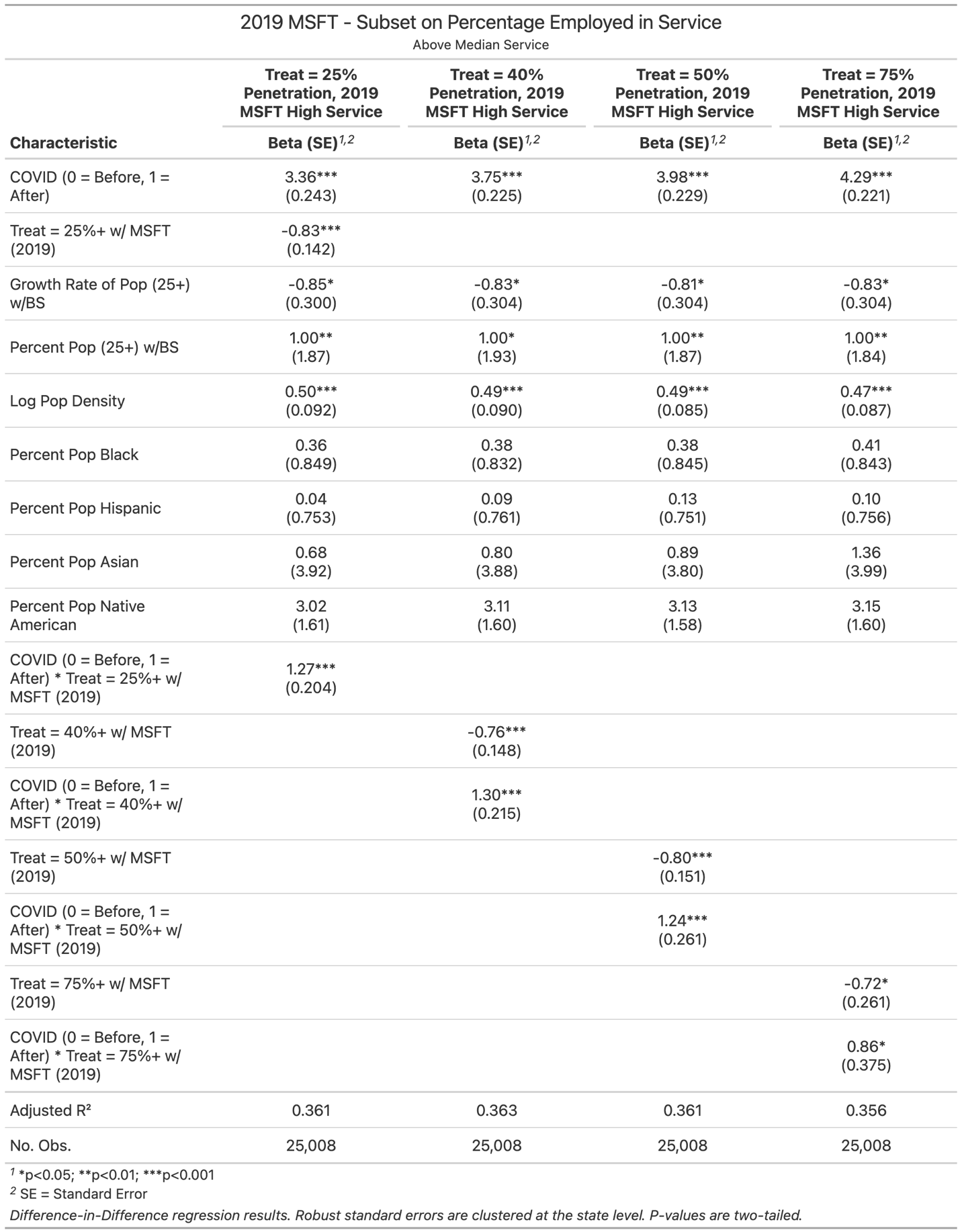
**

**Table I32: FCC 2019 Above Median Number of Service Workers**

**
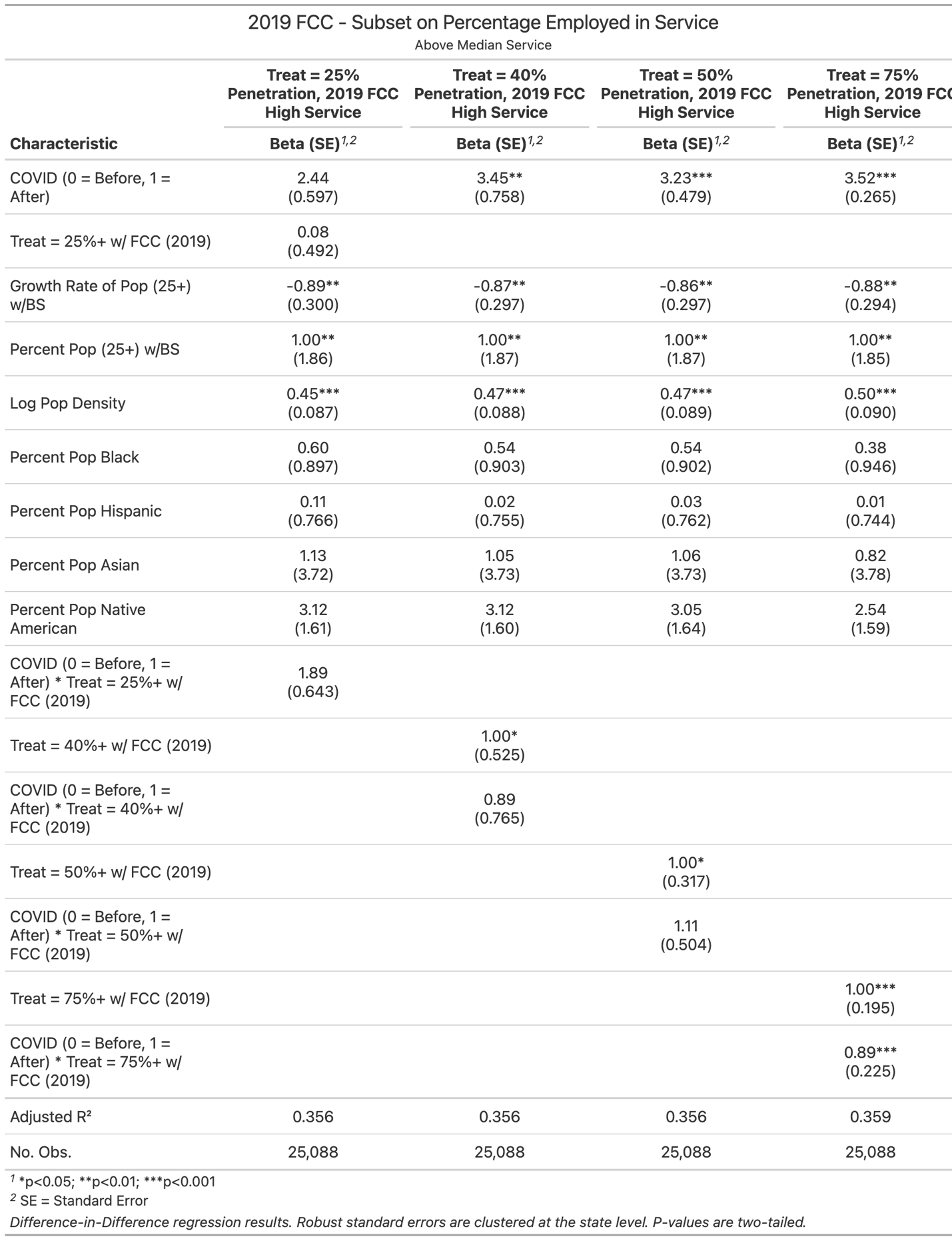
**

**Table I33: ACS 2020 Above Median Number of Service Workers**

**
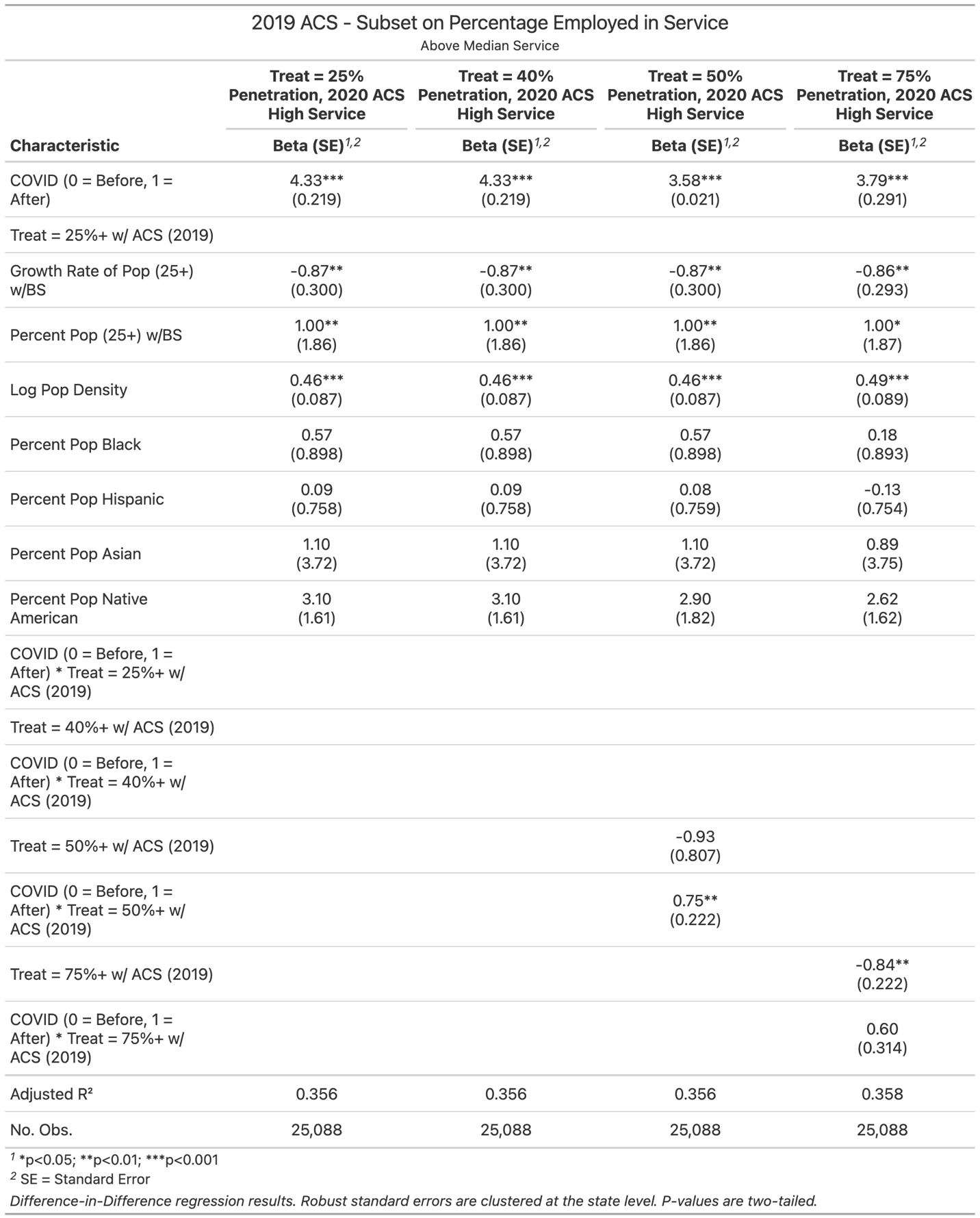
**

**Table I34: All Broadband Options Above Median People Who Can Work From Home**

**
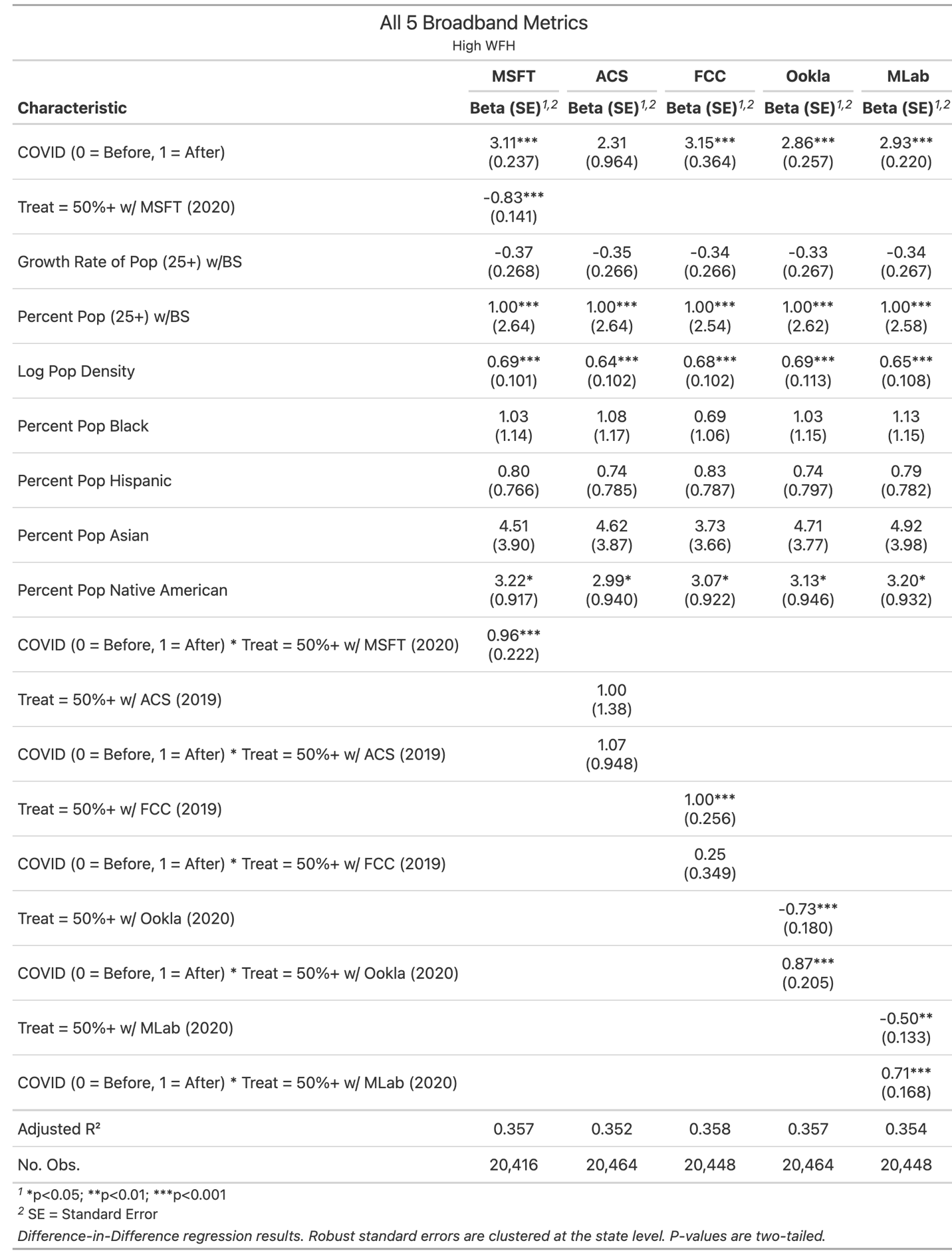
**

**Table I35: All Broadband Options Below Median People Who Can Work From Home**

**
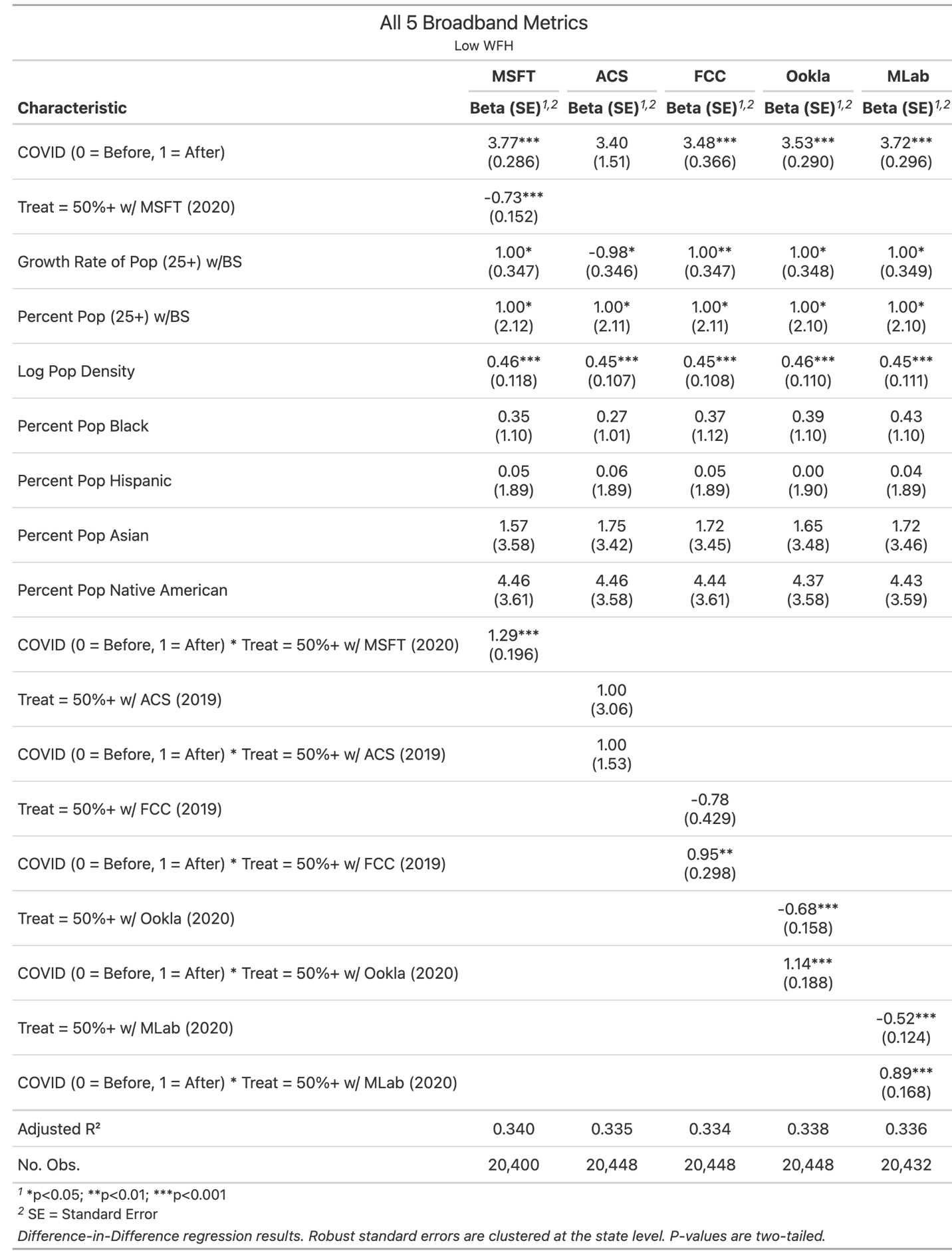
**

**Table I36: Ookla 2020 CT Penetration**

**
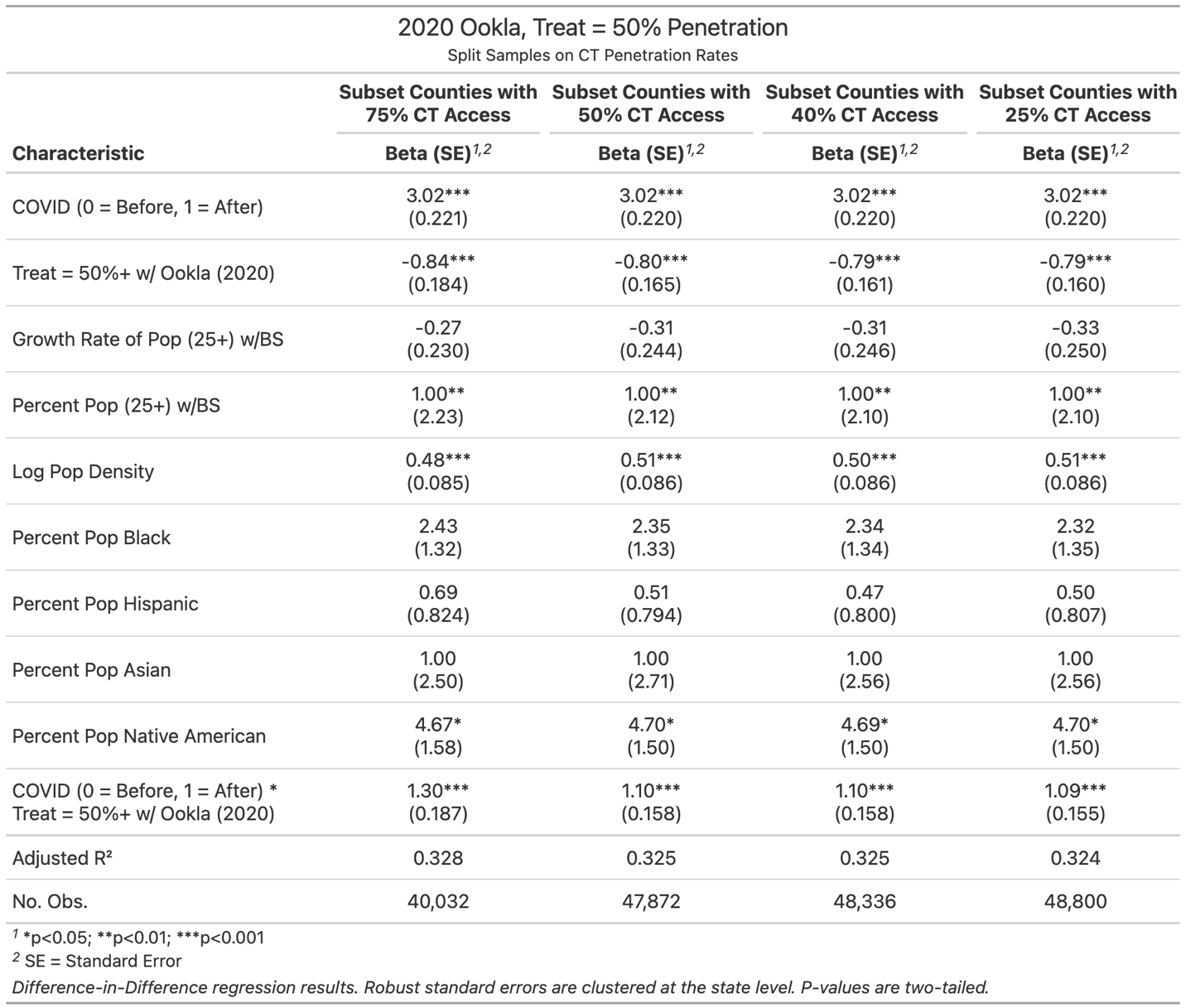
**

**Table I37: MSFT 2020 Above Median Buildings**

**
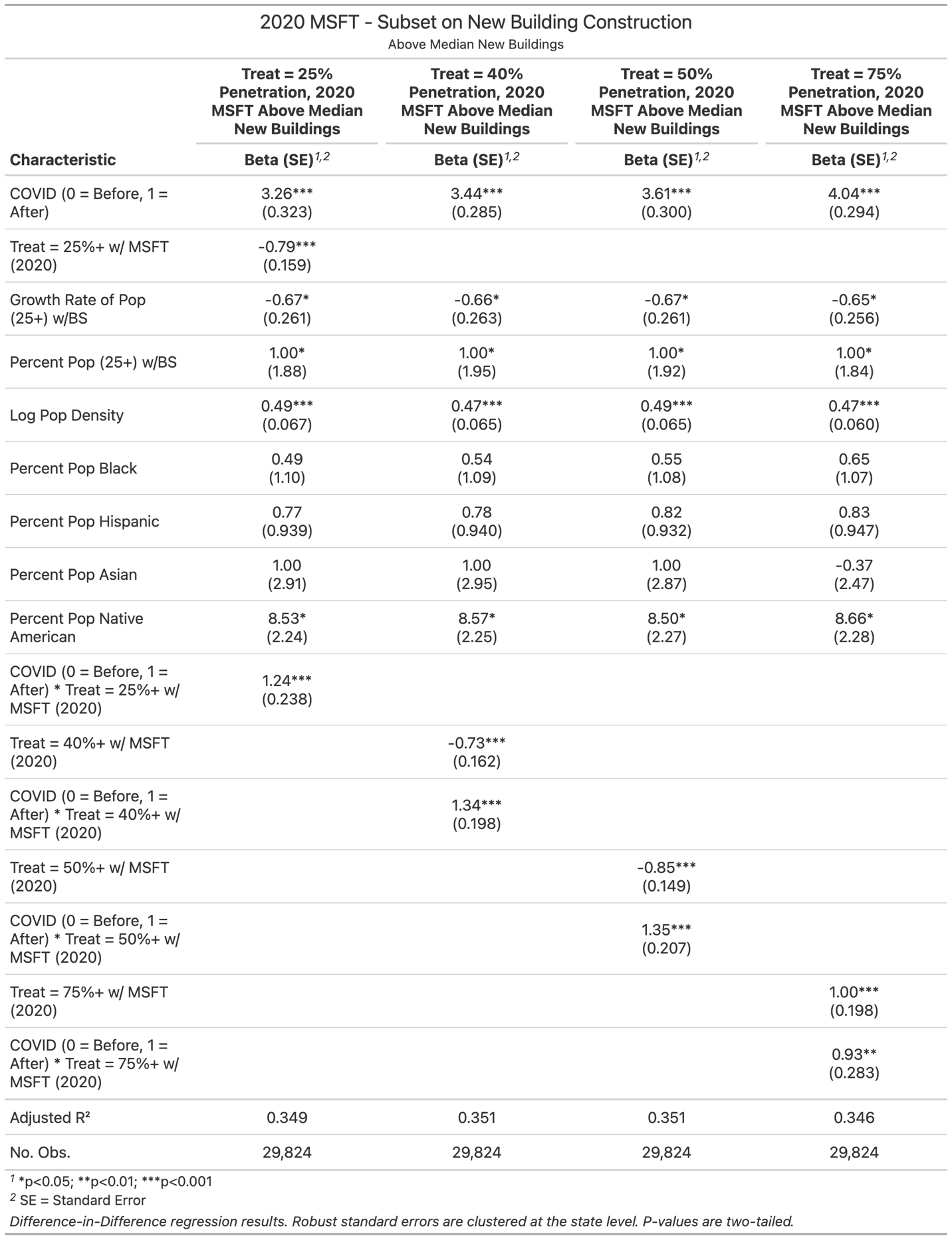
**

**Table I38: MSFT 2020 Below Median Buildings**

**
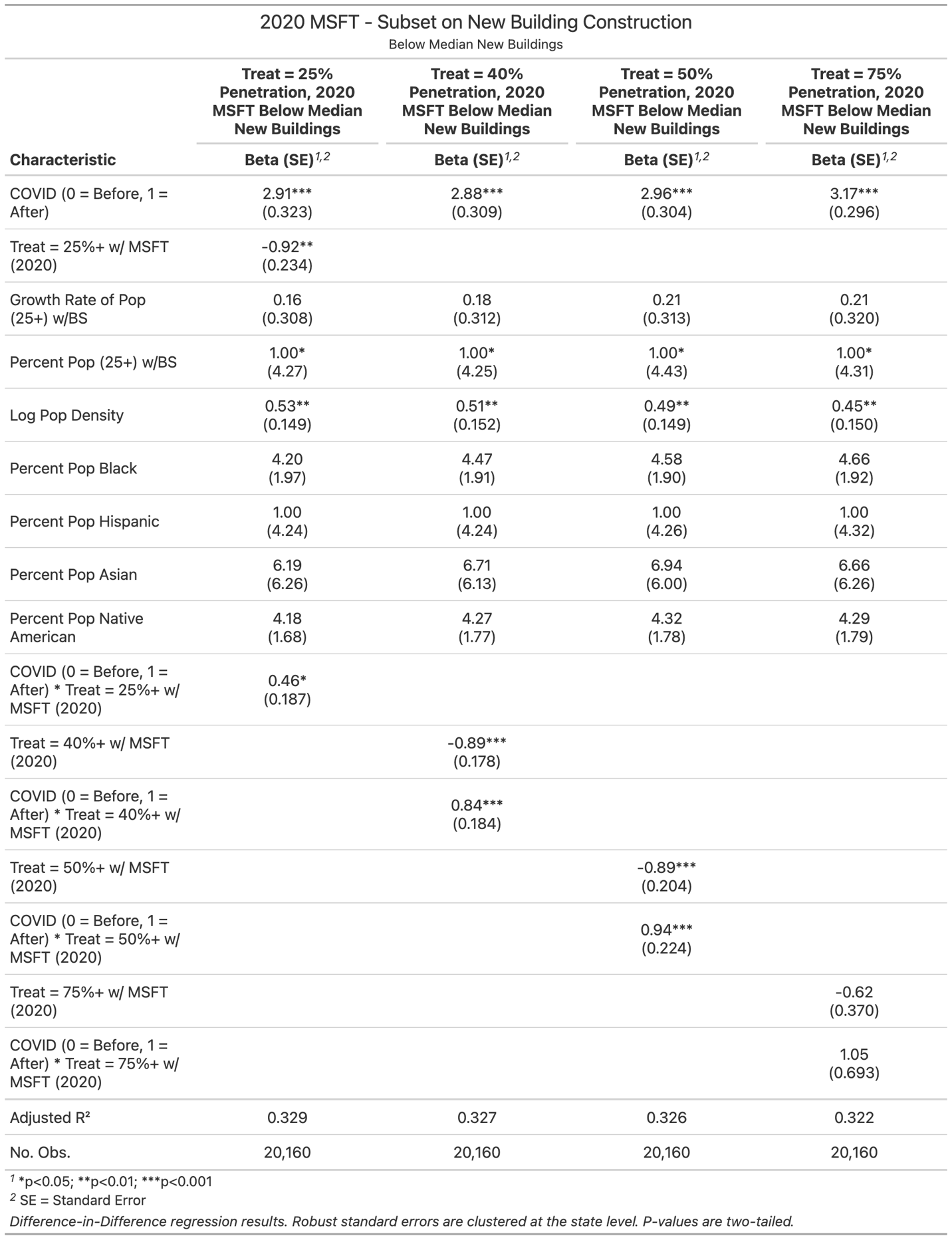
**

**Table I39: MSFT 2020 Above Median Bridges**

**
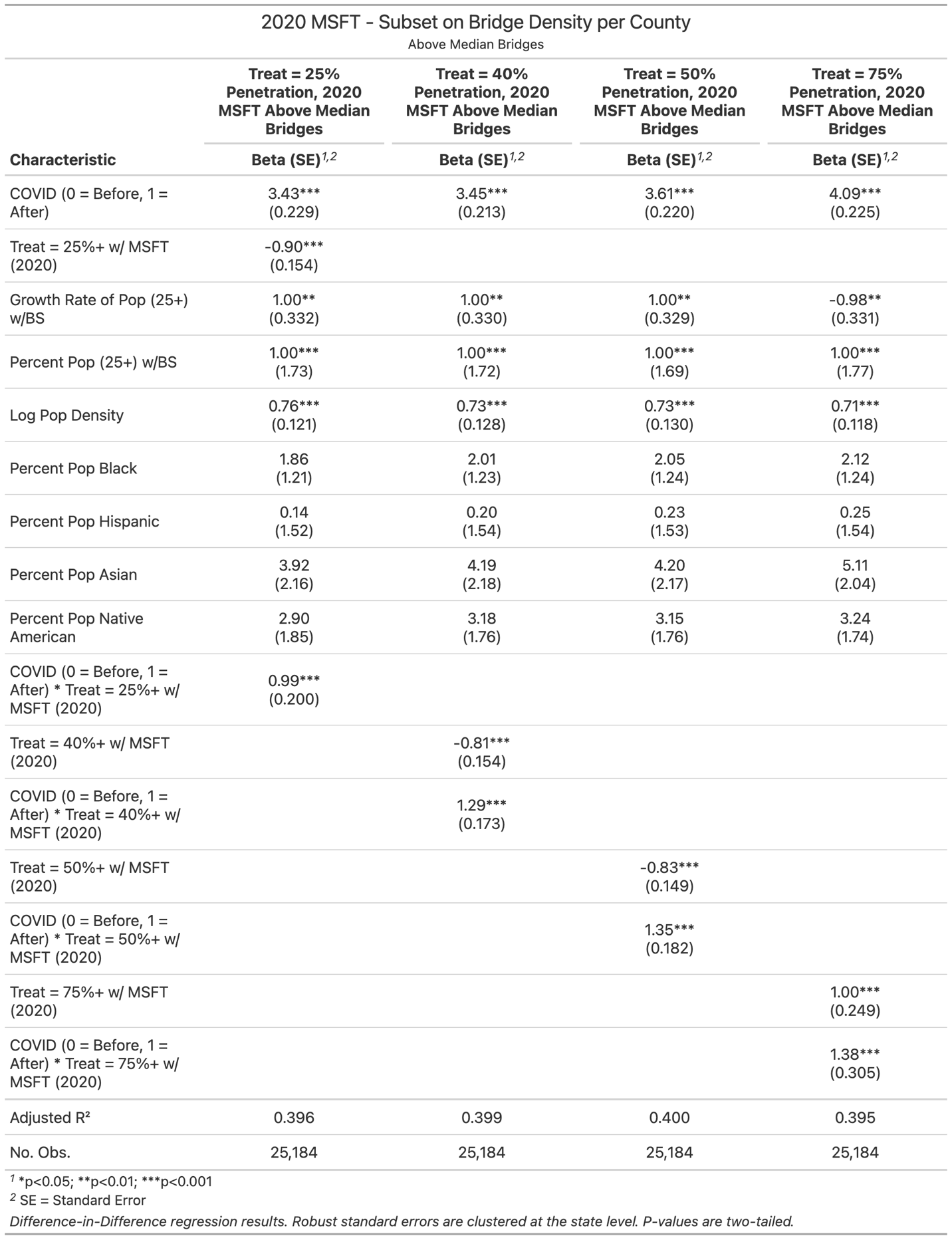
**

**Table I40: MSFT 2020 Below Median Bridges**

**
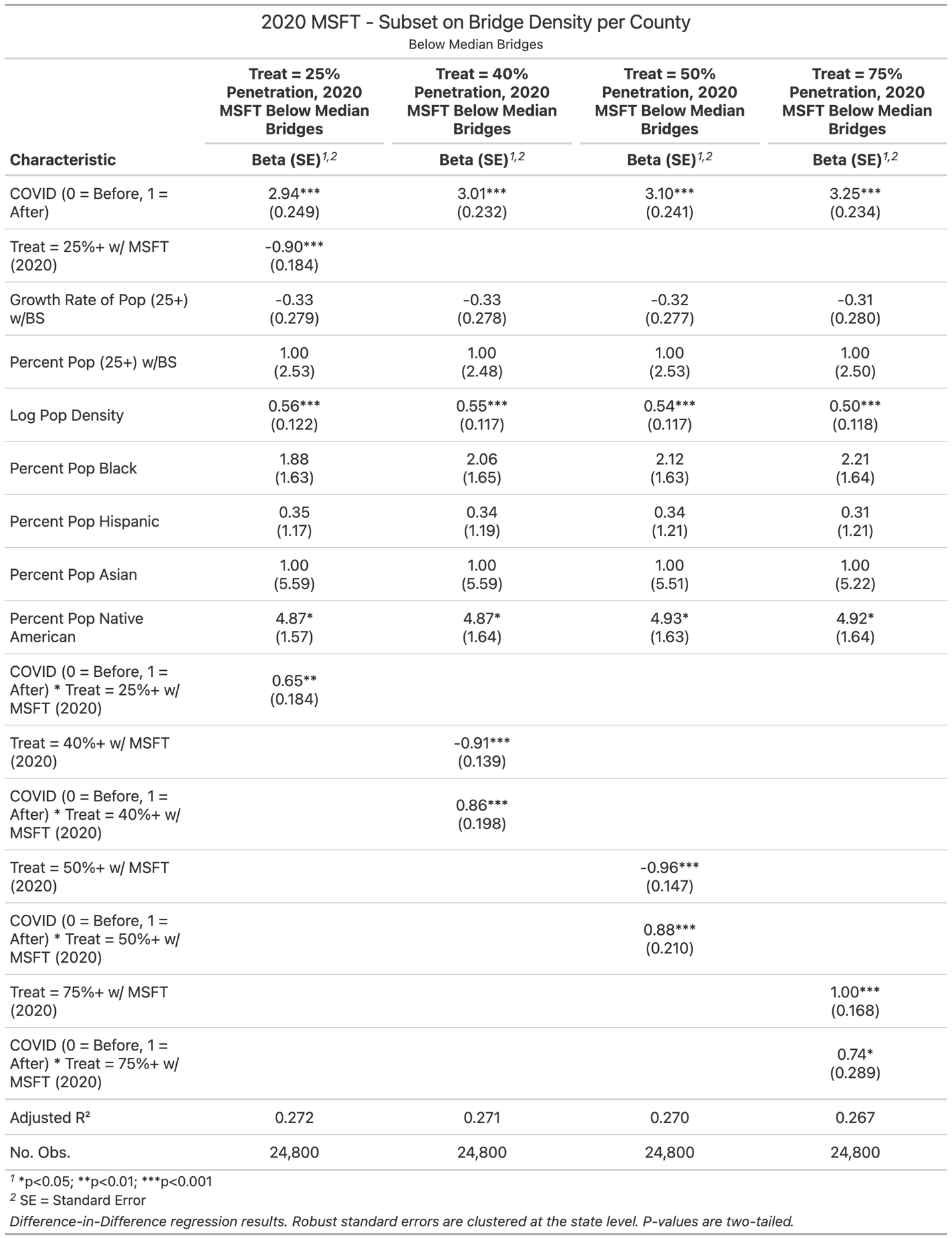
**

**Table I41: MSFT 2020 Buildings and Bridges**

**
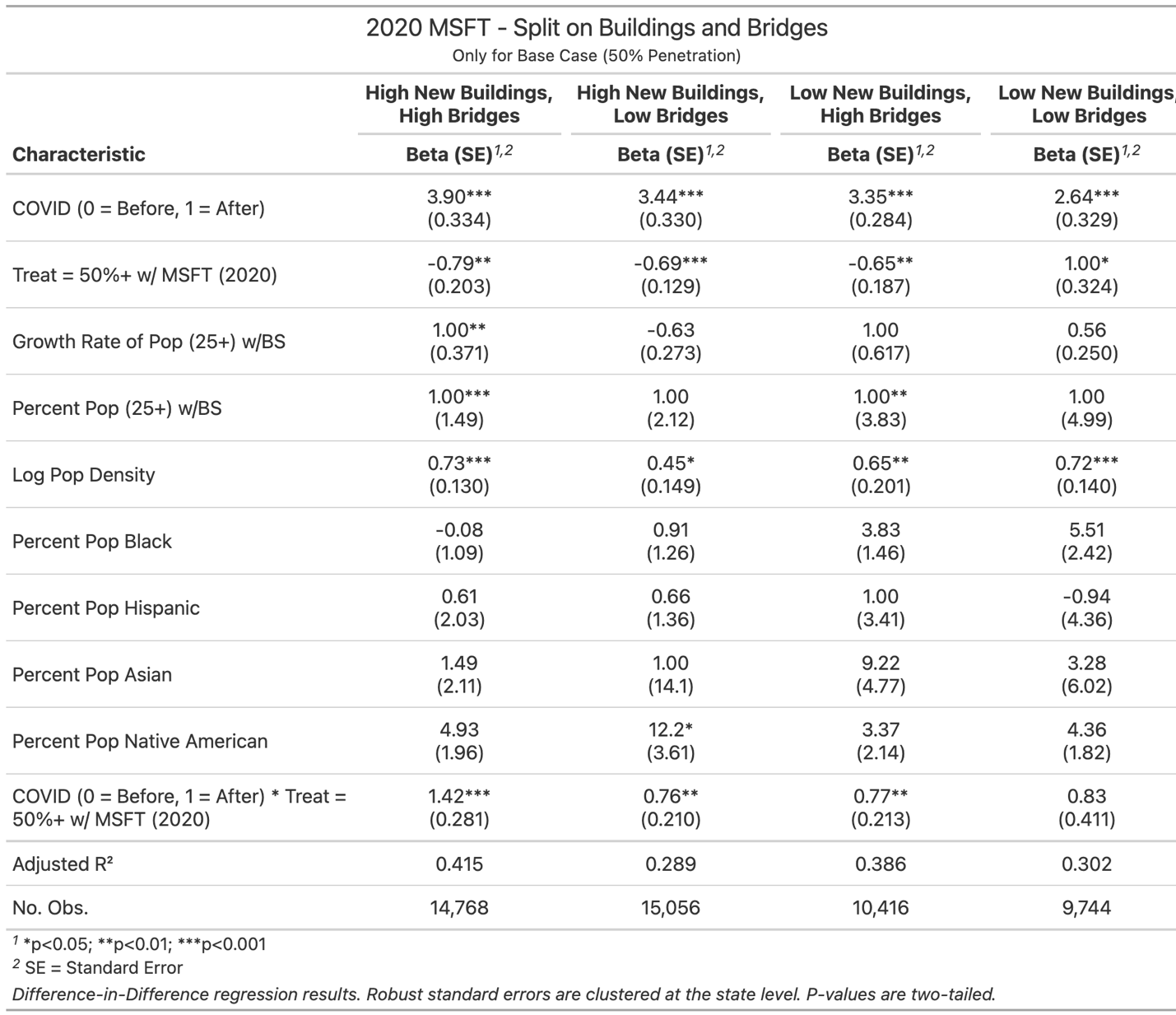
**

**Table I42: MSFT 2019 and MSFT 2020 No Change in Treatment vs Control**

**
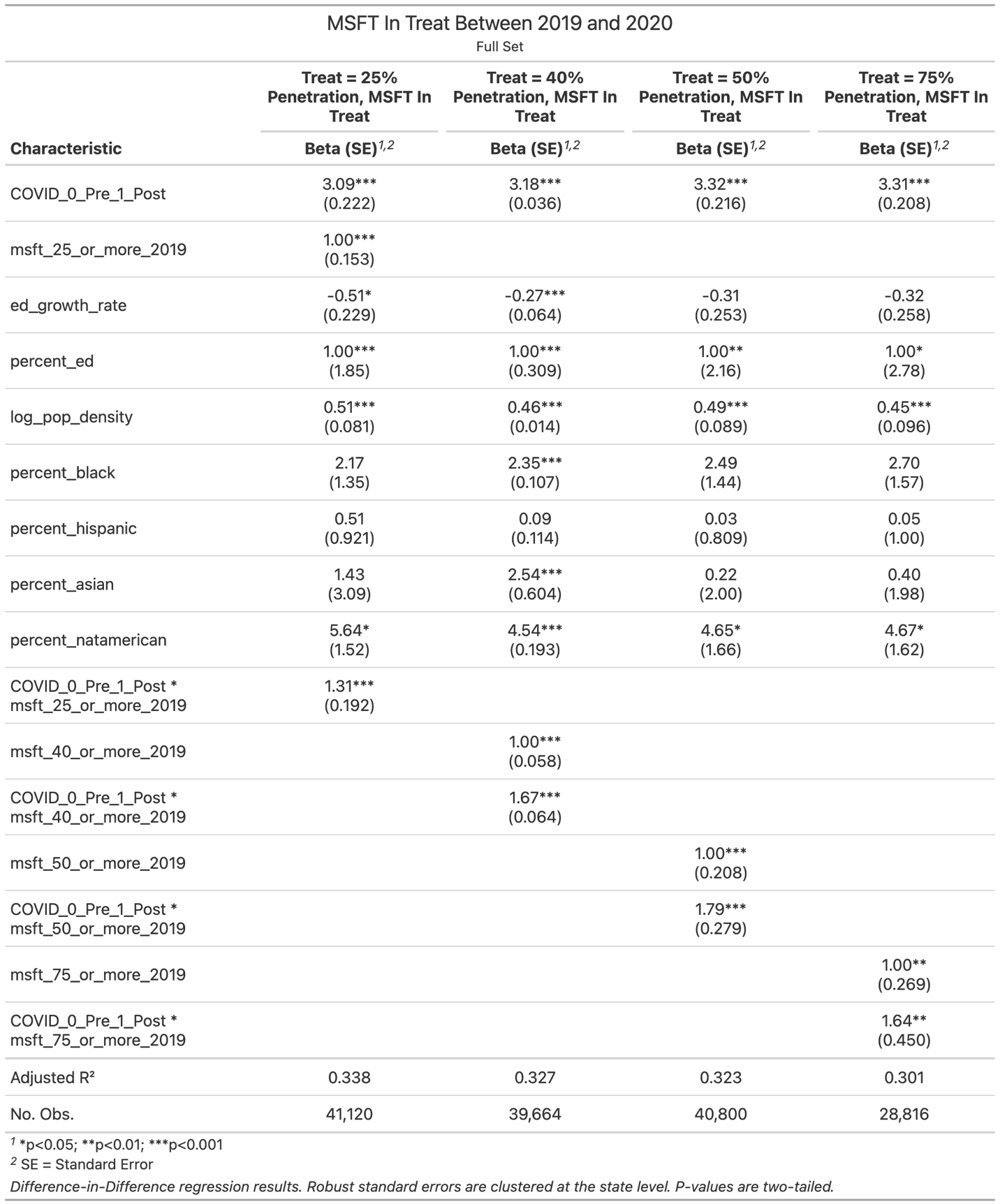
**

**Table I43: MSFT 2019 COVID in April**

**
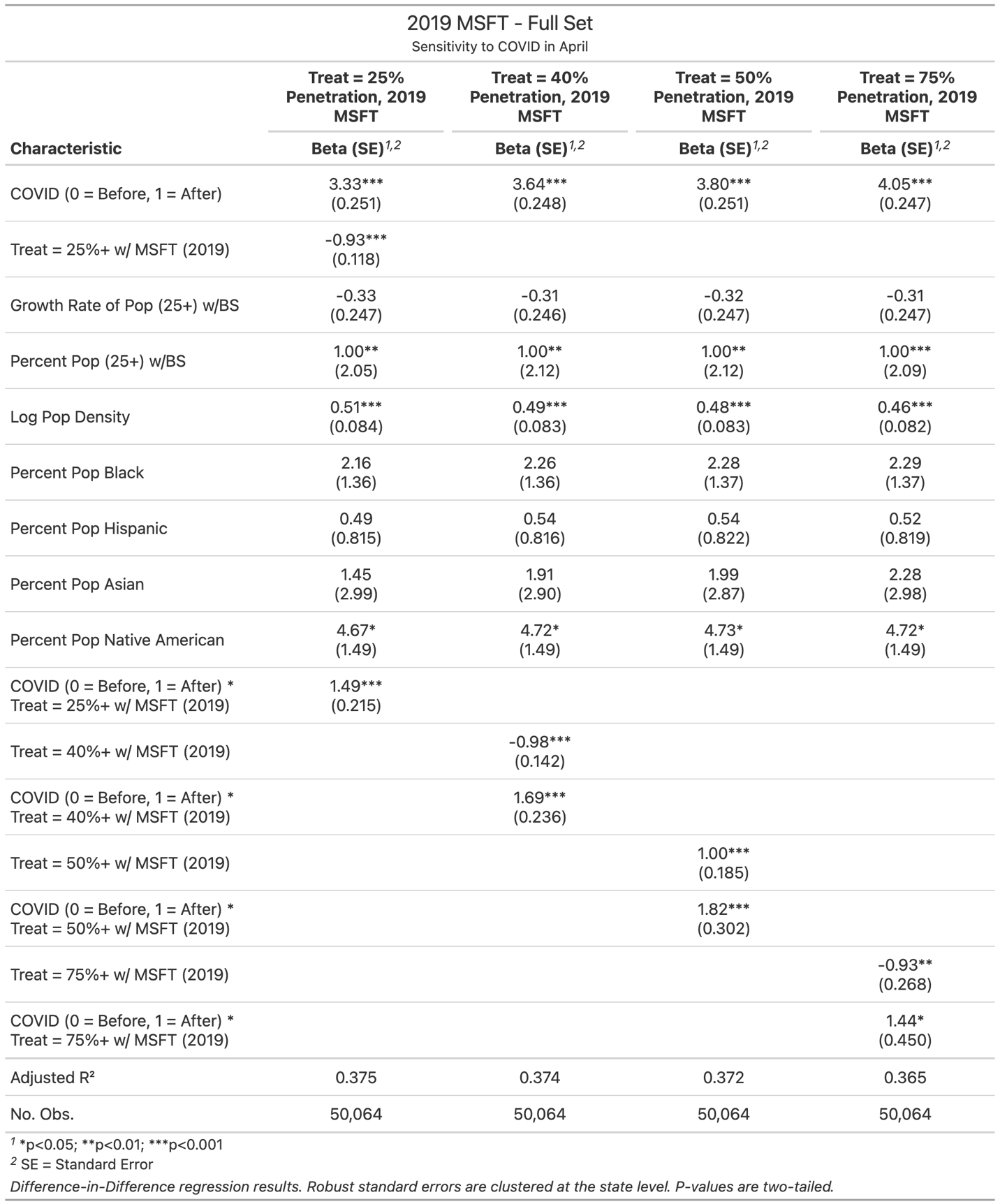
**

**Table I44: MSFT 2020 COVID in April**

**
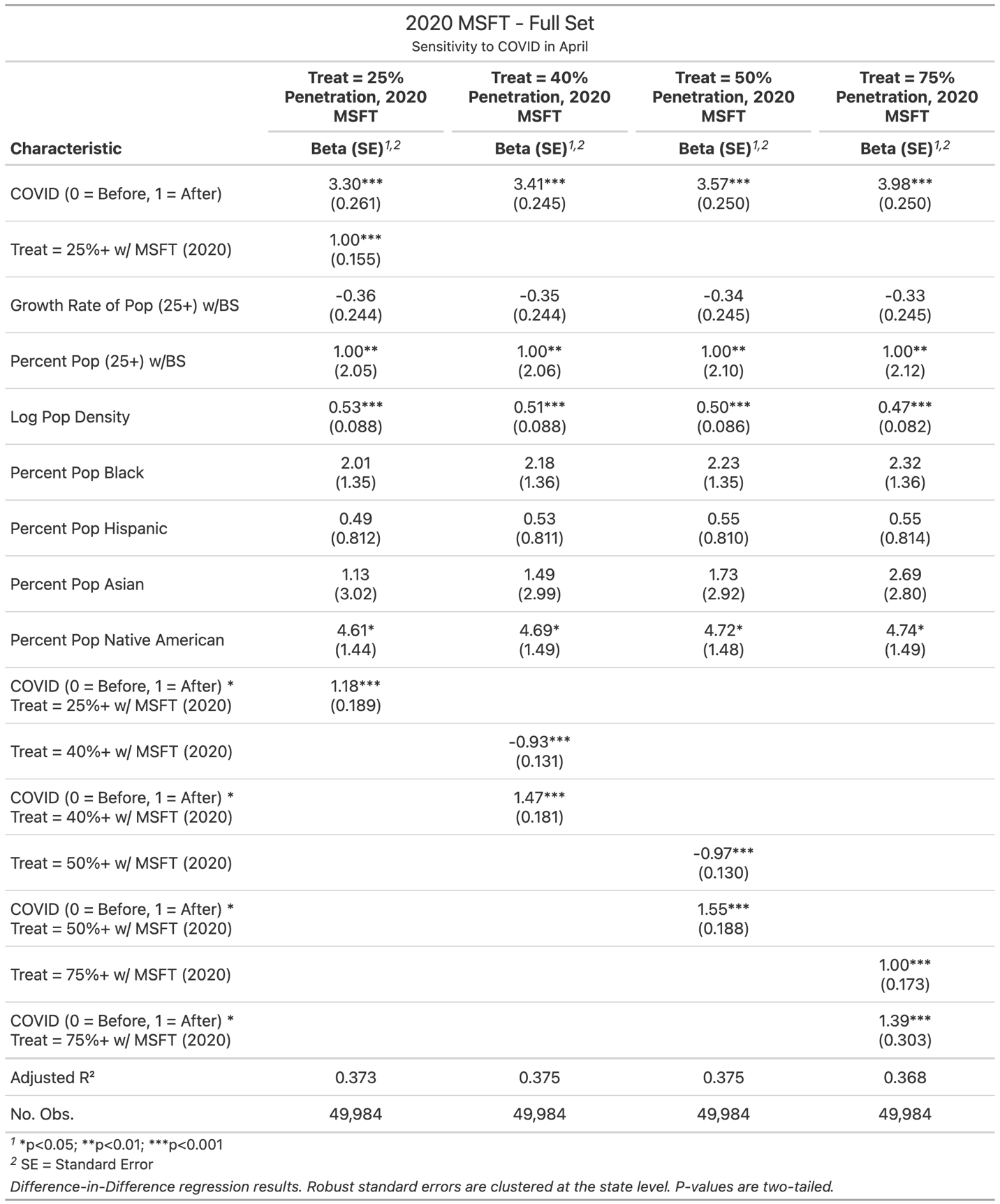
**

**Table I45: Public Libraries Base Case**

**
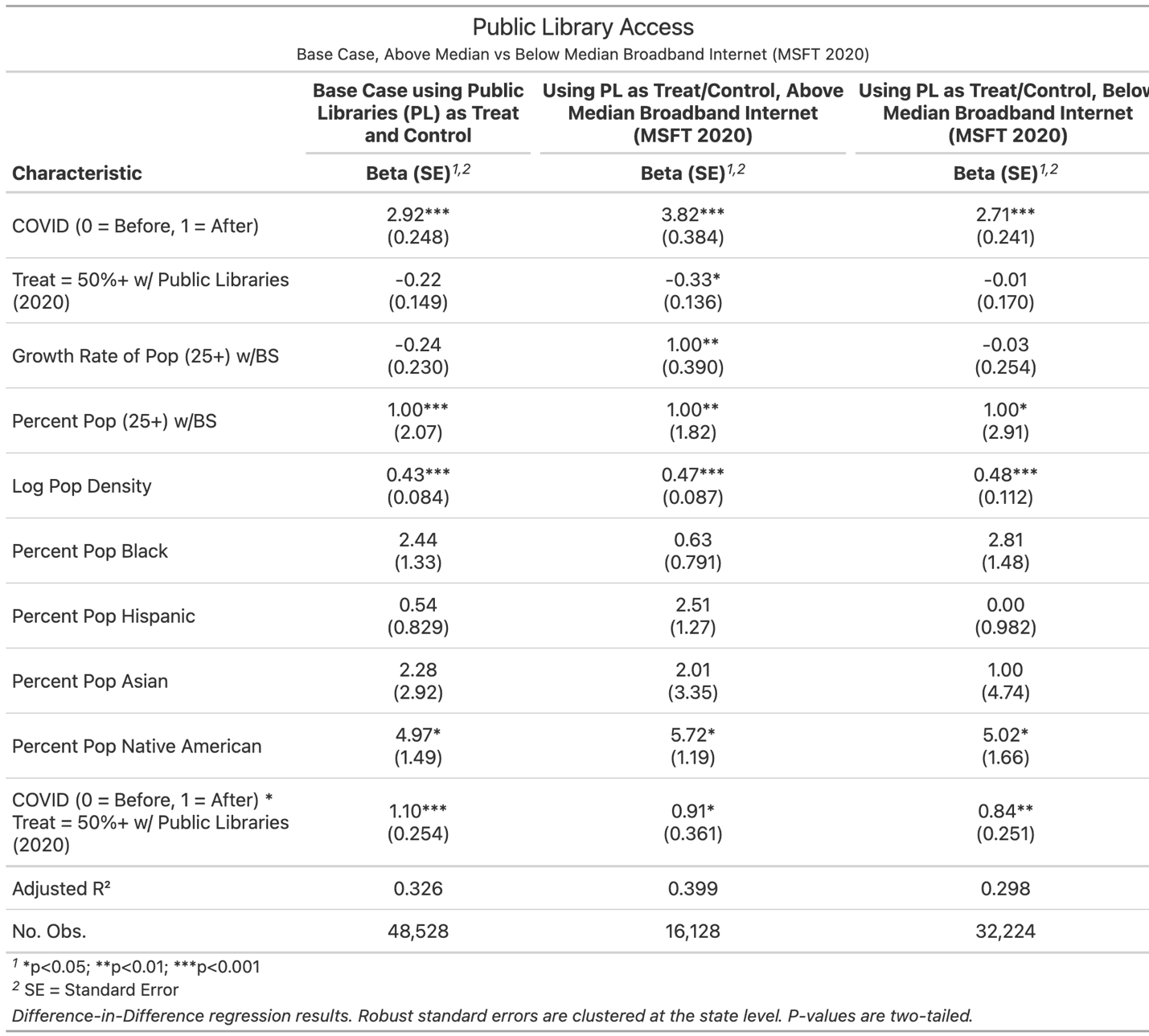
**

**Table I46: Public Libraries Above vs Below Black Population**

**
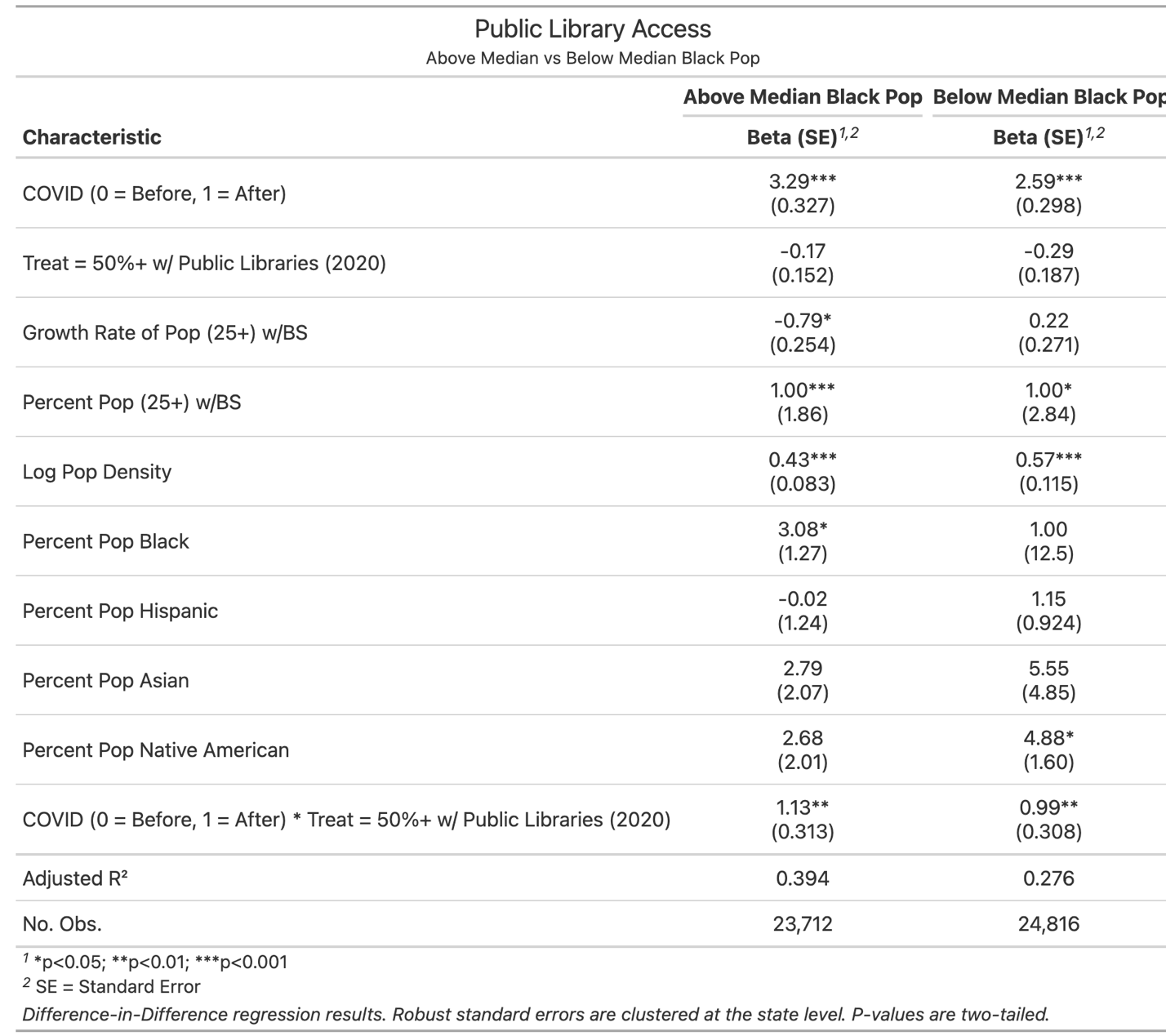
**

**Table I47: Public Libraries Above vs Below Hispanic Population**

**
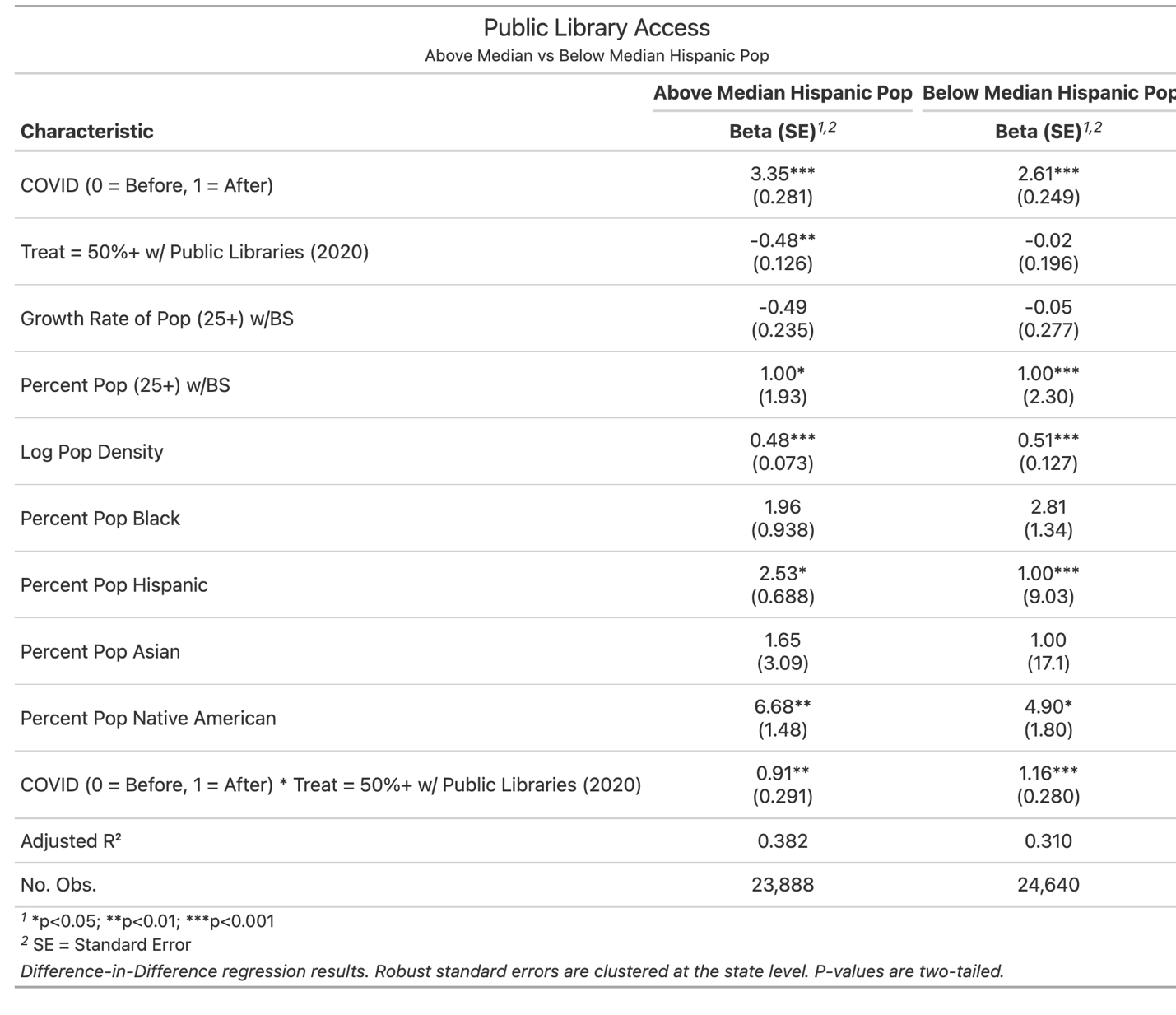
**

**Table I48: Public Libraries Above vs Below EID**

**
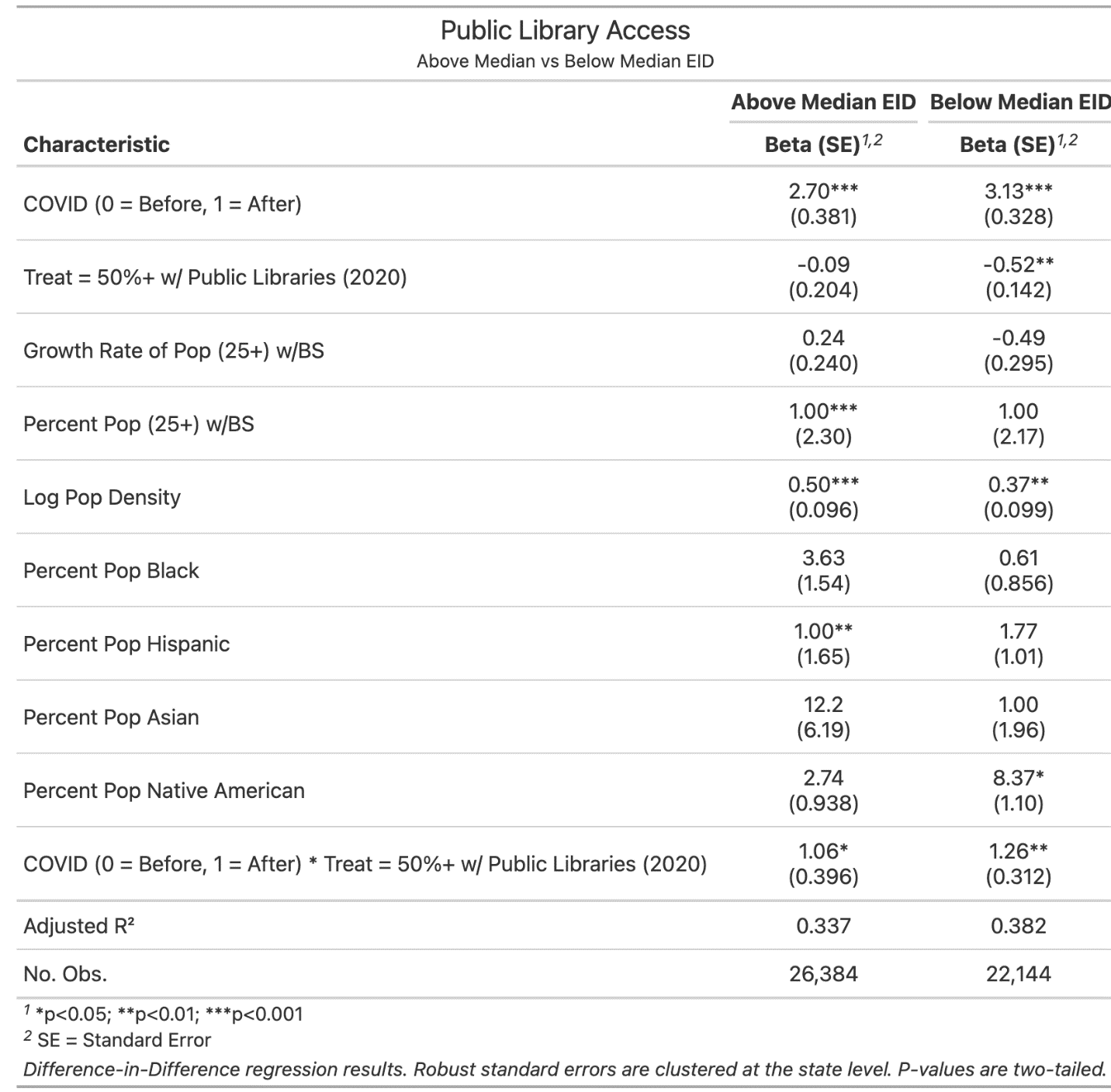
**

**Table I49: Public Libraries Above vs Below Income**

**
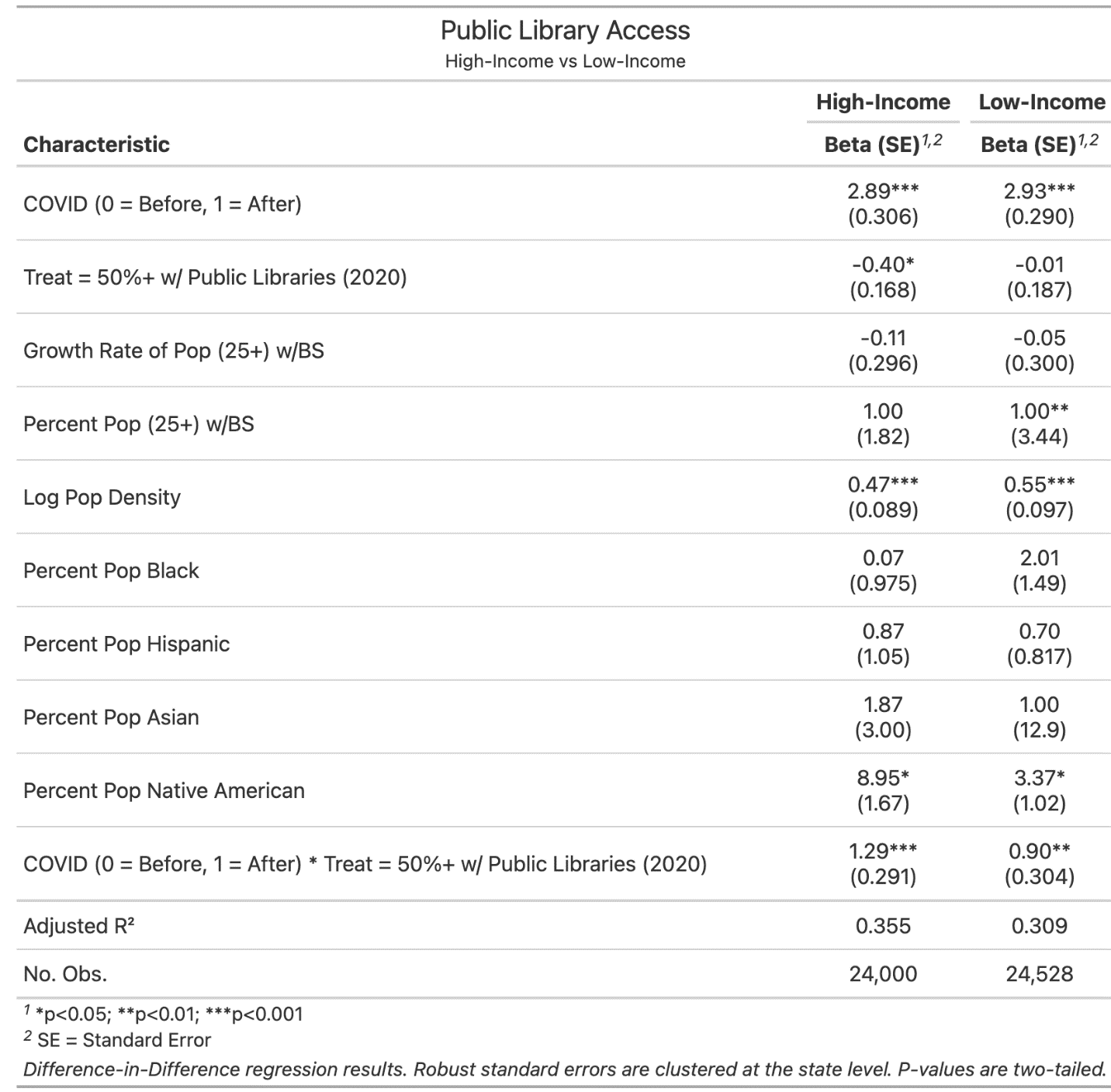
**

**Table I50: Public Libraries Urban vs Rural**

**
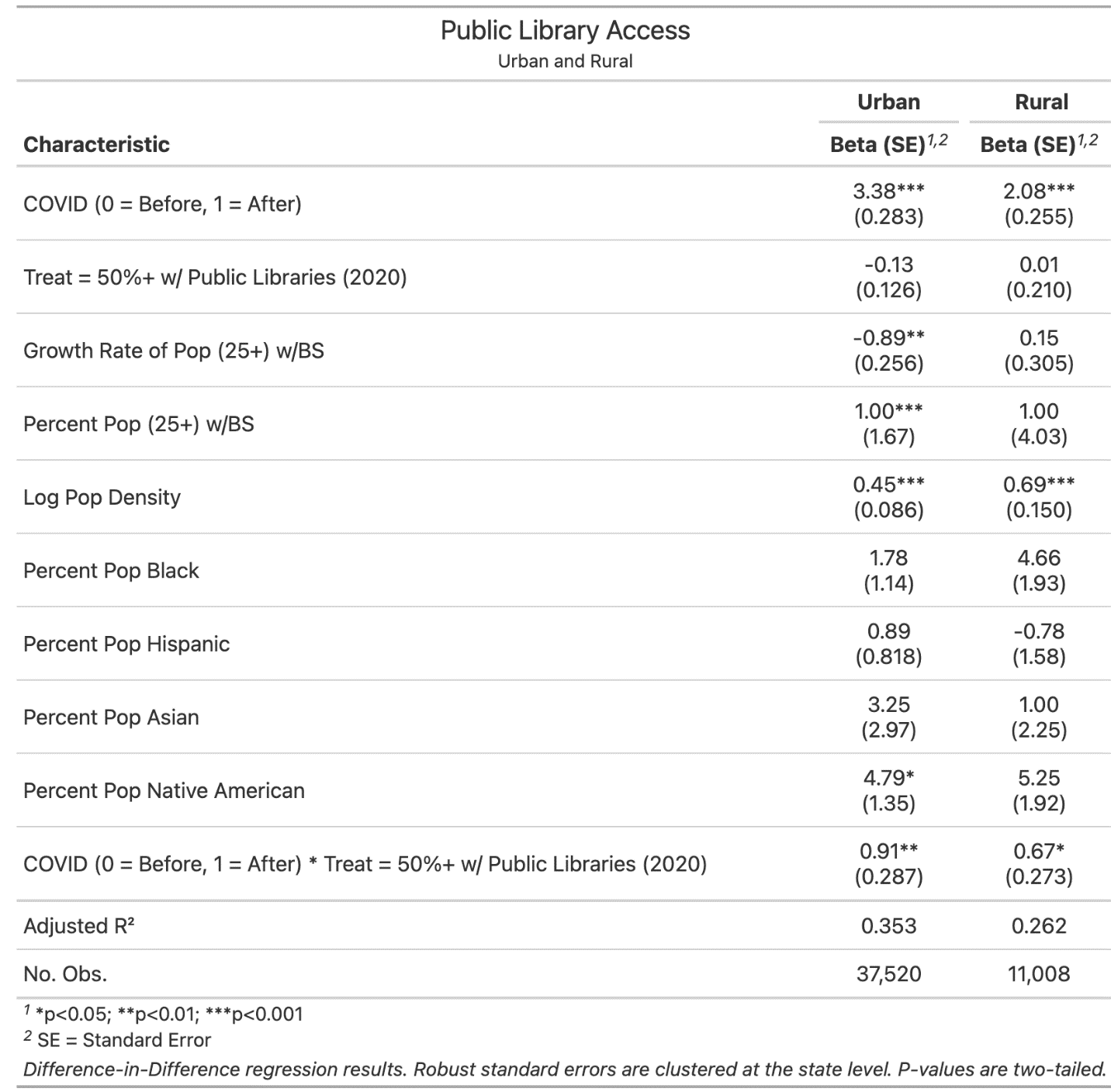
**

**Table I51: Public Libraries Above vs Below Median Employed in Tech**

**
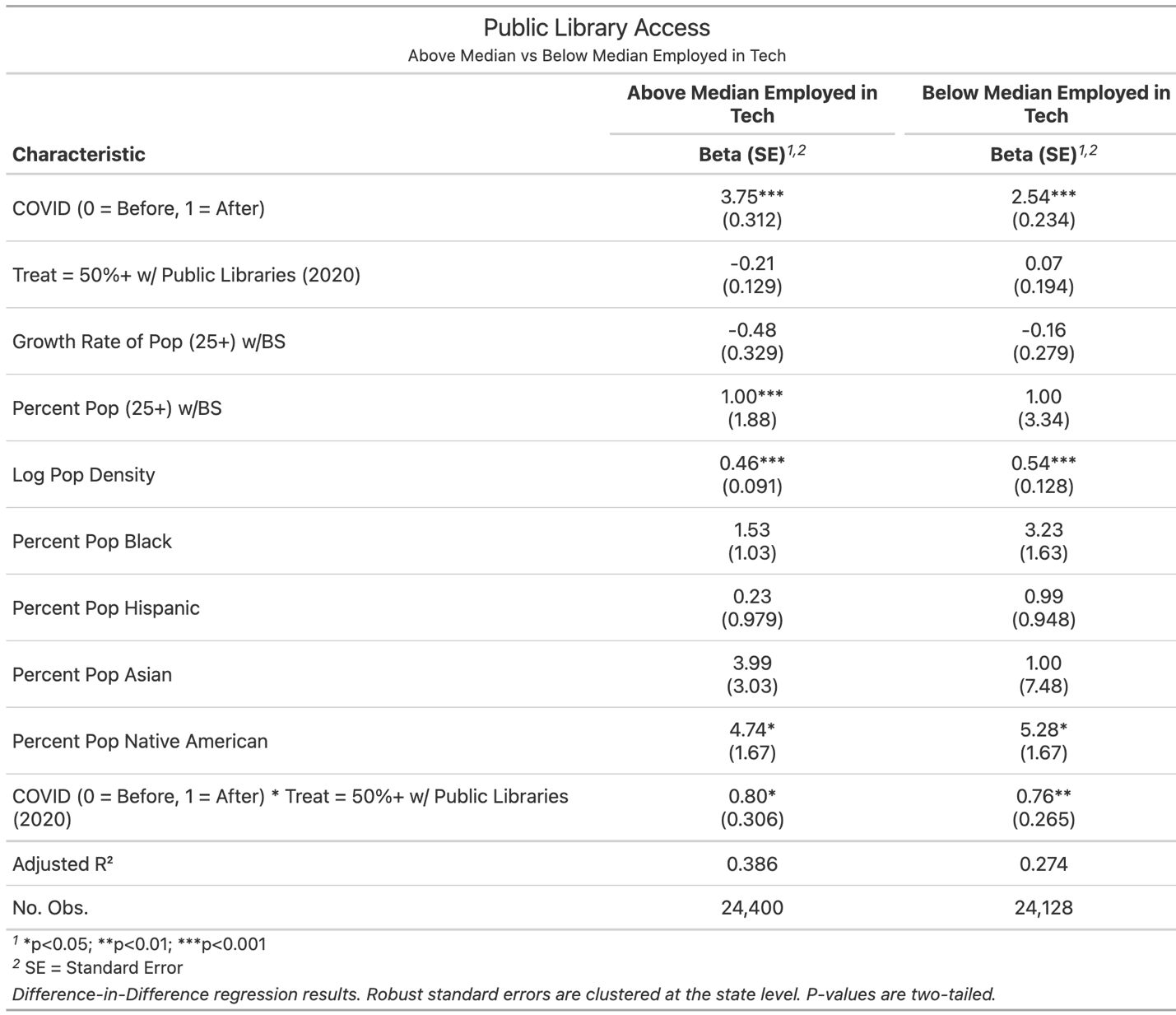
**

**Table I52: Public Libraries Above vs Below Median Employed in Service**

**
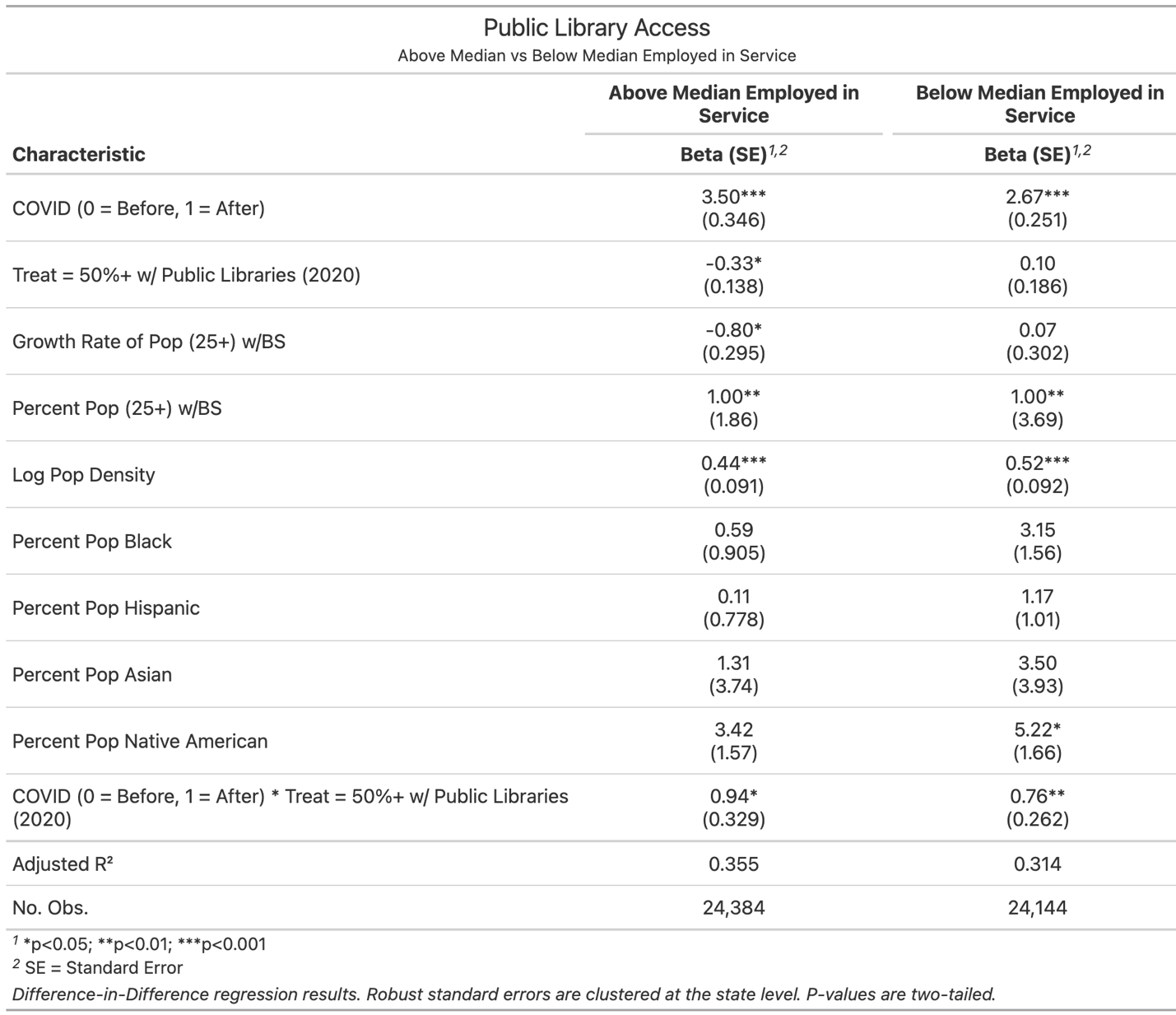
**

**Table I53: Public Libraries Above vs Below Median Employed in Industries which can Work From Home**

**
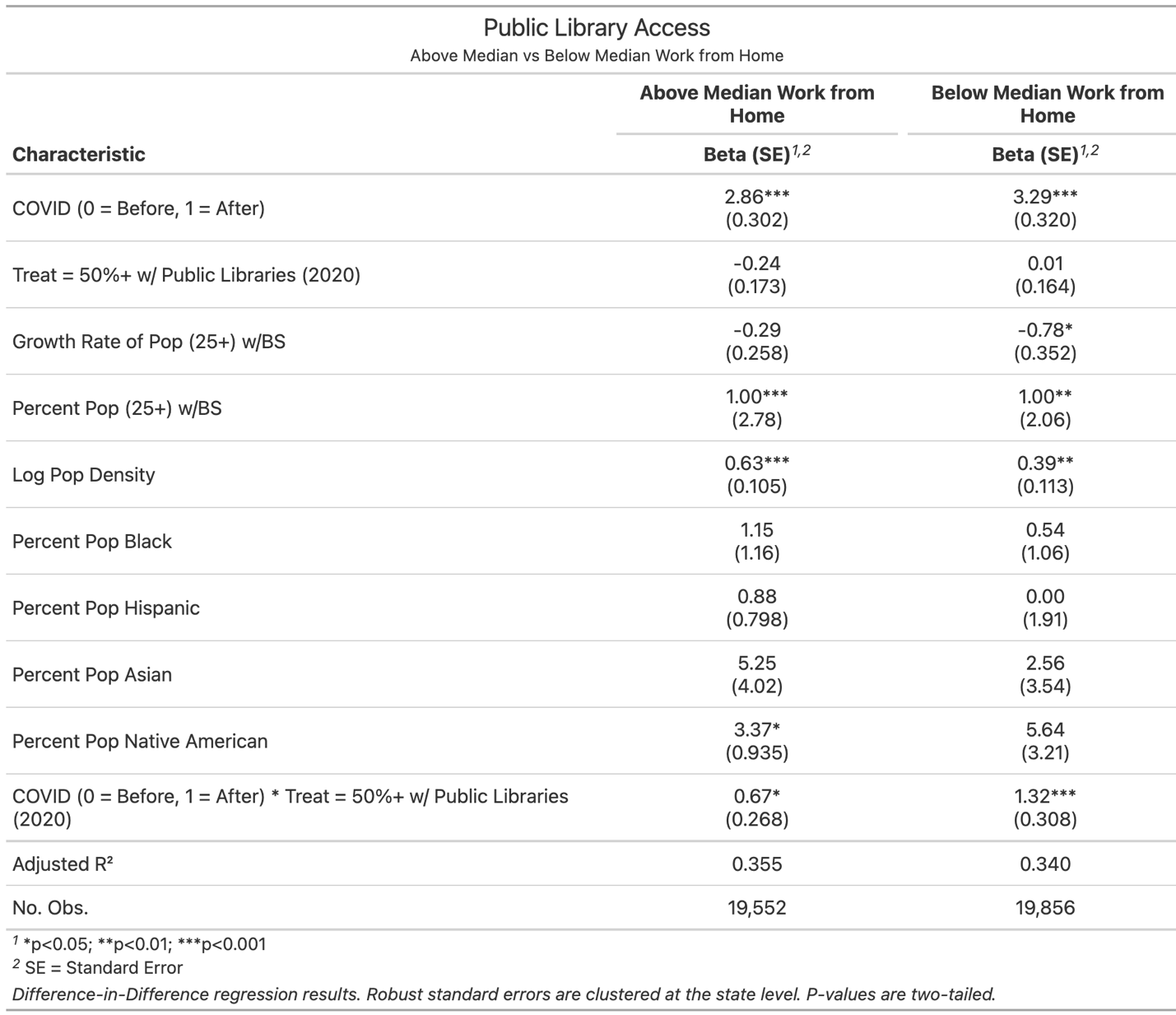
**

**Table I54: Public Libraries Above vs Below Median Single Parent Homes**

**
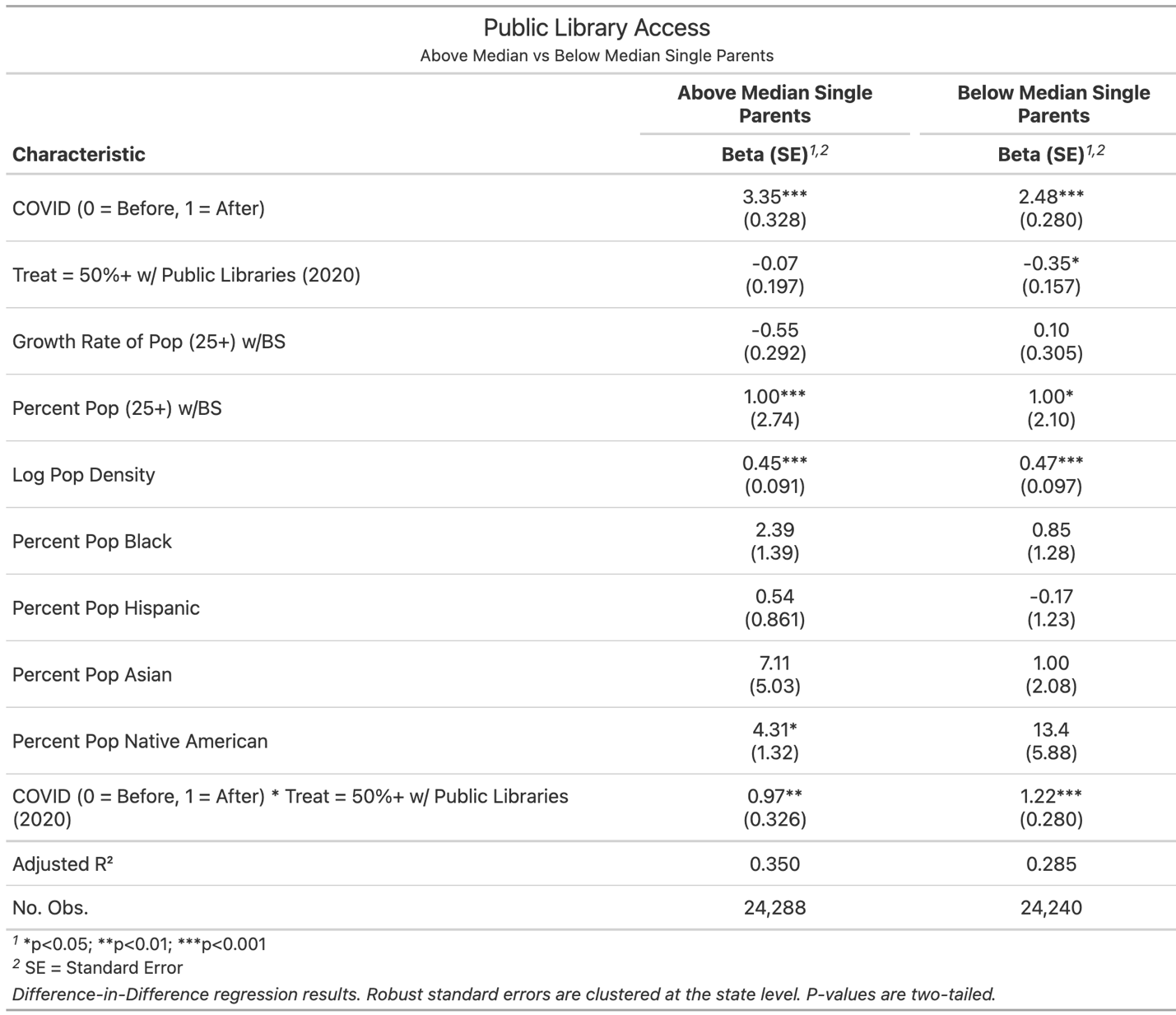
**

**Table I55: MSFT and COVID as Treat/Control Groups for High Accommodation Employment
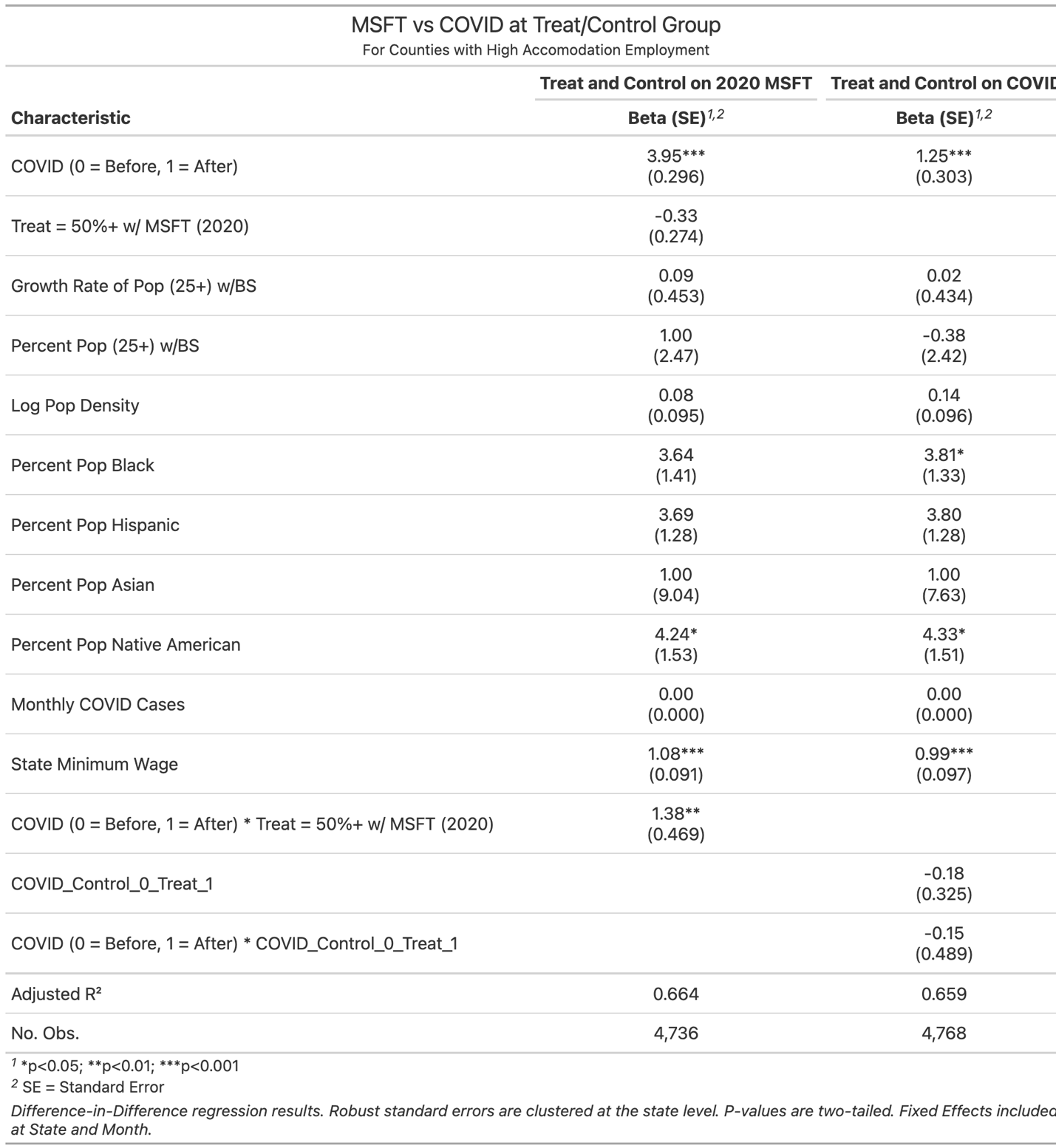
**

**Table I56: MSFT and COVID as Treat/Control Groups for High Admin Employment
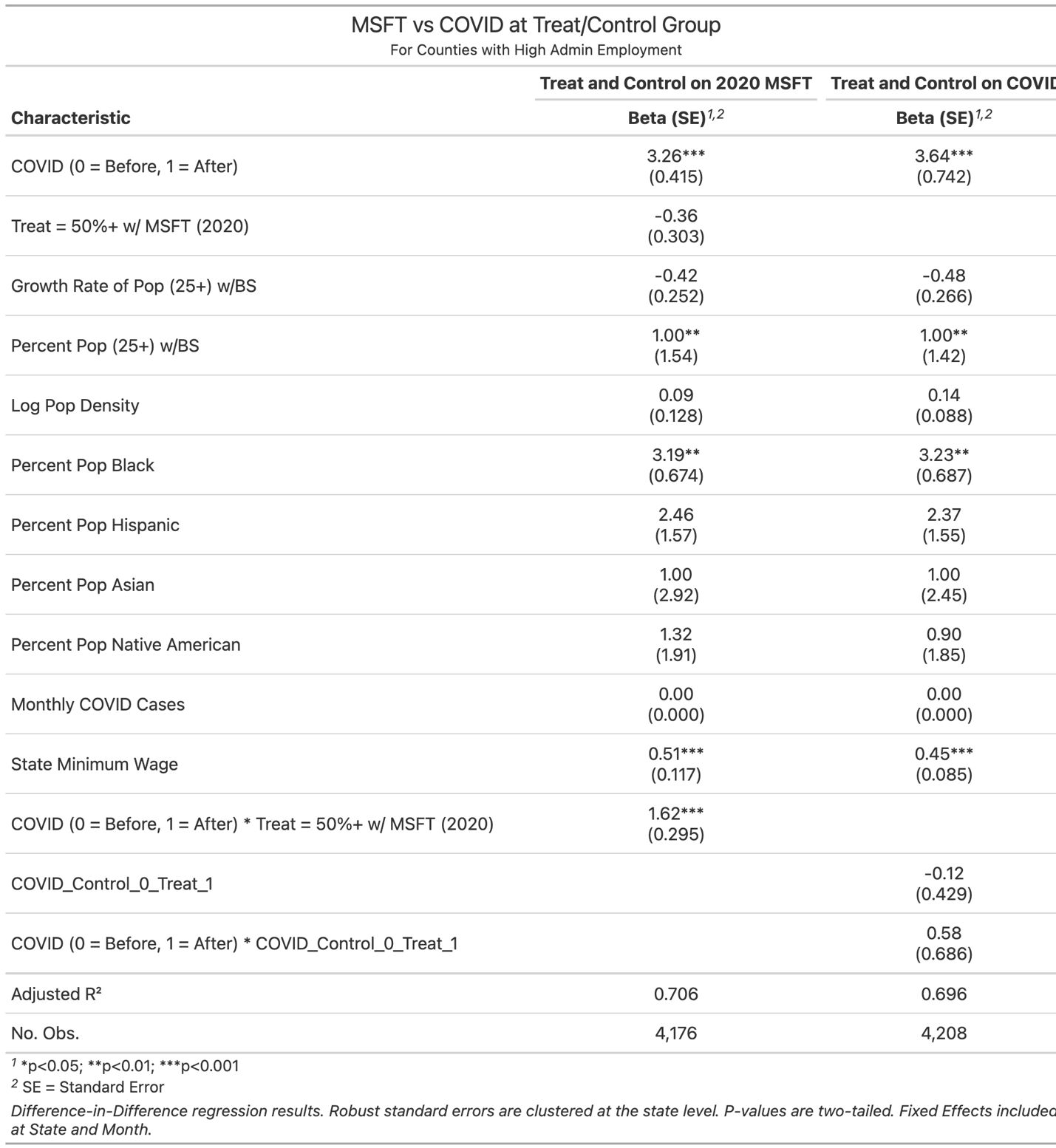
**

**Table I57: MSFT and COVID as Treat/Control Groups for High Agricultural Employment**

**
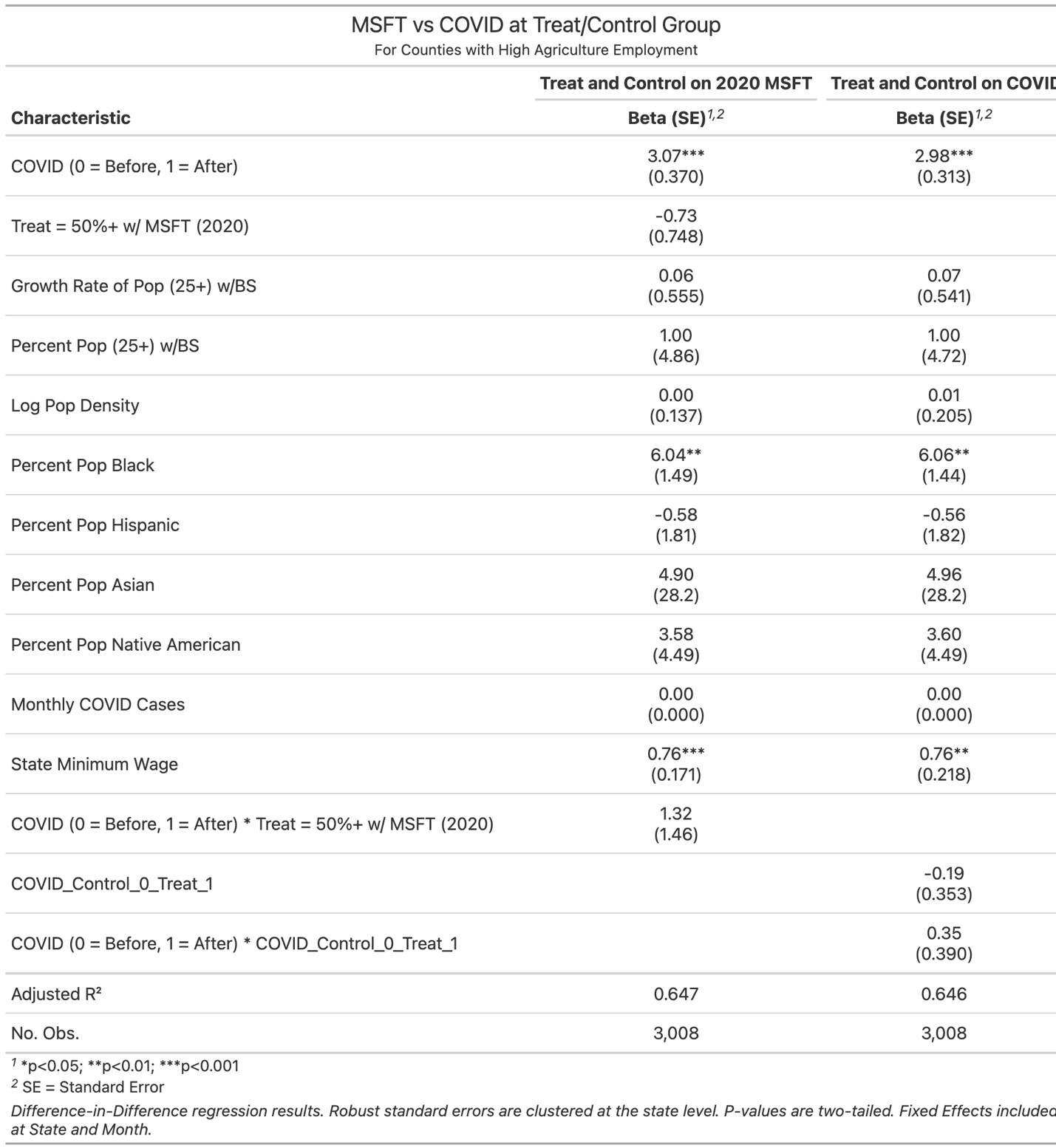
**

**Table I58: MSFT and COVID as Treat/Control Groups for High Arts Employment
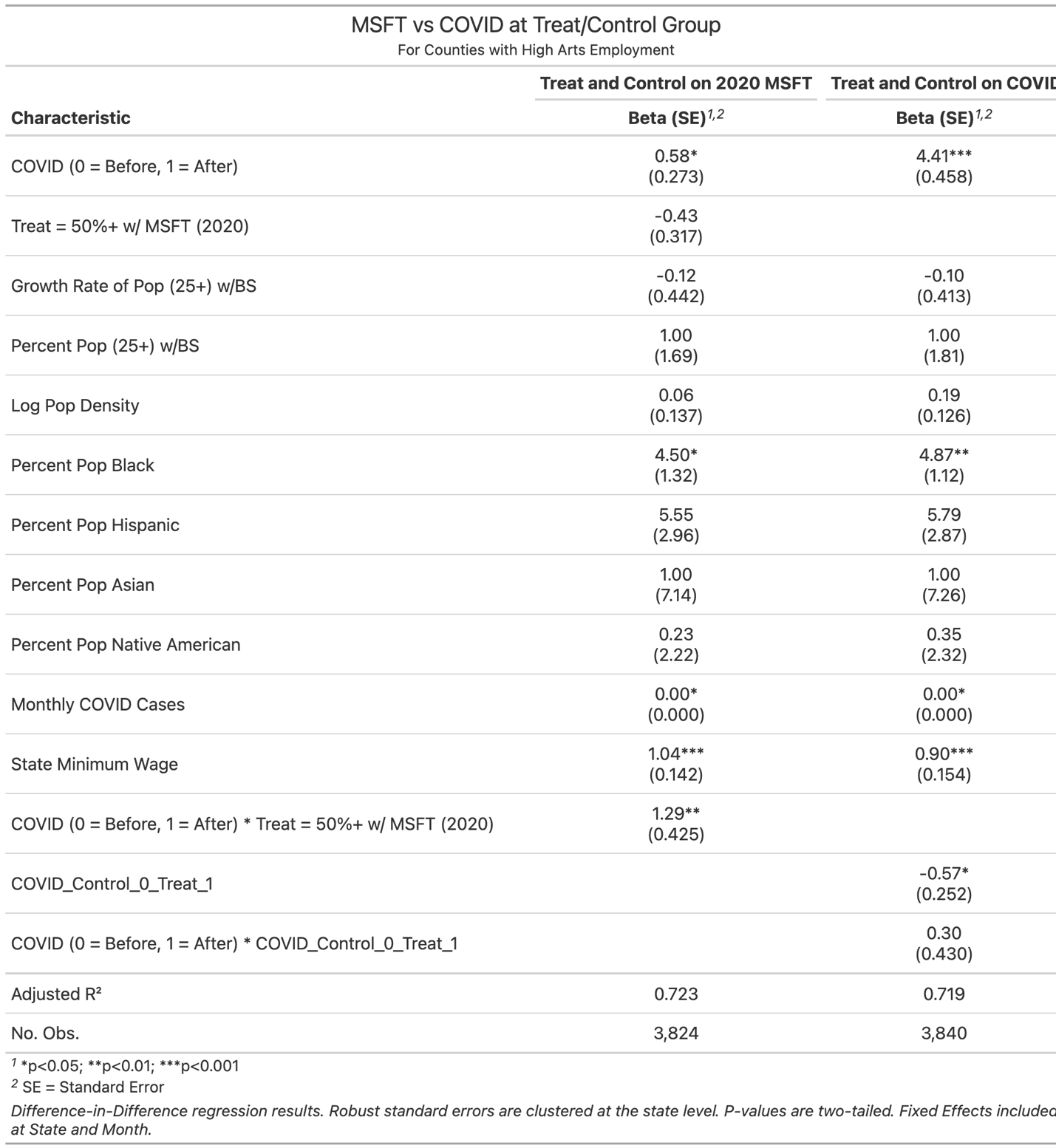
**

**Table I59: MSFT and COVID as Treat/Control Groups for High Construction Employment
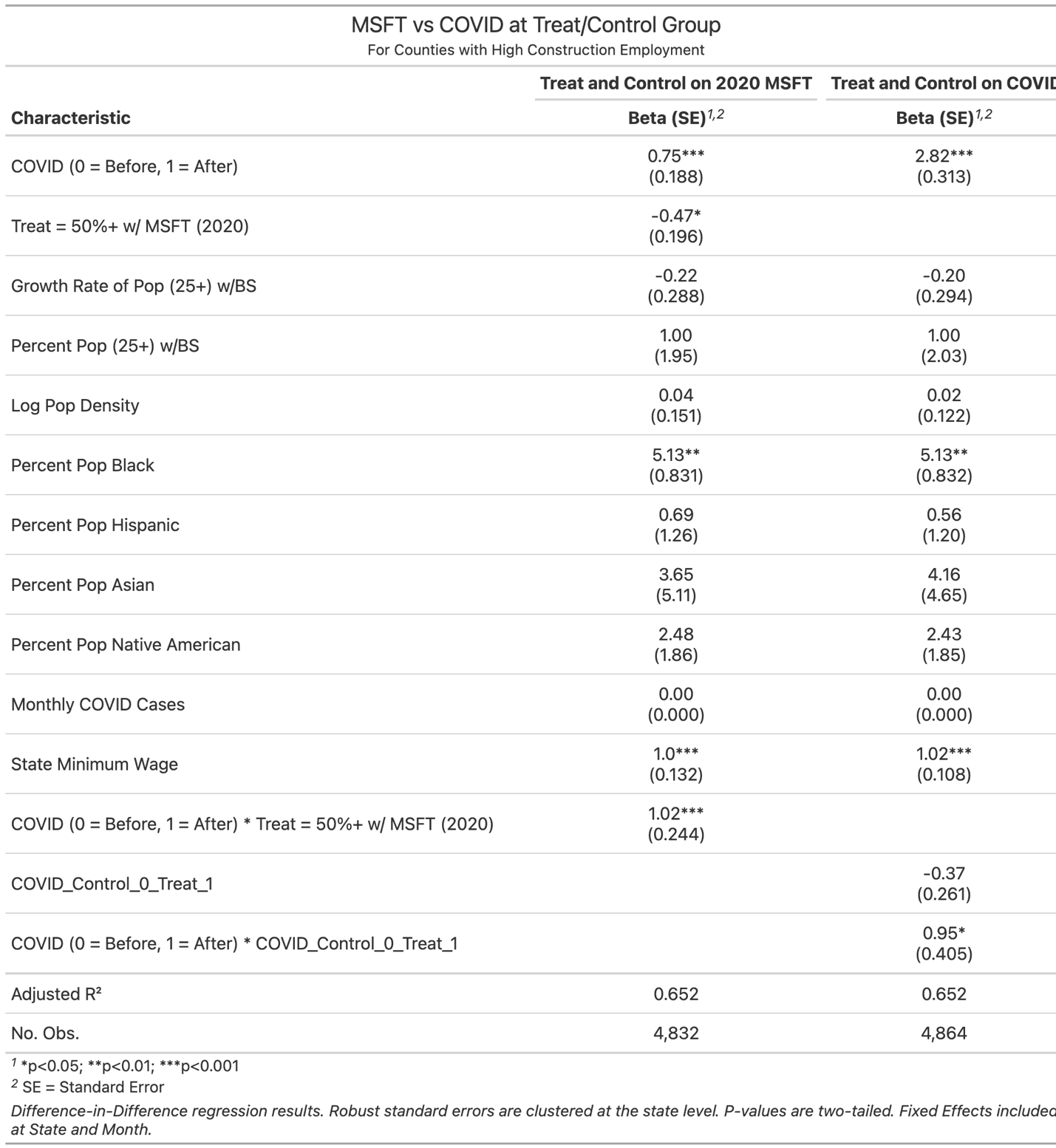
**

**Table I60: MSFT and COVID as Treat/Control Groups for High Education Employment
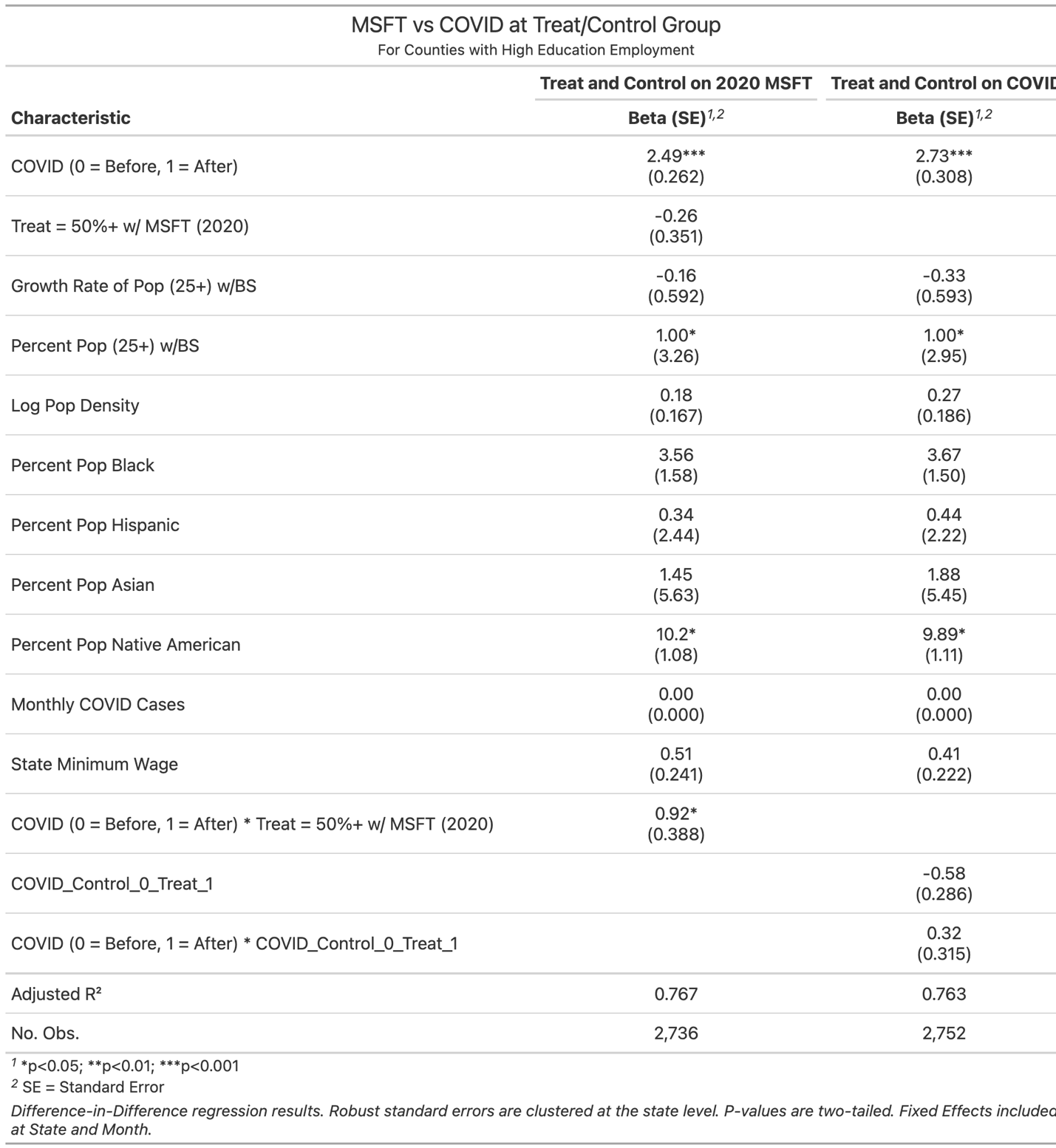
**

**Table I61: MSFT and COVID as Treat/Control Groups for High Finance Employment
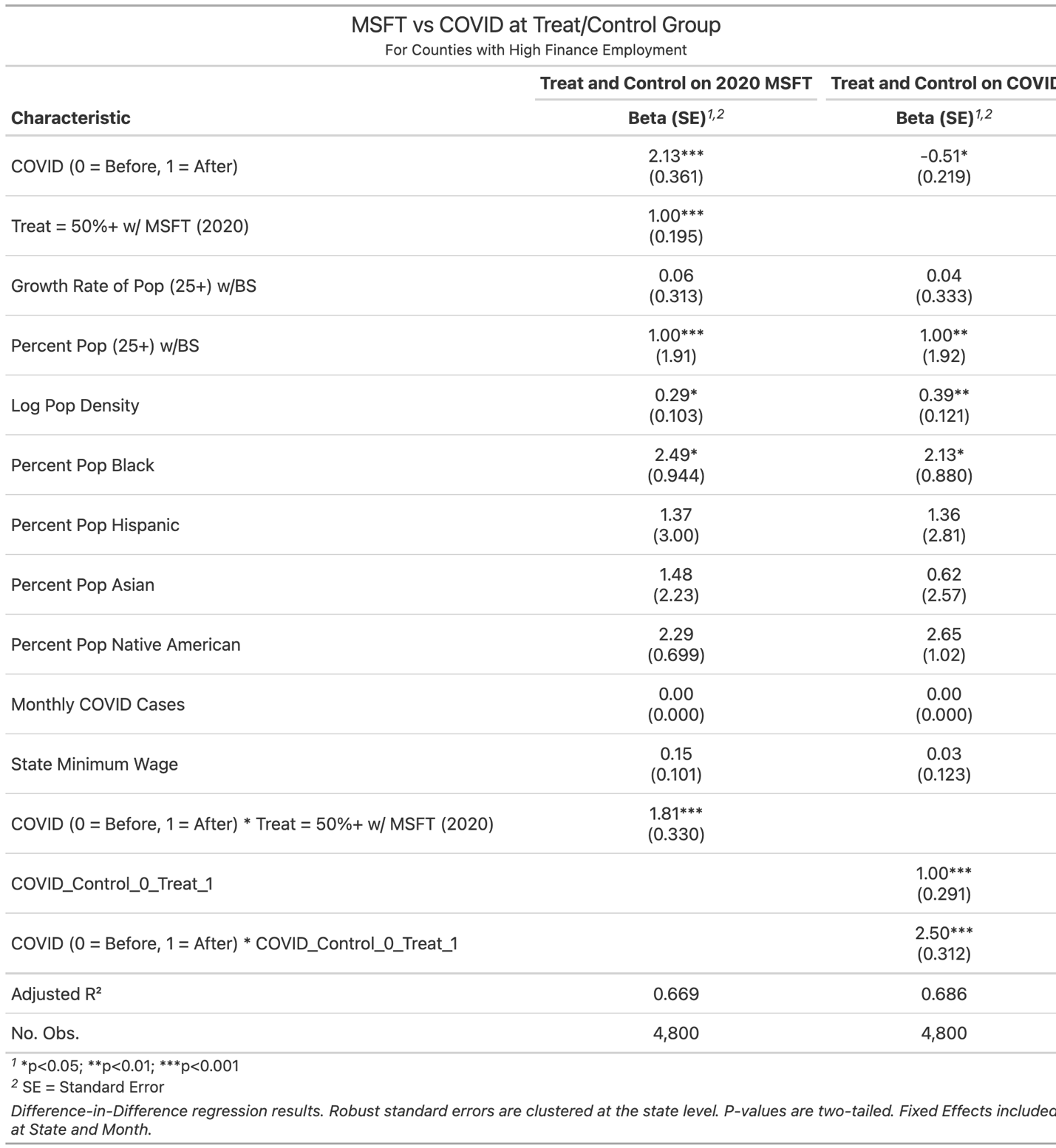
**

**Table I62: MSFT and COVID as Treat/Control Groups for High Health Employment
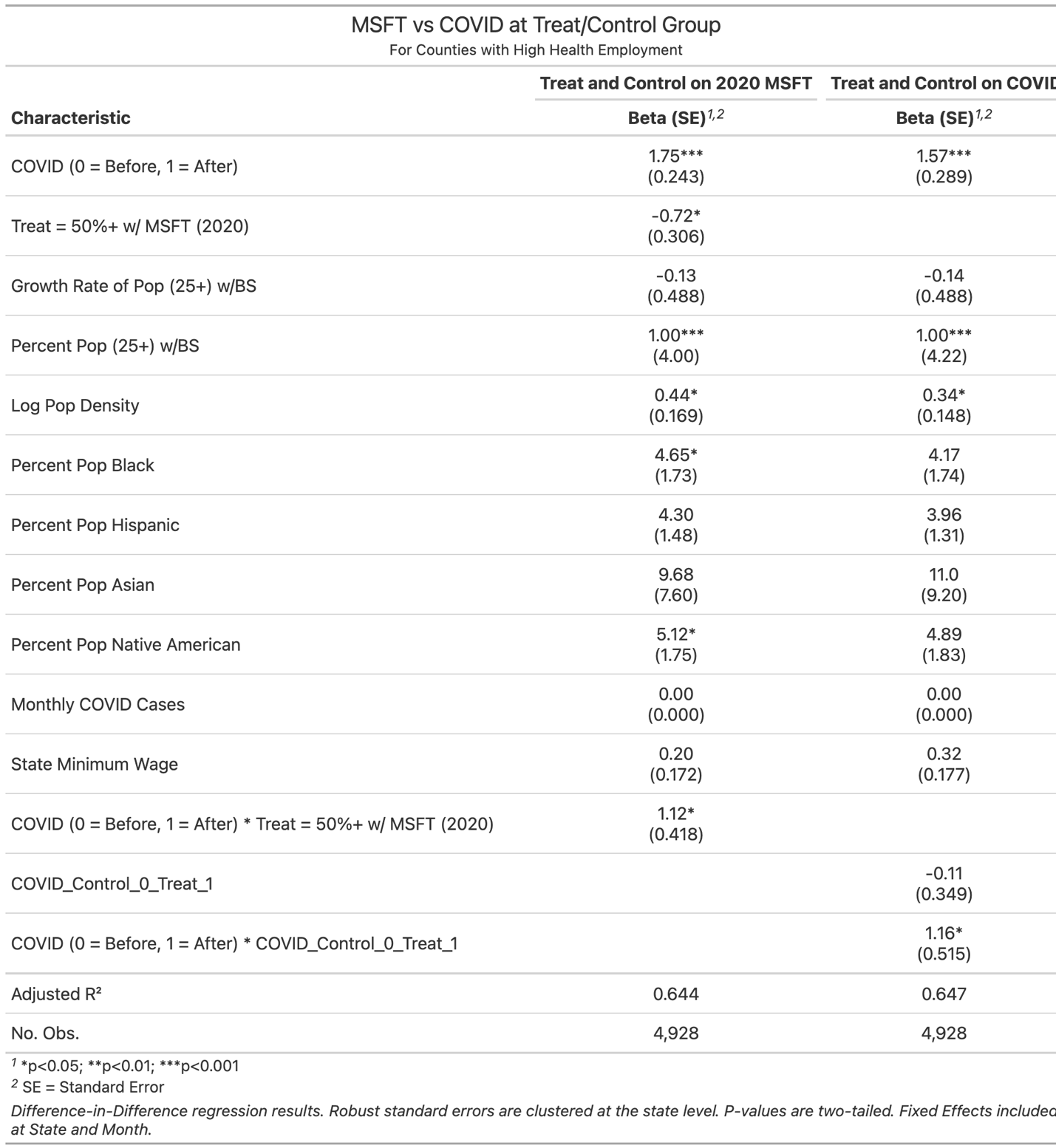
**

**Table I63: MSFT and COVID as Treat/Control Groups for High Information Employment
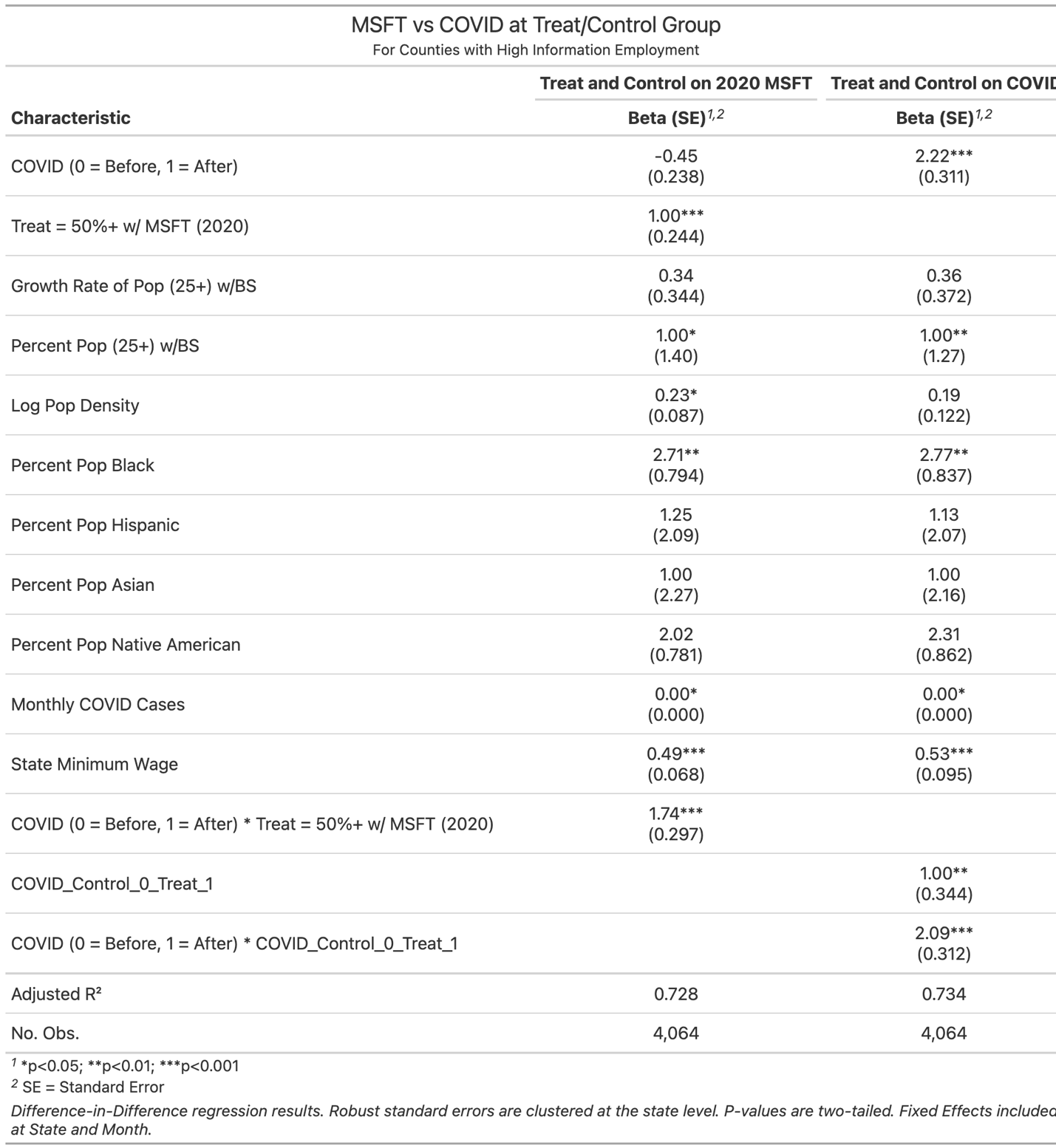
**

**Table I64: MSFT and COVID as Treat/Control Groups for High Manufacturing Employment
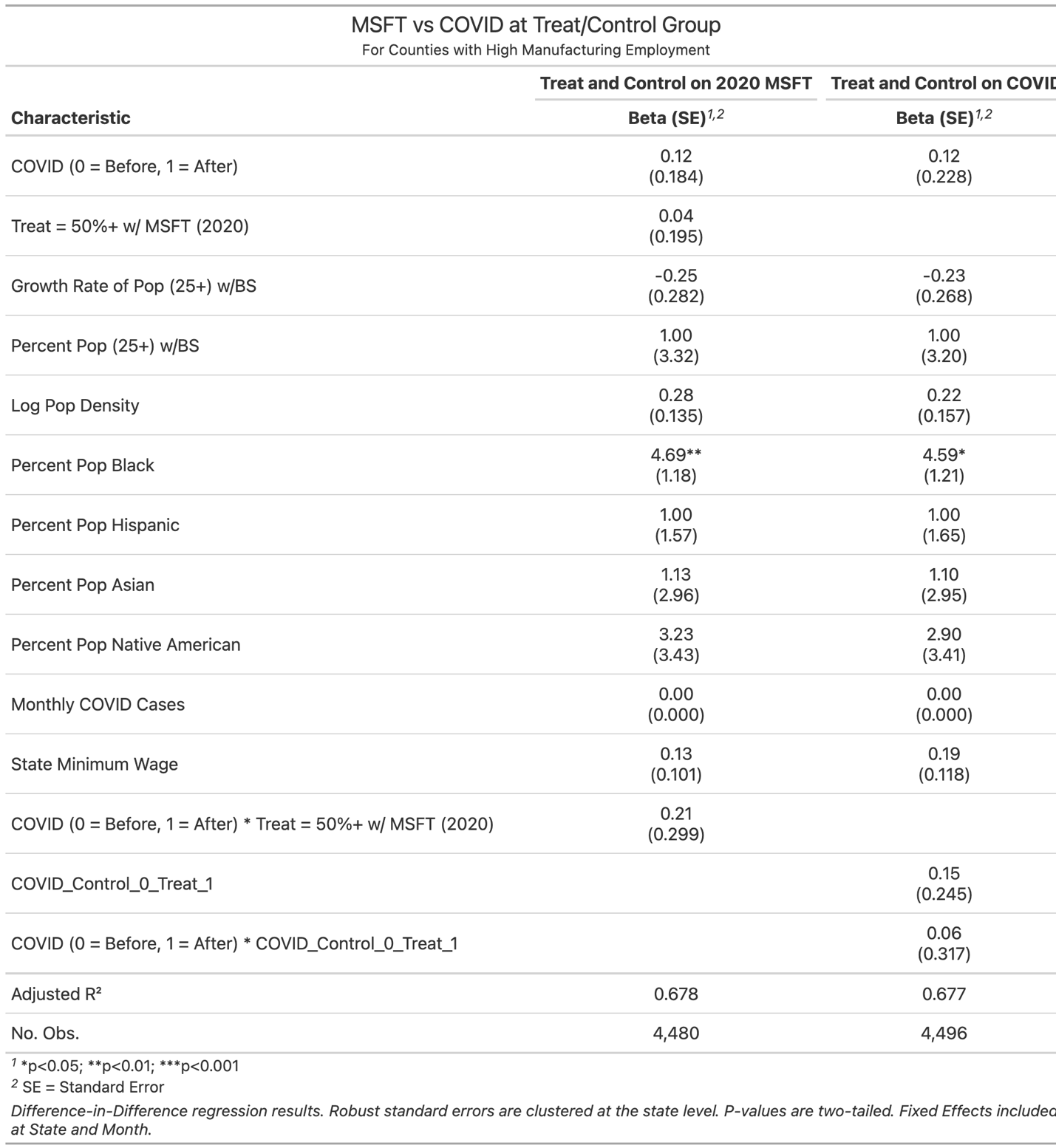
**

**Table I65: MSFT and COVID as Treat/Control Groups for High Mining Employment
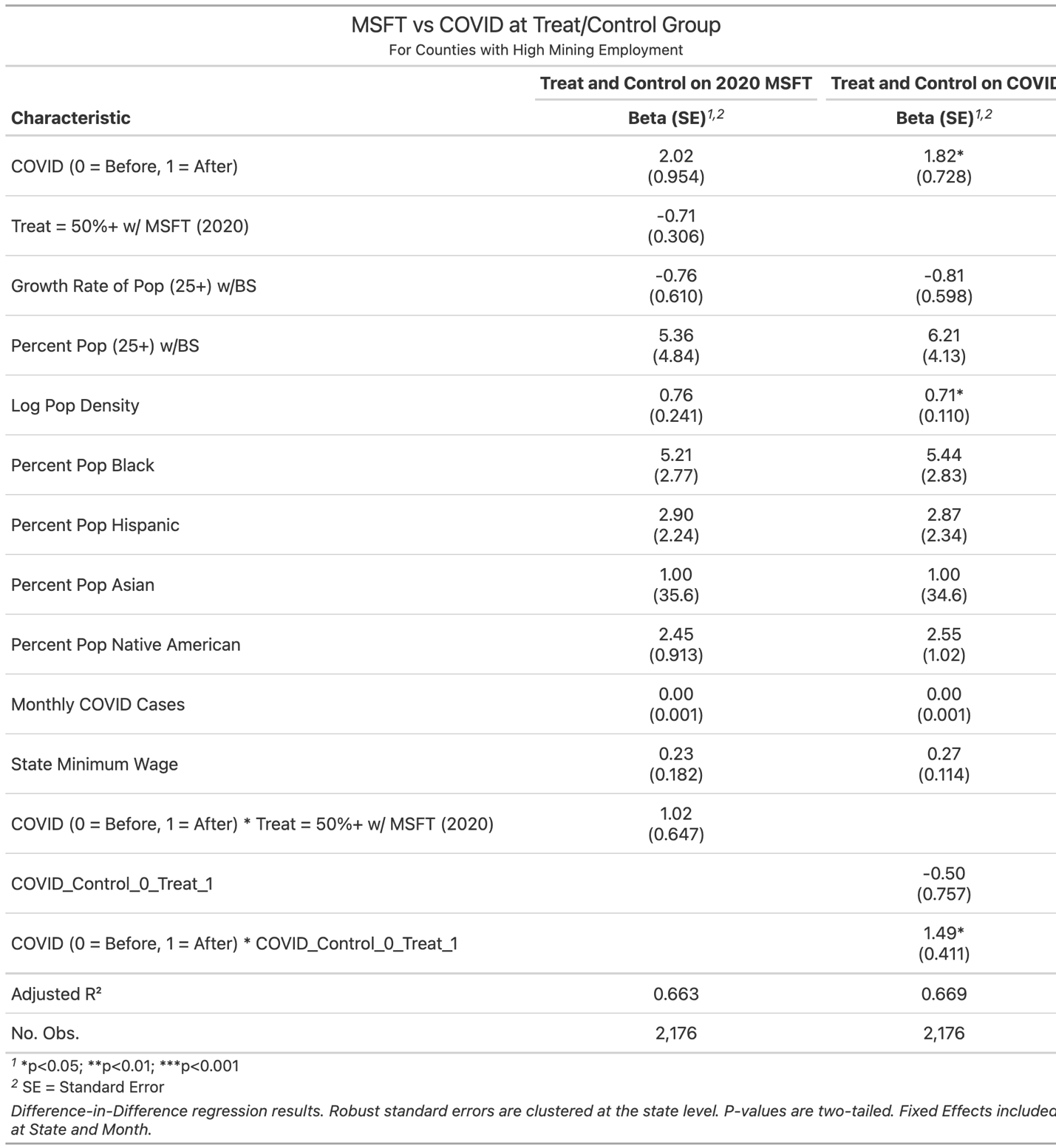
**

**Table I66: MSFT and COVID as Treat/Control Groups for High Other Employment**

**Table I67: MSFT and COVID as Treat/Control Groups for High Real Estate Employment**

**Table I68: MSFT and COVID as Treat/Control Groups for High Retail Trade Employment**

**Table I69: MSFT and COVID as Treat/Control Groups for High Tech Employment**

**Table I70: MSFT and COVID as Treat/Control Groups for High Transport Employment**

**Table I71: MSFT and COVID as Treat/Control Groups for High Utilities Employment**

**Table I72: MSFT and COVID as Treat/Control Groups for High Wholesale Trade Employment**

# Appendix J: Appendix References

1. Gillett, S., Lehr, W., Osorio, C. & Sirbu, M. *Measuring Broadbandʼs Economic Impact*. http://cfp.mit.edu/publications/CFP_Papers/Measuring_bb_econ_impact-final.pdf (2006).

2. Crandall, R., Lehr, W. & Litan, R. The Effects of Broadband Deployment on Output and Employment: A Cross-sectional Analysis of U.S. Data. *Issues in Economic Policy* **6**, (2007).

3. Shideler, D. The Economic Impact of Broadband Deployment in Kentucky. **3**, 31 (2007).

4. Lehr, W. H., Osorio, C. A., Gillett, S. E. & Sirbu, M. A. Measuring Broadband’s Economic Impact. 37 (2006).

5. Krishna Jayakar & Eun-A Park. Broadband Availability and Employment: An Analysis of County-Level Data from the National Broadband Map. *Journal of Information Policy* **3**, 181 (2013).

6. Kandilov, I. T. & Renkow, M. Infrastructure Investment and Rural Economic Development: An Evaluation of USDA’s Broadband Loan Program. *Growth and Change* **41**, 165–191 (2010).

7. Stenberg, P. *et al.* *Broadband Internet’s Value for Rural America*. 70 (2009).

8. Whitacre, B., Gallardo, R. & Strover, S. Broadband׳s contribution to economic growth in rural areas_ Moving towards a causal relationship | Elsevier Enhanced Reader. *TelecommunicationsPolicy* **38**, 1011–1023 (2014).

9. Kolko, J. Broadband and local growth. *Journal of Urban Economics* **71**, 100–113 (2012).

10. Ford, G. S. *Is Faster Better? Quantifying the Relationship between Broadband Speed and Economic Growth*. https://papers.ssrn.com/abstract=3138739 (2018).

11. Whitacre, B., Gallardo, R. & Strover, S. Does rural broadband impact jobs and income? Evidence from spatial and first-differenced regressions. *The Annals of Regional Science* **53**, 649–670 (2014).

12. Chiou, L. & Tucker, C. Social Distancing, Internet Access and Inequality. 27 (2021).

13. Isley, C. & Low, S. Broadband adoption and availability: Impacts on rural employment during COVID-19 - PMC. *Telecomm Policy* **46**, (2022).

14. Bauer, S., Clark, D. D. & Lehr, W. *Understanding Broadband Speed Measurements*. https://papers.ssrn.com/abstract=1988332 (2010).

15. NASHP. States’ COVID-19 Public Health Emergency Declarations and Mask Requirements – The National Academy for State Health Policy. https://www.nashp.org/governors-prioritize-health-for-all/.

16. FCC. Types of Broadband Connections. *Federal Communications Commission* https://www.fcc.gov/general/types-broadband-connections (2014).

17. 21st Century Michigan Infrastructure Comissions. *Michigan Broadband Roadmap*. https://connectednation.org/wp-content/uploads/sites/13/2019/01/Final-Roadmap-8-8-18.pdf (2018).

18. FCC. Household Broadband Guide. *Federal Communications Commission* https://www.fcc.gov/consumers/guides/household-broadband-guide (2020).

19. Ohlsen, L. Y. & Ritzo, C. NDT Data in NTIA Indicators of Broadband Need - M-Lab. https://www.measurementlab.net/blog/ntia/.

20. Isley, C. & Low, S. A. Broadband adoption and availability: Impacts on rural employment during COVID-19. *Telecommunications Policy* **46**, 102310 (2022).

21. Koutroumpis, P. The economic impact of broadband on growth: A simultaneous approach. *Telecommunications Policy* **33**, 471–485 (2009).

22. Abadie, A., Diamond, A. & Hainmueller, J. Synthetic Control Methods for Comparative Case Studies: Estimating the Effect of California’s Tobacco Control Program. *Journal of the American Statistical Association* **105**, 493–505 (2010).

23. Kreif, N. *et al.* Examination of the Synthetic Control Method for Evaluating Health Policies with Multiple Treated Units. *Health Economics* **25**, 1514–1528 (2016).

24. Xu, Y. Generalized Synthetic Control Method: Causal Inference with Interactive Fixed Effects Models. *Polit. Anal.* **25**, 57–76 (2017).
